
JOURNAL INFORMATION
==============================
NLM Title Abbreviation: Innov Surg Sci
Journal ID: Innov Surg Sci
NLM Journal ID: 101708165
Journal ID: iss

Innovative Surgical Sciences

EISSN: 2364-7485
Publisher: De Gruyter

ARTICLE INFORMATION
==============================
PMCID: PMC11005947
DOI: 10.1515/iss-2024-2003
Article ID: iss-2024-2003
Article version: 1
Subjects: Article

Abstracts DGAV

Electronic publication date: 2024 Apr 9
Publication date: 2024 Apr
Volume: 9
Issue: Suppl1
First page: 18
Last page: 203
Copyright: © 2024 by Walter de Gruyter Berlin/Boston
Copyright year: 2024
Copyright holder: De Gruyter
License: This work is licensed under the Creative Commons Attribution 4.0 International License.
License URL: https://creativecommons.org/licenses/by/4.0/

Page count: 186

==============================

**Increasing accessibility to laparoscopic simulator training - Comparison of a low-cost laparoscopic trainer**
ID: 94

Mark Enrik Geissler[1], Jean-Paul Bereuter[1], Rona Berit Geissler[1], Luisa Egen[2], Karl-Friedrich Kowalewski[2], Nathalie Buck[1], Grit Krause-Jüttler[3], Juliane Weiß[1], Jürgen Weitz[1 3 4], Marius Distler[1 3], Florian Oehme[1], Felix von Bechtolsheim[1 3]

[1]*Department of Visceral, Thoracic and Vascular Surgery, Medical Faculty and University Hospital Carl Gustav Carus, Technische Universität Dresden, Dresden, Deutschland*, [2]*Department of Urology and Urosurgery, University Medical Centre Mannheim, University of Heidelberg, Mannheim, Mannheim, Deutschland*, [3]*Centre for Tactile Internet with Human-in-the-Loop (CeTI), Technische Universität Dresden, Dresden, Deutschland*, [4]*National Center for Tumor Diseases (NCT), Medical Faculty and University Hospital Carl Gustav Carus, Technische Universität Dresden, Dresden, Deutschland*

**Background:**
Simulator training is an efficient training tool to develop surgical novice's basic laparoscopic skills. Currently simulator training takes mainly place in simulation centers at hospitals and universities. Additionally, most trainees have to use overtime for training purposes since it is mostly not included during normal working hours. Recent studies also indicated the possibility of low cost at-home training for laparoscopic surgery, opening up new personalized training possibilities. Such modalities support surgeons' initial acquisitions of basic laparoscopic skills as well as continuous development. Furthermore training at home could facilitate the possibility of skill retention, which might be important for longer times without specialized surgical training. This could for example include research time, maternity leave or gaining further education. Nevertheless, most currently available simulators are expensive and require additional equipment (e.g. monitors, stands, PCs) and, therefore, are not feasible for at-home training. Recently developed low-cost simulators show great promise to bridge this gap. We aimed to investigate if low-cost simulators are comparable to more expensive box trainers regarding surgeons performance and likability.

**Materials and methods:**
This randomized, open-label crossover multi-center study was performed at Dresden and Mannheim's university hospitals. Participants were randomly assigned either to the "Laparoscopy Boxx-Pro" (LBX) or "Lübecker Toolbox" (LBT) simulator and switched afterwards. The "Laparoscopy Boxx-Pro" is developed by the Dutch "Laparoscopy Boxx" company. The simulator consists of wooden parts, can be stored and shipped easily and is simple to build up. The simulator costs 309€ including instruments and does not have a built in camera device but allows for the use of a phone or tablet with a build-in camera. The "Lübecker Toolbox" on the other hand is a laparoscopic box trainer produced by the German company "Lübecker Toolbox". This simulator costs about 4,200€ without instruments and a screen. Each participant performed four laparoscopic tasks (Peg transfer, circle cutting, laparoscopic suture and knot, Balloon resection) on both the LBX and LBT. During the tasks, force exertion and completion time were measured using the ForceTrap system (Medishield B.V., Delft, The Netherlands). Additionally, errors were recorded, and the psychological workload was assessed using the NASA-TLX score. Furthermore, participants were tasked to rate the simulator regarding usability, view, and ease of performing the tasks in a self-designed questionnaire using a five-point Likert Skala, with one being the best and five the worst.

**Results:**
In total, seven abdominal surgeons, seven urologists, and sixteen medical students participated in the evaluation of the simulators. Of the 30 participants, the majority (80%) rated their laparoscopic skill level as beginners, whereas six participants (20%) rated their skills as proficient. The PEG task was performed significantly slower using the LBX compared to the LBT (192,1s vs 175,3 s; p=0.049). In contrast, the amount of time for the balloon resection were significantly faster performed with the LBX (compared to the LBT (198,6s vs 223,1s; p=0.049;). Similar, for the surgical knot and suturing taks a

Open Access. © 2024 The Author(s), published by De Gruyter. This work is licensed under the Creative Commons Attribution 4.0 International License.

faster completion time could be observed on the LBX (253,6s vs 293,9s; p=0,029). Regarding force parameters, only the PEG transfer showed significant differences, with the maximum force applied at the LBX being higher compared to the LBT (3,4 N vs 2,9N; p=0.008). The PEG transfer was also rated harder to perform on the ASQ (3.57 vs 2.7; p=0.022). The assessed errors showed no significant differences between both simulators for all four performed excercieses. Nevertheless, the assessment of the simulators showed several differences. The view of the LBX was rated significantly better compared to the LBT (1.9 vs 3.77) (p<0.001). The LBX was also rated significantly better in depth perception (2.27 vs 3.32; p=0.03), but worse regarding the movement of instruments (3.1 vs 2.37; p=0.04). Nonetheless, the pricing of the LBX was rated significantly better compared to the LBT (1.3 vs 4.17; p<0.001). The usage of the LBX for digital training was also rated significantly better than that of the LBT (1.63 vs 3.3; p<0.001).

**Conclusion:**

Surgical simulator training should be accessible and effective in the means of producing sufficient learning curves. Our study showed the comparability of low-cost simulators to more expensive versions. Regarding view, perception, and pricing, the low-cost simulator was rated even superior compared to the high-cost simulator. Nevertheless, the low-cost trainer showed some weakness in instrument movability, which should be improved in the future. The low-cost trainer was assessed as a good option for digital training. This could open new opportunities for continuous training and skill retention outside the hospital. Therefore, low-cost trainers might be feasible to extend the common training environment and allow for more personalized and flexible training, increasing the accessibility of surgical simulator training.

**Evaluation of Serious Gaming in the teaching of the E-FAST-algorithm**
ID: 446

Katharina Althammer[1]

[1]*VTG Chirurgie UKD, Viszeral-, thoracic and vascular surgery , Dresden, Deutschland*

**Background:**
Goal of the ultrasound examination "extended focused assessment with sonography for trauma" -in short E-FAST- is to rule out quickly free fluid in thorax and abdomen preclinically or at the emergency department. Key element of this project is a Serious Game. Serious Gaming combines learning with gaming. The examination is simulated virtually which offers a safe surrounding to practice without harming any patients. The acquired skills can be easily transferred to reality. Being easy and everywhere accessible the game will at best create an addictive character so that the user will want to play the game again.

**Materials and methods:**
For this prospective clinical study two groups of third to sixth year medical students will be randomized. To prove the effectiveness of Serious Gaming the theory of E-FAST is conveyed in one group reading a text compared to the other group playing the Serious Game. Due to the following E-FAST examination on the SonoSimulator a comparison of the teaching methods with determined criteria will be made. A SonoSimulator is a computer connected to a movable ultrasound transducer. It can be used to practice sonography by moving the transducer while seeing virtually within an imaginary body on the computer the sonography monitor.

**Results:**
For now, this project is still in the planning phase. However, at the "Deutscher Chirurgie Kongress 2024" first ideas and results will be presentable.
It is to be expected that Serious Gaming has an advantage over conventional methods of learning such as reading a text. The simulation of reality combined with learning while playing a game will presumably lead to better examination results within the group of students who played the E-FAST-Serious-Game compared to the group of students reading the text about E-FAST.

**Conclusion:**
Serious Gaming offers a fun, fast, always accessible and easy learning aid conveying theoretical knowledge. Medical students would benefit greatly from this learning method. We suggest that Serious Gaming should be included in the medical studies as an additional repetition and practicing tool.
Not only medical students but also expert staff of various specialties could use Serious Gaming on different topics to refresh their knowledge and recap and improve practical skills. Serious Gaming would improve teaching and learning, leading to better skilled doctors and medical staff and by doing so, improving at the end patient care.

**Literature:**
Abdulmajed H, Park YS, Tekian A. Assessment of educational games for health professions: a systematic review of trends and outcomes. Med Teach. 2015 Apr;37 Suppl 1:S27-32. doi: 10.3109/0142159X.2015.1006609. PMID: 25803590.

Helfen, T. (2016). *Notfall- und Rettungsmedizin* (3. Auflage). Elsevier GmbH.

Hempel, D., Pfister, R., & Michels, G. (2018). Strukturierte bettseitige Sonographie in der Intensivmedizin. *Zeitschrift Fur Herz-, Thorax- Und Gefasschirurgie*, *32*(1), 21–38. https://doi.org/10.1007/s00398-017-0201-y

Hofer, M. (2020). *Sono Grundkurs* (M. Hofer, Ed.; 10th ed.). Georg Thieme Verlag KG. https://doi.org/10.1055/b-007-173121

Olgers TJ, Bij de Weg AA, Ter Maaten JC. Serious Games for Improving Technical Skills in Medicine: Scoping Review. JMIR Serious Games. 2021 Jan 25;9(1):e24093. doi: 10.2196/24093. PMID: 33492234; PMCID: PMC7870348.

SonoSim, Inc.. (2023). *SonoSim.* Seen on the 28th of September 2023 on https://sonosim.com/.

Wang Y, Wang Z, Liu G, Wang Z, Wang Q, Yan Y, Wang J, Zhu Y, Gao W, Kan X, Zhang Z, Jia L, Pang X. Application of Serious Games in Health Care: Scoping Review and Bibliometric Analysis. Front Public Health. 2022 Jun 10;10:896974. doi: 10.3389/fpubh.2022.896974. PMID: 35757619; PMCID: PMC9231462.

**Comparison of digital versus in-person training using low-cost surgical simulators- a randomized controlled trial**
ID: 506

Mark Enrik Geissler[1], Jean-Paul Bereuter[1], Rona Geissler[1], Karl Kowalewski[2], Luisa Egen[2], Sofia Schmidt[1], Jürgen Weitz[1 3 4], Marius Distler[1 4], Florian Oehme[1], Felix von Bechtolsheim[1 4]

[1]*Department of Visceral, Thoracic and Vascular Surgery, Medical Faculty and University Hospital Carl Gustav Carus, Technische Universität Dresden, Dresden, Deutschland*, [2]*Department of Urology and Urosurgery, University Medical Centre Mannheim, University of Heidelberg, Mannheim , Deutschland*, [3]*National Center for Tumor Diseases (NCT), Medical Faculty and University Hospital Carl Gustav Carus, Technische Universität Dresden, Dresden, Deutschland*, [4]*Centre for Tactile Internet with Human-in-the-Loop (CeTI), Technische Universität Dresden, Dresden, Deutschland*

**Background:**
Simulation training programs are essential for young surgical residents to acquire basic skills in laparoscopic surgery. The effect of well-structured training curricula on surgical novice skill development has been studied extensively and shown to support laparoscopic skill development. However, current state-of-the-art laparoscopy simulators are still expensive, limiting the accessibility to practical training lessons. Furthermore, training is time intensive and needs building capacities to take place in. Low-cost simulators allow for a cost-effective alternative that still offers training effects and increases the accessibility to simulator training. In addition, simulators encompass new training modalities, such as remote training. This offers personalized surgical training personalized to the surgeons' schedule. Furthermore, it allows for the continuation of training during extended periods outside the operating room. This includes research stays abroad, maternity leave, or illness. Due to a lack of literature concerning the evaluation of at-home training for surgical novices, there is a pressing need to investigate digital training modalities. This study investigates if digital training is comparable to in-person training in developing basic laparoscopic skills in surgical novices. Furthermore, we aim to question novices' views on at-home training and feasibility of this training modality.

**Materials and methods:**
This study is a multi-center, prospective, open-label, randomized trial. Medical students of the medical school Dresden and Heidelberg/Mannheim were randomized to either the digital (intervention) or the in-person (control) group. Students were trained in a standardized laparoscopic training curriculum using the "Laparascopy Boxx-pro" simulator until they reached a predefined level of proficiency . After completing the curriculum, participants performed four different laparoscopic tasks (PEG transfer, surgical cutting, surgical knot and suture, and balloon resection). The "Force Sense" system (Medishield B.V., Delft, The Netherlands) was used for the final skill assessment, during which students had to perform all four exercises. Primary endpoints were maximum force, mean non-zero force, task time, error rate, and the overall force. Furthermore, the NASA-TLX score was utilized to assess the psychological workload during the task performance. In addition, students were asked to complete a self-designed questionnaire to perceive their view on digital simulation training.

**Results:**
In total, 40 laparoscopic novices participated from April to August 2023 and reached proficiency in basic laparoscopic skills. All students performed the four tasks in their final assessment. Regarding the rate of errors, there were no significant differences between the group with digital training and the group trained in person for all four exercises. In addition, there were no observable differences in the applied mean non-zero force or maximum force. Interestingly, the digital group performed the suturing and knot-tying task significantly faster than the in-person group (digital: 319.6s; in-person: 419.4s; $p = 0.015$). Besides, no significant differences in completion time for the other tasks were observed.
Regarding students rating of skill development overall the majority of students rated their skills to have improved (92.5%). There were no significant differences in students rating of the training quality throughout the course between both groups (digital: 94.7%; in-person: 90.5%; $p = 0.364$). Furthermore, there were also no significant differences in students rating of the ability to follow the tutor’s instructions (digital: 84.2%; in-person: 85.8%; $p = 0.756$) and observability of instrument movements (digital: 84.2%; in-person: 81%; $p = 0.364$). The majority of participants in both groups (84.3%) stated that online teaching is helpful in surgical training. Students also agreed that simulator training is essential for them (85%) and stated that online simulator training supports the skill development of surgical residents (85%) with no significant differences between the training groups.
Interestingly, all students (100%) in the in-person group would have wanted to be training in this modality, whereas nearly half of the students in the digital group (42.1%) would have also wanted to be trained in-person ($p < 0.001$).

**Conclusion:**
This study investigated the suitability of the training basic laparoscopic sills in a digital and in-person setting. The digital training setting showed no inferiority to the in-person setting in the development of basic laparoscopic skills. Therefore digital training proves to be a sufficient alternative to in-person training. This is especially important to consider if trainees are not able to attend simulation centers. Digital training overcomes this challenge and offers personalize training modalities, increasing the accessibility to surgical training. Digital training could be used as a sufficient alternative for trainees who cannot or do not want to attend in-person training. However, in-person training has shown to be more attractive for most untrained participants and therefore should be the focus of training providers. Nonetheless further research is needed to elucidate the best possible fit for digital training.

### Establishing a Student Minimal Invasive Surgical Simulation Laboratory for Research and Education

ID: 533

Hans Christian Schmidt[1], Pauline Schuppert[1 2], Lucie Rohwäder[1], Tobias Bokowski[3], Kira Meißner[3], Saman Keshtkaran[3], Abdulrahman Al-Harazi[3], Fabian Schönewald[3], Hagen Pöhlmann[3], Merle Juschka[3], Vu Khang Mervin Do[3], Amila Cizmic[4], Felix Nickel[4], Tobias Dust[5], Andreas Lindner[6], Oliver Muensterer[6], Konrad Reinshagen[1], Christian Tomuschat[1]

[1]*University Medical Center Hamburg-Eppendorf, Department of Pediatric Surgery*, [2]*Martin-Luther-Universität Halle-Wittenberg*, [3]*University Medical Center Hamburg-Eppendorf*, [4]*University Medical Center Hamburg-Eppendorf, Clinic and Policlinic for General-, Visceral- and Thoracic surgery*, [5]*University Medical Center Hamburg-Eppendorf, Clinic and Policlinic for Trauma Surgery and Orthopedics*, [6]*Dr. von Hauner Children´s Hospital, Ludwig-Maximilians-University Medical Center, Munich, Department of Pediatric Surgery*

**Background:**

In experienced hands laparoscopy reduces trauma to the abdominal wall, lowers blood loss and postoperative complications [1, 2]. However, it entails a steep learning curve [3, 4], highlighting the need of early-stage training. This initiative focuses on establishing a state-of-the-art surgical simulation laboratory with the focus on pediatric surgery at the University Medical Center Hamburg-Eppendorf. As surgery is a craft best learned through hands-on experience, we aim to refine surgical expertise through hands-on training. To foster a culture of hands-on learning, our student research group was formed and is supported by experienced cooperations. While theoretical instruction in surgery is provided at universities, there is a growing demand for the improvement of practical training. Our objective is to enhance the proficiency of medical students and physicians in the application of minimally invasive techniques (MIT), commencing with laparoscopic models made by medical students. Furthermore, we aim to institute a self-sustaining system, driven by generations of students passionate about surgery, leading to improved early-stage surgical skills and in the long-term optimized patient outcomes post-surgery.

**Materials and methods:**

The project encompasses training in laparoscopic surgery, spanning from the innovative self-designed neonatal Puppet (SuSiPed) [5] to commercially available adult-sized boxes. Student tutors will receive surgical training by experienced professionals. The team and tools serve as platforms for instructing students in basic modules, including camera navigation, instrument handling, cylinder stacking, cutting exercises, and knot tying techniques. Additionally, we transform 3D-datasets derived from MRI- and CT-scans in 3D-printable files to create realistic anatomical models and replicas of disease pathologies. Through the application of 3D printing and silicone molding, we will be able to offer advanced modules such as cyst resection, appendectomy, models for intestinal anastomoses, and more at low cost, which can also be used by advanced surgeons.

**Results:**

The enclosed puppets or boxes contain training modules, visualized through a 2D camera transmitting to a screen, necessitating operators to adapt to this spatial constraint while operating in a 3D environment. This presents a notable challenge especially for less experienced participants. Small proportions in pediatric surgery makes MIT more difficult to learn but can be practiced with this model. Training models were built from 3D models without instructions, following the example of SuSiPed. It appears comprehensible that a positive learning trend will be observed. We hypothesize increased motivation for the basic modules, while the participants anticipate the opportunity to tackle more realistic modules, e.g. laparoscopic appendectomy and other custom-created pathological models. However, evaluations regarding motivation and learning curves are content of future evaluation.

**Conclusion:**

To our knowledge we are the first student group establishing a surgical platform for the training of MIT in pediatric surgery and hope to encourage other students to start MIT programs. We highlight the necessity of cooperative professions to utilize the potential of motivation in the group of surgically interested students. This initiative will not only advance students' surgical skills but also equips tutors with pedagogical and communication experiences, contributing in long-term to the optimization of patient outcomes.

**References**

1. Buia, A., Stockhausen, F. & Hanisch, E. Laparoscopic surgery: A qualified systematic review. *World J Methodol* **5,** 238-54 (2015).
2. Lou, S., Yin, X., Wang, Y., Zhang, Y. & Xue, Y. Laparoscopic versus open gastrectomy for gastric cancer: A systematic review and meta-analysis of randomized controlled trials. *Int J Surg* **102,** 106678 (2022).
3. Brown, K. M. & Geller, D. A. What is the Learning Curve for Laparoscopic Major Hepatectomy? *J Gastrointest Surg* **20,** 1065-71 (2016).
4. Cizmic, A. et al. Telestration with augmented reality improves the performance of the first ten ex vivo porcine laparoscopic cholecystectomies: a randomized controlled study. *Surg Endosc* **37,** 7839-7848 (2023).
5. Weisser, N. et al. SuSiPed: An initial step towards a universal, low-cost, 3D-printable platform for pediatric minimal-invasive surgery training. *J Pediatr Surg* **58,** 675-678 (2023).

**Feasibility and outcome of virtual reality based topographic surgical anatomy teaching in medical students**
ID: 711

Jan Philipp Ramspott[1], Alexander Bungert[1], Isabelle Flammang[1], Felix Becker[1], Carsten Szardenings[2], Andreas Pascher[1], Morgül Haluk[1]

[1]*Department for General, Visceral and Transplant Surgery, University Hospital Muenster, Muenster, Deutschland*, [2]*Institute of Biostatistics and Clinical Research, University of Muenster, Muenster, Deutschland*

**Background:**
The need for precise, effective medical education that meets the expectations of medical students for a thorough education has led to technological developments in medical teaching strategies. Virtual reality (VR) simulation, which renders a fully computer-generated three-dimensional (3D) multimedia environment in real time has emerged as a promising teaching tool. Here, we investigated the feasibility, efficacy, and educational value of VR in medical students training for both topographic surgical anatomy and the subsequent surgical procedure in a clinical scenario. In addition, we investigated the impact of VR-based education on career choice.

**Materials and methods:**
Virtual dissection was performed using the Meta Quest 2 VR system (Meta, California, USA). The VR system included a virtual headset worn over the eyes and two wrist and touch motion controllers. Both Anatomy Master and Dissection Master XR software applications were provided by Medicalholodeck, Switzerland. The feasibility, efficacy, and educational value of VR were assessed using a 14-item questionnaire. Statistical analysis was performed using Fisher's exact test and Wilcoxon-Mann-Whitney test.

**Results:**
After a tutorial session with a VR-experienced tutor, 20 medical students (80% final year medical students) performed a dissection of a virtual 3D patient to display an anatomical 3D view of the upper gastrointestinal tract. The tutor virtually presented the surgical procedure for esophageal cancer resection. The final evaluation revealed eight students (40%) with previous VR experience. The median VR-usefulness score was nine (0=useless, 10=very useful). Almost all participants reported that VR-based topographic surgical anatomy training added a high value to classical teaching (median: ten; 0=none, 10=very much). In addition, all students reported that the simulated anatomical structures resembled with the properties of human tissue of their dissection class. Fifteen percent of all students began to consider a future surgical career after the VR session. Statistical analysis did not reveal any differences in scores between students of different ages or educational levels.

**Conclusion:**
Our results indicate that VR-based teaching of topographic surgical anatomy and the subsequent surgical procedure is suitable for medical students at all levels. Although limited by the number of subjects, the current study suggests that VR training adds value to traditional preparatory anatomical models. Further studies are needed to validate our findings and to explore the extent to which VR and augmented reality-based instruction could become a fundamental tool for medical and surgical education.

Abstracts – DCK 2024 – 141st Congress of the German Society of Surgery – Leipzig, April 24–26 • DOI 10.1515/iss-2024-2003
Innov Surg Sci 2024; 9 (Special Suppl 1): s18–s203

**Bariatric surgery counteracts insulin dependency of adipose patients under the simultaneous significant reduction of mTOR gene expression**
ID: 78

Laura Feiner[1], Sebastian Holländer[1], Matthias Glanemann[1], Claudia Rubie[1]

[1]*Universitätsklinikum des Saarlandes, Klinik für Allgemein-, Viszeral-, Gefäß- und Kinderchirurgie, Homburg, Deutschland*

**Background:**
The mechanistic target of rapamycin (mTOR) is known as a key control element of crucial regulatory pathways and a modulator of metabolic processes. The aim of the present study was to investigate our observation that the demand of anti-diabetic medication in adipose patients rapidly declines after bariatric surgery. We speculated that mTOR signalling may be involved in this process and analyze mTOR gene expression with respect to the effect of bariatric surgery on the insulin dependency of adipose patients.

**Materials and methods:**
Based on real time PCR mTOR gene expression was monitored in Peripheral Blood Mononuclear Cells (PBMC) from adipose patients (median BMI: 57 kg/m2; median age: 42) and healthy age- and BMI matched controls (median BMI: 22 kg/m2; median age: 35). We investigated mTOR gene expression in adipose patients preoperatively before crash diet and after crash diet just before the operation. Further study time points post-operatively included 1 day after the operation as well as 3 and 5 days and 3, 6 and 12 months after the operation.

**Results:**
Pre-operatively mTOR gene expression was revealed to be significantly elevated in the adipose patients in comparison to our control cohort. mTOR gene expression did not change significantly after crash diet in the adipose cohort. However, directly after the bariatric surgery on Post-OP day 1, a significant 2-fold decrease of mTOR expression was monitored. This effect was seen throughout all post-operative study time points. Most importantly, post-operatively we also observed a significant reduction of anti-diabetic medication in type 2 diabetes mellitus (T2DM) in our adipose patient cohort.

**Conclusion:**
We demonstrated that bariatric surgery counteracts insulin dependency of adipose patients while simultaneously mTOR gene expression was significantly reduced. We hypothesize that the mTOR signalling pathway may be involved in this metabolic process leading to the significant post-operative reduction of anti-diabetic medication in adipose patients who underwent bariatric surgery.

**Hypothalamic integrity is necessary for sustained weight loss after bariatric surgery: A prospective, cross-sectional study**
ID: 611

Ulrich Dischinger[1], Ann-Cathrin Koschker[1], Mohamed Hankir[2], Christoph-Thomas Germer[2], Martin Fassnacht[1], Florian Seyfried[2]

[1]*Department of Internal Medicine, Division of Endocrinology and Diabetes, University Hospital, University of Würzburg, Würzburg, Deutschland*, [2]*Department of General, Visceral, Transplant, Vascular and Pediatric Surgery, , University Hospital, University of Würzburg, Germany, Würzburg, Deutschland*

**Background:**
The hypothalamus is the main integrator of peripheral and central signals in the control of energy homeostasis. Its functional relevance for the effectivity of bariatric surgery is not entirely elucidated. Studying the effects of bariatric surgery in patients with hypothalamic damage might provide insight.

**Materials and methods:**
65 participants were included (HO-surgery: n = 8, HO-control: n = 10, CO-surgery: n = 12, CO-control: n = 12, Lean-control: n = 23). Body weight, levels of anorexic hormones, gut microbiota, as well as subjective well-being/health status, eating behavior, and brain activity (via functional MRI) were evaluated.

**Results:**
Patients with HO lost significantly less weight after bariatric surgery than CO-participants (total body weight loss %: 5.5 % vs. 26.2 %, p = 0.0004). After a mixed meal, satiety and abdominal fullness tended to be lowest in HO-surgery and did not correlate with levels of GLP-1 or PYY. Levels of PYY (11,151 ± 1667 pmol/l/h vs. 8099 ± 1235 pmol/l/h, p = 0.028) and GLP-1 (20,975 ± 2893 pmol/l/h vs. 13,060 ± 2357 pmol/l/h, p = 0.009) were significantly higher in the HO-surgery vs. CO-surgery group. Abundance of Enterobacteriaceae and Streptococcus was increased in feces of HO and CO after bariatric surgery. Comparing HO patients with lean-controls revealed an increased activation in insula and cerebellum to viewing high-caloric foods in left insula and cerebellum in fMRI.

**Conclusion:**
Hypothalamic integrity is necessary for the effectiveness of bariatric surgery in humans. Peripheral changes after bariatric surgery are not sufficient to induce satiety and long-term weight loss in patients with hypothalamic damage.

### Reflux Symptoms and Quality of Life after Fundoplication versus Roux-en-Y Gastric Bypass for GERD

ID: 790

Johanna Betzler[1 2], Nina Wiegand[1], Alida Finze[1], Sebastian Schölch[1 2], Christoph Reißfelder[1], Mirko Otto[1], Susanne Blank[1]

[1]*University Medical Center Mannheim, Department of Surgery, Mannheim, Deutschland*, [2]*German Cancer Research Center (DKFZ) Heidelberg, Junior Clinical Cooperation Unit Translational Surgical Oncology, Mannheim, Deutschland*

**Background:**

Gastroesophageal reflux disease (GERD) is a globally prevalent disease that shows rising incidence, particularly in nations with high obesity prevalence. Surgical intervention is considered for refractory GERD cases. In non-obese patients, laparoscopic fundoplication is the preferred surgical method, whereas in individuals with BMI >40 kg/m2 and GERD, laparoscopic Roux-en-Y gastric bypass (RYGB) is favored due to its concurrent weight reduction effects. Currently, there is no nationally standardized recommendation for patients with GERD and obesity WHO grade I and II (BMI 30-39,5 kg/m2).

**Materials and methods:**

Data of all patients who underwent surgery for GERD between January 2018 and October 2021 at University Hospital Mannheim was collected retrospectively. Patients were divided into two cohorts according to operative technique (Fundoplication vs. RYGB), and their outcomes compared.
To evaluate quality of life after reflux and bariatric surgery, BQL and QOLRAD questionnaires were analyzed. To evaluate symptom reduction after surgery the RSI questionnaire was used.
In a separate analysis, patients were propensity score matched according to BMI, age and gender.

**Results:**

115 patients (65.8% females and 34.2% males) were included in this study. Of those, 76 patients underwent laparoscopic fundoplication (52 patients Toupet fundoplication, 9 patients Nissen fundoplication) and 39 patients underwent laparoscopic RYGB.
45.6% of the patients were obese whereas 54.4% were normal weight (75.4%) or pre-obese (24.6%). Of the obese patients 46.1% had obesity WHO grade I (BMI 30-34,9 kg/m2) and 51.9% had obesity WHO grade II (BMI 35-39,9 kg/m2).
For 42.74% of all patients pre- and postoperative results of RSI questionnaires were available. Results of BQL questionnaires were available for 41.5% and results of QOLRAD questionnaire were available for 41.1% of the patients.
The results of propensity-score matching and the analysis of the questionnaires are currently in statistic evaluation and will be presented at the conference.

**Conclusion:**

RYGB is an important alternative to fundoplication for refractory GERD in mild to moderate obese patients and should be considered as a standard operative procedure for this indication.

Abstracts – DCK 2024 – 141st Congress of the German Society of Surgery – Leipzig, April 24–26 • DOI 10.1515/iss-2024-2003
Innov Surg Sci 2024; 9 (Special Suppl 1): s18–s203

### Initiating a robotic bariatric program in a tertiary hospital: early experience

ID: 855

Vasiliki Christogianni[1], Ashwini Rao[1], Christian Jörn Halter[1], Mohanned Raed[1], Panagiotis Bemponis[1], Martin Büsing[1]

[1]*Klinikum Vest, Surgery, Recklinghausen, Deutschland*

**Background:**

Although surgical robotic systems offer many technical advantages, the implementation in well-established laparoscopic bariatric centers is still controversial.

**Materials and methods:**

This is a retrospective study of all consecutive patients undergoing robotic bariatric surgery from July 2023 to September 2023. Demographic data, operative method, technical characteristics of the procedures as also hospital stay, and complications were analyzed.

**Results:**

Between 07.07.2023 and 09.10.2023 a total of 71 bariatric patients were operated using the daVinci Xi system (Intuitive Surgical, Sarl). 62 underwent a primary sleeve gastrectomy (SG) and 9 a secondary bariatric procedure. All operations were conducted from two well experienced laparoscopic surgeons with no previous robotic skills.

From the SG group, demographics were as followed: mean ASA (American society of anesthesiologists) score 2.5 (+0.5), mean body mass index (BMI) 48.7 (±7.2), prevalence of hypertonus 45.2%, of T2DM (type II diabetes mellitus) 16.1% and of OSA (obstructive sleep apnea) 30.6%.

Regarding the operative time, mean overall was 58.9 (±20) mins., from which 3.2 (±1.1) mins. for the incision and the establishment of the pneumoperitoneum, 3.6 (±1.6) mins for the docking of system, 41.6 (±14.9) mins for the robotic part of the procedure and 6.2 (±1.8) for the removal of the resected part of the stomach and the closing of the incisions. Active operative time using the instruments was 33.2 (±11.3) mins. Instruments were changed 4.6 (±2.2) times. Mean NRS (numeric rating scale) pain score at the first postoperative day was 1.6 (±0.8) and mean hospital stay 2.1 (±1.1) days. No intraoperative complications occurred. One patient was readmitted at the 3. postoperative day due to excess vomiting. Comparing the first and the last 10 cases, there a statistically significant improvement on the incision, docking and operative time.

From the second group, all 9 subjects had an index SG as their primary bariatric procedure. 2 patients received a resizing of the sleeve, 3 were converted to RYGB (Roux-en-Y gastric bypass), 3 to OAGB (one anastomosis gastric bypass) and one received a transit bipartition. Except one, all other patients had an uneventful postoperative course. One patient who was converted to RYGB needed to be reoperated duo to an incarcerated incisional hernia.

**Conclusion:**

Establishing a robotic bariatric program in a previously well-trained laparoscopic setting is feasible within a few months. Even more complexed procedures can be undertaken without compromising clinical outcomes.

**Patient-related outcomes in rigid versus flexible proctosigmoidoscopy – a randomized controlled trial**
ID: 404

Peter Wilhelm[1 2], Johanna Miller[3], Leonie Ritz[2], Steffen Axt[2], Jonas Johannink[2], Kai Jansen[3], Andreas Kirschniak[2 3], Jens Rolinger[2 3]

[1]*End- und Dickdarmpraxis Sindelfingen, Sindelfingen, Deutschland*, [2]*Universitätsklinik für Allgemeine, Viszeral- und Transplantationschirurgie, Universitätsklinikum Tübingen, Tübingen, Deutschland*, [3]*Klinik für Allgemein- und Viszeralchirurgie, Kliniken Maria Hilf GmbH, Mönchengladbach, Deutschland*

**Background:**
Endoscopic rectal examinations play a crucial role in diagnosing and managing various colorectal disorders, including colorectal and anal cancer, inflammatory bowel disease, and other benign anorectal conditions. Two primary modalities, rigid and flexible proctosigmoidoscopy, provide essential diagnostic information for accurate diagnosis and treatment planning. Historically, rigid proctosigmoidoscopy has been considered the gold standard due to its simplicity, reliability, and cost-effectiveness. However, the emergence of flexible sigmoidoscopy has introduced new possibilities for enhancing patient experience and diagnostic capabilities, including video display of findings and the ability to perform an inversion of the device. Despite these advancements, the choice between rigid and flexible proctosigmoidoscopy remains a subject of debate. The literature on this topic is sparse and mostly refers to outdated publications. This study aims to contribute to the ongoing discussion by evaluating patient satisfaction and in particular perceived pain associated with both rigid and flexible proctosigmoidoscopy.

**Materials and methods:**
Participants presented as outpatients at the consultation for colorectal and pelvic floor surgery at the Department for General, Visceral and Transplant Surgery, Tübingen University Hospital. Patients were eligible for inclusion if they exhibited symptoms or suspected medical conditions warranting diagnostic proctosigmoidoscopy, while exclusion criteria encompassed underage patients, cases involving oncologic evaluations, known anal canal stenosis, and recent resections in the rectosigmoid junction. Subjects were randomly assigned to either the rigid or flexible proctosigmoidoscopy group. Standard endoscopic equipment (Karl Storz SE & Co. KG, Tuttlingen, Germany), was used, including a rigid rectoscope per HEINKEL specifications (20 mm diameter, 25 cm working length) with video adapter and air insufflation, as well as the flexible SILVER SCOPE® rectoscope per TROIDL specifications (12 mm diameter, 40 cm working length). During subsequent proctosigmoidoscopy, conducted with the patient in a lithotomy position, did not involve therapeutic interventions. Following the examination, a standardized study questionnaire was completed by both the examiner and the patient. In addition to dichotomous questions, a numerical ten-level Likert-type response scale (1 = "no pain," 10 = "most severe pain") was used to assess participants' subjective perception of pain.

**Results:**
Between December 2019 and July 2021, a total of 48 patients participated in this study, comprising 30 females and 18 males. The median age was 57 years (range 19 - 89 years). No significant differences in gender or age distribution were observed between the two groups ($p = 0.233$ and $p = 0.680$, respectively). Regarding the subjectively perceived pain during proctosigmoidoscopy, neither the qualitative (pain vs. no pain) nor the quantitative (pain severity on the numerical rating scale) analysis revealed statistically significant differences between the two study arms ($\chi^2 = 0.087$, $p = 0.768$ and $U = 288.000$, $p = 1.0$, respectively). Irrespective of group affiliation, 15 of 23 patients who had reported pain during digital-rectal examination in the qualitative analysis also reported pain during proctosigmoidoscopy. A statistically significant correlation was observed between the qualitatively reported pain during digital-rectal examination and the qualitatively as well as quantitatively reported pain during proctosigmoidoscopy ($\chi^2 = 12.134$, $p < 0.001$, $\varphi = 0.503$ and $U = 136.500$, $p < 0.001$, respectively). In 2 of 48 patients, the respective investigator reported technical difficulties during the examination procedure, all of whom belonged to the "flexible sigmoidoscopy" group. No statistically significant association was found between group assignment and technical difficulties ($p = 0.489$). None of the examined patients ($n = 48$) experienced complications analogous to the Dindo-Clavien classification that would have altered the patient's further course of treatment in any way (corresponds to Dindo-Clavien ≥ 1).

**Conclusion:**
In summary, this randomized controlled trial found that both rigid and flexible proctosigmoidoscopy, when performed with a modern endoscopic setup, exhibit comparable levels of patient acceptance and subjective pain perception. Rigid endoscopy offers simplicity of performance, while flexible endoscopy provides greater insertion depth. Proctological examiners should take into account the individual pain perception of patients during digital-rectal examination for the subsequent examination course. Patient satisfaction and comfort during rectal examinations are crucial for ensuring compliance with screening protocols and early detection of colorectal diseases.

**Bariatric surgery inhibits primary colorectal carcinoma growth and significantly prevents hepatic metastasis in a mouse model. An approach for the future?**
ID: 41

Claudia Läßle[1], Bernhard Mauerer[1], Lisa Marx[1], Gabriel Seifert[1], Goran Marjanovic[1], Luisa Schäfer[1], Stefan Fichtner-Feigl[1], Rebecca Kesselring[1]

[1]*Uniklinik Freiburg - Allgemein- und Viszeralchirurgie, Freiburg im Breisgau, Deutschland*

**Background:**
Colorectal cancer (CRC) is the seventh leading cause of death in the Western world. Early incidence as well as distant metastasis are associated with more aggressive tumors and a significantly worse outcome. Overweight and obesity are associated with earlier incidence of metastasis. Valid and detailed quantification of the additional risk associated with preexisting obesity is difficult in clinical trials. Metabolic-bariatric surgery (MBS) has been shown to reduce the incidence of CRC in large cohort studies. Post-bariatric beneficial gut microbiome alterations as well as bile acid metabolic changes may be important factors underpinning anti-tumoral effects. Current oncologic therapy strategies target the tumor cell itself. Strategies for the prevention of metastasis via targeting circulating tumor cells or the pre-metastatic niche are currently unavailable. In this project, we aimed to investigate in which way and by which mechanisms bariatric surgery impacts primary colorectal tumor growth and secondary metastasis. The primary endpoints are primary tumor growth ($mm^3$) and hepatic metastasis (n) with and without RYGB.

**Materials and methods:**
We established a high fat diet (HFD) mouse model of obesity and bariatric surgery (Roux-en-Y-Gastric Bypass (RYGB)). We combined this model with a murine organoid CRC model to study primary CRC growth and metastasis in a post-bariatric metabolic state. We used an HFD as well as a normal chow (NC) group with sham surgery as control groups. To understand the role of systemic bile acids in this process, we compared results to isolated bile diversion in an additional model. We analyzed the intra-tumoral and intra-luminal microbiome as well as metabolomic analysis of portal vein blood.

**Results:**
Mice with RYGB showed significantly lower primary tumor growth as well as almost no hepatic metastasis during the progression of the murine CRC model compared to both control groups (HFD and NC). Post-bariatric weight loss alone was shown to be not significant in the process of primary and secondary carcinogenesis with a significant higher tumor burden in NC control group as RYGB group. The post-bariatric intra-tumoral microbiome was also not different from the intra-tumoral microbiome of the control groups, whereas the fecal microbiome was significantly different. The secondary systemic bile acids were significantly different in the RYGB group. To verify the hypothesis that secondary bile acids have a protective effect on CRC metastasis, a separate animal model of isolated bile diversion, similar to a biliodigestive anastomosis in humans, was combined with the organoid CRC model. Here, the same protective effects on primary and secondary CRC carcinogenesis were seen as after bariatric surgery.

**Conclusion:**
RYGB as well as bile diversion surgery are associated with lower tumor burden as well as decreased hepatic metastasis. Systemic secondary bile acids seem to have a protective effect.

**Freiburg Colorectal Cancer Microbiome Metastasis (REBALANCE) - a prospective trial**
ID: 124

Christian Hillebrecht[1], Bernhard Mauerer[1], Stefan Fichtner-Feigl[2], Melanie Börries[2], Alexandra Nieters[3], Rebecca Kesselring[1], Philipp Holzner[1], Christopher Berlin[1]

[1]*Universitätsklinikum Freiburg, Klinik für Allgemein- und Viszeralchirurgie, Freiburg im Breisgau, Deutschland*, [2]*Universitätsklinikum Freiburg, Institute of Medical Bioinformatics and Systems Medicine, Freiburg im Breisgau, Deutschland*, [3]*Uniklinik Freiburg - Centrum für Chronische Immundefizienz - CCI, Freiburg im Breisgau, Deutschland*

**Background:**
Colorectal cancer (CRC) is the third most frequently diagnosed malignant disease and second leading cause of cancer death with numbers expected to rise further[1]. Extent of disease at diagnosis is predictive of survival, locoregional and systemic spread dramatically reduce chances of survival. More than 30% of patients with CRC will present with syn- or metachronous liver metastases (LM) or peritoneal carcinomatosis (PC) according to population-based studies[2]. Tumour microbiome plays a role in carcinogenesis, progression and chemoresistance and even influences prognosis not just in CRC[3, 4]. Mechanistically, this is supposedly mediated through pro-inflammatory and immunosuppressive properties[5, 6]. With numerous studies linking microbiota to the development of primary CRC, their role in metastasis is only beginning to be understood. There is evidence that these bacteria can be intracellular[7, 8] and that these intracellular tumour microbiota can be transported with circulating tumour cells (CTCs) to metastatic sites and even enhance metastatic potential of CTCs[9]. Though links between tumour microbiome and CRC/metastases have been demonstrated[10], it is still unclear, what role the faecal microbiome plays and what environmental factors predispose to unfavourable microbiota. Dysbiosis could be caused by environmental and lifestyle factors such as living conditions, diet, antibiotic consumption[6, 11]. A better grasp of the faecal microbiome and its role for tumour progression, metastasis and recurrence has potential clinical implications for patients with CRC: preventive strategies, early detection or monitoring methods could be devised; therapeutically, detection of dysbiosis in patient samples could allow for targeted therapy and modification of intrinsic microbiome could improve efficacy of immunotherapy.
To evaluate the link between individual and environmental factors and non-tumour (faecal/intestinal) and tumour/metastasis microbiota, we have initiated the longitudinal F**reib**urg colorect**al** c**an**cer mi**c**robiome m**e**tastasis (REBALANCE) trial

**Materials and methods:**
REBALANCE has been approved by the institutional board of ethics and prospectively registered (Ethik-Kommission der Universität Freiburg No.: 21-1162_3; FRKS004149; DRKS00028615). Patients with CRC with syn-, metachronous or recurrent LM or PC presenting for surgical resection are included. After informed consent, samples of PT, LM, PC, blood, and stool are collected at different time points in cooperation with the Biobank of the Medical Center of the Freiburg University (FREEZE). 16S rRNA is extracted and sequenced for taxonomical profiling. Extensive questionnaires (devised in cooperation with Freiburg Center for Chronic Immunodeficiency (CCI) are used to screen for historical modifiers of intestinal and tumour or metastasis microbiome. Clinical and oncological follow-up data are recorded. The influence of personal and environmental factors on tumour/metastasis microbiome and oncological course (DFS/RFS/OS) will be explored. Concurrent single-cell RNA sequencing (scRNASeq) data from PT and LM samples will help elucidate microbiome-driven alterations in the TME.

**Results:**
Reverse translational research has shown the presence and transport of microbiota between primary tumour and metastasis via CTCs to be associated with pro-metastatic properties such as formation of pre-metastatic niches (PMN) and enhanced immune-escape due to changes in the tumour-microenvironment. Microbial diversity between primary tumour and metastases was predictive of patient survival and a stable microbiome (e.g. via CTC transport) was associated with worsened survival (Feuerstein, Berlin et al., currently under review).
The prospective trial will serve to identify historical modifiers of intestinal/tumoral microbiota. The above-mentioned findings will also be validated in this prospective cohort and a local cohort of healthy individuals will serve as validation. Further collaboration and a multi-centre approach are in the planning stage, we will cooperate with other (among others DKTK partner sites) centres.
Through immunomodulation and changes in the tumour microenvironment, tumour-intrinsic microbiota influence survival from colorectal liver metastasis.

**Conclusion:**
This trial will help gather a better grasp of the faecal microbiome and its role for tumour progression, metastasis and recurrence which has potential clinical implications for patients with CRC. Identification of risk factors and unfavourable faecal microbiota could lead to preventive measures, early or relapse detection. Dietary or environmental modifications could potentially alter the intestinal and therefore tumour microbiome which could in turn reduce the risk of recurrence or metastasis. Therapeutically, detection of microbiota in patient samples could influence management and treatment stratification, modifications of tumour and metastasis microbiome could improve efficacy of immunotherapy.
We have designed and launched a prospective trial to validate our reverse translational findings, a multicentric approach is planned.

1. Sung H, Ferlay J, Siegel RL, et al. Global Cancer Statistics 2020: GLOBOCAN Estimates of Incidence and Mortality Worldwide for 36 Cancers in 185 Countries. *CA Cancer J Clin*. May 2021;71(3):209-249. doi:10.3322/caac.21660
2. Hackl C, Neumann P, Gerken M, Loss M, Klinkhammer-Schalke M, Schlitt HJ. Treatment of colorectal liver metastases in Germany: a ten-year population-based analysis of 5772 cases of primary colorectal adenocarcinoma. *BMC Cancer*. Nov 4 2014;14:810. doi:10.1186/1471-2407-14-810
3. Arthur JC, Perez-Chanona E, Muhlbauer M, et al. Intestinal inflammation targets cancer-inducing activity of the microbiota. *Science*. Oct 5 2012;338(6103):120-3. doi:10.1126/science.1224820
4. Yu T, Guo F, Yu Y, et al. Fusobacterium nucleatum Promotes Chemoresistance to Colorectal Cancer by Modulating Autophagy. *Cell*. Jul 27 2017;170(3):548-563 e16. doi:10.1016/j.cell.2017.07.008
5. Mima K, Sukawa Y, Nishihara R, et al. Fusobacterium nucleatum and T Cells in Colorectal Carcinoma. *JAMA Oncol*. Aug 2015;1(5):653-61. doi:10.1001/jamaoncol.2015.1377
6. Gur C, Ibrahim Y, Isaacson B, et al. Binding of the Fap2 protein of Fusobacterium nucleatum to human inhibitory receptor TIGIT protects tumors from immune cell attack. *Immunity*. Feb 17 2015;42(2):344-355. doi:10.1016/j.immuni.2015.01.010
7. Nejman D, Livyatan I, Fuks G, et al. The human tumor microbiome is composed of tumor type-specific intracellular bacteria. *Science*. May 29 2020;368(6494):973-980. doi:10.1126/science.aay9189
8. Bullman S, Pedamallu CS, Sicinska E, et al. Analysis of Fusobacterium persistence and antibiotic response in colorectal cancer. *Science*. Dec 15 2017;358(6369):1443-1448. doi:10.1126/science.aal5240
9. Fu A, Yao B, Dong T, et al. Tumor-resident intracellular microbiota promotes metastatic colonization in breast cancer. *Cell*. Apr 14 2022;185(8):1356-1372 e26. doi:10.1016/j.cell.2022.02.027
10. Flemer B, Lynch DB, Brown JM, et al. Tumour-associated and non-tumour-associated microbiota in colorectal cancer. *Gut*. Apr 2017;66(4):633-643. doi:10.1136/gutjnl-2015-309595
11. Knoop KA, McDonald KG, Kulkarni DH, Newberry RD. Antibiotics promote inflammation through the translocation of native commensal colonic bacteria. *Gut*. Jul 2016;65(7):1100-9. doi:10.1136/gutjnl-2014-309059

**Outcome and long-term quality of life after multi-organ resection of locally advanced Gastrointestinal Stromal Tumors (GIST)**
ID: 229

Julian Musa[1 2 3], Sarah Kochendörfer[1], Franziska Willis[1 2], Mohammed Al-Saeedi[1], Martin Schneider[1 2]

[1]*Department of General, Visceral, and Transplantation Surgery, University Hospital Heidelberg, Deutschland*, [2]*Current address: Department of General, Visceral, Thoracic, Transplantation, and Pediatric Surgery, University Hospital Giessen and Marburg, Campus Giessen, Deutschland*, [3]*Department of Translational Pediatric Sarcoma Research, German Cancer Research Center (DKFZ) Heidelberg, Deutschland*

**Background:**
Resection of Gastrointestinal Stromal Tumors (GIST) represents the gold standard of primary therapy in cases of localized tumor stage. The significance of extensive resections in locally advanced GIST remains uncertain, particularly when considering neo-adjuvant Tyrosine Kinase Inhibitor (TKI) treatment. The aim of this study is to investigate postoperative morbidity, oncological outcomes, and postoperative long-term quality of life in patients undergoing multi organ resections (MOR) versus single organ resections (SOR) for locally advanced GIST.

**Materials and methods:**
A cohort of 160 primary GIST resected at the Department of General, Visceral, and Transplantation Surgery at the University of Heidelberg between 1995 and 2022 were retrospectively analyzed. Univariate Kaplan-Meier analyses and multivariate Cox regression analyses were employed to assess overall survival. Postoperative long-term quality of life was assessed using standardized questionnaires (EORTC QLQ-C30). Subgroup analyses were conducted for locally advanced tumors.

**Results:**
Out of 160 resected primary GIST, 82 (51.3%) exhibited tumor sizes ≥ 5cm (T3/4), 54 (65.9%) ≥ 8cm (T3/4), and 38 (23.8%) ≥ 10cm (T4). Neo-adjuvant TKI therapy was administered in 17 (10.6%) cases. Among locally advanced GIST, 34 (41.5%) underwent MOR, while 48 (58.5%) underwent SOR. Of those, patients undergoing MOR showed a higher amount of T4 tumors (68%) as compared to patients undergoing SOR (31%). Patients undergoing MOR with tumors ≥ 5cm showed a significantly higher rate of complications (P = 0.021) with similar severity of complications (P = 0.953) and no significant difference in overall survival (OS) (P = 0.199) compared to patients undergoing SOR. For tumors ≥ 8cm and ≥ 10cm, patients with MOR exhibited no significant differences in the number of complications (P = 0.126, P = 0.442), complication severity (P = 0.636, P = 0.506), or OS (P = 0.564, P = 0.649) compared to patients with SOR. In multivariate analyses, considering the AFIP risk score, MOR did not represent an independent prognostic factor for OS. There was no significant difference in postoperative long-term quality of life between patients undergoing MOR and SOR, regardless of tumor size.

**Conclusion:**
In locally advanced GIST with tumor sizes ≥ 8 cm, MOR does not result in increased complication rates compared to SOR and therefore appears to be safely applicable in large locally advanced GIST. Patients undergoing MOR do not differ in postoperative long-term quality of life from those undergoing SOR. MOR is not significantly associated with unfavorable OS and is no independent prognostic factor for OS, indicating an oncological benefit of exhaustive tumor resection in large locally advanced GIST that usually exhibit an unfavorable prognosis. In future studies, the oncological value of MOR should be re-evaluated in a cohort of neo-adjuvantly treated locally advanced GIST.

**Therapy and Outcomes of Retroperitoneal Soft Tissue Sarcomas Over the Last Two Decades – Introduction of Compartmental Resection Improves Disease-Specific Survival in Primary Retroperitoneal Liposarcomas.**
ID: 258

Franziska Willis[1], Julian Musa[1], Mohammed Al-Saeedi[1], Martin Schneider[1]

[1]*Department of General, Visceral and Transplantation Surgery, University hospital Heidelberg*

**Background:**
Retroperitoneal soft-tissue sarcomas (RPS) encompass a group of rare and heterogeneous tumors. A histologic tailored surgical approach for RPS is currently the subject of contentious debate, particularly regarding the utilization of compartmental resection, which involves the resection of adjacent organs even in the absence of signs of tumor infiltration. The objective of this study is to identify surgical factors associated with improved disease-specific survival following RPS resection, with a specific focus on tumor entity, tumor biology, and clinical tumor presentation.

**Materials and methods:**
A retrospective analysis was conducted on 363 patients who underwent resection for primary and recurrent RPS at the University Hospital Heidelberg between 2002 and 2019. Clinical parameters were analyzed separately for three time periods: TP1 (2002-2007), TP2 (2008-2013), and TP3 (2014-2019). Multivariate Cox regression analyses were performed to identify independent predictors of disease-specific survival. Subgroup analyses were conducted for patients with primary RPS, liposarcomas (LPS, including primary LPS), and leiomyosarcomas (LMS, including primary LMS).

**Results:**
Disease-specific survival improved over time ($p = 0.051$), with a significant effect observed particularly in the primary liposarcoma group ($p = 0.021$). Regarding clinical parameters, a significant increase in compartmental resections ($p < 0.001$) and a concomitant rise in the number of resected organs ($p = 0.001$) were the only significant changes over the three time periods, with no change in morbidity and mortality rates. The performance of compartmental resection was identified as an independent predictor of prolonged disease-specific survival in the primary liposarcoma group ($p = 0.047$). In the leiomyosarcoma subgroup, there was no improved disease-specific survival over the three time periods, and the introduction of compartmental resection did not impact disease-specific survival ($p > 0.05$).

**Conclusion:**
The enhancement of disease-specific survival is associated with the introduction of compartmental resection in the primary liposarcoma group. Therefore, a surgically tailored approach adapted to tumor histology and presentation appears to be a valuable strategy in the surgical management of RPS.

**Incidental Chemotherapy of an Adenoma (Subtitle: Therapeutic Response of a symptomatic Duodenal Adenoma to Breast Cancer Therapy)**
ID: 338

Alan Bareiß[1], Eva Grams[1], Uwe Markert[2]

[1]*Zollernalb Klinikum | Standort Balingen, Zentralradiologie, Balingen, Deutschland*, [2]*Zollernalb Klinikum gGmbH - Klinik Albstadt Klinik für Allgemein- und Viszeralchirurgie, Viszeralchirurgie, Albstadt, Deutschland*

**Background:**
Breast cancer is a common malignancy that affects millions of women worldwide. While chemotherapy is a well-established treatment for breast cancer it is not typically associated with treating benign tumors. However we have come across a case where a patient with breast cancer underwent chemotherapy and responded to the treatment by size reduction of a symptomatic duodenal adenoma. This case report sheds light on the effects of chemotherapeutic agents on benign tumors and emphasizes the importance of considering all possible outcomes when treating cancer patients. In this report we will delve into the specifics of this case. Discuss its implications, for future research and treatment possibilities.

**Materials and methods:**
A 68 year old woman was admitted to hospital for full diagnosis upon suspicion of breast cancer.
In 2021, a staging CT was performed as part of an initial baseline diagnosis of breast cancer. In addition to the expected finding of breast cancer, CT imaging revealed an unexpected intraluminal mass 3.9 cm in diameter in the duodenum, [which had likely been missed due to the focus on diagnosis of breast CA].
A retrospective chart review revealed that a gastroscopy had been performed previously in 2018 for hematemesis. During this gastroscopy, a mass in the duodenum was identified, which was biopsied as an adenoma at that time and was thought to be the cause of the hematemesis.
Neoadjuvant chemotherapy with 2 cycles of epirubicin/cyclophosphamide and 2 cycles of nab-paclitaxel was initiated for treatment of the breast carcinoma in 2021. A follow-up CT scan performed 3 months after initiation of therapy showed not only a response of the breast carcinoma but also a significant volume reduction of the duodenal adenoma. Meanwhile, the patient reported no hematemesis.
Despite these promising results, chemotherapy had to be suspended due to tolerability issues.
Fifteen months after the initial CT scan, the patient presented again radiologically due to recurrence of hematemesis. This new examination revealed a significant size progression of the duodenal adenoma, which has now almost doubled its initial size.
Open segmental resection of the duodenum with end-to-end anastomosis was performed. The histological report revealed a tubulovillous adenoma with low-grade epithelial dysplasia (low-grade IEN) with heterotopic pancreatic tissue within a widened submucosa.

**Results:**
Chemotherapeutic treatment of a benign adenoma is generally not indicated, as these tumors are typically treated with surgical resection or other less invasive methods [1].
Chemotherapeutic agents work to reduce the size of tumors by targeting various cellular processes that are essential for tumor growth and survival. Although specific information on epirubicin and paclitaxel's effects on adenomas is limited, we can discuss the general mechanisms of chemotherapeutic agents in reducing adenoma size:
1. Inhibition of DNA synthesis and function: Some chemotherapeutic agents, such as alkylating agents and antimetabolites, interfere with DNA synthesis and function, leading to cell cycle arrest and apoptosis [2]. Temozolomide would be an example of this group. It is an oral chemotherapy agent, has shown activity against pituitary adenomas [3].

2. Disruption of microtubule action: Certain chemotherapeutic agents, like paclitaxel, target microtubules and disrupt their dynamics, causing mitotic arrest and apoptosis [3]. Paclitaxel acts through this
3. Inhibition of topoisomerases: Topoisomerase- inhibitors, like epirubicin (a type of anthracycline-), disrupt the activity of topoisomerases. The-se enzymes play a vital role- in DNA replication and transcription [1]. By blocking their action, these- agents impede DNA function and can le-ad to cell death [5].
4. Induction of oxidative stress: Some chemotherapeutic agents, like anthracyclines, can generate reactive oxygen species (ROS) that cause oxidative stress and damage cellular components [5]. This oxidative stress ultimately leads to cell death and a reduction in tumor size.
In summary, chemotherapeutic agents reduce adenoma size by targeting various cellular processes. Some are aided at changing the microenvironment of a tumor. The mechanism of action of chemotherapeutic agents is not specific to whether a tumor is benign or malignant. Fast-growing tissues, however, are more susceptible to the effects mentioned above.
We were able to find only one recently published similar case, in which a colorectal adenoma responded to a immune-checkpoint-inhibition-based chemotherapy against gastric cancer [6].
In terms of limitations, we do not know if the adenoma responded to both of the chemotherapeutic agents or just one of them. We also do not have consistent duodenal contrast in the different CT scans; however, it is adequate for drawing the conclusions we arrived at.

**Conclusion:**
It is quite unusual and remarkable to come across a case where a patient, with breast cancer and a symptomatic duodenal adenoma responded positively to chemotherapy in a double fashion. The significant shrinkage of the adenoma and its cessation of hemorrhage indicate that chemotherapeutic agents do have a potentially clinically relevant impact on noncancerous tumors. This case report emphasizes the consideration of chemotherapy in challenging scenarios, where surgical removal may not be feasible or preferred. However further scientific investigation is necessary to ascertain the safety and effectiveness of using chemotherapy for treating tumors. The decision to use chemotherapy for benign lesions should be carefully evaluated, taking into account the wide range of potential side effects impacting various bodily systems such, as hematological issues, gastrointestinal disturbances, dermatologic problems, neurological complications and reproductive concerns – many of which can significantly affect the overall health condition of the patient.

1. Rocha P et a . Liver Transplantation for Treatment of Unresectable Spontaneous Ruptured Hepatocellular Adenoma: A Rare Indication. Cureus. Published online August 8, 2022. doi:10.7759/cureus.27788
2. Bukowski K et al. Mechanisms of Multidrug Resistance in Cancer Chemotherapy. IJMS. 2020;21(9):3233. doi:10.3390/ijms21093233
3. Lin AL. Is there a role for early chemotherapy in the management of pituitary adenomas? NEUONC. 2016;18(10):1350-1356. doi:10.1093/neuonc/now059
4. Weaver BA. How Taxol/paclitaxel kills cancer cells. Bement W, ed. MBoC. 2014;25(18):2677-2681. doi:10.1091/mbc.e14-04-0916
5. Morelli et al. Cardiotoxicity of Anticancer Drugs: Molecular Mechanisms and Strategies for Cardioprotection. Front Cardiovasc Med. 2022;9. doi:10.3389/fcvm.2022.847012
6. Cheema et al. Recent advances in endoscopic management of gastric neoplasms. World J Gastrointest Endosc. 2023;15(5):319-337. doi:10.4253/wjge.v15.i5.319

**Comparison of the peritoneal carcinomatosis index (PCI) assessed during open surgery to histopathological findings in patients with peritoneal metastasis from gastrointestinal and gynaecological malignancies**
ID: 345

Olivia Zoe Zwerenz[1], André Mihaljevic[1], Philipp Horvath[1 2], Can Yurttas[1]

[1]*University Hospital Tübingen, Department of General, Visceral and Transplant Surgery, Tübingen, Deutschland*, [2]*Landeskrankenhaus Feldkirch, Department of General, Visceral and Thoracic Surgery, Feldkirch, Österreich*

**Background:**
The peritoneal cancer index (PCI) is a commonly used score, calculated based on the intraoperative findings during surgical exploration of the abdominal cavity to operationalize the extent of peritoneal surface malignancies. For patients undergoing cytoreductive surgery (CRS), the surgically assessed PCI (sPCI) has proven to be a useful prognostic marker. However, it's significance is limited as it is determined by macroscopic findings only and therefore frequently overestimated due to false-positive appraisal. Thus far there are few reports about a comparison of the PCI results by histological examination found in the literature.

**Materials and methods:**
In this retrospective study we evaluated patients with indication for CRS with or without HIPEC at our center for surgical treatment of peritoneal carcinomatosis between June 2005 and October 2021. Specified criteria for inclusion in the study were gastrointestinal or gynaecological peritoneal metastasis, indication for CRS and an intraoperatively determined sPCI. The PCI assessed according to the pathology report (pPCI) was calculated and compared for each patient based on existing data by correlating the pathology reports according to the method by Jacquet et al. Statistical analysis was employed using Cohen's weighted Kappa (K) for correlation evaluation. Moreover, influence of tumor entity, sex, age and prior systemic chemotherapy were investigated.

**Results:**
In total, 704 patients were evaluated, 454 patients met the predefined inclusion criteria. Those excluded from consideration either had a sufficient lack of available data, Completeness of Cytoreduction Scores > 1, did not undergo CRS procedures, had only exploratory laparotomies performed, did not show macroscopic peritoneal carcinomatosis, or CRS performed after prior PIPAC cycles. Within this cohort, comprising 63.2% females, the median age was 54 years. The target population enclosed 19 different tumor entities. Most prevalent being colon cancer (26.9%), followed by appendiceal neoplasms with pseudomyxoma peritonei (PMP) (19.6%), ovarian malignancies (16,3%) and gastric cancer (11.5%). The median pathological PCI (pPCI = 8) was notably lower than the surgical PCI assessed during surgery (PCI = 15). Using Cohens weighted Kappa for analysis of ordinal categories to measure agreement levels, data yielded the following results: For the general study population the concordance level indicated fair agreement (K = 0.310). In 25 patients (0.055 %) patients, the pathological PCI was identical to the PCI assess during surgery. In general, the surgical PCI exceeded the pathological PCI in 399 patients (87.88 %). No remarkable difference in concordance was observed when comparing females (K = 0.305) and males (K = 0.312). Patients with PMP showed slight agreement (K = 0.184), whereas colorectal cancer (K = 0.430) showed moderate agreement, indicating the highest concordance in PCI values within the available dataset. When considering preoperative chemotherapy (K = 0.328) or chemotherapy in combination with immunotherapy (K = 0.455), an influence on the agreement was found. In comparison, among the subset of patients not receiving preoperative therapy regimens, concordance was lower (K = 0.280). This suggests a correlation between concordance and the intensity of therapy prior to CRS. The interpretation of the linear kappa values was based on the scheme established by Altman.

**Conclusion:**

Considering surgeons individual subjectivity when rating the sPCI, it becomes evident that the sPCI tends to predominantly overestimate. Given the substantial deviation between the pPCI and the sPCI, we propose that once it's been reviewed furthermore, pathological affirmation on a microscopic level may offer a more precise score. Consequently, the pPCI is expected to be of greater prognostic value by exclusion of non-malignant lesions. Yet prospective trials are needed to determine the objective. Moreover, standardized calculation guidelines in conformity with surgeons and pathologists need to be developed. Equally important is the investigation of potential underestimations linked to the pPCI. This is crucial for further argumentation.

**Role of lymph node metastases (LNM) after total neoadjuvant treatment (TNT) in locally advanced rectal cancer (LARC)**
ID: 378

Timotheus Ritter-Vanfloorop[1], Jana Nele Leoni Vanfloorop[1], Julia Kitz[2], Heiko Sülberg[3], Johanna Kreutzer[1], Andreas Leha[4], Julie Schanz[5], Torsten Liersch[1]

[1]*Klinik für Allgemein-, Viszeral- und Kinderchirurgie, Universitätsmedizin Göttingen, Göttingen, Deutschland*, [2]*Institut für Pathologie, Universitätsmedizin Göttingen, Göttingen, Deutschland*, [3]*X-act Cologne Clinical Research GmbH, Köln, Deutschland*, [4]*Institut für Medizinische Statistik, Universitätsmedizin Göttingen, Göttingen, Deutschland*, [5]*Klinik für Hämatologie und Medizinische Onkologie, Universitätsmedizin Göttingen, Göttingen, Deutschland*

**Background:**
Locoregional LNM represent one of the most prognosis-limiting factors for survival after standard multimodality treatment of patients with locally advanced rectal cancer (LARC; stage ≥ II). But what is their relevance still after total neoadjuvant treatment (TNT)?

**Materials and methods:**
62 patients (15 f, 47 m; median age: 62 years) with LARC of the lower or mid rectum (< 12 cm from anal verge) were included in these monocentric analyses. TNT was performed according to the TransValid-B-phase-I/II-protocol (WHO-UTN-U1111-1132-0235). Staging revealed clinical stages II to IV in 1.8%, 90.3% and 8.1%, respectively. A positive nodal status was diagnosed in 21.0% (N1) and 77.4% (N2) at staging. CRT was performed with 28 fractions of irradiation (1.8 Gy/d; total dose 50.4 Gy) and concomitant CTx with 5-FU (250 mg/m$^2$/d, civ, d1-d14 and d22-d35) and OX (50 mg/m$^2$ on d1, d8, d22 and d29). Four weeks later, FOLFOX-CTx was applied with OX (80 to 100 mg/m$^2$), folinic acid (400 mg/m$^2$) and 5-FU (2400 mg/m$^2$ civ over 46h) on d1, d15 and d30. TME was performed five weeks later, followed by extended histopathologic work-up of the specimen using the acetone-compression method for LN assessment (7th TNM/UICC-classification). Survival was calculated using Kaplan-Meier or Aalen-Johansen estimators, Logrank- or Gray-tests and multiparametric Cox proportional-hazards regression models.

**Results:**
58 patients (93.5%) underwent TME-surgery with R0, negative CRM and optimal TME quality in 94.8%, 91.4% and 89.7%, respectively. Postoperatively, stages 0 to IV and pCR (ypT0 N0) were diagnosed in 15.5%, 19%, 31%, 24.1%, 10.3% and 17.2%, respectively. In a total of 1176 LN examined, 60 LNM were detected (LN-ratio = 0.051). On average, 20 ± 9.3 LN were analyzed per specimen. ypN0 status was diagnosed in 69%, but 1-3 LNM and > 4 LNM were detected in 20.3% and 10.3% of specimen, respectively. During 63 months (median) of follow-up (mean value 71 ± 33 mo) no local relapse occurred. The cumulative incidence of distant metastases (DM) at 5 years was 24.2%. Patients with residual LNM after TNT had a higher and earlier risk for DM vs. patients without LNM (5-year-DM-risk: 45% vs. 15%; p = 0.013). Patients with ypN0 status had a significant better CSS vs. patients with LNM (5-year-risk for cancer-related death: 5% vs. 34%; p = 0.01). Further results will be presented at the DCK 2024 in detail.

**Conclusion:**
TNT resulted in a promising rate of pCR and long-term local control in patients with LARC. Even after TNT, a large yield of LN could be assessed for improved differentiation between ypN0 vs. ypN+ status. Patients with ypN0 status after TNT had a significantly lower risk of DM and better CSS.

Abstracts – DCK 2024 – 141st Congress of the German Society of Surgery – Leipzig, April 24–26 • DOI 10.1515/iss-2024-2003
Innov Surg Sci 2024; 9 (Special Suppl 1): s18–s203

### The Prognostic Significance of Pyruvate Dehydrogenase Kinase (PDK) 1-4 Expression in Patients with Resectable Gastric Cancer

ID: 410

Noelle-Marie Paeger[1], Thomas Schaschinger[1], Marc Bleul[1], Sophia Holten[1], Sofia Quitter[1], Christopher Tuffs[1 2], Henrik Nienhüser[1], Nathalia Giese[1], Martin Schneider[2], Moritz Strowitzki[1 2]

[1]*Heidelberg University, Department of General, Visceral and Transplantation Surgery, Heidelberg, Deutschland*, [2]*University Hospital Gießen, Department of General, Visceral, Thoracic, Transplantation & Paediatric Surgery, Gießen, Deutschland*

**Background:**

Gastric cancer (GC) is one of the deadliest cancers worldwide. While multimodal therapies improve survival in early, non-metastatic stages prognostic biomarkers to predict tumor growth and outcome of patients undergoing surgery are lacking. Tumor cells undergo a metabolic switch from oxidative phosphorylation to anaerobic glycolysis, which promotes cell survival and proliferation. Metabolic reprogramming in tumor cells is partly meditated by an upregulation of pyruvate dehydrogenase kinase (PDK) isoenzymes 1-4 which has been shown to promote tumorigenesis in, for example, metastasized colorectal cancer. Here we investigated the significance of PDK1-4 as prognostic markers for resectable GC.

**Materials and methods:**

Specimen of healthy and tumor tissue of patients undergoing multimodal treatment (surgery and perioperative chemotherapy) of resectable gastric cancer were collected. In total, 129 patient samples were analyzed by immunolabeling to determine the protein expression of PDK1-4 in healthy and tumor tissue. The expression of PDK1-4 was semi-quantitatively assessed using the tumor H-score implemented in QuPath. Clinical data and patient outcome were collected in a prospective database and retrospectively analyzed. Expression levels of PDK1-4 were correlated with overall survival and disease-free survival using the Kaplan-Meier model and the Log rank test in R.

**Results:**

Protein expression levels of PDK1-4 were determined and patients showed overall heterogenous expression level with, for example, high expression of PDK4 within adjacent healthy crypt cells and tumor cells. The patient cohort was thus further stratified in patients with high (>median) and low (<median) protein expression of PDK1-4 within tumor tissue. Interestingly, preliminary results show that a lower protein expression of PDK1 and PDK4 within the tumor tissue was associated with a shorter overall survival in patients with GC. In contrast, protein expression of PDK2 and PDK3 was not associated with altered patient survival.

**Conclusion:**

Contrasting our initial hypothesis that a high PDK1-4 expression within the tumor is associated with a shorter overall survival in cancer patients, our data suggest that a low expression of PDK1 and PDK4 (not PDK2 or PDK3) is associated with a shorter overall survival in patients with resectable GC. Multivariate analyses will determine whether PDK1 and PDK4 expression is an independent prognostic factor in resectable GC next to known risk factors, such as age, perioperative chemotherapy, and pathological scoring classification.

**Inferior Vena Cava Resections Without Reconstructions: A Case Report**
ID: 436

Iris-Iuliana Adam[1 2], Bogdan Moldovan[2], Liliana Vecerzan[2]

[1]*Facultatea de Medicină,ULBS, Sibiu, Romania*, [2]*Spitalul Sf. Constantin, Brașov, Romania*

**Background:**
Retroperitoneal tumors are considered to be a group of heterogenous formations, which include among others renal carcinomas, retroperitoneal liposarcomas, leiomyosarcomas as well as metastatic lymph nodes. We aim to present our experience with the resection of the inferior vena cava (IVC) without reconstruction in a patient diagnosed with a low-grade endometrial adenocarcinoma.

**Materials and methods:**
A 54-year-old patient with the historyof an radio- and chimiotreated low-grade endometrial adenocarcinoma, presented to the general surgery service of the St. Constantine Hospital Brașov, with a thrombosis of the inferior vena cava and a giant retroperitoneal tumorous formation. The invasion of both iliac veins was also identified, altering the normal blood flow.

**Results:**
The excision of the giant tumor was performed using a complex approach, in which the dissection of the giant retroperitoneal tumor was performed, together with the resection of the thrombosed IVC, without the reconstruction of it. The next operative time consisted of the resection of the inferior part of the duodenum D2, D3 and successive resections of the liver in the third and sixth segment. Subsequently, due to its invasion and lack of normal functioning a nephrectomy was performed. Subsequent histopathological examination revealed that the histological appearance and immunohistochemical profile correspond to the previous histopathological results (2022) and confirmed an low-grade endometrial adenocarcinoma, G3, along with a retroperitoneal recurrence andd multtiple metastases located in the liver.

**Conclusion:**
The involvement of the inferior vena cava in the context of an oncological pathology is considered to be a criteria of inoperability in most of the cases, even more so when it's due to a thrombotic vascular obstruction. The 'St. Constantin 'Hospital of Brasov surgical team highlighted the surgical algorithm to be used in these cases in 2023, proving the fact that they can be applied even in patients with a thrombus at IVC levels, which have a well preserved functional blood flow. Safe R0 surgical resections of the tumor can be performed with the help of a thrombectomy through a cavotomy followed by cavoraphy. This will further ensure a long term disease free survival rate.

### The Therapeutic Approach Of Peritoneal Carcinomatosis In Poorly Cohesive Gastric Cancers: Quo Vadis?

ID: 439

Iris-Iuliana Adam[1 2], Bogdan Moldovan[2], Liliana Vecerzan[1]

[1]*Facultatea de Medicină,ULBS, Sibiu, Romania*, [2]*Spitalul Sf. Constantin, Brașov, Romania*

**Background:**

Considered as a multifactorial disease, the gastric cancer, can be determined by environmental factors as well as genetic ones, occupying the fourth place in the ranking of mortality, whilst the diagnosis of gastric cancer being most often diagnosed in advanced stages, limiting the chances of an optimal therapeutical approach. About 40% of patients present at the time of diagnosis secondary determinations with multiple localisations, peritoneal carcinomatosis being one of the main sites of metastasis. Its complication most often leads to intestinal occlusion, the appearance of a significant ascitis quantities, leading limitations in the therapeutic conduct, often resulting in a poor prognosis. The favorable response rate to systemic chemotherapy is low in patients with peritoneal carcinomatosis, especially in those ones, which originated fromthe gastrointestinal tube. Neoadjuvant chemotherapy is the standard of treatment in these cases, thus not always obtaining an operability status afterwards for most of the patients.Cytoreductive surgery (CRS) followed by hyperthermic intraperitoneal chemotherapy (HIPEC) is considered to be the only potentially curative treatment, showing a better prognosis in cases where the peritoneal cancer index (PCI) lower scores, helping in achieving radical macroscopic cytoreduction.

**Materials and methods:**

A 63-year-old patient with no significant personal pathological medical history presents to the general surgery unit of the St.Constantin HospitalBrașov, with the diagnosis of poorly cohesive gastric carcinoma with bilateral ovarian metastases and limited peritoneal carcinomatosis, as well as an form of atypical right pleurisy.

**Results:**

Cytoreductive surgery consisted in a total splenogastrectomy, total histerectomy with bilateral anexectomy, perigastric lymphadenectomy and selective peritonectomy, along with an abdominal 4-quadrant and double right pleural drainage, followed by hyperthermic intraperitoneal chemotherapy procedure which consisted of: Cisplatin 150 mg/m2 and Mitomycin C 30 mg/m2. Given the massive right pleurisy as well as the cytology showing signs of malignancy, HITOC lavage without pleurectomy is decided and performed. Histopathological and immunohistochemical examinations revealed the presence of a poorly cohesive gastric carcinoma (G3) with the presence of lymphonodal, ovarian, appendicular and peritoneal metastases.

**Conclusion:**

The surgical approach in patients presenting peritoneal metastases coming from gastric carcinomas is very complex and difficult. The HIPEC/HITOC procedures require extensive surgical exploration and may lead in most cases in the removal of all the affected organs. The most common way of HIPEC administration in these cases of peritoneal metastases consists of an intra-abdominal lavage done after the gastrointestinal anastomosis at 42–43°C for 60–90 minutes through the drainage tubes placed inabdomen with Cisplatin 150 mg/m2 and Mitomycin C 30 mg /m2 previously dissolved in 4 L of saline solution at a flow rate of 500 ml/min in a closed HIPEC system. Complete cytoreduction followed by systemic chemotherapy remains the main treatment modality and should be considered as a first intention in patients with secondary determinations given by poorly cohesive gastric cancers.

**The Effect Of Adjuvant Chemotherapy In Patients With Resected Distal Cholangiocarcinoma -– A Population-Based Analysis Of The German Cancer Registry Group Of The Society Of German Tumor Centers**
ID: 445

Jannis Duhn[1], Lennart von Fritsch[1], Kim Honselmann Genannt Humme[1], Rüdiger Braun[1], Markus Kist[1], Thaer Abdalla[1], Sylke Zeißig[2 3], Kees Kleihues-van Tol[2], Tobias Keck[1], Monika Klinkhammer-Schalke[2], Richard Hummel[4], Ulrich Wellner[1], Louisa Bolm[1]

[1]*Universitätsklinikum Schleswig-Holstein, Campus Lübeck, Klinik für Chirurgie, Lübeck, Deutschland*, [2]*Arbeitsgemeinschaft Deutscher Tumorzentren e.V. , Berlin, Deutschland*, [3]*University of Würzburg, Institute of Clinical Epidemiology and Biometry (ICE-B), Würzburg, Deutschland*, [4]*Universitätsmedizin Greifswald, Klinik und Poliklinik für Allgemeine Chirurgie, Greifswald, Deutschland*

**Background:**
Distal Cholangiocarcinomas (dCCA) are rare malignant neoplasms of the extrahepatic common bile duct, with a 5-year overall survival (OS) of below 50% even after surgical resection. The benefit of adjuvant chemotherapy (AC) following oncological resection remains controversial.

**Materials and methods:**
Data was obtained from 16 clinical cancer registries participating in the German Cancer Registry Group of the Society of German Tumor Centers (ADT). Patients being diagnosed with dCCA (ICD-10: C24.1) between 2000 and 2021 who underwent upfront surgery were selected. For independent variables with less than 20% missing data, a single imputation was performed. Negative prognostic factors were identified using uni- and multivariate cox-regression analyses. Following up, the effect of AC on the OS was assessed in subgroup analyses of patients harboring negative prognostic factors. Statistical analyses were performed with R statistics.

**Results:**
We identified 1430 patient that received primary resection following diagnosis with dCCA. Of these, 206 patients (14.4 %) received AC. Patients treated with AC had significantly larger tumors (pT 3-4: 74.3 % vs. 42.9 %, $p < 0.001$) and more lymph node positive disease (pN+: 69.7 % vs. 42.8 %, $p < 0.001$). In univariate analysis, AC did not improve OS compared to surgery only (median OS: 31.9 ± 5.5 vs. 24.8 ± 3.3 months, $p = 0.367$). However, in a multivariable cox regression analysis including sex, age, tumor size, lymph node metastases, resection margins, as well as lymph- and hemangiosis, AC was proven as independent positive prognostic factor for the OS (HR 0.7 (0.54 – 0.91, $p = 0.007$). A subgroup analysis was performed in order to identify patients profiting from AC. Thereby, adjuvant chemotherapy significantly improved the OS of patients with pT3-4 staged tumors (HR 0.76 (0.60 – 0.98), $p = 0.029$), lymph node positive disease (HR 0.72 (0.55 – 0.93), $p = 0.01$) and undifferentiated tumor grading (G3-4) (HR 0.71 (0.50 – 0.99), $p = 0.038$). In other subgroups, AC did not prolong long-term outcomes.

**Conclusion:**
As far to our knowledge, this is the first population-based analysis evaluating the effects of AC on the OS of patients with dCCA. Hereby, we show that a more distinguished selection of patients suitable for AC is necessary, as only subgroups harboring negative prognostic factors, such as pT3-4 tumors, lymph node metastasis and high tumor grading profit from AC.

**Oncologic Outcomes After Surgical Resection of Pancreatic Mucinous Adenocarcinoma – A Retrospective Real-World Data Analysis From The German Cancer Registry Group Of The Society Of German Tumor Centers**
ID: 449

Jannis Duhn[1], Christoph Gerling[1], Lennart von Fritsch[1], Kim Honselmann Genannt Humme[1], Louisa Bolm[1], Thaer Abdalla[1], Markus Kist[1], Sylke Zeißig[2 3], Kees Kleihues-van Tol[2], Tobias Keck[1], Monika Klinkhammer-Schalke[2], Richard Hummel[4], Ulrich Wellner[1], Rüdiger Braun[1]

[1]*Universitätsklinikum Schleswig-Holstein, Campus Lübeck, Klinik für Chirurgie, Lübeck, Deutschland*, [2]*Arbeitsgemeinschaft Deutscher Tumorzentren e.V., Berlin, Deutschland*, [3]*University of Würzburg, Institute of Clinical Epidemiology and Biometry (ICE-B), Würzburg, Deutschland*, [4]*Universitätsmedizin Greifswald, Klinik und Poliklinik für Allgemeine Chirurgie, Greifswald, Deutschland*

**Background:**
Mucinous adenocarcinoma (MACP) is a rare histological subtype of pancreatic cancer, defined by mucous content within the tumor of more than 50%. Whether MACP arise from premalignant cystic lesions or display a subtype of pancreatic ductal adenocarcinoma (PDAC) is yet incompletely understood. Recently, a retrospective analysis from the National Cancer Database showed that MACP tumors are diagnosed more often as stage I disease compared to PDAC and are associated with better overall survival (OS) in stage matched cohorts. Our analysis aims to compare the oncological outcomes and survival rates of MACP patients compared to PDAC patients in real-world data from the German Cancer Registry Group of the Society of German Tumor Centers.

**Materials and methods:**
Data were retrieved from 16 clinical cancer registries participating in the German Cancer Registry Group of the Society of German Tumor Centers (ADT). Patients diagnosed with Pancreatic Cancer (ICD-10: 25.0 – 25.9) between 2000 and 2021, that underwent surgical resection, were selected. In addition, histological subtypes were differentiated by ICD-O3 Histology Code (MACP: 8480/3; PDAC: 8500/3). Histopathological features as well as tumor stages were compared between MACP and PDAC using Chi-squared tests. We performed a multivariable analysis using a cox-proportional hazards model to assess the prognostic impact of a MACP histology. Finally, prognostic factors in MACP were evaluated by uni- and multivariable analyses using R.

**Results:**
We included 345 patients with MACP and 8959 patients with PDAC, that received surgical resection between 2000 and 2001. MACP patients had significantly more often pT1 stage disease (8.1 % vs. 3.2 %, $p < 0.001$) and less lymph node metastases (pN0: 43.7 % vs. 30.5 %, $p < 0.001$). In addition, MACP tumors had lower grading, and less lymphatic and blood vessel invasion ($p < 0.001$). However, the R0 resection rate was comparable between both groups. In total, MACP was associated with a better OS compared to PDAC (median OS: 21.7 ± 3.2 vs. 15.7 ± 0.2 months, $p < 0.001$). In a multivariable analysis including age, sex, tumor stage, lymph node metastases, grading, and usage of adjuvant chemotherapy, MACP histology was an independent positive prognostic factor (HR 0.82 ± 0.13, $p = 0.02$). In univariable analyses of MACP patients, tumor stage, lymph node metastasis, grading, lymph- and blood vessel invasion as well as resection margins were identified as significant prognostic factors. Of these, lymph node metastasis, grading and resection margins were proven as independent prognostic factor in a multivariable analysis. Importantly, adjuvant chemotherapy significantly improved OS of MACP patients (median OS: 25.0 ± 2.5 vs. 17.1 ± 4.7 months, $p = 0.045$), which was confirmed in a multivariable analysis (HR 0.67 ± 0.2, $p = 0.02$).

**Conclusion:**
We confirm the superior oncological outcome of MACP compared to PDAC following surgical resection in a large, cancer-registry based German real-world data analysis. Besides presenting with lower tumor stages, MACP histology was proven as independent positive prognostic factor. As previously observed in PDAC, patients with MACP benefit from adjuvant chemotherapy independent of the tumor stage. This study highlights the relevance of differentiating histologic subtypes in pancreatic cancer for patient prognosis.

Abstracts – DCK 2024 – 141st Congress of the German Society of Surgery – Leipzig, April 24–26 • DOI 10.1515/iss-2024-2003
Innov Surg Sci 2024; 9 (Special Suppl 1): s18–s203

**Lymphadenectomy is indicated in non-functional pancreatic neuroendocrine neoplasms larger than 2 cm: A population-based analysis of the German Clinical Cancer Registry Group.**
ID: 482

Stanislav Litkevych[1], Thaer Abdalla[1], Louisa Bolm[1], Kim C. Honselmann[1], Rüdiger Braun[1], Hryhoriy Lapshyn[1], Monika Klinkhammer-Schalke[2], Sylke Zeißig[3], Kees Kleihues-van Tol[2], Tobias Keck[1], Richard Hummel[4], Ulrich Wellner[1], Steffen Deichmann[1]

[1]*University Medical Center Schleswig Holstein- Campus Lübeck, Lübeck, Deutschland*, [2]*German Cancer Registry Group of the Society of German Tumor Centers - Network for Care, Quality and Research in Oncology (ADT), Berlin, Deutschland*, [3]*University of Würzburg, Institute of Clinical Epidemiology and Biometry (ICE-B), Würzburg, Deutschland*, [4]*University Medical Center Greiswald, Department of Surgery, Greifswald, Deutschland*

**Background:**
Patient selection for lymphadenectomy remains a controversial aspect in the treatment of pancreatic neuroendocrine neoplasms (pNEN), given the growing importance of parenchyma-sparing resections and minimal invasive procedures.

**Materials and methods:**
This population-based analysis was derived from German Cancer Registry Group (GCRG-ADT) during the period from 2000 to 2021. Patients with upfront resected non-functional non-metastatic pNEN were included.

**Results:**
L Out of 5520 patients with pNEN, 1006 patients met the inclusion criteria. (53%) of the patients were male. The median age was 64 ± 17 years. G1, G2 and G3 pNEN were found in 57%, 37% and 7% of the patients, respectively. Lymph node metastasis (N+) was present in 253 (24%) of all patients. N+ was an independent prognostic factor (HR 1.79, CI 95% 1.21-2.64, p =0.001) for disease-free survival (DFS). The 3-,5- and 10- disease survival in nodal negative tumors compared to nodal positive was 82% vs. 53, 75% vs. 38, and 48% vs. 16%. N+ was present in 5 % of T1 tumors, 25% of T2 tumors, and 49 % of T3-T4 tumors. In T1 tumors, G1 was the most predominant tumor grade ( 80%). However, in T2 tumors, G2 and G3 represented 44% and 5% of all tumors. N+ was associated with tumors located in the pancreatic head (p < 0.001), positive resection margin (p < 0.001), tumors larger than 2 cm (p < 0.001) and lower tumor differentiation (G2 vs G1)/(G3 vs G1) (p < 0.001, respectively). The multivariable analysis showed that tumor size, tumor grade and location were independent prognostic factors associated with N+ that could identified preoperatively, which can be used to predict N+.

**Conclusion:**
N+ is an independent negative prognostic factor for DFS. Due to the low incidence of N+ in T1 tumors (5%), parenchyma-sparing surgery is oncologically adequate in small G1 and pNETs. However, in T2- or G2 / G3 tumors regional lymphadenectomy should be recommended.

**Prognosis in perihilar cholangiocarcinoma comparing right- vs left-sided**
ID: 535

Svenja Sliwinski[1], Hanan El Youzouri[1], Jan Heil[1], Michael Heise[1], Faruk Koca[1], Wolf Bechstein[1], Ekaterina Petrova[1], Andreas Schnitzbauer[1]

[1]*University Hospital Frankfurt, Goethe University Frankfurt/Main , Department for General, Visceral, Transplant and Thoracic Surgery, Frankfurt/Main, Germany, Frankfurt am Main, Deutschland*

**Background:**
The extent of liver resection in perihilar cholangiocarcinoma (pCC) is still a debated topic. We investigated the correlation between surgical approaches comparing left vs. right resections in pCC hypothesising that the pure trisectorectomy right approach is not superior to individual decision making in pCC resection strategies.

**Materials and methods:**
A retrospective evaluation of all patients receiving liver resection for pCC over a 17-year period was performed. Correlations with outcomes such as mortality and postoperative complications according to the Clavien-Dindo (CD) classification were performed. T-test, $\chi^2$, univariate and multivariate analyses as well as survival studies via Kaplan-Meier-curves were undertaken using SPSS.

**Results:**
A total of 123 patients were included (31.7% female and 68.3% male). The median age was 67 (28-87) years. Concomitant underlying liver diseases ranged from choledocholithiasis (8.1%), PSC (4.1%), fibrosis/cirrhosis (5.4%), NASH/ASH (8.1%) to viral hepatitis (8.9%). Most frequent symptoms were jaundice (73.2%), weight loss (32.5%), pain (16.3%) and inappetence (10.6%). 16.7% of the patients received neoadjuvant treatment. A right hepatectomy was performed in 17.1% (16.3% extended right, 14.6% trisectorectomy), a left hepatectomy in 17.9% of the cases (17.9% extended left, 5.7% left trisectorectomy). 10.6% with a Bismuth grade I tumor received a bile duct resection only. Major complication rates CD≥IIIb were 41.5%. 90-day mortality rate was 22%. Median overall survival was 20 months (CI 13.3.-26.7). Differences in complication rates or the overall survival between the groups left or right resections were not seen. Postoperative liver failures (p=<0.001) and major complications (p=0.025) were more frequent in right resections. Accordingly, 90-day mortality was higher (p=0.011). Biliary leakage (p=0.003) occurred more often in left resections. Subgroups left vs. right hepatectomies only differed in the number of postoperative lifer failures (p=0.008) favoring left hepatectomies. When comparing right trisectorectomies as a subgroup only rates of postoperative biliary leakage were higher in this group (p=0.002). Univariate predictive markers for postoperative mortality were age >65 years, postoperative liver failure, hemorrhage, major complications and recurrence in right resections, and major complications and recurrence in left resections.

**Conclusion:**
Resection strategies should be made on an individual level for each patient and do not necessarily require a right trisectorectomy in pCC surgical approaches. As perioperative mortality up to 90 days is still high in pCC there should be a major focus on prevention of post hepatectomy liver failure as one of the driving risk factors for mortality.

### Complete tumor resection and Hyperthermic Intraperitoneal Chemotherapy (HIPEC) for prevention and treatment of peritoneal metastases in patients with colorectal cancer - a meta-analysis

ID: 561

Franziska Willis[1], Martin Schneider[1], Tim O. Vilz[2], Maria A. Willis[2]

[1]*Klink für Allgemein-, Viszeral-, Thorax- und Transplantationschirurgie , Universitätsklinikum Gießen und Marburg, Gießen, Deutschland*, [2]*Klinik für Allgemein-, Thorax-, Viszeral- und Gefäßchirurgie, Universitätsklinikum Bonn, Bonn, Deutschland*

**Background:**
Peritoneal metastases are frequent in colorectal cancer (CRC) and are associated with a poor prognosis. Complete cytoreductive surgery is associated with improved survival in patients with peritoneal carcinomatosis (PC). However, the contribution of supplementary Hyperthermic Intraperitoneal Chemotherapy (HIPEC) in both the prophylaxis and therapeutic management of PC remains to be definitively elucidated. The objective of this study was to assess the evidence for the use of HIPEC in combination with complete tumor resection in CRC patients for prevention and treatment of peritoneal metastases.

**Materials and methods:**
We systematically searched Medline, CENTRAL and Embase databases for randomized controlled trials comparing surgery In combination with HIPEC compared to surgery alone in CRC. Outcomes of interest were overall survival (OS) and peritoneal metastases-free survival (PCFS) as well as HIPEC associated side effects. We excluded studies in which surgery and HIPEC were compared to palliative therapy approaches without complete tumor resection. Pooled results were reported as mean difference (MD) or risk ratio (RR) and 95% confidence intervals (CI) using the Mantel-Haenszel method. The certainty of evidence was assessed with GRADE.

**Results:**
Four RCTs including 801 participants were identified, three on prophylactic HIPEC to prevent peritoneal metastases and one RCT on therapeutic HIPEC to treat peritoneal CRC metastases. For both used chemotherapeutics and timing of application the studies used different protocols. Possible HIPEC associated side effects were not reported in all studies. Anemia was only reported in one study, leukopenia and thrombocytopenia were reported in two studies. Preliminary analyses of current randomized evidence do not support any benefit for OS or PCFS. However, only one study investigated HIPEC with Mitomycin C and found improved PCFS in the HIPEC group (RR 0.21, 95% CI 0.05-0.95) with no difference in OS (RR 0.53, 95% CI 0.05-5.78). Neutropenia and Thrombocytopenia were diagnosed more frequently in the HIPEC group (neutropenia: RR 2.45, 95% CI 1.29-4.66; thrombocytopenia: RR 7.47, 95% CI 1.70-32.85).

**Conclusion:**
The current evidence from RCTs does not support the application of HIPEC in treatment or prevention of PC in CRC. However, the use of Mitomycin C instead of platin-based HIPEC regimens seems promising and should be further investigated in both the prevention and treatment peritoneal metastases. But until further evidence from multicenter studies is available the use of HIPEC in CRC should be considered experimental.

**Impact of Nintedanib on tumor angiogenesis and vascular normalization in a mouse model of colorectal cancer**
ID: 587

Naita Wirsik[1], Jens Seeger[2], Thomas Schmidt[1], Frank Hilberg[3], Hans Fuchs[1], Christiane J. Bruns[1], Oliver Coutelle[2], Hamid Kashkar[2 4], Lars Schiffmann[1 2 4]

[1]*Department of General, Visceral, Cancer and Transplant Surgery, , University of Cologne, Köln, Deutschland*, [2]*Institute for Molecular Immunology, Faculty of Medicine and University Hospital of Cologne, Cologne*, [3]*Boehringer Ingelheim RCV, Vienna*, [4]*Center for Integrated Oncology (CIO) Cologne Bonn, Cologne, Deutschland*

**Background:**
Anti-angiogenic treatment with the VEGF antibody bevacizumab is standard of care for many cancer entities including colorectal cancer. However, long-term anti-angiogenic treatment alone or in combination with other therapies can lead to resistance through the stimulation of alternative pro-angiogenic pathways. In this study, we investigated the effects of nintedanib, an anti-VEGFR/PDGFR/FGFR kinase inhibitor, on tumor vasculature to determine the potential benefits of combined inhibition of multiple pro-angiogenic factors.

**Materials and methods:**
Subcutaneous xenograft tumors were treated with the VEGFR/PDGFR/FGFR inhibitor nintedanib. Changes in tumor growth were measured and parameters of structural and functional tumor angiogenesis and vascular normalization were analysed by immunohistochemistry.

**Results:**
Inhibition of VEGFR/PDGFR/FGFR by Nintedanib inhibited tumor growth in a dose dependent manner and was accompanied by a reduction in vessel density leading to tumor cell death. The remaining vessels showed decreased vascular leakage and improved oxygen delivery, indicating a functionally and structurally improved vascular bed resulting from vascular normalization.

**Conclusion:**
Nintedanib showed anti-tumoral effects and led to vascular normalization in a xenograft mouse model of colorectal cancer. Our findings suggest for the first time that Nintedanib is able to foster drug and oxygen delivery to solid tumors thereby improving efficacy of conventional oncological therapies such as chemotherapy and radiation.

**HPV genotype landscape in primary, recurrent and metastatic anal squamous cell carcinoma – a retrospective single center analysis**
ID: 596

Rose Seiberth[1], Daniel Gödde[2], Hans Michael Kvasnicka[2], Florian Gebauer[1], Lars Bönicke[1]

[1]*Helios Universitätsklinikum Wuppertal Universität Witten/Herdecke, Klinik für Allegmein-, Viszeral- und Onkologische Chirurgie, Wuppertal, Deutschland*, [2]*Helios Universitätsklinikum Wuppertal Universität Witten/Herdecke, Institut für Pathologie und Molekularpathologie, Wuppertal, Deutschland*

**Background:**
The occurrence of anal squamous cell carcinoma (SCCA) is predominantly preceded by persistent HPV infections. HPV 16 positivity in SCCA has been identified as a positive prognostic factor associated with improved response to CRT. However, more than 14 high-risk genotypes have been linked to cancer development. Until now, HPV genotyping was primarily carried out on pre-treatment local biopsies alone. To identify tumor-specific patterns of HPV infections throughout all stages of SCCA, we conducted a retrospective analysis of primary, recurrent and metastatic tumors for HPV genotyping.

**Materials and methods:**
In a retrospective analysis, patients treated for SCCA in our high volume institution between 2009 and 2019 were identified using our clinical (n = 179 patients) and pathology databases (n = 620 tumor specimens). Initial treatment consisted of either local resection or chemoradiation therapy according to the tumor stage. A total of n = 242 tumor specimens (primary, recurrent and metastases) of n = 142 patients were finally tested for HPV using a single reaction Multiplex real-time PCR with high sensitivity and specificity by utilization of DPO™ and TOCE™ technologies**.** Here, tumor samples with inadequate DNA recovery were excluded. Accurate genotyping of 28 HPV types (19 high-risk genotypes, 9 low-risk HPV genotypes) was performed on n = 213 specimens of n **=** 136 patients (female n = 82 (60.3%), male n= 54 (39.7%))**.**

**Results:**
In total, intratumoral HPV presence was detected in 179 of all samples (84.0%), while HPV was absent in 34 specimens (16.0%). Of those 179 HPV positive samples, HPV was detected in 125 primary untreated tumors (69.8%), in 39 local relapse samples (21.8%), in 13 metastases (7.2%) and in 2 re-relapse samples (1.1%). Throughout all 179 HPV positive tumor samples, HPV genotyping detected a total of n = 24 different subtypes, consisting of n = 15 high-risk subtypes (62.5%) HPV 16, 18, 31, 33, 35, 39, 51, 52, 53, 56, 58, 66, 68, 73 and n = 9 low-risk subtypes (37.5%) HPV 6, 11, 40, 42, 43, 44, 54, 61, 70.
HPV positivity was present in a total of n = 269 times, ranging from single subtype presence to eightfold subtype combinations, with high-risk HPV subtypes present in n = 219 of all combinations (81.4%) and low-risk HPV subtypes present in n = 50 combinations (18.6%). The most common high-risk HPV subtypes were HPV 16 in 60.2% (n = 162), HPV 33 in 6,1% (n = 16) and HPV 31 in 2,60% (n = 7) of all combinations. Persisting HPV positivity from pre-treatment samples to metastases occurred in 61.5% (n = 8) of all HPV positive metastases (n = 13).
Loss of HPV positivity from pre-treatment samples to metastases occurred in 40% (n = 2) of all HPV negative metastases (n = 5).
Gain of HPV positivity from HPV negative pre-treatment tumor to HPV positive metastatic disease was shown in one patient (7.7%) of all HPV positive metastases.
Similarly, gain of HPV positivity from HPV 16 positive pre-treatment tumors to HPV 31/33 positive metastases occurred in 2 patients (15.4%) of all HPV positive metastases.
While all HPV positive metastases carried high-risk HPV genotypes exclusively, 2.4% of all HPV positive pre-treatment tumors harbored only low-risk HPV genotypes.

**Conclusion:**

We are the first to describe a standardized analysis of accurate HPV genotyping for a SCCA tumor collective of this magnitude, including primary, recurrent and metastatic tumors.
Even with current HPV vaccination for subtypes 16 and 18 available, in our collective 18.6% of all HPV positive tumors harbored non-HPV16/18-high-risk genotypes.
HPV genotype transformation from initial diagnosis to metastatic disease, developing either HPV gain or HPV loss, may carry implications for future targeted SCCA therapy.

**Artificial Intelligence and Clinical Decision-Making: A Study of ChatGPT in Gastrointestinal Cancer Treatment**
ID: 643

Ughur Aghamaliyev[1], Matthias Ilmer[1], Clemens Gießen-Jung[2], Bernhard W Renz[1], Jens Werner[1]

[1]*Klinikum der Universität München, Klinik für Allgemein-, Viszeral- und Transplantationschirurgie, München, Deutschland*, [2]*Klinikum der Universität München, Medizinische Klinik und Poliklinik 3, München, Deutschland*

**Background:**
Artificial intelligence (AI) has shown great promise in improving clinical decision-making, especially in complex cases such as cancer treatment recommendations [1]. One such AI model is ChatGPT, a natural language processing (NLP) model that has been shown to accurately generate responses to clinical questions [2] [3]. This study aimed to compare the decision-making ability of ChatGPT with that of a multidisciplinary tumor board (MDT) in providing gastrointestinal cancer treatment recommendations.

**Materials and methods:**
The retrospective study included 101 patients with gastrointestinal malignancies who received treatment recommendations from the MDTB. The patients' information, including diagnosis, age, sex, and ECOG-status, was inputted manually into ChatGPT by physicians. The recommendations generated by ChatGPT were compared with those of the MDTB. Responses by ChatGPT were regenerated three times. The results were considered concordant when the treatment strategy recommended by ChatGPT matched that of the MDTB.

**Results:**
The study found that ChatGPT was able to accurately advise on whether neoadjuvant, adjuvant, or primary surgery was the best course of action in 86% of cases. Furthermore, 92% of the recommendations were reproducible. However, the study also found that ChatGPT's provided precise and reproducible recommendations for chemotherapy regimens or types of radiological interventions in 70% of cases, with non-reproducible recommendations identified in almost 20% of cases.

**Conclusion:**
Our study demonstrated ChatGPT's high accuracy in advising on neoadjuvant, adjuvant, or primary surgery, with reproducible recommendations in the majority of cases. However, its limitations in recommending specific chemotherapy regimens or radiologic intervention types suggest that further research is necessary to better understand its implications and limitations in clinical settings. Nonetheless, ChatGPT's accuracy in conceptual questions indicates its potential usefulness in the treatment of tumor patients, making it a valuable resource for medical professionals.

**Extending the indication for CRS and HIPEC in PMP of appendical origin – a clinical analysis.**
ID: 701

Kefah Jaber[1 2], Julian Kirchner[3], Fabian Klingler[1], Jan Luca Skruodies[1], Alexander Rehders[1], Wolfram Trudo Knoefel[1], Georg Flügen[1 2]

[1]*Universitätsklinikum Düsseldorf, Klinik für Allgemein-, Viszeral- und Kinderchirurgie, Düsseldorf, Deutschland*, [2]*Universitätsmedizin Essen, Klinik für Allgemein-, Viszeral- und Transplantationschirurgie , Essen, Deutschland*, [3]*Universitätsklinikum Düsseldorf, Institut für diagnostische und interventionelle Radiologie, Düsseldorf, Deutschland*

**Background:**
Pseudomyxoma peritonei (PMP) is an extremely rare disease with an incidence of approximately 0.1-0.3 per 100,000 per year. This disease, characterized by mucin producing malignant cells, is often diagnosed as an incidental finding after an appendectomy, with appendix carcinomas known to be the most common cause of pseudomyxoma peritonei.  Historically, peritoneal carcinosis was classified as the final stage of cancer to be treated palliatively. Usually, the burden of disease is assessed intraoperatively using the peritoneal carcinosis index (PCI) established by Sugarbaker. In several entities, a PCI >20 is proposed as a cut off for CRS + HIPEC. However, such a cut off does not exist for PMP of appendical origin.

**Materials and methods:**
We included 20 patients presenting with PMP of appendical origin with very high PCI (median= 25) in our analysis. We evaluated the perioperative course and the clinical management of these patients following CRS + HIPEC. For this purpose, several pre-, intra- and postoperative indicators, including postoperative complications and length of hospital stay were collected and correlated with the survival data.  Additionally, preoperative CT-scans were revisited by a blinded radiologist and a radiologic PCI was assessed.

**Results:**
We found a correlation between the intraoperative PCI and the radiologic PCI. The length of ICU and hospital stay was not correlated with extend of PCI. Interestingly, despite the multivisceral resections performed, the rate of postoperative complications was not higher than in published cohorts with less extensive PMP. While median survival was limited in our extensive cases, it was never the less longer than in published comparable cohorts of palliatively treated patients.

**Conclusion:**
A modern multimodal treatment concept is indicated and useful even in extensive pseudomyxoma peritonei with a high peritoneal carcinosis index. Our results indicate, that a cut off for the intraoperative PCI is not suitable for PMP patients. A radiological PCI can be reliably used to assess intraoperative extent of PMP. Surgical approach as part of a multimodal treatment concept is a useful option for many patients who, until recently, were considered exclusively palliative cases

Abstracts – DCK 2024 – 141st Congress of the German Society of Surgery – Leipzig, April 24–26 • DOI 10.1515/iss-2024-2003
Innov Surg Sci 2024; 9 (Special Suppl 1): s18–s203

### Prognostic factors after resection of locally advanced non-functional pancreatic neuroendocrine neoplasm – an analysis from the German Cancer Registry Group of the Society of German Tumor Centers

ID: 763

Thaer Abdalla[1], Ulrich Wellner[1], Tobias Keck[1], Kim Honselmann Genannt Humme[1], Rüdiger Braun[1], Louisa Bolm[1], Stanislav Litkevych[1], Monika Klinkhammer-Schalke[2], Sylke Ruth Zeissig[2 3], Steffen Deichmann[1]

[1]*University Medical Center Schleswig Holstein- Campus Lübeck, Department of Surgery, Lübeck, Deutschland*, [2]*German Cancer Registry Group of the Society of German Tumor Centers - Network for Care, Quality and Research in Oncology (ADT), Berlin, Deutschland*, [3]*University of Würzburg, Institute of Clinical Epidemiology and Biometry (ICE-B), Würzburg, Deutschland*

**Background:**
The available literature regarding outcome after pancreatic resection in locally advanced non-functional pNEN (LA-pNEN) is sparse. Therefore, this study evaluates the current survival outcomes and prognostic factors in after resection of LA-pNEN

**Materials and methods:**
This population-based analysis was derived from 17 German cancer registries from 2000 to 2019. Patients with upfront resected non-functional non-metastatic LA-pNEN were included.

**Results:**
Out of 2776 patients with pNEN, 277 met the inclusion criteria. 137 (45%) of the patients were female. The median age was 63 ± 18 years. Lymph node metastasis was present in 45%. G1, G2 and G3 pNEN were found in 39%, 47% and 14% of the patients, respectively. Resection of LA-pNEN resulted in favorable 3-, 5- and 10-year overall survival of 79%, 74%, and 47%. Positive resection margin was the only potentially modifiable independent prognostic factor for overall survival (HR 1.93, 95% CI 1.71 – 3.69, p-value = 0.046), whereas tumor grade G3 (HR 5.26, 95% CI 2.09-13.25, p-value <0.001) and lym-phangiosis (HR 2.35, 95% CI 1.20 - 4.59, p-value = 0.012) were the only independent prognostic factors for disease-free survival.

**Conclusion:**
Resection of LA-pNEN is feasible and associated with favorable overall survival. G1 LA-pNEN with negative resection margins and absence of lymph node metastasis and lymphangiosis might be considered as cured, while those not fulfilling these criteria might be considered as a high-risk group for disease progression. Herein, negative resection margins represent the only potentially modifiable prognostic factor in LA-pNEN but seem to be influenced by tumor grade.

**Prognostic accuracy of the international classification for grading the resectability of pancreatic cancer**
ID: 776

Alexander Genchev[1], Ugur Göckcelli[1], Georg Wiltberger[1]

[1]*Aachen, Uniklinik RWTH, Aachen, Deutschland*

**Background:**
The 2017 international consensus criteria expanded the definition of anatomically borderline resectable pancreatic ductal adenocarcinoma to include both a biological and a conditional dimension. An inflammatory variable was mentioned but not included. To date, the prognostic accuracy for all individual subgroups is understudied and the impact of an inflammatory response is uncertain.

**Materials and methods:**
Between 2010-2022, a total of 419 patients who underwent pancreatic resection due to adenocarcinoma were included retrospectively in the study. They were divided into 12 groups according to the international consensus classification with all possible combinations of the different variables. Inflammatory variable was created based on Modified Glasgow Prognostic Score and neutrophil-to-lymphocyte ratio.

**Results:**
OS for the entire cohort was 24 months (95% CI, range 20.6 – 27.4 months). Recurrence occurred in 215 Patients (52.4%) and the median DFS was 9 months (95% CI, range 7.8 – 10.2 months). 324 patients (78%) staged PR–A, 54 (13%) BR-A and 41 (9%) LA-A. PR–A had median OS of 26 and median DFS of 10 months, BR-A – 26 months and 8 months and LA-A – 18 and 7 months respectively. 343 patients (82%) were PR-B and 76 (18%) BR-B with an OS of 29 months for the PR-B group and 11 months for the BR-B group. Patients with BR-I showed an OS of 23 months.

**Conclusion:**
The preoperative classification into anatomical, biological and conditional resectability criteria has a high prognostic accuracy. The inflammatory variable has been demonstrated to have a significant predictive role in determining the outcome.

**Influence of certification programme on treatment quality and survival for rectal carcinoma in Germany. Results of 13 cerftified centers collaborated to AN-institute.**
ID: 831

Mihailo Andric[1], Jessica Stockheim[1], Mirhasan Rahimli[1], Sara Al-Madhi[1], Sara Acciuffi[1], Maximilian Dölling[1], Roland S. Croner[1], Aristotelis Perrakis[1]

[1]*Universitätsklinikum Magdeburg, Klinik für Allgemein-, Viszeral-, Gefäß- und Transplantationschirurgie, Magdeburg, Deutschland*

**Background:**
The certification of oncological units as Colorectal Cancer Center (CrCC) has been proposed to standardize the oncological treatment and improve outcomes of colorectal cancer (CRC). The proportion of patients with CRC in Germany being treated in a certified center is around 53%. The effect of certification on treatment outcome have been lately critically discussed.
**Aim:**
Our aim was to investigate treatment outcomes in patients with rectal carcinoma in certified CrCC, in German hospitals of different medical care level.

**Materials and methods:**
We performed a retrospective analysis of prospective, multicentric database (AN Institute) of adult patients who underwent surgery for rectal carcinoma between 2002 and 2016. We included patients from 13 hospitals of different medical care levels (basic, priority and maximal care) in periods of 3 years (collective A: n=625) and 5 years (collective B: n= 563) before and after certification.

**Results:**
Certified CrCC showed a significant increase of minimally-invasive approach for rectal cancer surgery (A: 24% vs. 46%, p<0.001; B: 5% vs. 55%, p<0.001), of proportion of LARR (A: 37% vs. 45%, p=0.05) and of stapled anastomosis technique (A: 86% vs. 96%, p=0.019). However, we observed a significant prolonged mean duration of surgery in certified CrCC (A: 172 Min. vs. 186 Min., p= 0.011; B: 161 Min. vs. 192 Min, p< 0.001). The overall morbidity did not significantly improve (A: 38% vs. 31%, p=0.093; B: 32% vs. 38%, p=0.174), but the appearance of intraoperative complications (A: 6% vs. 2%, p=0.019) decreased significantly in CrCC-certified units. As far as the overall in-hospital mortality is concerned, we registered a positive trend in certified centers during the five-year period after the certification (B: 5% vs. 3%, p=0.190). The length of preoperative hospitalization (preop. LOS) has been shortened significantly (A: 3.13 days vs. 3.05 days, p=0.016, B: 4.7 vs. 4.1 days, p<0.001), while the overall length of in-hospital stay seemed also to be shorter in CrCC-centers (B: 20.32 vs. 19.54 days, p= 0.065). We also registered a clear advantage in detailed, high-quality histopathological examination as far as N-, L, V- and MERCURY-status are concerned. In the performed subgroup analysis, a significantly longer overall survival after certification is registered for maximal medical care units (B: p=0.029) and in patients with UICC stage IV-disease (B: p=0.041). In patients with UICC stadium I and III disease we registered a significant improvement of disease-free survival (A: UICC I: p=0.040; B: UICC III: p=0.050).

**Conclusion:**
The results of the present study indicate an improvement in terms of treatment quality and outcome in certified CrCC, enforced by certification-specific aspects such as more differentiated surgical approach, less intraoperative complications, and the multidisciplinary approach. Further prospective clinical trials are necessary to investigate the influence of certification in the treatment of CRC patients.

Abstracts – DCK 2024 – 141st Congress of the German Society of Surgery – Leipzig, April 24–26 • DOI 10.1515/iss-2024-2003
Innov Surg Sci 2024; 9 (Special Suppl 1): s18–s203

**Prediction of therapy response after resection of (metastasized) colorectal cancer using patient-derived micro-organospheres**
ID: 884

Nikolai Schleußner[1 2 3], Else Driehuis[4], Uraz Yaser[1], Ella Leugner[1], Julia Volk[2 3], Xiling Shen[5], Bruno Köhler[6 7], Thomas Schmidt[8], Christoph Michalski[1], Christoph Kahlert[1], Rene Jackstadt[2 3]

[1]*Universitätsklinikum Heidelberg, Allgemein-, Viszeral-und Transplantationschirurgie, Heidelberg, Deutschland*, [2]*Deutsches Krebsforschungszentrum (DKFZ), Cancer Progression and Metastasis, Heidelberg, Deutschland*, [3]*Heidelberg Institute for Stem Cell Technology and Experimental Medicine (HI-STEM gGmbH), Heidelberg, Deutschland*, [4]*Xilis BV, Utrecht, Netherlands*, [5]*Xilis INC., Durham, United States*, [6]*Nationales Centrum für Tumorerkrankungen, Heidelberg, Deutschland*, [7]*Deutsches Krebsforschungszentrum (DKFZ), Heidelberg, Deutschland*, [8]*Uniklinik Köln, Klinik für Allgemein-, Viszeral-, Tumor- und Transplantationschirurgie, Köln, Deutschland*

**Background:**
Patient derived organoids (PDO) are used as a preclinical prediction tool for therapy response, but the generation of PDOs is time-consuming and requires a lot of manual work, therefore, high-throughput drug screenings of PDOs take too long to guide clinical decision making. Furthermore, the tumor microenvironment as part of the heterogeneity plays a crucial role in therapy response and clinical outcome, which cannot be simulated with PDOs. Micro-organospheres (MOS) are microscale tumor models generated using an automatic microfluids droplet system enabling the rapid generation of thousands of patient-derived tumor avatars in few weeks. The MOS consist of PDOs as well as the various cells of the TME allowing quick and clinical-relevant drug assays.

**Materials and methods:**
A retrospective cohort of patients receiving colon, rectal or hepatic resection for colorectal cancer with and without multimodal approach was collected. The samples analyzed include primary and metastatic lesions and were used to generate MOS for further drug screenings for standard-of-care chemotherapies. The patients were divided into three groups including no treatment, neoadjuvantly and adjuvantly treated patients. The in-vitro treatment response was correlated to clinical and pathological outcome of the patients.

**Results:**
MOS could be generated of all different patient cohorts including primary and metastatic lesions and the time from isolation to screening was only days to weeks. The preliminary success rate differed between the groups: Whereas a high success rate could be shown in the treatment naïve cohort, n=8, >90%), the samples in the neoadjuvant group had a slightly diminished success rate, with outgrowth of about 50%, n=52, patient samples. Hereby, samples from patients treated neoadjuvantly with FOLFIRI showed a lesser likelihood to establish MOS compared to FOLFOX treated specimen. High-throughput drug screening of MOS were performed using the same treatment regimen as in the clinics. It revealed an individual treatment response for each patient. So far, correlation of the in vitro treatment response data to the clinical response using RECIST criteria of a small training cohort showed a sensitivity of 100% and a specificity 91% for the predictive value of the MOS.

**Conclusion:**
MOS can be rapidly generated and used for drug screening, showing a promising correlation to the clinical response. Furthermore, drug screening results were reached in a period of time (<4-6 weeks) that could allow a personalized adjuvant therapy for patients undergoing resection for advanced colorectal cancer.

Abstracts – DCK 2024 – 141st Congress of the German Society of Surgery – Leipzig, April 24–26 • DOI 10.1515/iss-2024-2003
Innov Surg Sci 2024; 9 (Special Suppl 1): s18–s203

**Proposal of a new visual analogue scale to describe the extend of lymphadenectomy in right-sided colcectomy for cancer.**
ID: 888

Frank Pfeffer[1], Petter Kalgraff[1], Kristin Lygre[2], Håvard Forsmo[1]

[1]*Haukeland hospital, Norway*, [2]*Haraldsplass Diakonale Sykehus, Norway*

**Background:**
Central lymphadenectomy in right-sided colon cancer involves dissection along the superior mesenteric axis. There is no clear consensus regarding the extend and the terminology of lymphadenectomy. Two sets of terms describe the lymph node harvest and dissection along embryological planes. The Japanese D3 classification refers to central lymph node stations (1) and the European complete mesocolic excision (CME) to dissection between embryological planes and central vascular tie (2). In Norway, due to the fear of major complications, the dissection and vascular ligation along the right side of the superior mesenteric vein is proposed as standard. The term complete D2 (cD2) is introduced. To overcome lack of consensus, a visual analogue scale (VAS) to describe the extend of lymphadenectomy was developed.

**Materials and methods:**
Patients operated for right-sided colon cancer at Haukeland University Hospital in Bergen, Norway between January 2021 and October 2023 were included. Data were collected and analyzed with a web-based database (Ledidi AS, Oslo, Norway). Details of the operation including the VAS score (figure 1) were provided by the operating surgeon immediately after the operation. A VAS score < 7,8 describes a D2 lymphadenectomy, a VAS score between 7,8 and 7,9 a cD2 and a VAS score between 8-10 a D3 lymphadenectomy. The quality of the specimen was described by the Benz classification system by the operating surgeon (3)

**Results:**
Data of 126 patients were analyzed. The extend of lymphadenectomy described by the VAS score correlated significantly with the number of lymph nodes harvested (r= 0,44; p< 0,001). The operations were classified as D2 (n= 22), cD2 (n= 60) and D3 (n= 39) by the surgeon. The VAS score differed significantly between the classifications. Data were given as mean (median, lower- upper Interquartile range). D2: 7,1 (7,0, 6,9-7,0), cD2: 8,1 (8,0, 8,0-8,4) and D3: 9,5 (9,7, 9,2-9,8) p< 0,001. The superior mesenteric vein was described as not visualized (n= 18), visualized lateral (n= 59) or complete (n= 44). The VAS score differed significantly between the groups. Not visualized: 7,1 (7,0, 6,9-7,5); lateral: 8,0 (8,0, 7,9-8,2) and complete: 9,4 (9,6, 8,9-9,8) p= 0,043. The extend of lymphadenectomy resulted in a better quality of the specimen. No specimen was graded as Type III (poorest). Distribution was Type 0 (best) (n= 64), Type I (n= 44) and Type II (poor) (n =9). The VAS score for Type 0 was 8,9 (8,7, 8,3-9,6), Type I: 7,9 (7,9, 7,5-8) and Type II: 7,2 (6,9, 6,5-7,9) p< 0,001.

**Conclusion:**
The proposed VAS score may be relevant to describe the extend of lymphadenectomy in right-sided colectomy for cancer. In contrast to categorical classifications as CME or D2/3 the score relates to anatomical landmarks and is not dependent on definitions and consensus. The extend of lymphadenectomy seems to have impact on the specimen quality and the number of lymph nodes harvested.

Literature:
1. Yamamoto S, Inomata M, Katayama H, Mizusawa J, Etoh T, Konishi F, et al. Short-term surgical outcomes from a randomized controlled trial to evaluate laparoscopic and open D3 dissection for stage II/III colon cancer: Japan Clinical Oncology Group Study JCOG 0404. Ann Surg. 2014;260(1):23-30.

2. Hohenberger W, Weber K, Matzel K, Papadopoulos T, Merkel S. Standardized surgery for colonic cancer: complete mesocolic excision and central ligation - technical notes and outcome. Colorectal Disease. 2009;11(4):354-64.
3. Benz S, Tannapfel A, Tam Y, Grunenwald A, Vollmer S, Stricker I. Proposal of a new classification system for complete mesocolic excison in right-sided colon cancer. Techniques in coloproctology. 2019;23(3):251-7.

Figure 1

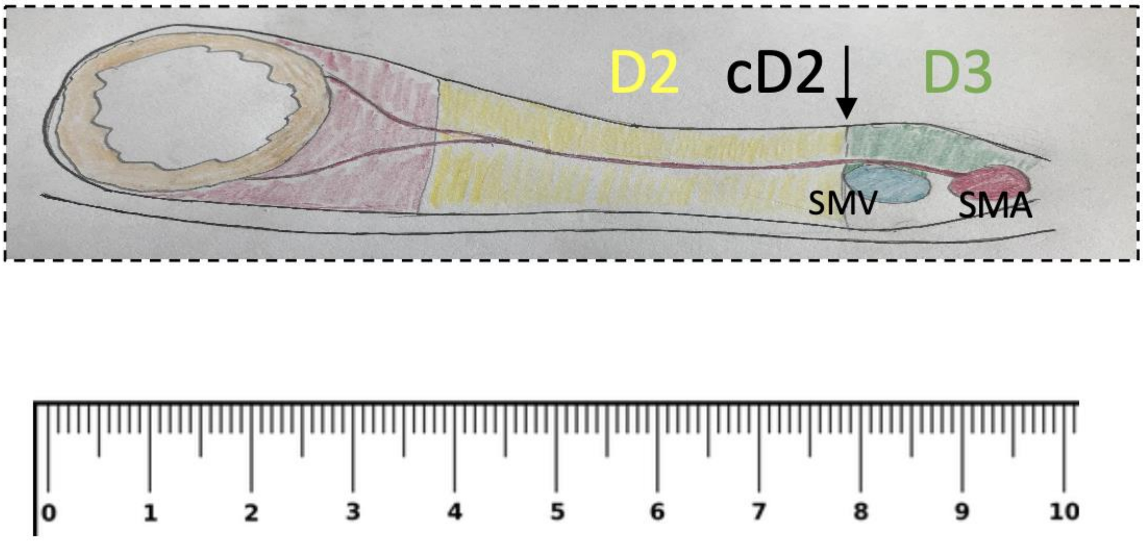

**Predictive factors for the extend of Hashimoto´s thyroiditis**
ID: 156

Kirsten Lindner[1], Elena Vorona[2], Andreas Pascher[3], Philipp Houben[3]

[1]*Lakumed, Endokrine Chirugie, Vilsbiburg, Deutschland*, [2]*Universitätsklinikum Münster, Medizinische Klinik B, Münster, Deutschland*, [3]*Universitätsklinikum Münster, Klinik für Allgemein-, Viszeral- und Transplantationschirurgie, Münster, Deutschland*

**Background:**
The possibility of surgery for Hashimoto's thyroiditis (HT) as a therapeutic option is discussed increasingly in the literature. The present study evaluates preoperative ultrasound (US) and thyroid peroxidase antibodies (TPO-Ab) concerning their predictive value for inflammation-induced adhesions of the thyroid as a possible decision-making aid.

**Materials and methods:**
Using our StuDoQ data from April 1, 2017, to June 1, 2023 (n=1520), 112 patients with histologically confirmed HT (male n=8, 7.1%) were included. 25 patients with papillary thyroid carcinoma as the primary diagnosis, 24 patients with hyperthyroidism and Graves' disease, and one with concomitant IgG4 thyroiditis were excluded.

**Results:**
HT was known preoperatively in 50% of the patients (n=56). 80.4% of all patients had no complaints, 3.6% had functional complaints, 4.5% had difficult hormone adjustment, 9.8% had local complaints, and 1.8% had several complaints. The main indication for the operation was the exclusion of malignancy in 68.8% (n=77), local complaints in 30.4% (n=34), and an accompanying autonomic adenoma in one patient (0.9%). US was performed preoperatively in all patients. In 41.1% (n=46), there was a homogeneous sonographic appearance of the thyroid gland, 23.2% (n=26) showed a slight heterogeneous echo pattern, and 31.3% (n=35) showed a clear one. In 4.5%, there was an overall nodular appearance of the thyroid. TPO-Ab was available in 48.2% (n=54) preoperatively. Of these, 27.8% (15/54) were negative. In 38.9% (21/54), the value was increased up to 500IU/ml, in 11.1% (6/54) between 500-1000IU/ml, and in 22.2% (12/54) >1000IU/ml.
There was no significant correlation between TPO-Ab and US appearance (p=0.733). However, there was a trend towards a more homogeneous echo pattern with normal or slightly increased TPO-Ab up to 500IU/ml (16/36, 44.4%). In contrast, in the case of TPO-Ab>1000IU/ml, there was an increased inhomogeneous image (8/12, 66.7%).
There was neither a connection between intraoperative inflammation-induced adhesions and TPO-Ab (p=0.867) nor for US appearance (p=0.412). However, there was a trend towards fewer adhesions with homogeneous and slightly heterogeneous sonographic appearance (41/67, 61.2%) in contrast to a high degree of adhesions in clear heterogeneous echo patterns (22/35, 62.9%). The extent of the intraoperative adhesions did not influence postoperative complications (recurrent nerve palsy p=0.967, hypoparathyroidism (included only patients with thyroidectomy n=56), p=0.230).

**Conclusion:**
Explicit predictive factors for intraoperative adhesions in HT could not be identified. However, preoperative diagnostics can be helpful. While a preoperative US is often easily available, there is no urgent need for the preoperative determination of the TPO-Ab.

**Portal Vein Resection in Pancreatic Neuroendocrine Neoplasms.**
ID: 384

Anna Nießen[1 2], Ulla Klaiber[3], Madgalena Lewosinska[1], Felix Nickel[1 2], Franck Billmann[1], Ulf Hinz[1], Markus W. Büchler[1 4], Thilo Hackert[1 2]

[1]*Heidelberg University Hospital , Department of General, Visceral and Transplantation Surgery, Heidelberg*, [2]*University Hospital Hamburg-Eppendorf, Department of General, Visceral and Thoracic Surgery, Hamburg, Deutschland*, [3]*Medical University of Vienna, Department of General Surgery, Division of Visceral Surgery*, [4]*Champalimaud Foundation, Botton-Champalimaud Pancreatic Cancer Center, Lisboa, Portugal*

**Background:**
Neuroendocrine neoplasms of the pancreas (pNEN) are rare tumors with increasing incidence. With advancements in pancreatic surgery for borderline resectable pancreatic cancer (PDAC), the development of portal vein resection (PVR) techniques has increased the amount of potential radical (R0) resections and PVR has become standard procedure in surgery for PDAC. Data on outcome of locally advanced or borderline resectable pNEN and especially the incidence, perioperative and long-term outcomes of PVR for pNEN are scarce. However, the available data however suggests an acceptable perioperative and oncological outcome for radical resection and thus surgery offers the only cure in those patients. This study aimed to analyze outcome and prognostic factors of portal vein resection (PVR) in surgery for pNEN.

**Materials and methods:**
Consecutive patients were identified from a prospectively collected database. PVR was classified according to the International Study Group of Pancreatic Surgery (ISGPS). Clinicopathological features, overall (OS) and disease-free survival (DFS) were assessed and compared to standard resection (SR) in a 1: 2 matched-pair analysis.

**Results:**
54 of a total of 666 (8%) resected pNEN patients underwent PVR, including 7 (13%) tangential resections with venorrhaphy (ISGPS type 1), 2 (4%) patch reconstructions (ISGPS type 2), 35 (65%) end-to-end anastomoses (ISGPS type 3) and 10 (19%) graft interpositions (ISGPS type 4). 52% of those underwent pancreatoduodenectomy, 22% distal pancreatectomy and 26% total pancreatectomy. Postoperative PV thrombosis occurred in 19%. PV thrombosis occurred in one patient after type 2 venous resection, 4 patients after type 3 venous resection and 5 patients after type 4 venous resection. Analysis of the type of pancreatic resection showed a significant difference in PV thrombosis rate occurring in 3 of 12 DP (25%), 2 of 28 PD (51.9%) and 5 of 14 (26%) TP patients ($p = 0.043$). Postoperative pancreatic fistula grades B/C (9% versus 16%, $p = 0.357$), complications Clavien-Dindo Grade 3b or higher (28% versus 13%, $p = 0.071$) and 90-day mortality rate (2% each) was not significantly different compared to 108 matched patients. 5-year OS was 45% (SR: 68%, $p = 0.432$), 5-year DFS 25% (SR: 34%, $p = 0.716$). Radical resection (R0) was associated with 5-year OS of 51% and 5-year disease-specific survival of 75%.

**Conclusion:**
This is the largest single-center analysis evaluating perioperative and long-term outcome of PVR for pNEN. Postoperative complication rate after PVR is comparable to SR. 90-day mortality is low. PVR can offer excellent disease-specific as well as overall survival if a radical (R0) resection can be achieved and should therefore be considered in patients with advanced pNEN.

**The role of the microbiome in pancreatic cancer and its precursors. The MiBiPanc systematic review**
ID: 99

Alexander Werba[1], My-Lan Kha[2], Rosa Klotz[1], Susanne Roth[1], Eva Kalkum[1], Solveig Tenckhoff[1], Frank Pianka[1]

[1]*Chirurgische Klinik, Heidelberg, Deutschland*, [2]*Medizinische Mikrobiologie und Hygiene, Heidelberg, Deutschland*

**Background:**
Pancreatic ductal adenocarcinoma (PDAC), a malignancy of the exocrine pancreas, is the most common form of pancreatic cancer and one of the deadliest malignancies with a 5-year survival of 10%. Surgery remains the only potentially curative treatment, but only a small number of patients in early stages are amenable for surgical treatment. Chemotherapy as a standard of care faces multiple obstacles in patients with advanced disease, such as drug-resistance, toxicity, and other treatment failures. Due to limited therapy options, mortality is high and predicted to soon become the second leading cause of cancer mortality. Therefore prevention, early diagnostic and new effective treatment strategies are needed.
Multiple factors are involved in the development of pancreatic cancer e.g., age, diet, lifestyle, inflammation, and microbiome. Chronic pancreatitis (CP) and the presence of Intraductal papillary mucinous neoplasms (IPMN), mucinous cystic neoplasms (MCN) or pancreatic intraepithelial neoplasia (PanIN) confer an increased risk. Depending on size, location, histology and biochemistry, the risk of cancer development is variable, requiring surveillance or even surgical resection. Several studies have already investigated the role of the gut microbiome in premalignant cells and pancreatic cancer progression. For example, a higher level of bacterial 16s DNA copies in cyst fluid from IPMN and invasive cancer could be detected. Also, some studies could identify a unique gut microbiome composition in pancreatic cancer.
The role of microbiota in carcinogenesis has been proven in various tumor types (e.g., Fusobacterium nucleatum in oral squamous cell carcinoma and colorectal cancer, Helicobacter pylori in gastric cancer). Nevertheless, up to date the existence of the pancreatic microbiome is still controversially discussed.

**Materials and methods:**
Under the directive of the Preferred Reporting Items for Systematic Reviews and Meta-Analyses (PRISMA) guidelines a literature search in Medline, Web of Science, Cochrane Central Register of Controlled Trials (CENTRAL) and EMBASE will be performed. All stages of the study selection, quality assessment and data extraction will be independently carried out by two reviewers. Any disagreement will be resolved by consensus, or by consultation with a third reviewer. All outcome measures will be reported in a descriptive summary.
The dichotomous measures, in how much of the measured samples a specific bacterium (or strain, or family) was identified, will be pooled as single proportions in a meta-analysis resulting in an overall proportion regarding the presence of bacteria. If studies are to be identified that report on a comparison between groups, for example samples of healthy tissue versus samples of pancreatic disease tissue regarding the presence of bacteria, a meta-analysis will be conducted regarding the mentioned comparison.

**Results:**
The systematic literature search was not restricted to date, language or country of publication and the preliminary search yielded 3.086 results at the time of writing (Medline). Randomized controlled trials (RCT), prospective comparative cohort studies, and retrospective comparative cohort studies will be used for the qualitative and quantitative synthesis of the systematic review. Animal studies, meeting abstracts, letters/comments/editorials, and publications for which the full text is irretrievable will be excluded. The methodological quality of included RCT and non-randomized studies will be assessed using the Cochrane Collaboration tool for assessing risk of bias 2.0 and the ROBINS-I-tool when necessary. In addition, to detect the risk of bias the Checklist for critical Appraisal and data extraction for systematic reviews of prediction Modelling Studies (CHARMS) as well as the Prediction model Risk of Bias Assessment Tool (PROBAST) for in-depth assessment of risk of bias will be used. Both tools will work complementary with Grading of Recommendations Assessment, Development and Evaluation (GRADE) tool for evaluation of evidence.

**Conclusion:**

Due to the limited therapeutic options and poor prognosis of pancreatic cancer, research in this field gains more and more relevance, as the identification of new strategies for novel screening tests, early diagnostics, chemoprevention and targeted therapies is of utmost importance.

This systematic review will analyze and summarize the latest data on the impact of microbiome on the development of pancreatic cancer and will focus on the early steps of carcinogenesis. Recent systematic reviews did not include microbiome studies with a relation to cancer or analyzed IPMN and PDAC without considering microbiome studies on chronic pancreatitis or did not differentiate between pancreatic cancer types.

Most important are the data about the microbial composition and methods to obtain these data. A comparison of the microbial composition in various stages before and during pancreatic carcinogenesis is essential to identify potential microbial members or carcinogenic bacteria for further investigations. This will help to enhance the necessity of studies about bacterial members in precancerous stages to prevent cancer development and to form new research hypotheses. Additionally, we will discuss some proposed mechanisms in which the microbiome elicits its tumorigenic effect in PDAC, and the potential for targeting the microbiome for therapeutic purposes.

The aim of the presented systematic review is to summarize the complete available body of evidence to serve as a landmark for pancreatic microbiome research.

This study is funded by the German federal ministry of education and research (BMBF – Bundesmisterium für Bildung und Forschung)

Abstracts – DCK 2024 – 141st Congress of the German Society of Surgery – Leipzig, April 24–26 • DOI 10.1515/iss-2024-2003
Innov Surg Sci 2024; 9 (Special Suppl 1): s18–s203

**Elevated APRI score and ALBI grade as indicators of chemotherapy-induced hepatic damage, can predict postoperative complications following curative resection of colorectal liver metastases.**
ID: 177

Gerrit Josephs[1], Alexandros Chrysos[1], Shari David[1], Robert Siepmann[2], Daniel Truhn[2], Theresa H. Wirtz[3], Tom Lüdde[4], Steven W.M. Olde Damink[5], Bas Groot Koerkamp[6], Florian Vondran[1], Iakovos Amygdalos[1]

[1]*University Hospital RWTH Aachen, Department of General, Visceral, and Transplantation Surgery*, [2]*University Hospital RWTH Aachen, Department of Diagnostic and Interventional Radiology*, [3]*University Hospital RWTH Aachen, Department of Internal Medicine III*, [4]*University Hospital Düsseldorf, Department of Gastroenterology, Hepatology and Infectiology*, [5]*Maastricht University Medical Center+, Department of Surgery*, [6]*Erasmus MC Rotterdam, Department of Surgery*

**Background:**
In patients with colorectal liver metastases (CRLM), preoperative chemotherapy may induce hepatic injury, leading to an elevated aspartate aminotransferase-to-platelet ratio index (APRI), as well as higher albumin-bilirubin grade (ALBI). This study aims to examine the potential link between these scoring parameters and the occurrence of postoperative complications, in patients undergoing major liver resection for CRLM.

**Materials and methods:**
This retrospective study included patients who underwent major curative liver resection for CRLM following preoperative chemotherapy at the University Hospital RWTH Aachen between 2010-2021. Exclusion criteria encompassed missing laboratory parameters or chemotherapy data, resection of recurrent metastases and explorative laparotomies without curative resection. Preoperative laboratory parameters were analyzed to calculate APRI and ALBI scores. Receiver-operating characteristic (ROC) analyses were performed to predict postoperative complications according to Clavien-Dindo Grade 3 or above (CD≥3). Youden Index (YI) analysis was employed to determine the optimal cutoff values of APRI and ALBI for prediction of CD≥3 complications. Group comparisons were conducted using either the chi-square test or the Mann-Whitney- U test, as appropriate. Continuous variables were analyzed using the Spearman correlation coefficient.

**Results:**
A total of 115 patients were included in the study. Both APRI (AUC = 0.64; $p = 0.021$) and ALBI (AUC = 0.68; $p = 0.004$) were shown to be predictive for CD≥3 complications and the relevant cut-offs were calculated as 0.41 (YI = 0.26) and -2.31 (YI = 0.33), respectively. Patients with scores above / below the cut offs were shown to have significantly different rates of CD≥3 complications (APRI: 56% vs 33%, $p$=0.024, ALBI: (84% vs 35%, $p$<0.001). Elevated APRI scores exhibited significant correlations with oxaliplatin administration (p = 0.004), increased spleen volume (p < 0.001), prolonged hospital ($p$ = 0.007) and ICU stays ($p$ = 0.008), and a higher total comprehensive complication index (CCI, $p$ = 0.028). Similarly, higher ALBI scores were significantly associated with elevated APRI scores ($p$ = 0.008), increased use of intraoperative transfusions ($p$ < 0.001), extended hospital ($p$ < 0.001) and ICU stays ($p$ < 0.001), as well as higher CCI scores ($p$ < 0.001).

**Conclusion:**
In patients with preoperative chemotherapy and major resection of CRLM, elevated APRI and ALBI scores are valuable predictors of postoperative morbidity. Therefore, these parameters could be used to detect patients at increased risk due to chemotherapy induced hepatic damage. Further studies are needed to assess the impact on larger cohorts and to explore preventive strategies for managing chemotherapy-induced hepatic injury.

**A comparison of clinical risk scores for survival prediction after curative-intent resection of colorectal liver metastases.**
ID: 178

Felix Schmidt[1], Katharina Jöchle[1], Alexandros Chrysos[1], Lea Hitpass[2], Daniel Truhn[2], Theresa H. Wirtz[3], Tom Lüdde[4], Steven W.M. Olde Damink[5], Bas Groot Koerkamp[6], Andreas Lambertz[1], Sven A. Lang[1], Florian Vondran[1], Iakovos Amygdalos[1]

[1]*University Hospital RWTH Aachen, Department of General, Visceral and Transplantation Surgery*, [2]*University Hospital RWTH Aachen, Department of Diagnostic and Interventional Radiology*, [3]*University Hospital RWTH Aachen, Department of Internal Medicine III*, [4]*University Hospital Düsseldorf, Department of Gastroenterology, Hepatology and Infectiology*, [5]*Maastricht University Medical Center, Department of Surgery*, [6]*Erasmus MC Rotterdam, Department of Surgery*

**Background:**
Colorectal liver metastases (CRLM) are the predominant factor limiting survival in patients with colorectal cancer and radical resection is the best option for long-term survival. However, patients present with varying clinicopathological and oncological characteristics, necessitating careful planning of treatment strategies and preoperative patient selection. To this end, several clinical risk scores (CRS) have been developed to predict oncological outcomes, such as overall survival (OS), after liver resection for CRLM. In this study, we compare the predictive capability of 11 previously described CRS on a monocentric cohort.

**Materials and methods:**
This retrospective study included 528 patients, who underwent curative-intent liver resection for CRLM between 2010-2021, at the University Hospital RWTH Aachen. Patients undergoing resection of recurrent metastases or explorative laparotomies only, as well as those who passed away within 90 days after surgery, were excluded. The following 11 CRS were assessed: Fong, Nordlinger, Nagashima, Konopke, Basingstoke Predictive Index (BPI), tumour burden score (TBS), resection severity index (RSI), Kulik, *RAS*-mutation-CRS, modified TBS (mTBS), genetic and morpholigal evaluation (GAME) score. Differences in survival between patient groups stratified through each CRS, were compared using Kaplan-Meier analyses and the log-rank test. The predictive ability of each CRS was evaluated using the Akaike-Information-Criterion (AIC) and Harrell's-C-Index for OS, and AUC-analyses for 1-year and 5-year survival.

**Results:**
Median OS for the whole cohort was 26 months (95% CI 23-28 months). Apart from RSI, all CRS could stratify patients into groups with statistically significant differences in OS: Fong, $p$=0.007; Nordlinger, $p$<0.001; GAME-score, $p$=0.006; Nagashima, $p$<0.001; Konopke, $p$<0.001; BPI, $p$<0.001; TBS, $p$<0.001; RSI, $p$=0.570; Kulik, $p$<0.001; *RAS*-mutation-CRS, $p$<0.001; mTBS, $p$<0.001. Of all scores, mTBS consistently performed within the top 3 regarding predition of OS (AIC 1725, C-Index 0.61). The TBS (AUC= 0.654, $p$= 0.001) and mTBS (AUC= 0.62, $p$< 0.001) scores were shown to be the most predictive of 1-year- and 5-year-survival, respectively.

**Conclusion:**
This study showed, that almost all externally developed and validated CRS could stratify patients undergoing liver resection for CRLM according to postoperative survival. Among these, the mTBS was consistently among the best performing CRS.

**Epidemiological outcomes of German high-volume centres compared to German and international benchmark data.**
ID: 196

Jan Heil[1], Svenja Sliwinski[1], Franziska Stickl[1], Hanan El Youzouri[1], Michael Heise[1], Ekaterina Petrova[1], Wolf Otto Bechstein[1], Andreas Schnitzbauer[1]

[1]*Klinik für Allgemein-, Viszeral-, Transplantations- und Thoraxchirurgie, Uniklinik Frankfurt, Frankfurt, Deutschland*

**Background:**
In a recent analysis of hospital discharge data by our group, an unexpected high mortality rate of 10.4% was demonstrated after major liver resection in Germany. Additionally, an association between caseload and postoperative outcome was shown as high caseload centres provided a lower mortality. In this current study, we performed a caseload-dependent analysis of the postoperative outcome of primary liver tumours. Outcome data were also compared to international benchmark data.

**Materials and methods:**
All patients of centres participating in the StuDoQ liver registry were retrospectively analysed that had liver resection for cholangio- (CCC) or hepatocellular carcinoma (HCC) between 2019 and 2021. Participating centres were classified into 2 categories depending on the annual number of liver resections performed. Outcome quality was defined by a selection of parameters that were considered to be determinant for a textbook outcome: major complication (Dindo-Clavien ≥3A), 90-day readmission and mortality rate, and length of hospital stay. Factors that might have an influence on the outcome quality were assessed by multivariate analysis using stepwise regression.

**Results:**
Overall, 4203 cases have been entered into the registry in 4 years, whereby 1371 of those (33%) had liver surgery for CCC (n=665 (49%)) or HCC (n=706 (51%)). In centres with a caseload of >100 liver resections per year (n=5), major complication and 90-day readmission rates, and proportion of patients hospitalised for >30 days were significantly higher (figure 1) compared to centres with <100 resections per year (n=25). There was no difference in the 90-day mortality between centres. In the multivariate analysis, only resection of ≥3 liver segments and bile duct reconstruction seemed to have an influence on the outcome quality, but not the caseload of centres.

**Conclusion:**
This first volume-outcome assessment of primary liver tumours from the StuDoQ liver registry showed significant differences between centres, although mortality <u>did not</u> differ between centres. However, outcome quality seems not to be depended on the centre size, according to the MVA in certified and highly specialized hepatobiliary centres. Moreover, German (high-volume) centres provide comparable outcome quality to recently published international benchmark data.

Figure 1

Abstracts – DCK 2024 – 141st Congress of the German Society of Surgery – Leipzig, April 24–26 • DOI 10.1515/iss-2024-2003
Innov Surg Sci 2024; 9 (Special Suppl 1): s18–s203

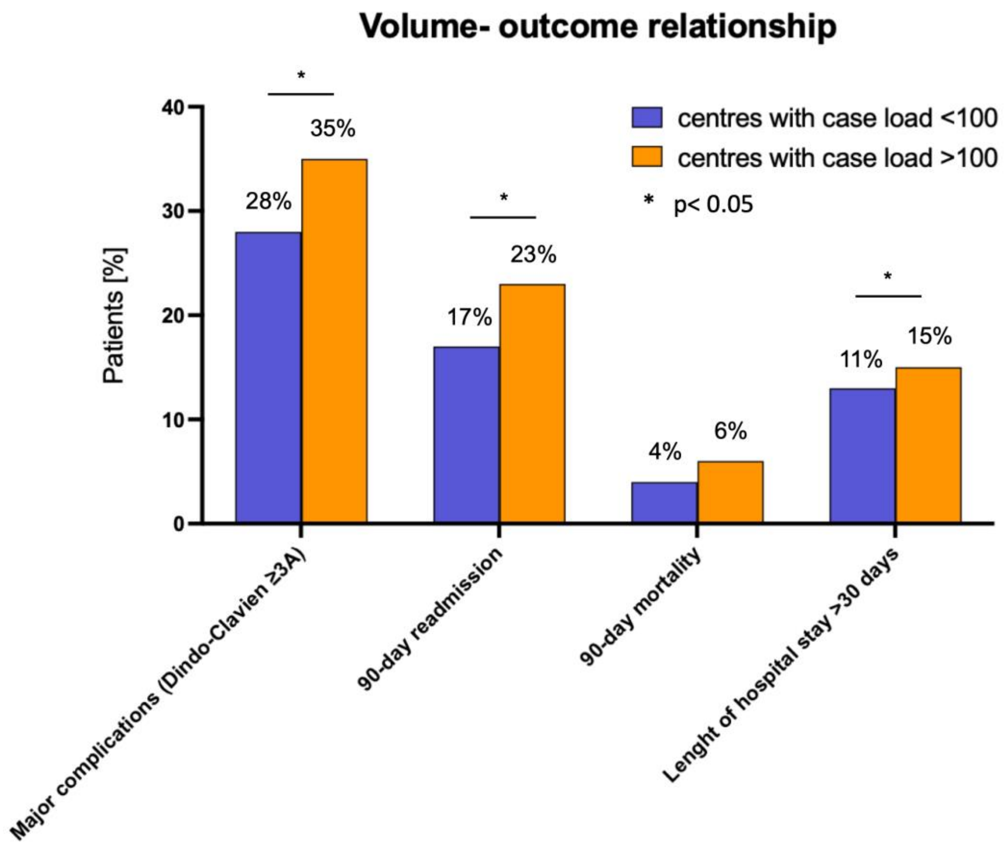

**Preoperative sarcopenia at the T4 vertebral level predicts postoperative major complications in patients undergoing major liver resection for colorectal metastases.**
ID: 224

Suzan Elmaagacli[1], Gregory van der Kroft[2], Franziska Meister[1], Robert Siepmann[3], Daniel Truhn[3], Johannes Bickenbach[4], Bas Groot Koerkamp[2], Steven W.M. Olde Damink[5], Florian Vondran[1], Iakovos Amygdalos[1]

[1]*University Hospital RWTH Aachen, Department of General, Visceral and Transplantation Surgery*, [2]*Erasmus MC Rotterdam, Department of Surgery*, [3]*University Hospital RWTH Aachen, Department of Diagnostic and Interventional Radiology*, [4]*University Hospital RWTH Aachen, Department of Operative Intensive Care and Intermediate Care*, [5]*Maastricht University Medical Center+, Department of Surgery*

**Background:**
Colorectal liver metastases (CRLM) are the predominant factor limiting survival in patients with colorectal cancer. Patients requiring major resection for CRLM are at increased risk of perioperative morbidity. This study examines the predictive capability of skeletal muscle index (SMI) measurements carried out on preoperative computed tomographies (CT) of the thorax, in terms of postoperative complications.

**Materials and methods:**
Patients undergoing major curative liver resection for CRLM between 2010-2021 at the University Hospital RWTH Aachen, with a preoperative CT scan of the thorax within 6 weeks of surgery, were eligible for this retrospective study. Patients with recurrent metastases or explorative laparotomies without resection were excluded. The skeletal muscle index at T4 vertebral level (SMI-T4) was measured using 3D-Slicer software (version 4.11.20210226 including Chest Imaging Platform extension). Receiver-operating characteristic (ROC), area under the curve (AUC) and Youden Index (YI) analyses were used to define a cut-off value for SMI-T4, for the prediction of major surgical complications, defined as Clavien-Dindo grade 3b or higher (CD≥3b). Differences between patients with SMI-T4 below and above the cut-off were examined with chi square and Mann-Whitney U tests, and logistic regression was used to determine independent risk factors for the development of CD≥3b complications.

**Results:**
A total of 123 patients were included, of which 31 (25%) developed CD≥3b complications. The ROC analyses were almost significant for the whole cohort (AUC=0.62, p=0.053) and significant for patients undergoing open resections (n=100, 81%) with an AUC of 0.66 (p=0.01). In this group of patients, a cut-off value for SMI-T4 of 55cm2/m2 (YI=0.264) was calculated. Patients with SMI-T4<55cm2/m2 suffered from significantly higher rates of CD≥3b complications (44% vs 22%, p=0.026). Logistic regression showed an increased risk of CD≥3b complications in patients with SMI-T4<55cm2/m2 (odds ratio 2.75, 95%CI 1.11-6.78, p=0.028).

**Conclusion:**
Preoperative CT-based measurements of SMI at the T4 vertebral level can predict the development of CD≥3b complications after major open liver resection for CRLM. These patients could benefit from aggressive pre-habilitation and postoperative physiotherapy. Further studies with a larger cohort and prospective design are necessary to explore the value and limitations of this technique, particularly in patients undergoing minimally invasive liver resection.

**Pericystectomy and liver scoring systems in cystic echinococcosis: A retrospective one-center review study**
ID: 239

Tristan Wagner[1], Sebastian Struck[1], Raphael Stier[1], Thorsten Persigehl[2], Christiane J. Bruns[1], Dirk Ludger Stippel[1], Michael Thomas[1]

[1]*Universitätsklinikum Köln (AÖR), Klinik und Poliklinik für Allgemein-, Viszeral- und Tumorchirurgie, Köln, Deutschland*, [2]*Universitätsklinikum Köln (AÖR), Institut für Diagnostische und Interventionelle Radiologie , Köln, Deutschland*

**Background:**
The primary objective of this study is to provide comprehensive insights into the surgical procedure of pericystectomy in patients diagnosed with cystic echinococcosis. Cystic echinococcosis, caused by the parasitic tapeworm Echinococcus granulosus, is a significant public health concern worldwide.
Controversy exits regarding the best surgical treatment. In all surgical approaches the goal is to eradicate the parasite and avoid intraoperative spillage of cyst contents. Pericystectomy hereby provides a radical treatment, removing the whole cyst while preserving healthy liver tissue. This study aims to address several key aspects related to pericystectomy and to investigate its validity in the treatment of cystic echinococcosis. Furthermore, this study was designed to investigate whether frequently used liver scoring systems that evaluate liver function and liver fibrosis can be used to establish a possible screening method in an E. granulosus infection.

**Materials and methods:**
A total of 36 patients who had been diagnosed with cystic echinococcosis underwent therapeutic intervention using the surgical technique known as pericystectomy between 2000 and 2023 at the University clinic of Cologne, Germany. The surgical technique of pericystectomy was subject to a comprehensive analysis considering multiple parameters to evaluate effectiveness and safety. The study focused on assessing surgery time, blood loss, postoperative complication, and recurrence rate in comparison to other known surgical techniques used to treat cystic echinococcosis.
To evaluate a possible connection between an E. granulosus infection and liver disfunction and/or fibrosis frequently used scoring systems such as MELD-Score (Model for End-Stage Liver Disease), the updated MELD 3.0, Child-Pugh-Score and the Fib-4-Score were assessed preoperatively. Furthermore, values for aspartate transaminase (AST), alanine transaminase (ALT), gamma-glutamyl transferase (GGT) and alkaline phosphatase (AP) were analyzed preoperatively and postoperatively to evaluate liver function.

**Results:**
Pericystectomy was performed on 36 patients with a total of 47 cysts. The mean age was 40.93 (± 12.44). Preoperative adjunctive anthelmintic therapy was administered with a mean time of 7.2 weeks. Multiple cysts were found in 30.56 % of cases. Singular cysts occurred in 69.44 %. The right liver lobe was affected in 91.49 %. Ectopic cysts were detected in 4 patients (lung n=3; spleen n=1). Preoperative measurements of ALT, AST, GGT and AP were not significantly elevated. The MELD, MELD 3.0, Child-Pugh-Score and Fib-4-Score did not show signs of increased liver failure or liver fibrosis preoperatively in cases with confirmed E. granulosus infections. Mean surgical time of pericystectomy was 232.78 min (± 76.62) with an average blood loss of 402.94 ml (± 182.96). The relevant complication rate (Clavien-Dindo >III) was 2.7 %. No postoperative liver failure was observed. The average hospital stay was 13 days (± 5 days). No recurrent disease could be detected after a mean follow up time of 120 months.

**Conclusion:**
Due to the solid shell of the echinococcus cysts, tissue-sparing surgical removal is possible without an increased rate of intraoperative spillage. Vital liver parenchyma can therefore be preserved. Despite a longer surgery time, average relevant postoperative complication rates (Clavien-Dindo III) were lower in comparison to other open or laparoscopic approaches. Biliary complication occurred with a comparable rate. The mean complication and bile leak rates must be considered in a relative context, given that this study included a higher number of multicystic cases compared to the average rate reported in the literature. With a mortality rate of 0%, pericystectomy can be considered a safe surgical procedure. After pericystectomy no recurrent disease could be documented after a follow up time of 120 months. Liver scoring systems (MELD, MELD 3.0, Child-Pugh score, Fib-4 Score) do not appear to be clinically relevant as screening markers in an infestation with E. granulosus.

### The benefit of adjuvant therapy in patients with duodenal adenocarcinoma and intestinal subtype ampullary carcinoma

ID: 241

Louisa Bolm[1 2], Sarah Finton[2], Martina Nebbia[3], Natalie Petruch[1 2], Carlos Férnandez-Del Castillo[2], Motaz Qadan[2], Keith Lillemoe[2], Ulrich Wellner[1], Marius Distler[4], Carolin Zimmermann[4], Jürgen Weitz[4], Felix Rückert[5 6], Nuh Rahbari[5], Christoph Reissfelder[5], Gennaro Nappo[3], Tobias Keck[1], Alessandro Zerbi[3], Cristina Ferrone[2 7]

[1]*Universitätsklinikum Schleswig-Holstein, Campus Lübeck, Klinik für Chirurgie, Deutschland*, [2]*Massachusetts General Hospital, Department of Surgery, Boston, United States*, [3]*Humanitas Milano, Department of Surgery, Italy*, [4]*Universitätsklinikum Dresden, Klinik für Chirurgie*, [5]*Universitätsklinikum Mannheim, Klinik für Chirurgie, Deutschland*, [6]*Diakonissen-Stiftungs-Krankenhaus Speyer, Klinik für Chirurgie*, [7]*Cedars' Sinai Los Angeles, Department of Surgery, United States*

**Background:**
Duodenal adenocarcinoma (DUOAC) and intestinal subtype ampullary carcinoma (int AMPAC) share a similar histological differentiation but the benefit of adjuvant therapy remains unclear. The objective of this study is to define the role of adjuvant therapy in DUOAC and int AMPAC.

**Materials and methods:**
Patients undergoing curative-intent surgical resection for DUOAC and int AMPAC between 2010 and 2021 at five high-volume centers were included. Patient baseline, perioperative and long-term oncological outcomes were evaluated. Statistical testing was performed with SPSS 25 (IBM).

**Results:**
A total of 136 patients with DUOAC and 171 with int AMPAC were identified. Patients with DUOAC had more advanced tumors than those with int AMPAC. Median overall survival (OS) in DUOAC patients was 101 months versus 155 months for int AMPAC patients (p=0.098). DUOAC had a higher rate of local (14.1% vs. 1.2%, p<0.001) and systemic recurrence (30.4% vs. 3.5%, p<0.001). Adjuvant therapy failed to improve overall survival in all patients with DUOAC and int AMPAC. For DUOAC, patients with perineural invasion, but not other negative prognostic factors had improved OS rates with adjuvant therapy (72m vs. 44m, p=0.044). Int AMPAC patients with N+ (190m vs. 57m, p=0.003), T3-4 (177m vs. 59m, p=0.050) and perineural invasion (150m vs. 59m, p=0.019) benefitted from adjuvant therapy.

**Conclusion:**
While adjuvant therapy fails to improve OS in all patients with DUOAC and int AMPAC, it is beneficial in DUOAC patients with perineural invasion and in int AMPAC patients with T3-4 tumors, positive lymph nodes, and perineural invasion.

**An R0 resection margin does improve overall survival after PDAC resection– real-world evidence from 3309 cases from the Association of German Tumor Centers**
ID: 282

Lennart von Fritsch[1], Jannis Duhn[1], Kim Honselmann Genannt Humme[1], Louisa Bolm[1], Rüdiger Braun[1], Markus Kist[1], Thaer Abdalla[1], Sylke Zeissig[2], Kees Kleihues-van Tol[2], Tobias Keck[1], Monika Klinkhammer-Schalke[2], Richard Hummel[3], Ulrich Wellner[1]

[1]*Department of Surgery, University Medical Center Schleswig-Holstein, Campus Lübeck, Lübeck, Deutschland*, [2]*Network for Care, Quality and Research in Oncology (ADT), German Cancer Registry Group of the Society of German Tumor Centers, Berlin, Deutschland*, [3]*Department of Surgery, University Hospital Greifswald, Greifswald, Deutschland*

**Background:**
Pancreatic ductal adenocarcinoma (PDAC) is one of the most common malignant neoplasms1. The only curative treatment option is surgical resection of the tumor. Resection margins have long been a widespread instrument to evaluate the success of the operation. Even though the concept seems intuitively plausible, early studies did not find a significantly improved outcome of R0 over R12. This was attributed to a lack in a standardized pathological workup and an inconsistent definition of R0, resulting in large variations of reported R1-rates3. Interestingly, more recent studies using a standardized pathology protocol or subgroup analyses for different R0 definitions still yielded contrary results4,5.
The aim of this study was to evaluate the prognostic value of resection margins in surgery of PDACs using real-world data from the Association of German Tumor Centers (ADT).

**Materials and methods:**
The study was carried out according to the data use regulations of the ADT. Patients with one or more distant metastases (M+) at diagnosis, neoadjuvant treatment, no surgical treatment, a R2-resection, or palliative operation were excluded. Differences between the R0 and R1 group were assessed using a Mann-Whitney-U test or chi-square test as appropriate. Overall survival (OS) or disease-free survival (DFS) was defined as the time from the date of surgery to the date of death/recurrence of disease or last follow-up (censored). Subgroup analyses were performed after stratifying for the type of surgery (pancreatoduodenectomy [PD], distal pancreas resection/pancreas left resection [DPR], or total pancreatectomy [TPE]). Survival analyses were performed using the Kaplan-Meier-method and compared using the log-rank test. Clinically significant difference was assumed for a at least 10% difference or 6 months in the mean OS or DFS. Furthermore, we performed a 1:1 case-control matching including the T-status, N-status, grading, and perineural invasion and repeated Kaplan-Meier analyses. To evaluate the influence of T-/N-/L-/V-/Pn-status or grading on the likelihood of an R1-resection, we performed a binary logistic regression with a R0/R1 resection as endpoint.

**Results:**
A total of 3.309 PDACs were included for analyses. Mean age was 69.2 years (35 - 91) and 48.6% were female. The rate of R0 was 75.5%.
R1 was associated to a higher T-status and more likely to have positive lymph nodes (N+). Furthermore, vascular invasion (V1), perineural invasion (Pn1) or lymphatic invasion (L1) was less frequent in the R0 group. The rate of different procedures did not vary across groups.
Mean OS after the operation in the R0 group was 24.6 months (95% confidence interval [CI]: 22.5 - 26.6) and in the R1 group 17.5 months (95% CI: 15.9 - 19.2; log rank test $p < 0.01$). Mean DFS was 32.2 months (95% CI: 28.2 - 36.3) and 19.2 months (95% CI: 15.6 - 22.7; log rank test $p <0.01$) in the R0 and R1 group, respectively.
As previous studies have found the predictive value of a R1 resection margin to vary between procedure types, the cohort in the present study was divided into patients undergoing PD, DPR or TPE. While the OS and DFS in the PD group remained significantly improved for a R0 resection over a R1 resection, there was no difference for the OS in the TPE group or DPR group. DFS was significantly longer for the R0 group after TPE and DPR.

After case-control matching for T-status, N-status, grading and perineural invasion repeated Kaplan-Meier analyses found a significantly improved OS in the R0 group (29.0 months [95% CI: 24.8 - 33.1]) compared to the R1 group (17.4 months [95% CI: 15.1 - 19.7], p <0.01). DFS however was statistically but not clinically improved (19.8 months [95% CI: 16.4 - 23.1] vs. 15.6 months [95% CI: 13.0 - 18.3], R0 vs. R1 respectively, p = 0.03).
Multivariable regression analysis revealed a higher T-status (odds ratio [OR] 7.68 [95% CI: 3.55 - 16.58], p <0.01), N+ (OR 1.72 [95% CI: 1.26 - 2.34], p <0.01), V1 (1.48 [95% CI: 1.13 - 1.94], p <0.01) and Pn1 (2.30 [95% CI: 1.43 - 3.73], p <0.01) to be independent predictors of a R1 resection. The grading and L-status were not significant predictors.

**Conclusion:**
In this study we analyzed real world data from 3309 surgical PDAC resections and the prognostic value of a R0/R1 resection. We found a R1 resection to be associated with a decreased DFS and OS. After controlling for T-status, N-status, grading and perineural invasion, R1 was no longer associated to a decreased DFS but OS. These results show that a R1 resection is not just a mediator variable between the tumor size and tumor biology as previously suggested2, but an independent risk factor influencing the OS. Furthermore, we showed that the prognostic value of R0/R1 varies depending on type of surgery. Our multivariable regression model provides evidence that the grading as an expression of tumor biology does not influence the rate of a R1 resection. The tumor size i.e. the T-status however was the most impactful predictor variable for a R1 resection.
This study underlines the important of a tumor free resection margin and highlights the prognostic value using a large multicenter cohort.

1 Ferlay, J. et al. Cancer incidence and mortality patterns in Europe: Estimates for 40 countries and 25 major cancers in 2018. Eur J Cancer 103, 356-387 (2018). https://doi.org:10.1016/j.ejca.2018.07.005
2 Demir, I. E. et al. R0 Versus R1 Resection Matters after Pancreaticoduodenectomy, and Less after Distal or Total Pancreatectomy for Pancreatic Cancer. Ann Surg 268, 1058-1068 (2018). https://doi.org:10.1097/SLA.0000000000002345
3 Schlitter, A. M. & Esposito, I. Definition of microscopic tumor clearance (r0) in pancreatic cancer resections. Cancers (Basel) 2, 2001-2010 (2010). https://doi.org:10.3390/cancers2042001
4 Strobel, O. et al. Pancreatic Cancer Surgery: The New R-status Counts. Ann Surg 265, 565-573 (2017). https://doi.org:10.1097/SLA.0000000000001731
5 Gebauer, F. et al. Resection margin clearance in pancreatic cancer after implementation of the Leeds Pathology Protocol (LEEPP): clinically relevant or just academic? World J Surg 39, 493-499 (2015). https://doi.org:10.1007/s00268-014-2808-4

Abstracts – DCK 2024 – 141st Congress of the German Society of Surgery – Leipzig, April 24–26 • DOI 10.1515/iss-2024-2003
Innov Surg Sci 2024; 9 (Special Suppl 1): s18–s203

**Selection of the volume of surgical intervention in patients with complicated forms of chronic pancreatitis and high risk of developing of pancreatic cancer.**
ID: 302

Olexander Usenko[1], Anatolii Sukhodolia[2], Sergii Sukhodolia[3], Vadym Tolochyk[4]

[1]*Shalimov National Institute of Surgery and Transplantology, Kyiv, Ukraine*, [2]*Pirogov National Medical University, Vinnytsya, Ukraine, Vinnytsya, Ukraine*, [3]*Pirogov National Medical University, Vinnytsya, Ukraine*, [4]*Sana Klinikum Hof*

**Background:**

Chronic pancreatitis (CP) is a common recurrent pathology of the pancreas. The long course of the inflammatory process, accompanied by chronicity, is often attributed to the causes that lead to the development of a pathology with an extremely unfavorable prognosis - pancreatic cancer (PC). That is why the search for diagnostic criteria that would allow predicting the transition of inflammatory processes in pancreas to hyperproliferative ones and the risk of development PC.

**Materials and methods:**

41 patients were operated on in the period 2018-2022 and were included in the study. The average age was 56 years (38-79 years). In 22 of them (54%), despite a thorough preoperative examination, we were unable to clearly and convincingly differentiate CP from PC, since quite often the clinical symptoms and diagnostic picture of these diseases are common. In the remaining 19 (45%) patients, it was confirmed with all the inherent clinical signs of CP and ductal hypertension. In 34 (83%) patients (men 20 (60%), women 14 (40%)) moderate and severe exocrine insufficiency was observed, confirmed by a significant decrease in fecal elastase, manifested by indigestion, loss of body weight, weakness. We diagnosed diabetes mellitus first detected in adulthood in 14 (33.5%) of 41 patients.
Taking into account the changes in the protein profile of plasma and pancreas tissue, the total proteolytic activity, the activity of MMTs and serine proteinases in pancreas, in the experiment, as well as studying the changes in blood plasma in the preoperative period of patients from the risk group, we focused on intraoperative biopsy and diagnosis (fig.1).

**Results:**

Among the operated patients of the main group, which consisted of 22 (55%), 13 (60%) were diagnosed with diabetes mellitus and changes in MMTs, TIMP and peptide pool, which were characteristic of the malignant tissue studied by us in the experiment. Intraoperatively, after performing a punch biopsy in 3 (23%) we found acinar metaplasia, in 7 (54%) tissue changes characteristic of PanIN2-PanIN3, in 3 (23%) intraoperatively confirmed PC in the area of the head of the pancreas. Changes in the protein profile of the plasma, which we determined at the preoperative stage, clinical manifestations, as well as characteristic changes in the pancreas tissue, gave us grounds for performing extended resection interventions. Therefore, 7 (32%) Whipple operations (fig 2.), 9 (40,5%) left-sided distal resections were performed. In the remaining six (27,5%) patients with complicated CP, ductal hypertension, pain syndrome, but without suspicion of malignancy, drainage surgery was performed.

**Conclusion:**

Thus, a thorough analysis of patients, the use of as many methods as possible for differential diagnosis between CP and PC, and a comprehensive approach to the patient will allow for the most correct and effective intervention. In our study, we confirmed the effectiveness of the intraoperative biopsy of the pancreas, especially multiple, for the verification of malignant pancreas tissue, as well as the significant risk of further development of PC in complicated and clinically manifest forms of CP.
Using all the described methods of pre- and intraoperative diagnosis, we managed to optimize the selection of the volume of surgical intervention in complicated forms of CP in patients with a high risk of developing PC. The results we obtained improved the quality and radicalism of the surgical interventions performed.

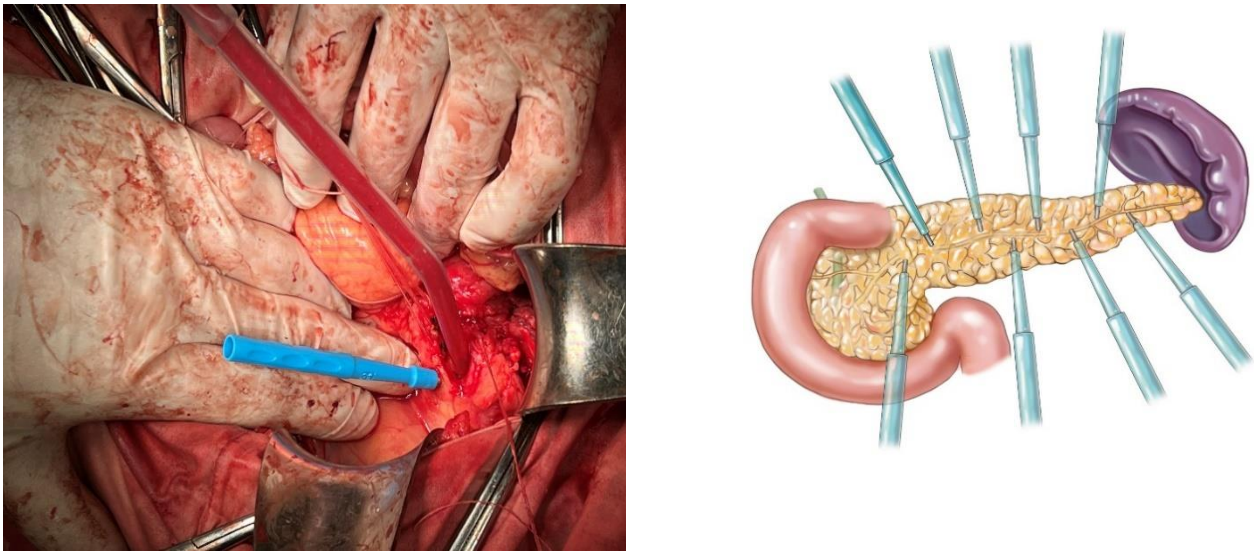

fig.1 Intraoperative biopsy.

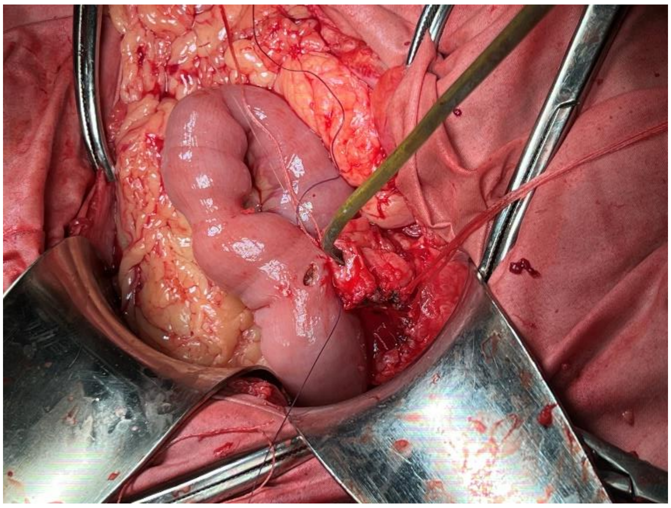

fig.2 Whipple operations.

Characteristics of patients

41 patients
34 (83%) -endo, exocrine insufficiency.
19 (45%) compliceted CP
(fibrosis, pain, ductal hypertension, -endo-exocrine insufficiency).
22 (54%) compliceted CP
(fibrosis, pain, ductal hypertension, -endo-exocrine insufficiency
however, there is a suspicion of malignancy and PC.
17 (91,5%) CP.
2 (8,5%) compliceted CP - bleeding (ductal-venous fistula) - left side resection.
9 (40%) after examinations without signs of malignancy.
13 (60%)
- DM (first discovered)
- TIMP ↑, MMP ↑
- punch biopsy (3- PC, 3 - acinar metaplasia, 7 - PanIN).
8 (45%) Frey procedure.
9 (55%) modified Puestow procedure.
6 (67%) Frey and Puestow procedure.
3 (33%) left side resection of the pancreas.
6 (46%)- Whipple procedure.
7 (%) left side resection of the pancreas.

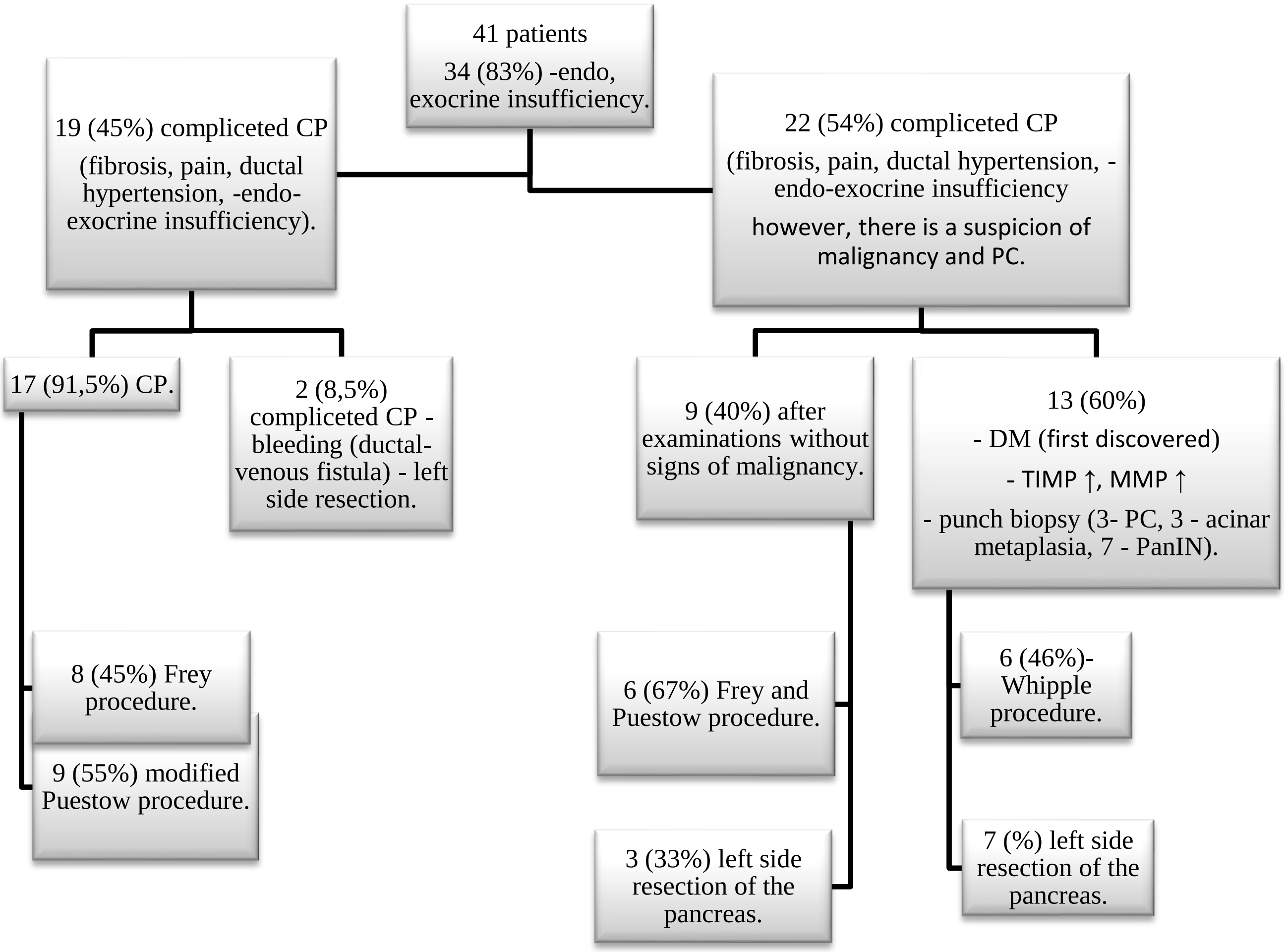

**Surgical Solutions for Senior Livers: Unraveling Drug Metabolism Changes in Elderly Patients**
ID: 351

Dominik Schröter[1], Alexander Berndt[2], Eva Kindler[3], Laura Bütow[3], Johannes Dr. Roth[4], Uta Dahmen[1], Hans-Michael Tautenhahn[5]

[1]*Jena University Hospital, Clinic for Gerneral-, Viszeral-, and Vascular surgery, Jena, Deutschland*, [2]*Jena University Hospital, Institut for pathology, Jena, Deutschland*, [3]*Universitätsklinikum Jena, Clinic for Gerneral-, Viszeral-, and Vascular surgery, Jena, Deutschland*, [4]*Jena University Hospital, Clinic for emergency medicine, Jena, Deutschland*, [5]*Leipzig University Hospital, Clinic for Gerneral-, Viszeral-, and Vascular surgery, Leipzig, Deutschland*

**Background:**
Due to the demographic change, the number of elderly patients is rising, resulting in an increase in patients with multiple comorbidities.[1] According to surveys by "*Die gesetzlichen Krankenkassen*", every third patient over 75 years of age takes more than 8 drugs per day.[2] This leads to a higher risk of interactions causing side effects. This risk is even more pronounced when elderly patients with comorbidities are subjected to liver surgery substantially reducing the functional liver mass.[3]
One key function of the liver is the metabolism of xenobiotics and drugs. Excretion is done in three steps: oxidation, conjugation, and excretion. Oxidation is catalysed by CYP enzyme system.[4] Therefore, it is of clinical importance to understand the impact of ageing and fibrosis on drug metabolism. In this study we show the impact on CYP expression and activity.

**Materials and methods:**
20 young (<45 years) and 20 old (>65 years) patients who underwent major liver surgery in the years 2019 – 2023 were included into this study. Based on the histological assessment, these two groups were further divided in those with 20 and without 20 additional morphological impairment such as different grades of fibrosis (F1-F4) to identify the impact of ageing itself.[5]
Liver tissues sections were subjected to multiplex immunofluorescence staining (mIF) using an antibody panel against 6 markers: CYP 1A2 and CYP3A4 as 2 important markers of drug metabolism, E-cadherin as periportal marker [6], CD31 to visualize vessels and sinusoids, alpha-smooth muscle antigen (ASMA) to detect soft tissue activation in fibrosis, CPS1 (carbamoyl phosphate synthetase I) as a periportal marker of acute liver injury.[7] mIF was performed using the Opal 6-Plex Manual Detection Kit (AKOYA Biosciences). Visualization was done using the Vectra Polaris™ Automated Quantitative Pathology Imaging System (AKOYA Biosciences).
Furthermore, CYP1A2, 3A4 and 2C19 activity was assessed in the same patients *in-vitro using* a fluorometric detection. In order to determine the absolute and relative CYP activity, the total volume of liver tissue was determined by subtracting tumor volume from the total liver volume. Clinical data of the patients including laboratory data, medication, MELD score, CP score, APRI/ALBI score and the LiMAx® assay were collected to be correlated with the liver function parameters.

**Results:**
Up to now, 20 tissue samples from the planned 40 patients, five per group were examined.
Despite the high interindividual variability in CYP expression, there was a decreased expression of CYP 3A4 in old age. The pericentral expression pattern showed no changes. In cirrhotic patients, there was an increased periportal expression resulting from an alteration in zonation. However, total CYP1A2 and 3A4 expression was decreased.
ASMA expression was increased in the periportal and pericentral area in the cirrhotic patients. Here no influence of ageing was detected. CD31 was increased in the cirrhotic liver reflecting an increase of blood vessels and CPS1 was decreased in cirrhotic tissue.

**Conclusion:**

Regardless of the small samples size, substantial differences in the spatial distribution of CYP1A2 and CYP3A4 between normal and cirrhotic liver were detected. These results support the hypothesis, that activity should be affected in a similar way. However, this needs to be confirmed by the ongoing experiments. In a perspective this could help to avoid over- or underdosing of drugs in those patients at risk.

**References:**

1. Younossi ZM, Blissett D, Blissett R, Henry L, Stepanova M, Younossi Y, Racila A, Hunt S, Beckerman R. The economic and clinical burden of nonalcoholic fatty liver disease in the United States and Europe. Hepatology 2016;64:1577-1586.
2. Arzneiverordnungs-Report 2018 : aktuelle Daten, Kosten, Trends und Kommentare. In: Schwabe U, Paffrath D, Ludwig W-D, Klauber J, eds. SpringerLink. Berlin ; Heidelberg: Springer, 2018.
3. Wuensch T, Heucke N, Wizenty J, Quint J, Sinn B, Arsenic R, Jara M, Kaffarnik M, Pratschke J, Stockmann M. Hepatic CYP1A2 activity in liver tumors and the implications for preoperative volume-function analysis. Am J Physiol Gastrointest Liver Physiol 2019;316:G608-g614.
4. Seifert R. Basic Knowledge of PharmacologySpringer eBooks. Cham: Springer, 2019.
5. Batts KP, Ludwig J. Chronic hepatitis. An update on terminology and reporting. Am J Surg Pathol 1995;19:1409-17.
6. Cunningham RP, Porat-Shliom N. Liver Zonation - Revisiting Old Questions With New Technologies. Front Physiol 2021;12:732929.
7. Kwan R, Chen L, Park M-J, Su Z, Weerasinghe SVW, Lee WM, Durkalski-Mauldin VL, Fontana RJ, Omary MB. The Role of Carbamoyl Phosphate Synthetase 1 as a Prognostic Biomarker in Patients With Acetaminophen-induced Acute Liver Failure. Clinical Gastroenterology and Hepatology 2023.

**Transfusions of packed red blood cells in surgery for liver cancer: predictor of impaired overall survival but not recurrence-free survival**
ID: 409

Esther Giehl-Brown[1], Eileen Geipel[1], Steffen Löck[2], Karolin Dehlke[1], Johannes Schweipert[1], Jürgen Weitz[1], Carina Riediger[1]

[1]*Viszeral-, Thorax- und Gefäßchirurgie (VTG), Universitätsklinikum Carl Gustav Carus, Dresden, Deutschland*, [2]*OncoRay, Dresden, Deutschland*

**Background:**
Liver surgery remains a cornerstone of potentially curative multimodal treatments for primary malignancies of the liver and hepatic metastases. Improving perioperative safety is a prerequisite in this context. Perioperative blood transfusions negatively influence postoperative recovery. This study aims to identify risk factors for perioperative packed red blood cell (PRBC) transfusion and to elucidate its effect on postoperative outcomes.

**Materials and methods:**
This is an observational study of a prospective data collection. A monocentric, retrospective analysis of 1118 hepatectomies at the University Hospital Carl Gustav Carus between 2013 and 2021 was conducted to compare postoperative short- and long-term outcomes in patients undergoing curative intended liver resection of hepatic primary or secondary malignancies. Outcomes were compared between 356 (31.8%) patients receiving PRBC transfusions during surgery or within the following 7 days or not (762 patients, 68.2%).

**Results:**
Preoperative anemia could be observed in 45.0% of the whole cohort; 65.7% in the PRBC group and 35.3% in the non-transfused group. Postoperative complications were significantly more common in the PRBC transfusion group in association with prolonged lengths of hospital stay and increased 30-day mortality. After adjustment for possible confounders, pre-existing kidney failure, preoperative hemoglobin and albumin outside of the reference range, intraoperative plasma transfusions and overall surgery time were recognized as negative predictors for perioperative PRBC transfusions. Transfusion of PRBCs increased the risk of death by approximately 38.8% (HR 1.388, 95%CI 1.027-1.876, p=0.033), while no influence on recurrence-free survival was observed.

**Conclusion:**
PRBC transfusions are associated with worse postoperative morbidity and mortality after curative-intended surgery for liver cancers and represent an independent poor prognostic indicator for overall survival but not recurrence-free survival.

**Does patients' age influence 5-year survival in intrahepatic cholangiocarcinoma?**
ID: 419

Astrid Bauschke[1], Annelore Altendorf-Hofmann[1], Aladdin Ali-Deeb[1], Felix Dondorf[1], Falk Rauchfuss[1], Oliver Rohland[1], Utz Settmacher[1]

[1]*Klinik für Allgemein-, Viszeral- und Gefäßchirurgie, Universität Jena, Jena, Deutschland*

**Background:**
Most solid malignant tumors in the liver are hepatocellular carcinoma (HCC). Intrahepatic cholangiocarcinoma (iCC) is the second most common primary liver tumor. The aim of this study is to find prognostic factors for intrahepatic cholangiocarcinoma considering patient's age, type of

**Materials and methods:**
We reviewed the records of 403 consecutive patients with iCC introduced to our department from 1995 to 2021. Cases with mixed HCC and iCC and the cases without histological proof of iCC were not included in statistical analyses.
All patients were followed until death or at least for 5 years after diagnosis. Treatment decisions for all patients were made in an interdisciplinary conference. Follow-up time was 92 months.

**Results:**
We included 403 tumors diagnosed as diagnosed as iCC (8160/3) by the pathologist. Patients with iCC were female in 43%, 23% of iCC tumors had distant metastases at diagnosis. Systemic therapy only or best supportive care was given to more than one third of the iCC patients (37%).
Between 1995 and 2009 we saw 141 patients, between 2010 and 2021 we saw 262 patients. Dependent on age we divided patients in three groups < 50 years (41 patients), 50-69 years (237 patients) and ≥70 years (125 patients).

| | | All n | Age ≤ 49 | Age 50-69 | Age **≥70** | p |
|---|---|---|---|---|---|---|
| Year of diagnosis | 1995-2009 | 141 | 11% | 67% | 22% | **0,015** |
| | 2010-2021 | 262 | 10% | 54% | 36% | |
| Number of lesions | Solitary | 207 | 46% | 51% | 56% | **0,471** |
| | Multiple | 192 | 54% | 49% | 44% | |
| Therapy | Partial hepatectomy / LTX | 148 | 32% | 39% | 34% | **0,611** |
| | Local therapy | 31 | 12% | 7% | 7% | |
| | No local therapy | 224 | 56% | 54% | 59% | |
| M category | M0 | 310 | 71% | 76% | 81% | **0,355** |
| | M1 | 93 | 29% | 24% | 19% | |

The number of patients **≥70 years of age increased statistically significant during the study time from 22% to 36%. Among all 403 patients** number of lesions, type of therapy and number of patients with distant metastases were not statistically significant different between age groups (table).
**148 patients had a potential curative tumor resection (R0 / R1). Among these patients younger than 50 years had statistically more tumors ≤ 5cm and showed statistically significant more often lymph node metastases. The percentage of R0 resections, grade 3, vessel invasion, UICC stage, perioperative complications and perioperative mortality did not differ between the three age groups.**

44 of 138 resected patients with with iCC have survived for more than 5 years. The crude 5 year survival rate was 28% for all patients and did not differ statistically significant for the three age groups (10%, 27% and 34%, respectively). There was only a trend to better survival for the rest of the patients in comparison to the group of younger patients (p=0.082).

In a multivariate Analysis including age, tumor grade, diameter, number of tumors, pT category, pN category, UICC-stage, and year of diagnosis only pN category and number of tumors proved to be independent statistically significant predictors of 5-year survival after diagnosis, especially age was

**Conclusion:**
As expected, the proportion of patients with more than 70 years of age is rising. We could demonstrate that older age does not impair short term and long-term results. The whole of younger patients had the lowest 5 year survival rate of the three age groups. Multivariate analysis suggests that the low survival rates of the younger patients are due to the higher number of intrahepatic lesions and the higher frequency of lymph node metastases.

### Preoperative predictors for non-resectability in perihilar cholangiocarcinoma

ID: 472

Carlos Otto[1 2], Anna Mantas[1 2], Lara Heij[1 2], Daniel Heise[1 2], Maxime Dewulf[3], Sven Lang[1 2], Tom Ulmer[1 2], Edgar Dahl[4], Philipp Bruners[5], Ulf Neumann[1 2], Jan Bednarsch[1 2]

[1]*Department of Surgery and Transplantation, University Hospital RWTH Aachen, Aachen, Germany*, [2]*Department of Surgery and Transplantation, University Hospital Essen, Essen, Germany*, [3]*Department of Surgery, Maastricht University Medical Center (MUMC), Maastricht, Netherlands*, [4]*Institute of Pathology, University Hospital RWTH Aachen, Aachen, Germany*, [5]*Department of Radiology, University Hospital RWTH Aachen, Aachen, Germany*

**Background:**
Despite improvement of numerous preoperative imaging modalities in last decades, explorative laparotomy without subsequent curative-intent liver resection remains a major clinical problem in the treatment of perihilar cholangiocarcinoma (pCCA) and results in impaired quality of life, delayed induction of systemic therapy and increased costs. Thus, we aimed to identify preoperative risk factors for non-resectability in pCCA patients.

**Materials and methods:**
In our monocentric cohort, patients diagnosed with pCCA who underwent surgical exploration between 2010 and 2022 were eligible for analysis. Separate binary logistic regression analyses were used to determine risk factors for non-resectablity during explorative laparotomy due to technical (tumor extent, vascular infiltration), oncological (peritoneal carcinomatosis, distant nodal or liver metastases) or liver function reasons. To assess the prognostic impact of non-resectability, survival analyses were conducted.

**Results:**
This monocentric study cohort includes 318 patients with 209 (65.7%) being resected and 109 (34.3%) being surgically explored (explorative laparotomy: 87 (27.4%), laparoscopic exploration: 22 (6.9%)). Risk factors for non-resectability were advanced age (HR=3.76, p=0.003), portal vein embolization (PVE, HR=5.73, p=0.007) and arterial infiltration >180° (HR=8.05 p<0.001) for technical non-resectability and PVE (HR=4.67, p=0.018), arterial infiltration >180° (HR=3.24, p=0.015) and elevated CA 19-9 (HR=3.2, p=0.009) for oncological non-resectablility. In survival analyses, median cancer specific survival was 32 months (95%CI:20 - 44 months) after curative liver resection and 6 months (95%CI:4 - 8 months) in patients after being surgically explored but not resected.

**Conclusion:**
Advanced age, PVE, arterial infiltration and elevated CA19-9 are major risk factors for non-resectability in pCCA. Our data strongly advocates diagnostic laparoscopy in pCCA, especially in high-risk situations, to reduce the number of explorative laparotomies without subsequent liver resection. Further, our results underline the role of surgery to achieve long-term survival in pCCA.

Abstracts – DCK 2024 – 141st Congress of the German Society of Surgery – Leipzig, April 24–26 • DOI 10.1515/iss-2024-2003
Innov Surg Sci 2024; 9 (Special Suppl 1): s18–s203

**Prognostic value of resection margin and lymph node status in perihilar cholangiocarcinoma**
ID: 487

Faruk Koca[1], Hanan El Youzouri[1], Jan Heil[1], Michael Heise[1], Svenja Sliwinski[1], Wolf Otto Bechstein[1], Andreas Schnitzbauer[1], Ekaterina Petrova[1]

[1]*Goethe-Universität Frankfurt, Universitätsklinikum, Klinik für Allgemein-, Viszeral-, Transplantations- und Thoraxchirurgie, Frankfurt am Main, Deutschland*

**Background:**
The impact of resection margin and lymph node status on survival in patients undergoing resection for perihilar cholangiocarcinoma remains controversial. Especially the prognostic value of negative resection margin in the case of regional lymph node metastases has been called into question. The aim of this study is to further investigate the influence of nodal status and resection margin status on the long-term survival after resection for perihilar cholangiocarcinoma.

**Materials and methods:**
Retrospective analysis of patients resected for perihilar cholangiocarcinoma at the University Hospital Frankfurt between 1999 and 2022. The patients were categorized in four groups according to resection status and nodal status: R0N0, R0N+, R+N0, R+N+. Median survival and follow-up times were estimated retrospectively using the Kaplan-Meier and the reversed Kaplan-Meier methods. Univariable Cox regression was used to analyse the prognostic value of the four categories and other common clinicopathological parameters. Parameters with a p-value <0.1 in the univariable Cox regression were included in a multivariable Cox regression model.

**Results:**
A total of 100 patients with long-term survival were included. Of those, 65 (65%) patients had no lymph node metastases on pathological evaluation (pN0) and 35 (35%) patients had regional lymph node metastases (pN+). Seventy-one patients had negative resection margin (pR0), while 29 (29%) patients had positive resection margin (pR+). The estimated median follow-up was 78.6 [95% confidence interval (CI) 57.8-112] months. The estimated median overall survival after resection was 27.1 (95% CI 22.8 - 38.5) months. In the univariable analysis, negative resection margin [pR0 vs. pR+, 31.8 vs. 18.4 months, hazard ratio (HR) 1.87, 95% CI 1.1-3.17, p=0.02] and lower grade (G1/2 vs G3, 37.2 vs. 18.4 months, HR 2.22, 95%CI 1.3-3.81, p=0.004) were the only significant positive prognostic factors, while the difference between pN0 and pN+ was not statistically significant (33.4 vs. 24.8 months, HR 1.62, 95% CI 0.98-2.7, p=0.062). [Figure 1, A.-C.] Median survival in the groups R0N0, R0N+, R+N0 and R+N+ groups was 40.1, 29.9, 18.4 and 18.9 months, respectively (p=0.03). [Figure 1, D.] In the multivariable analysis, after adjusting for grade, nodal status, resection margin status and lymphovascular invasion (pL), only grade and resection margin had prognostic significance. [Figure 2]

**Conclusion:**
Patients with negative resection margin after resection for pCCA have a better prognosis, regardless of the presence of lymph node metastases, so R0 resection should be aimed for.

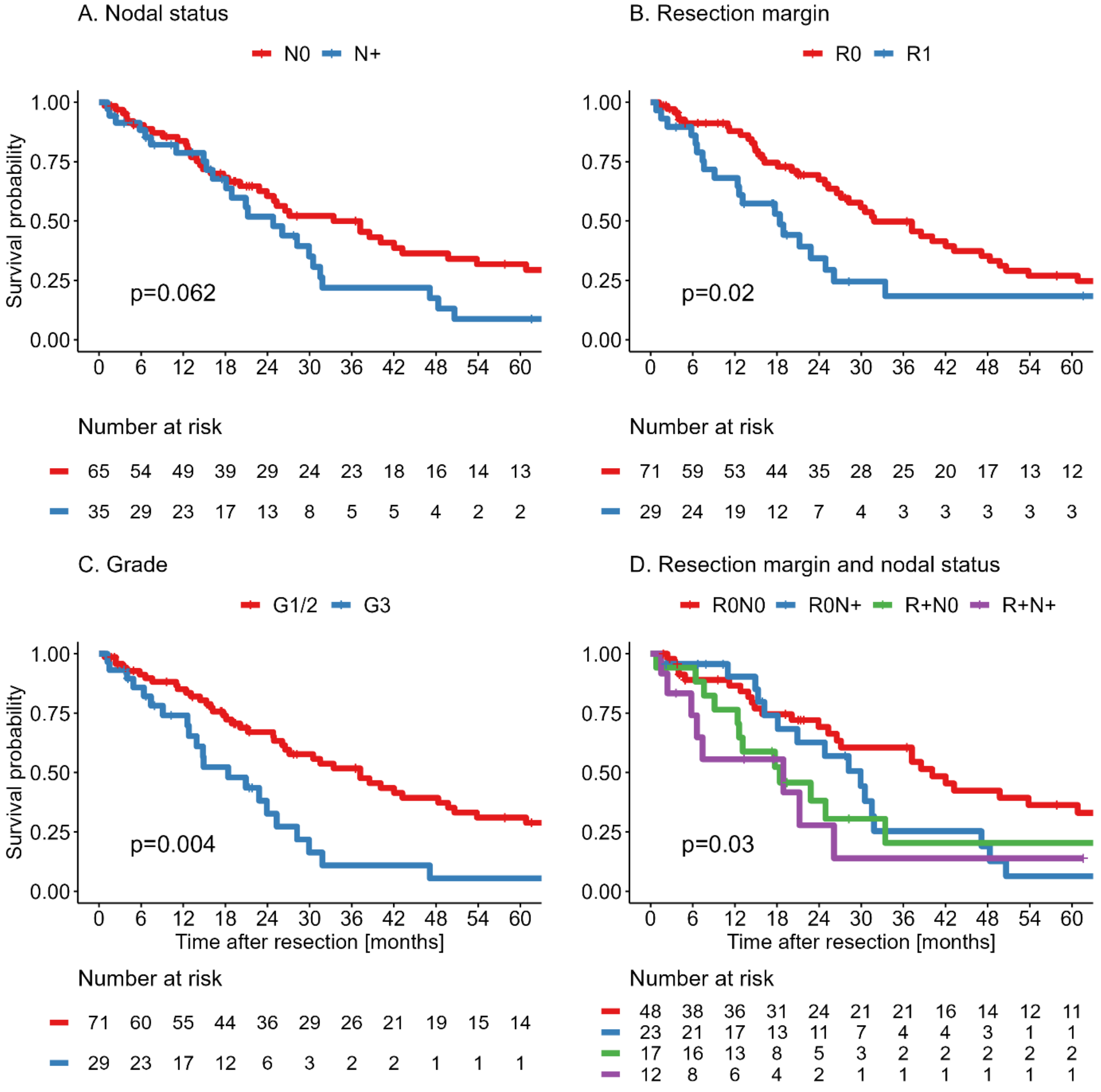

*Figure 1: Kaplan-Meier survival curves*

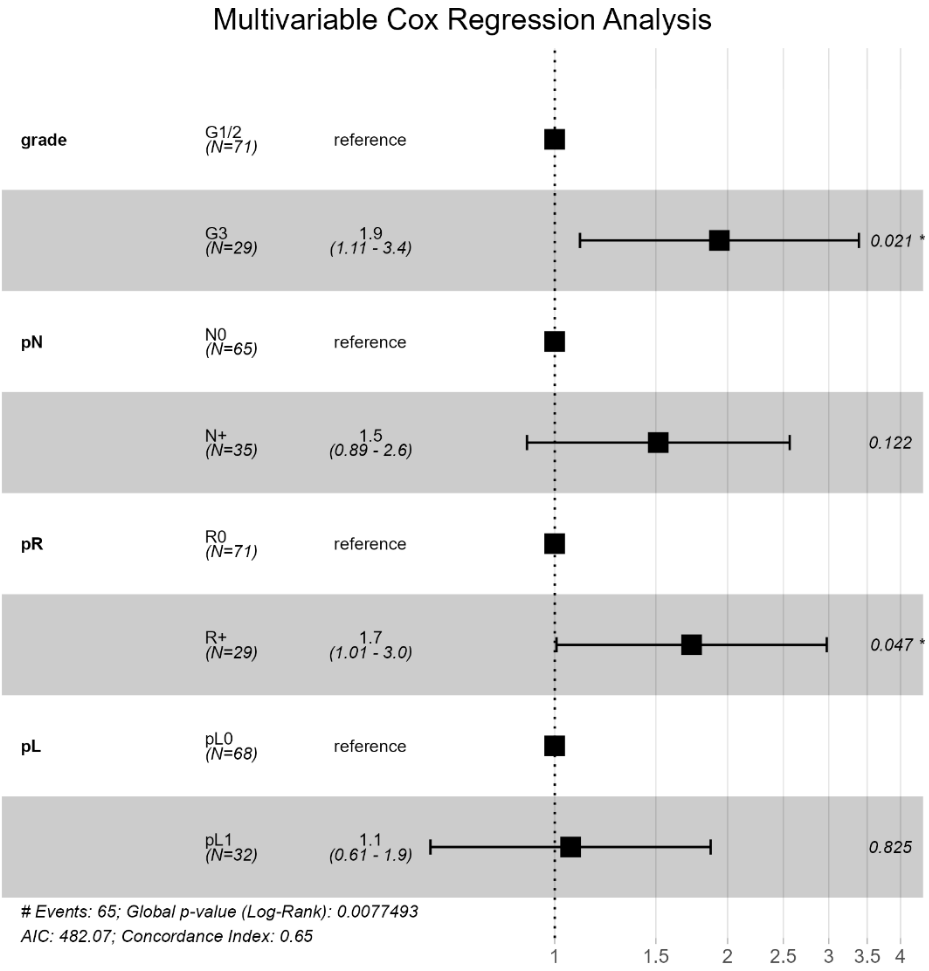

*Figure 2: Forrest plot of the multivariable survival analysis*

**Should Octogenarians Undergo Resection in PDAC? A Population-based Analysis on Effect of Age on Survival After Resection of PDAC from the German Clinical Cancer Registry Group.**
ID: 503

Thaer Abdalla[1], Louisa Bolm[1], Kim Honselmann[1], Sylke Ruth Zeissig[2 3], Kees Kleihues-van Tol[3], Monika Klinkhammer-Schalke[3], Tobias Keck[1], Richard Hummel[4], Ulrich Friedrich Wellner[1]

[1]*University Medical Center Schleswig Holstein- Campus Lübeck, Department of Surgery, Lübeck, Deutschland*, [2]*University of Würzburg, Institute of Clinical Epidemiology and Biometry (ICE-B), Deutschland*, [3]*German Cancer Registry Group of the Society of German Tumor Centers - Network for Care, Quality and Research in Oncology (ADT), Berlin, Deutschland*, [4]*University Medical Center Greiswald,, Department of Surgery, Greifswald, Deutschland*

**Background:**
Pancreatic ductal adenocarcinoma (PDAC) is frequently found in the geriatric population. Therefore, the decision to operate on the elderly for whom treatment morbidities may not be tolerable, is frequent. According to the current S3-German guidelines, age is not a limitation for pancreatic resection in the case of pancreatic cancer.

**Materials and methods:**
This population-based analysis was derived from 16 German cancer registries from 2000 to 2021. Patients were grouped by age (<65, 65-79, and ≥80 years) and categorized by treatment regimen. Postoperative outcomes and survival were analyzed.

**Results:**
A total of 28253 patients were included in this analysis. The median age was 69 years (IQR ± 9). Males were more common than females 54%. The median age increased from 64 years in 2000 to 75 in 2021. At diagnosis, 9484 (34%) were <65y, 15552 (55%) were 65-80y, and 3217 (11%) were ≥ 80 y. Patients ≥ 80y were less likely to receive curative treatment compared to patients aged <65y or 65-80y (42% vs. 59 vs. 55%). In the case of curative therapy, surgery alone was the most common strategy in patients ≥ 80y. Although there was no difference regarding tumor size, lymph node metastasis, distant metastasis, or tumor grading (p=n.s, respectively), only 27% received postoperative chemotherapy compared to 50% and 52% in patients <65y or 65-80y respectively. Operation followed by chemotherapy was associated with improved OS compared to surgery alone in patients ≥ 80y (median= 15 vs. 8 months, p<0.001). The 1-year survival was 76% for patients undergoing surgery followed by chemotherapy, 48% after pancreatic resection, and 30% in the case of palliative chemotherapy alone.
The 30- and 90-day postoperative mortality was 11% and 23% in patients >= 80 compared to 4% and 8% in patients <65 and 7% and 13% in patients aged 65-80. In patients ≥ 85 years, the 30- and 90 days after tumor resection was as high as 12% and 29%.

**Conclusion:**
Pancreatic resection in very old patients (>80) is associated with high mortality compared to compared to younger patients. Nevertheless, patients surviving the operation will have a survival advantage compared to patients undergoing palliative therapy alone. Therefore, patient counseling and selection for surgical treatment are crucial in octogenarians.

**Postoperative prophylactic antibiotic therapy following pancreaticoduodenectomy in bile duct-stented patients reduced postoperative major complications**
ID: 517

Marcella Steffani[1], Carsten Jäger[1], Norbert Hueser[1], Helmut Friess[1], Daniel Hartmann[1], Ihsan Ekin Demir[1], Florian Scheufele[1]

[1]*Klinikum rechts der Isar, Technical University of Munich, School of Medicine, Department of Surgery, München, Deutschland*

**Background:**
Pancreaticoduodenectomy (PD) can entail a high complication rate, especially in patients who underwent preoperative bile duct drainage through bile duct stenting (BDS). PD-BDS patients frequently receive prophylactic antibiotic therapy in the postoperative period. However, the exact value and the benefit of prophylactic antibiotic therapy in PD-BDS patients remains under-investigated and thus unclear.

**Materials and methods:**
We conducted a retrospective single-center study of PD-BDS patients between January 2007 and December 2022. Demographic, clinical, laboratory and pathology data of 370 patients were collected, and intraoperatively swab cultures of the bile were obtained from all patients upon transection of the common bile duct. The groups to be investigated were formed on the basis of postoperative antibiotic prophylaxis. Postoperative complications and antibiotic resistance analysis were recorded.

**Results:**
Postoperative antibiotic prophylaxis in stented patients after PD significantly reduced major complications (OR: 0.547; p=0.02). Antibiotic prophylaxis reduced the risk of readmission to intensive care unit (ICU) (OR: 0.457; p=0.041), re-operation (OR: 0.477; p=0.045) and pneumonia (OR: 0.275; p=0.050). Patients with Enterococcus faecalis (OR:1.699; p=0.048), Enterococcus faecium (OR: 1.808; p=0.050) or Citrobacter (OR: 2.211; p=0.029) in their bile had a higher probability of developing wound infections. Appropriate antibiotic prophylaxis according to the bile duct swab significantly reduced the risk of wound infection (OR: 2.239; p=0.015).

**Conclusion:**
Postoperative antibiotic prophylaxis in bile duct-stented patients after PD significantly reduced major complications such as readmission to ICU and re-operation. Targeted antibiotic treatment according to the biliary swabs reduced the incidence of wound infections.

**The use of a Surgical Procedure Manager in open liver surgery - An open label RCT**
ID: 601

Fabian Haak[1, 2], Savas Soysal[3], Philip Müller[2], Otto Kollmar[2], Marco von Strauss[2]

[1]*Universitätsklinikum Leipzig, Leipzig, Deutschland*, [2]*Clarunis Universitäres Bauchzentrum Basel Standort Claraspital, Basel, Schweiz*, [3]*Universität Basel, Basel, Schweiz*

**Background:**
Accurate perioperative processes have an enormous impact on overall quality and safety of surgery.1,2 Communication between team members has a strong influence on perioperative processes and compared to other high risk professions surgeons often do not emphasize the importance of communication within the involved surgical team.3 However, sharing information about certain steps during an intervention is of great relevance regarding a well-structured procedure.4 A possible solution to this is the usage of a surgical procedure manager (SPM). This is a software tool designed to track various steps involved in a surgical intervention, aiding healthcare professionals in management and execution of procedures. The objective of this study was to investigate the capability of a SPM to optimize the process of open liver resections.

**Materials and methods:**
This study is designed as a single center, randomized controlled trial. In a period of November 2022 and August 2023 open liver resections using a SPM were collected and compared to a control group of patients who were operated without a SPM. Our primary endpoint was the difference between actual operation time (OT) in minutes and planned OT in minutes, divided by the amount of planned operation hours (MINOPTI).

**Results:**
36 consecutive patients were included in the study. There were no differences in patient or procedure characteristics between the two groups. The control group consisted of 3 procedures with low complexity, 9 procedures with medium complexity and 6 procedures with high complexity. The SPM group included 7 procedures with low complexity, 7 procedures with medium complexity and 3 procedures with high complexity ($p = 0.172$). MINOPTI in SPM group was -14.33 min (IQR 24.33) and -11.27 min (IQR 13.37) in the control group. ($p = 0.643$) There was no difference in perioperative outcomes between the two groups. The intervention did not cause more stress to the involved team. This was shown by a median Raw NASA Task Load Index of the lead surgeon of 37.22 (IQR 31.67) in the control group and 42.82 (IQR 30) in the SPI group ($p = 0.398$). The SPM displayed 6 consecutive phases of the operation. Procedure complexity dependent phases (Phase 3 = Preparation Resection, phase 4 = Resection, phase 5 = Completion) showed high IQR: Phase 3 IQR = 51.4 min, phase 4 IQR = 32.1 min and phase 5 IQR = 59 min. This improved when stratifying for procedure complexity. Independent of operation complexity the final phase 6 with a median OT of 22 min showed a low IQR of 7.9 min.

**Conclusion:**
Our data show no significant reduction of MINOPTI. Further studies are needed to investigate if stratification by procedure difficulty can lead to significant improvement of MINOPTI.
However, the low IQR demonstrates that the timepoint of the end of the operation can be accurately conveyed by the SPM. This can be of high value for anesthetists who prepare the patient for the end of anesthesia or further team members involved in the immediate aftercare of the patient.
Furthermore, this study verifies, that the usage of a SPM during a surgical intervention does not increase stress of the involved team nor lead to negative perioperative outcomes. It is therefore safe to use.
We believe a SPM should be considered as a highly beneficial safe method to improve communication between team members in the operating room.

1. Haugen AS, Søfteland E, Almeland SK, et al. Effect of the World Health Organization checklist on patient outcomes: a stepped wedge cluster randomized controlled trial. Ann Surg. 2015;261(5):821-828. doi:10.1097/SLA.0000000000000716
2. von Strauss Und Torney M, Aghlmandi S, Zeindler J, et al. High-resolution standardization reduces delay due to workflow disruptions in laparoscopic cholecystectomy. Surg Endosc. 2018;32(12):4763-4771. doi:10.1007/s00464-018-6224-y
3. Haak F, Poletti T, Grossman N, et al. Interprofessional differences in preoperative planning—the individualist surgeon. Eur Surg. 2022;54(5):267-272. doi:10.1007/s10353-022-00761-z
4. Neumuth T. Surgical process modeling. Innov Surg Sci. 2017;2(3):123-137. doi:10.1515/iss-2017-0005

Abstracts – DCK 2024 – 141st Congress of the German Society of Surgery – Leipzig, April 24–26 • DOI 10.1515/iss-2024-2003
Innov Surg Sci 2024; 9 (Special Suppl 1): s18–s203

**KRAS & GNAS mutations in cfDNA and circulating epithelial cells in patients with intraductal papillary mucinous neoplasms – an observational pilot-study regarding the risk of progression to pancreatic cancer**
ID: 607

Christine Nitschke[1], Marie Tölle[1], Mara Goetz[1], Jolanthe Kropidlowski[2], Jakob R. Izbicki[1], Felix Nickel[1], Thilo Hackert[1], Klaus Pantel[2], Faik G. Uzunoglu[1], Harriet Wikman[2]

[1]*Klinik für Allgemein-, Viszeral- und Thoraxchirurgie, Universitätsklinikum Hamburg-Eppendorf, Hamburg, Deutschland*, [2]*Institut für Tumorbiologie, Universitätsklinikum Hamburg-Eppendorf, Hamburg, Deutschland*

**Background:**
Intraductal papillary mucinous neoplasms (IPMN) belong to the pancreatic cystic benign neoplasms, of which some may progress into an invasive ductal pancreatic adenocarcinoma (PDAC). For main duct IPMN, malignant progression into PDAC is about 40-92% in lifetime.
Our study evaluates the suitability of *KRAS* and *GNAS* mutated cell-free DNA (cfDNA) as a biomarker for risk stratification in IPMN - by correlating their prevalence in blood samples with the current risk classification of IPMN.

**Materials and methods:**
We collected peripheral EDTA blood samples from patients who underwent surgery to remove main duct or “worrisome feature” IPMN (n=25) or patients who are under clinical surveillance for branch duct IPMN (n=23). All blood samples were processed for cfDNA isolation, before being screened for *KRAS* and *GNAS* mutations via digital-droplet PCR.

**Results:**
Overall, *KRAS* mutations were found in 10% and *GNAS* mutations in 21% of cfDNA. Among the operative IPMN cohort, the detection of *KRAS* mutations was 16% and of *GNAS* mutations 36%. In the conservatively managed IPMN cohort, *KRAS* mutations were observed in 4% and *GNAS* mutations in 4%. Especially *GNAS* mutations in cfDNA were significantly more often observed in operated IPMN patients compared to conservatively managed IPMN (p=0.024).

**Conclusion:**
In summary, especially the prevalence of *GNAS* mutations is higher in the group of IPMN patients who underwent surgery – compared to conservatively managed IPMNs -, which reflects the correlation mutations with potential for increased malignant progression. The results of this preliminary pilot-study justify future studies focusing on *GNAS* and additional liquid biopsy markers.

Abstracts – DCK 2024 – 141st Congress of the German Society of Surgery – Leipzig, April 24–26 • DOI 10.1515/iss-2024-2003
Innov Surg Sci 2024; 9 (Special Suppl 1): s18–s203

**Body composition is associated with postoperative complications in perihilar cholangiocarcinoma**
ID: 707

Anna Mantas[1, 2], Guanwu Wang[2], Lara R. Heij[2], Tarick M. Al-Masri[2, 3], Dong Liu[2], Daniel Heise[1, 2], Sophia M. Schmitz[1 2], Stephen A. W. Olde Damink[4], Tom Luedde[5], Sven A. Lang[1 2], Tom F. Ulmer[1, 2], Ulf P. Neumann[1, 2, 4], Jan Bednarsch[1, 2]

[1]*Department of Surgery and Transplantation, University Hospital Essen, Essen, Deutschland*, [2]*Department of Surgery and Transplantation, University Hospital RWTH Aachen, Aachen, Deutschland*, [3]*University of Applied Science Aachen, Aachen, Deutschland*, [4]*Department of Surgery, Maastricht University Medical Centre (MUMC), Maastricht, Netherlands*, [5]*Department of Gastroenterology, Hepatology and Infectious Diseases, Heinrich Heine University Duesseldorf, Duesseldorf, Deutschland*

**Background:**
Perihilar cholangiocarcinoma (pCCA) is a malignant tumor of the hepatobiliary system which is still associated with a challenging prognosis. Postoperative complications play a crucial role in determining the overall prognosis of patients with pCCA. Changes in body composition (BC) have been shown to impact the prognosis of various types of tumors. Therefore, our study aimed to investigate the correlation between BC, postoperative complications and oncological outcome in patients with pCCA.

**Materials and methods:**
All patients with pCCA who underwent curative-intent surgery for pCCA between 2010 and 2022 in a single hepatobiliary center were included in this analysis. BC was assessed using preoperative computed tomography and analyzed with the assistance of a 3D Slicer software. Univariate and multivariate binary logistic regression analyses were conducted to examine the relationship between BC and clinical characteristics including various measurements of postoperative complications (overall complications, infectious complications, liver failure, hemorrhage, bile leakge) and Cox regressions and Kaplan-Meier analysis to evaluate oncological risk factors in the study cohort.

**Results:**
BC was frequently altered in patients undergoing curative-intent liver resection for pCCA (n=204) with 52.5% of the patients showing obesity, 55.9% sarcopenia, 21.6% sarcopenic obesity, 48.5% myosteatosis and 69.1% visceral obesity. In multivariate analysis, severe postoperative complications (Clavien-Dindo ≥3b) were associated with body mass index (BMI) (Odds ratio (OR)=2.001, p=0.024), sarcopenia (OR=2.145, p=0.034), and myosteatosis (OR=2.097, p=0.017) as independent predictors. Furthermore, sarcopenia was associated with reduced overall survival (OS) in pCCA patients (sarcopenia vs. no-sarcopenia, 21 months vs 32 months, p=0.048 log rank).

**Conclusion:**
Altered BC is major problem in patients with pCCA. More than half of show notable changes in BC due to cancer-cachexia and chronic infections. In our large cohort, we demonstrated a notable association of altered BC with severe postoperative complications in patients who underwent liver resection and show a tendency of impaired overall survival. Preoperative assessment of BC and interventions to improve BC might therefore be key to improve outcome in pCCA patients undergoing surgical therapy.

Abstracts – DCK 2024 – 141st Congress of the German Society of Surgery – Leipzig, April 24–26 • DOI 10.1515/iss-2024-2003
Innov Surg Sci 2024; 9 (Special Suppl 1): s18–s203

**Achieving clinical applicability for circulating tumor DNA (ctDNA) as biomarker for neoadjuvant chemotherapy vs. surgery in localized pancreatic cancer**
ID: 731

Patrick Kirchweger[1, 2, 3, 4], Bernhard Doleschal[5], Alexander Kupferthaler[3, 6], Simon Schwendinger[7], Anna Ennemoser[7], Emina Jukic[7], Jonathan Burghofer[8], Gerald Webersinke[8], Helwig Wundsam[9], Holger Rumpold[2, 3, 5], Matthias Biebl[1 3]

[1]*Ordensklinikum Linz, Department of Surgery, Seilerstätte 4, Linz*, [2]*Ordensklinikum Linz, Gastrointestinal Cancer Center, Linz, Österreich*, [3]*Johannes Kepler University Linz, Medical Faculty, Linz, Österreich*, [4]*VYRAL, Visionary Young Researcher Association Linz, Lichtenberg*, [5]*Ordensklinikum Linz, Department of Oncology, Linz, Österreich*, [6]*Ordensklinikum Linz, Department of Diagnostic and Interventional Radiology, Linz, Österreich*, [7]*Medical University Innsbruck, Institute of Human Genetics, Innsbruck, Österreich*, [8]*Ordensklinikum Linz, Laboratory of Molecular Genetic Diagnostics, Linz, Österreich*, [9]*Ordensklinikum Linz, Department of Surgery, Linz, Österreich*

**Background:**
ctDNA derives from distant metastases and advanced locoregional disease in pancreatic cancer (PDAC). Furthermore, ctDNA can be used to predict response to treatment after only 2 weeks of systemic treatment in mPDAC. Applicability for NACT evaluation in localized PDAC was hampered by low detection rates and thus necessity of broad-spectrum analysis and potential delay of treatment in clinical practice up to now.

**Materials and methods:**
Liquid biopsy samples of n=147 patients with locPDAC were collected preoperatively, one (n=66) and 10 days (n=30) after surgery. Samples were evaluated using ddPCR in a novel testing regimen and results were correlated to volumetric findings and clinical outcome, especially disease-free survival.

**Results:**
Pretherapeutic detection rates have improved from 10% in previous analyses with ddPCR from last year to 36.1% (19.7% (1st day), 13.3% (10th day)). ctDNA did not correlate with primary tumor volume (R=0.071, p=0.605), but nodal positivity (p=0.029).
Pretherapeutic ctDNA was associated with significantly worse OS (10.1 (95%CI 4-16.2) vs. 36 (95%CI 22.1-49.9) months) and DFS (6 (95%CI 2.6-9.4) vs. 23.5 (95%CI 17.9-29.1) months).
Pretherapeutic CA19-9 was not associated with OS or DFS. Neither above 500 U/ml (p=0.285, p=0.162), nor >1000U/ml (p=0.419, p=0.495).
Postoperative ctDNA detectability did not reach statistical significance but showed strong correlation to DFS (12.9 (95% CI 6.1-19.7) vs. 29.1 (95% CI 16.6-41.6) months) and correlation to OS (low numbers, HR 3.120, p=0.083).

**Conclusion:**
Our new testing strategy significantly improves the pretherapeutic detection rate (3.6x) with high prognostic impact. Turnaround time from sampling of liquid biopsy to results and thus clinical assessment for treatment decisions is only 3-5 days.
Thus, these findings pave the way for ctDNA guided treatment decisions in locPDAC (NACT vs. upfront surgery) under study conditions.
Based on these findings, we are launching a prospective multicenter study in cooperation of all big HPB units of Austria starting early 2024 with preoperative liquid biopsy based treatment decision (neoadjuvant chemotherapy or upfront surgery) in localized pancreatic cancer.

Abstracts – DCK 2024 – 141st Congress of the German Society of Surgery – Leipzig, April 24–26 • DOI 10.1515/iss-2024-2003
Innov Surg Sci 2024; 9 (Special Suppl 1): s18–s203

**Clinical features and prediction of long-term survival after surgery for perihilar cholangiocarcinoma**
ID: 795

Anna Mantas[1, 2], Carlos C. Otto[1, 2], Daniel Heise[1, 2], Sven A. Lang[1, 2], Tom F. Ulmer[1, 2], Ulf P. Neumann[1, 2], Jan Bednarsch[1, 2]

[1]*Department of Surgery and Transplantation, University Hospital Essen, Essen, Deutschland*,
[2]*Department of Surgery and Transplantation, University Hospital RWTH Aachen, Aachen, Deutschland*

**Background:**
Perihilar Cholangiocarcinoma (pCCA) remains a challenging liver malignancy not only due to its high perioperative complication but also due its dismal long-term prognosis with only a few long-term survivors (LTS) among the patients. Thus, we investigated characteristics and predictors of LTS in pCCA patients.

**Materials and methods:**
Patients undergoing curative-intent liver resection for pCCA between 2010 and 2022 were categorized into long-term and short-term survivors (STS) excluding perioperative mortality. Binary logistic regression was used to determine key differences between the groups and to develop a survival predictor. This predictor variable was subsequently tested in the whole cohort of surgically treated pCCA patients using Cox Regression analysis for cancer-specific survival (CCS).

**Results:**
Within a cohort of 209 individuals, 27 patients were identified as LTS (median CCS= 125 months) and 55 patients as STS (CCS=16 months). Multivariable analysis identified preoperative portal vein filtration (OR =5.85, p=0.018) and intraoperative packed red blood cell (PRBC) transfusions (OR=10.29, p=0.002) as key differences between the groups. A predictor variable based on these two features was created and transferred into a Cox regression model of the whole cohort. Here, the predictor variable (HR=0.35, p<0.001) and nodal metastases (HR=2.15, p=0.001) as well as postoperative complications (HR=3.06, p<0.001) were identified as independently associated with CCS.

**Conclusion:**
Long-term survival after surgery for pCCA is possible and was strongly associated with preoperative portal vein filtration and intraoperative PRBC transfusion. As both variables are part of preoperative staging or can be modulated by intraoperative technique, the proposed predictor variable can easily be transferred into clinical management to predict the oncological risk of patients undergoing surgery for pCCA.

Abstracts – DCK 2024 – 141st Congress of the German Society of Surgery – Leipzig, April 24–26 • DOI 10.1515/iss-2024-2003
Innov Surg Sci 2024; 9 (Special Suppl 1): s18–s203

**Comparison of postoperative hyperlipasemia and hyperamylasemia in predicting pancreatic fistula after distal pancreatectomy**
ID: 797

Ughur Aghamaliyev[1], Gwendolin Seidel[1], Yannick Meyer[1], Bernhard W. Renz[1], Jens Werner[1]

[1]*Klinikum der Universität München, Klinik für Allgemein-, Viszeral- und Transplantationschirurgie, München, Deutschland*

**Background:**
Postoperative pancreatic fistula (POPF) is the most significant source of postoperative morbidity after pancreatic resection and contributes to reduced short and long-term survival. Hence, multiple efforts have been undertaken to predict POPF and identify patients at risk. Recently, emerging data indicate that postpancreatectomy acute pancreatitis (PPAP) might be a critical factor in POPF pathophysiology. Although serum amylase activity has been shown to predict PPAP and POPF, there are currently few reports to predict POPF based on serum lipase activity. Particularly, studies included the predictive role of serum lipase in patients after distal pancreatectomy are lacking. Accordingly, we sought to clarify whether serum lipase can predict POPF in our patient cohort after distal pancreatectomy.

**Materials and methods:**
Consecutive patients who underwent pancreatic resection from January 2018 to December 2022 at our hospital were identified retrospectively. Clinical data were collected including age, gender, BMI, serum amylase and lipase levels on POD0, POD1, POD2 and POD3.

**Results:**
A total of 203 patients underwent distal pancreatectomy (DP) between January 2018 and December 2022 at our institution. The median age of the patients was 65 (range 18–90) years, and there were 112 men (55.2%) and 91 women (44.8%). Postoperatively, median range of serum lipase and amylase was higher in the POPF group compared to the non-POPF group. Out of the 104 patients for whom serum lipase levels were available on POD1, 54.6% exhibited postoperative hyperlipasemia (POHL) on POD1, while only 26.7% presented with postoperative hyperamylasemia (POHA). In the multivariate analysis, POHL on POD1 was identified as a significant predictor of POPF.

**Conclusion:**
Postoperative hyperlipasemia on POD1 is an effective tool in predicting POPF after distal pancreatectomy.

**The PAncreatic surgery Composite Endpoint (PACE) – Development and validation of a clinically relevant endpoint requiring lower sample sizes**
ID: 820

Felix Nickel[1, 2], Christoph Kuemmerli[3], Philip Müller[3], Mona Schmidt[4], Leon Schmidt[4], Philipp Wise[1, 2], Rosa Klotz[2], Christin Tjaden[2], Pascal Probst[5], Thilo Hackert[1, 2], Markus Büchler[2, 6]

[1]*Klinik für Allgemein-, Viszeral- und Thoraxchirurgie - Universitätsklinikum Hamburg-Eppendorf - UKE, Hamburg, Deutschland*, [2]*Chirurgische Klinik, Heidelberg, Deutschland*, [3]*Clarunis Universitäres Bauchzentrum Basel , Basel, Schweiz*, [4]*Universitätsmedizin der Johannes Gutenberg-Universität Mainz, Mainz, Deutschland*, [5]*Kantonsspital Frauenfeld, Frauenfeld, Schweiz*, [6]*Botton-Champalimaud Pancreatic Cancer Centre, Lisbon, Portugal*

**Background:**
Advances in pancreatic surgery and perioperative care have led to a decline in morbidity and mortality. Single endpoints in prospective and randomized studies have become impractical due to their low frequency and the marginal benefit of new interventions. Composite endpoints aim to increase the clinically relevant event rate and minimize the required patient sample size.

**Materials and methods:**
Individual data of 1’273 patients from six randomized multicentric studies and one prospective study were used to develop and validate a composite endpoint based on the pancreatectomy-specific outcome measurements postoperative pancreatic fistula (POPF), postpancreatectomy hemorrhage (PPH) as well as reoperation and reinterventions. All patients had pancreatectomies of different extents. The association of the developed PAncreatic surgery Composite Endpoint (PACE) with length of hospital stay (LOS) and mortality was assessed. A single-institution database was used to externally validate the novel composite endpoint on data from 2’666 distinct patients. Sample size calculations were made for single outcomes and the composite endpoint.

**Results:**
POPF, PPH and reoperation/reintervention were included in the model. In the validation cohort, the PACE demonstrated a ROC of 78.0%, a sensitivity of 90.4% and a specificity of 67.6% in predicting a prolonged LOS (>75th percentile). In the external cohort, the ROC was 76.9% with a sensitivity of 73.8% and a specificity of 80.1%. The 90-day mortality rate was significantly different for patients with a positive versus a negative PACE both in the development (5.1% vs 0.9%; p< 0.001) and external validation cohort (8.5% vs 1.2, p< 0.001). The PACE enabled sample size reductions of up to 80.5% compared to single endpoints.

**Conclusion:**
The PAncreatic surgery Composite Endpoint PACE performed well in predicting prolonged hospital stays and perioperative mortality. PACE includes POPF, PPH and reoperation/reintervention as a binary endpoint. PACE can be used as a standardized and clinically relevant endpoint for future prospective trials with lower sample sizes and therefore improved feasibility compared to single outcome parameters.

Abstracts – DCK 2024 – 141st Congress of the German Society of Surgery – Leipzig, April 24–26 • DOI 10.1515/iss-2024-2003
Innov Surg Sci 2024; 9 (Special Suppl 1): s18–s203

**Perioperative risk factors for Liver Perfusion Failure in patients undergoing Pancreatoduodenectomy**
ID: 836

Sakher Shraim[1], Julian M. Deisenhofer[1], Hendrik B. Sauer[1], Leonie Frank-Moldzio[1], Tom Bruckner[2], Alina S. Ritter[1], Gabriel Salg[1], Philipp Mayer[3], Miriam Klauss[3], Martin Loos[1], Arianeb Mehrabi[1], Martin Schneider[1], Thilo Hackert[1], Markus W. Büchler[1], Oliver Strobel[1 4], Mohammed Al-Saeedi[1]

[1]*Department of General, Visceral and Transplantation Surgery, Heidelberg University Hospital, Heidelberg, Germany*, [2]*Institute of Medical Biometry and Informatics, Heidelberg University Hospital, Heidelberg, Germany*, [3]*Department of Diagnostic and Interventional Radiology, Heidelberg University Hospital, Heidelberg, Germany*, [4]*Division of Visceral Surgery, Department of General Surgery, Medical University of Vienna, Vienna, Austria*

**Background:**
Ischemic liver complications following pancreatic surgery are known to be rare but potential life-threatening events. The precise definition and perioperative risk factors remain inadequately understood. This observational study aimed to establish a comprehensive definition for liver perfusion failure (LPF) and to identify perioperative risk factors associated with its occurrence in patients undergoing pancreatoduodenectomy (PD) or total pancreatoduodenectomy (TP).

**Materials and methods:**
Data of 918 patients who underwent PD/TP between 2014 and 2017 at Heidelberg University Hospital was retrospectively analyzed. Various potential definitions of LPF were assessed for their clinical relevance, ultimately defining LPF as a two-consecutive- day increase in liver enzymes (AST/ALT) to ≥200 U/l . LPF severity was categorized as mild (≥200-<500 U/l), moderate (≥500-<1000 U/l), or severe (≥1000 U/l). Univariate and multivariate analyses were conducted to identify perioperative risk factors.

**Results:**
Of all patients, 81 (9.2%) were identified with LPF, with 29 (3.3%) categorized as mild, 29 (3.3%) as moderate, and 23 (2.6%) as severe. LPF was significantly associated with postoperative complications, including the need of additional radiological interventions, liver-specific complications, liver failure, longer hospital stays, and IMCU stays ($p<0.001$). Multivariate analysis identified celiac axis stenosis (CAS), arterial resection, and the duration of the operation ($p<0.001$) as independent risk factors for LPF.

**Conclusion:**
LPF is a serious and often underestimated postoperative complication in patients undergoing PD/TP. This study introduces a well-defined classification of LPF and highlights critical risk factors. Future research should focus on strategies to mitigate these risk factors.

**Anticoagulation after portal vein reconstruction during pancreaticoduodenectomy – A systematic review**
ID: 853

Max Heckler[1], Susanne Roth[1], Thomas Hank[1], Jörg Kaiser[1], Benedict Kinny-Köster[1], Martin Loos[1]

[1]*Universitätsklinikum Heidelberg, Klinik für Allgemein-, Viszeral- und Transplantationschirurgie*

**Background:**
Portal vein (PV) and superior mesenteric vein (SMV) resection and reconstruction enables surgical removal of borderline-resectable and locally advanced tumors of the pancreatic head. Thrombosis/Occlusion of the reconstructed vessel represents a major source of morbidity, as the growing literature on this increasingly popular intervention suggests. To date no universally accepted standard for anticoagulation exists. Here we aim to evaluate the existing literature on the topic with special regard to type of reconstruction.

**Materials and methods:**
This systematic review was conducted in adherence to the 2009 PRISMA guidelines. Studies reporting on portomesenteric (PM) resection and reconstruction reporting data on occlusion/thrombosis rates were included. Pubmed/Medline and Embase databases were searched. The following parameters were assessed: Study type, year of publication, number of patients, type/number of PM reconstruction (according to the International Study Group for Pancreatic Surgery), follow up period, mortality, rate of thrombosis/occlusion of reconstructed PM axis, intraoperative blood loss, anticoagulation strategy. Study quality and risk of bias for the included studies was assessed using the Newcastle-Ottawa scale for non-randomized trials.

**Results:**
16 studies eligible for inclusion were identified in the systematic search conducted. A total of 1844 patients were included. All studies were observational, retrospective studies. No randomized controlled trial was identified.
Of a total of 1844 patients among the 16 studies, 520 received tangential resection of the SMV/PV with venorrhaphy or patch repair, 961 patients had segmental resection with end-to-end reconstruction and 363 patients received a type 4 reconstruction with an interposition graft/conduit. Type 4 graft patients were further stratified into synthetic/PTFE grafts (n=138 patients), interposition via autologous grafts (n=160 patients) and via allogenic/cadaver graft (n=65 patients).
Overall thrombosis/occlusion rate was 18% and subgroup analysis according to the type of reconstruction revealed highest occlusion rates of 26% in patients with synthetic PM conduits and 54% in patients with allogenic grafts.
Data on anticoagulation was provided for 14 studies. In 2 studies no postoperative anticoagulation was prescribed, while postoperative low-molecular weight heparinization (LMWH) in varying doses was utilized in 4 studies. Warfarin and Aspirin were reported as postoperative anticoagulation in 2 studies each. 2 studies had no explicit institutional anticoagulation strategies. Reporting of anticoagulation strategies was heterogeneous, with relevant information missing in most included studies. Intraoperative heparinization strategies were reported in 4 studies.
Thrombotic/occlusive events within the early postoperative phase occurred in 5% and 6% after tangential resection and reconstruction (Type 1 and 2) and segmental resection with direct anastomosis (Type 3). Rates were elevated in patients after type 4 reconstruction: PTFE grafts had occlusion rates of 9%, autologous veins of 8%, and reconstruction with allogenic grafts resulted in occlusion rates of 21%.
Patients with an acute thrombotic event suffered from significant perioperative mortality: Data from 6 studies allowed for correlation of mortality and acute occlusive event. Among 29 early events, 7 were directly associated with the postoperative death of the patient (24%).

**Conclusion:**
Synthetic and allogenic interposition grafts harbor the highest risk of thrombosis/occlusion among the different types of reconstruction after PM resection. Early thrombosis/occlusion is associated with relevant morbidity and mortality. Anticoagulation protocols show great heterogeneity, no single approach can be considered beneficial on the basis of the existing literature.

Abstracts – DCK 2024 – 141st Congress of the German Society of Surgery – Leipzig, April 24–26 • DOI 10.1515/iss-2024-2003
Innov Surg Sci 2024; 9 (Special Suppl 1): s18–s203

**Impact of an enhanced anti-infection prophylaxis strategy for pancreatoduodenectomy – A single centre analysis**
ID: 856

Benjamin Müssle[1], Tina Gross[2], Felix Merboth[3], Anna Klimowa[4], Christoph Kahlert[5], Jürgen Weitz[6], Thilo Welsch[7]

[1]*Uniklinik Ulm, Klinik für Allgemein und Viszeralchirurgie, Ulm, Deutschland*, [2]*Uniklinik Hamburg Eppendorff, Chirurgische Klinik, Hamburg, Deutschland*, [3]*Klinik für Allgemein-, Viszeral- und Thoraxchirurgie, Chirurgische Klinik, Dresden, Deutschland*, [4]*NCT Dresden, Dresden, Deutschland*, [5]*Uniklinik Heidelberg, Chirurgische Klinik, Heidelberg, Deutschland*, [6]*Uniklinik Dresden, Klinik für Allgemein- Viszeral- und Thoraxchirurgie, Dresden, Deutschland*, [7]*Uniklinik Hamburg Eppendorff, Klinik für Allgemein- Viszeral- und Thoraxchirurgie, Hamburg, Deutschland*

**Background:**
Surgical site infection (SSI) after pancreatoduodenectomy (PD) is a significant concern. Targeted antibiotic prophylaxis (pAP) has been tested to mitigate antibiotic resistance patterns, especially after preoperative bile duct stenting. The aim of this study was to investigate the effect of enhanced anti-infective prophylaxis (EAP) on the incidence of superficial and intraabdominal SSI.

**Materials and methods:**
All patients who underwent PD at a single centre between May 2018 and May 2021 were retrospectively analysed. A control cohort of patients who received pAP with intravenous cefuroxime and metronidazole and routine intraoperative abdominal lavage according to the surgeons' preferences. Since March 2020, pAP has been changed to piperacillin/tazobactam according to local resistance patterns and combined with routine intraoperative extended abdominal lavage (EIPL). Preoperative selective decontamination of the digestive tract (SDD) has been applied routinely since Jan 2019.

**Results:**
In total, 163 patients were included. The standard (n=100) and EAP (n=63) groups did not significantly differ with regard to pertinent patient and operative characteristics. In the EAP group, the rates of SSI (14% vs. 37%, p=0.002, total rate: 28%) and urinary tract infection (24% vs. 8%, p=0.011, total rate 18%) were significantly lower. Other septic complications were not significantly different. In addition, the risk of developing gastrointestinal bleeding and delayed gastric emptying was significantly lower in the EAP group. Multivariate analysis showed that EAP and age >67 years were significant risk factors for SSI.

**Conclusion:**
The results indicate that enhanced anti-infective prophylaxis may significantly decrease the incidence of SSI in patients after PD.

**Whole 16S rRNA sequencing of the oral microbiome predicts postoperative pancreatic fistula – a prospective observational cohort study**
ID: 878

Azadeh Azizian[1, 2], Christoph Ammer-Herrmenau[3], Catharina Lingens[3], Charlotte Ratei[3], Cathleen Heuer[3], Kai Antweiler[4], Jacob Hamm[3], Soeren Buchholz[3], Michael Ghadimi[2], Volker Ellenrieder[3], Albrecht Neeße[3]

[1]*Städtisches Klinikum Karlsruhe, Allgemein-/Viszeralchirurgie, Karlsruhe, Deutschland*, [2]*Universitätsklinikum Göttingen, Allgemein-, Viszeral- und Kinderchirurgie, Göttingen, Deutschland*, [3]*Universitätsmedizin Göttingen, Klinik für Gastroenterologie und gastrointestinale Onkologie, Göttingen*, [4]*Universitätsmedizin Göttingen, Institut für Medizinische Statistik, Deutschland*

**Background:**
Postoperative pancreatic fistula (POPF) is a common and potentially severe complication following pancreas surgery. Its prevalence ranges from 30 – 40 % for pancreatic left-sided resection, and 15 – 20 % for pancreatic head surgery. To date, there is no objective predictor anticipating the occurrence of POPF.

**Materials and methods:**
In total 105 patients were prospectively recruited before elective pancreas surgery. Exclusion of patients with pancreatectomy and delayed surgery. N=75 buccal and n=70 rectal samples were included in the subsequent analysis. Microbial composition was determined by Oxford Nanopore Technologies using whole 16S rRNA and metagenomic sequencing.

**Results:**
Significant differences between groups were detected for β-diversity (unweighted UniFrac, p-value = 0.003) and α-diversity (observed species, p-value = 0.004) in buccal but not in rectal swabs. After considering of 67 potentially confounding variables, the results remain significant.
BMI was the most obvious confounding factor. Four species were found to be differentially abundant in the POPF group (buccal samples): *Streptococcus gorondii, Gemella haemolysans, Haemophilus parainfluenzae, Gemella sanguinis* (Figure 3). Building a model with these species and the BMI resulted in an AUROC of 93.9 %

**Conclusion:**
Our results show that POPF can be predicted by the oral microbiome in combination with BMI.

Abstracts – DCK 2024 – 141st Congress of the German Society of Surgery – Leipzig, April 24–26 • DOI 10.1515/iss-2024-2003
Innov Surg Sci 2024; 9 (Special Suppl 1): s18–s203

**Single Center Experience in Implementing a Minimally Invasive Program for Liver Surgery: A Comparative Analysis of Hand-Assisted Laparoscopic Surgery (HALS), Laparoscopic Liver Resection (LLR), and Robotic Liver Resection (RLR)**
ID: 891

Nils Haep[1, 2], Lucas Illies[1], Felix Krenzien[1, 2], Georg Lurje[1], Johann Pratschke[1], Wenzel Schöning[1]

[1]*Charité, Chirurgische Klinik | CCM | CVK, Berlin, Deutschland*, [2]*Berlin Institute of Health (BIH)*

**Background:**
In the past decade, minimally invasive techniques have gained increasing importance in liver surgery. This study describes our single-center experience implementing a minimally invasive liver surgery programme, including hand-assisted laparoscopic surgery (HALS), laparoscopic liver resection (LLR), and robotic liver resection (RLR). The primary objective was to evaluate patient safety using the Clavien-Dindo Classification for complications in 1009 surgical procedures performed between 2015 and 2023.

**Materials and methods:**
We performed a retrospective analysis of 1009 liver surgeries, which included 106 HALS, 643 LLR, and 260 RLR cases. Age, sex, and diagnosis were compared between the three groups. The resections were classified as major or minor, and postoperative complications (Clavien-Dindo Grade >III) were assessed. To identify factors associated with postoperative complications, univariable and multivariable analyses were performed. Using logistic regression models, the impact of various variables on the complication rates was determined.

**Results:**
The demographics of the patients, including age, sex, and diagnosis, did not differ significantly between the HALS, LLR, and RLR groups. However, we found that LLR was utilised more frequently for minor liver resections than HALS and RLR (p=0.001). There were no significant differences in the incidence of severe complications (Clavien-Dindo >III) between the three surgical techniques. In our univariable and multivariable analyses, age, sex, diagnosis, and surgical year had no significant impact on the incidence of Clavien-Dindo complications >III. However, the odds ratio (OR) for major liver resections was 1.96 (95% confidence interval [CI]: 1.45-2.65, p=0.001), indicating a higher risk of complications in major resections. In contrast, LLR was associated with a lower risk of complications with an odds ratio (OR) of 0.47 (95% confidence interval [CI]: 0.31 to 0.71, p=0.001).

**Conclusion:**
Our single-center experience demonstrates that it is safe to implement a minimally invasive liver surgery programme that includes HALS, LLR, and RLR. There are no significant differences between these techniques in terms of the incidence of severe postoperative complications. During the implementation phase, HALS is a viable option for complex procedures without a significant increase in complications. Major resections can be performed safely by RLR. These findings support the continued adoption of minimally invasive techniques in liver surgery to enhance patient outcomes and surgical practise.

**Recurrence Patterns After Resection of Non-invasive IPMN with Main Duct Involvement: Challenges in Postoperative Management of IPMN.**
ID: 893

Jörg Kaiser[1], Jin Lee[1], Ulf Hinz[1], Philipp Mayer[2], Christine Tjaden[1], Susanne Roth[1], Max Heckler[1], Benedict Kinny-Koester[1], Markus W. Büchler[1], Martin Loos[1]

[1]*Universitätsklinikum Heidelberg, Abteilung für Allgemein-, Viszeral- u. Transplantationschirurgie, Heidelberg, Deutschland*, [2]*Universitätsklinikum Heidelberg, Abteilung für Radiologie, Heidelberg, Deutschland*

**Background:**
Intraductal papillary mucinous neoplasms (IPMN) exhibit the risk of malignant progression, especially when the main pancreatic duct is involved. Several studies have focused on surgical indications and methods for IPMN, leaving optimal postoperative management measures undetermined. The aim of the study was to assess risk factors and rates of recurrence in patients after surgery for non-invasive IPMN with main duct involvement.

**Materials and methods:**
Clinicopathological records and long-term follow-up data from patients who underwent resection for non-invasive main duct- and mixed-type IPMN between 2004 and 2020 were reviewed and analyzed using uni- and multivariate analyses.

**Results:**
Among 225 patients who underwent resection for non-invasive IPMN with main duct involvement, median follow-up duration was 63 months. Recurrence of IPMN was identified in 92 patients (41%). Seven out of 27 patients (26%) who underwent a second surgery due to IPMN recurrence in the remnant pancreas displayed histopathological evidence of IPMN-associated carcinoma. Remarkably, five of these patients had initially presented with low- or intermediate-grade IPMN. Higher-grade dysplasia observed during the index operation did not correlate with higher postoperative recurrence rates. Independent predictors of recurrence in patients with IPMN and main duct involvement included the presence of multifocal lesions throughout the entire pancreas (p=0.024, HR 2.276) and the persistence of residual lesions in the remnant pancreas (p=0.026, HR 1.866). Notably, 15% of recurrent lesions emerged more than 5 years after the initial surgery.

**Conclusion:**
Given the considerable recurrence rate following surgery for non-invasive IPMN with main duct involvement (41%) and the potential for late-onset recurrence, the imperative of lifelong surveillance and regular follow-up examinations becomes evident. Our data underscore the necessity of implementing a postoperative management scheme, irrespective of the initial histological grading, as any IPMN lesion involving the main duct may harbor the potential for malignant progression.

**Safety and performance of a synthetic sealant patch aimed to prevent postoperative pancreatic fistula after distal pancreatectomy (SHIELDS) –prospective international multicenter phase II study**

ID: 896

Mara Goetz[1], Asmus Heumann[2], Luca Landoni[3], Karl Khatib-Chahidi[4], Michael Ghadimi[5], Maximillian von Heessen[5], Frederik Berrevoet[6], Filip Gryspeerdt[6], Marc Besselink[7], Eduard van Bodegraven[7], Mustapha Adham[8], Johann Pratschke[9], Wenzel Schöning[9], Jakob Izbicki[1], Maximilian Bockhorn[4]

[1]*Department of General, Visceral and Thoracic Surgery, University Medical Centre Hamburg-Eppendorf, Hamburg, Deutschland*, [2]*Department of General, Visceral and Thoracic Surgery, University Medical Centre Hamburg-Eppendorf, Deutschland*, [3]*Department of Surgery, Pancreas Institute, Verona University Hospital, Verona, Italy*, [4]*Department for General- and Visceral Surgery, University Hospital Oldenburg, Oldenburg, Deutschland*, [5]*Department of General, Visceral, and Pediatric Surgery, University Medical Center Göttingen, Göttingen, Deutschland*, [6]*Department of General and Hepatopancreatobiliary Surgery and Liver Transplantation, Ghent University Hospital, Ghent, Belgium*, [7]*Department of Surgery, Amsterdam UMC, Location University of Amsterdam, Amsterdam, Netherlands*, [8]*Department of Digestive, Hepato-Biliary and Pancreatic Surgery, Edouard Herriot Hospital, Lyon, France*, [9]*Department of Surgery, Campus Charité Mitte and Campus Virchow-Klinikum, Charité- Universitätsmedizin Berlin, Berlin, Deutschland*

**Background:**

The incidence for clinically relevant postoperative pancreatic fistulas (CR-POPF) in distal pancreatectomy (DP) ranges up to 25%. None of the available sealants significantly reduce CR-POPF and demonstrate the need for a new innovative approach. A new biodegradable sealant patch was developed to reduce fluid leakage and to achieve bleeding control. This sealant patch was able to reduce POPF and was superior to the standard of care in a preclinical porcine DP model. The aim of this first-in-human study was to clinically assess the safety and performance of the sealant patch.

**Materials and methods:**

In this multicenter, single-arm, study 40 patients undergoing distal pancreatectomy were prospectively enrolled from 8 centers in 5 countries. Following surgical resection, the transection plane was closed according to the standard of care and subsequently covered with the sealant patch. As primary endpoint the incidence of CR-POPF up to 30-days postoperatively was evaluated. The secondary endpoints included the assessment of bleeding control, leak-associated comorbidities and device usability.

**Results:**

Among 40 patients after distal pancreatectomy, CR-POPF occurred in 7 (17.5%) up to postoperative day 30. No type C POPF (0%) was observed. There was no intraoperative bleeding observed after patch application.

**Conclusion:**

The results of this international phase II study demonstrate promising results of a new sealant patch regarding the rate of CR-POPF. Randomized studies are now needed to confirm the superiority of the current patch as compared to the best current practice.

Abstracts – DCK 2024 – 141st Congress of the German Society of Surgery – Leipzig, April 24–26 • DOI 10.1515/iss-2024-2003
Innov Surg Sci 2024; 9 (Special Suppl 1): s18–s203

### Long-term Quality of Life after Surgery for Intraductal Papillary Mucinous Neoplasms in Young Patients

ID: 902

Jörg Kaiser[1], Ulf Hinz[1], Philipp Mayer[2], Christine Tjaden[1], Susanne Roth[1], Max Heckler[1], Benedict Kinny-Köster[1], Markus W. Büchler[1], Martin Loos[1]

[1]*Universitätsklinikum Heidelberg, Abteilung für Allgemein-, Viszeral- u. Transplantationschirurgie, Heidelberg*, [2]*Universitätsklinikum Heidelberg, Abteilung für Radiologie, Heidelberg*

**Background:**
Surgery for intraductal papillary mucinous neoplasms (IPMN) of the pancreas is infrequent among young individuals and carries inherent morbidity risks. The potential influence of postoperative morbidity on long-term post-pancreatic surgery quality of life (QoL) remains uncertain. The aim of the following study was to evaluate the QoL outcomes following IPMN resection in patients aged 50 or younger.

**Materials and methods:**
Perioperative and long-term follow-up data of patients who had undergone resection for IPMN between 2004 and 2020, were analyzed. QoL was assessed using the EORTC QLQ-C30 and QLQ-PAN26 questionnaires.

**Results:**
A total of 78 patients underwent surgical treatment, including those with benign IPMN (low-grade: n=22 and intermediate-grade: n=21) as well as malignant IPMN (high-grade: n=16 and IPMN-associated carcinoma: n=19). Severe postoperative morbidity (Clavien-Dindo ≥III) occurred in only 14 patients (18%). No perioperative mortality was observed. Evaluation using the QLQ-C30 questionnaire revealed a mean global health status of 71.1%, alongside overall functioning and symptom scores of 82.9% and 16.8%, respectively. Additionally, QLQ-PAN26 showed an overall symptom score of 20.7%.

**Conclusion:**
Surgery for IPMN appears to be a safe option with low morbidity and potentially no mortality for young patients. It offers young patients excellent long-term outcomes in terms of QoL. Until the true biological nature of IPMN is better understood, along with individual risk factors for IPMN progressing to malignancy, the excellent long-term quality of life outcomes support the consideration of pancreatic resection in young individuals with a long life expectancy.

Abstracts – DCK 2024 – 141st Congress of the German Society of Surgery – Leipzig, April 24–26 • DOI 10.1515/iss-2024-2003
Innov Surg Sci 2024; 9 (Special Suppl 1): s18–s203

**Osteopenia is not associated with worse perioperative and oncological outcome in patients undergoing liver resection for cholangiocarcinoma**
ID: 915

Zoltan Czigany[1], Franziska Meister[2], Jan Bednarsch[3], Iakovos Amygdalos[2], Katharina Jöchle[2], Esref Belger[2], Florian Vondran[2], Ulf Neumann[2], Martin von Websky[2]

[1]*Universitätsklinikum Heidelberg, Heidelberg, Deutschland*, [2]*Uniklinik RWTH Aachen, Aachen, Deutschland*, [3]*Universitätsklinikum Essen, Essen, Deutschland*

**Background:**
Cholangiocarcinoma (CCA) of the liver is an uncommon and aggressive cancer that originates in the bile ducts. The overall prognosis for CCA is unfavorable due to limited treatment options and often late diagnosis. Until today, radical surgery is still the only curative treatment option. Patients in need of a major liver resection for CCA face an elevated risk of perioperative morbidity, thus proper patient selection and risk-stratification are essential. This study aims to investigate the predictive potential of bone mineral density (BMD), assessed using preoperative computed tomography (CT), in the context of major perioperative morbidity (Clavien Dindo >3b and Comprehensive Complication Index (CCI)), mortality, and long-term oncological results

**Materials and methods:**
A total of 202 patients who underwent surgery with curative intent for either intrahepatic cholangiocarcinoma (iCCA; n=97) or perihilar cholangiocarcinoma (pCCA; n=105) during the period from 2010 to 2019 were included into the analysis. Preoperative computed tomography-based segmentation was used to evaluate BMD. The measurement was acquired at the level of the 12th thoracic vertebra. Osteopenia was defined based on pre-described cutoffs.

**Results:**
Osteopenia was highly prevalent in both iCCA (53/97, 54%) and pCCA (54/105, 51%) sub-cohorts. Patients suffering from Osteopenia were significantly older than those without (71.1 [62-76.6] years vs. 61.3 [52.9-69.2] years; p<0.001) Alteration in BMD did not demonstrate a significant prognostic effect in terms of perioperative morbidity (Mann-Whitney U; CCI: 34 [9-56] vs. 40 p=0.185; iCCA:p=0.803; CCI: pCCA:p=0.165). The median overall survival in our cohort was 19 [14-25] months. Patients with Osteopenia did not exhibit a significantly different overall survival compared to those with normal bone mineral density (log-rank p=0.234)

**Conclusion:**
In contrast to our previous observations in other oncological patient cohorts, osteopenia alone had no significant negative impact on clinical outcomes in patients undergoing curative-intent surgery for CCA. Further prospective studies are warranted to validate these retrospective findings.

**Robotic Minimally Invasive Inguinal Hernia Repair with The Dexter Robotic System™: a Prospective multicenter clinical investigation**
ID: 307

Julius Pochhammer[1, 2], Thomas Becker[1, 2], Lukas Gantner[3], Hubert Mignot[4], Stefan Breitenstein[3]

[1]*Klinik für Allgemein, Viszeral-, Thorax-, Transplantations- und Kinderchirurgie, UKSH, Campus Kiel, Kiel, Deutschland*, [2]*Kurt-Semm-Zentrum für laparoskopische und roboterassistierte Chirurgie, UKSH, Campus Kiel, Kiel, Deutschland*, [3]*Klinik für Viszeral- und Thoraxchirurgie, Kantonsspital Winterthur, Winterthur, Schweiz*, [4]*Chirurgie urologique et générale, Centre hospitalier de Saintes, Saintes, France*

**Background:**
Robot-assisted transabdominal preperitoneal inguinal hernia repair (rTAPP) has been proven safe and feasible with various robotic platforms. The Dexter robotic system is an open platform consisting in a sterile surgeon’s console, two robotic instrument arms, and one robotic endoscope arm. This study aimed to confirm the perioperative and early postoperative safety and clinical performance of the Dexter Robotic System, in patients undergoing primary transperitoneal inguinal hernia repair.

**Materials and methods:**
We conducted a prospective multicentre study at three centres in France, Germany, and Switzerland (ClinicalTrials.gov Identifier NCT05873582). The primary objectives of the study were to document the successful completion of the rTAPP procedures and to collect data on the occurrence of serious adverse events (Clavien - Dindo grades III-V), device-related events, and other adverse events perioperatively and up to 30 days post-surgery. The procedures were performed by three surgeons with varying levels of experience in robotic systems, including novice, intermediate and expert.

**Results:**
Fifty patients were operated for inguinal hernia repair (33 unilateral, 17 bilateral). The mean age and BMI of the patients were 60 years (± 15) and 25.55 kg/cm$^2$ (± 3.33), respectively. All surgeries were successfully completed as planned using three standard laparoscopy trocars sized 10/12mm. Each procedure was performed with robotic assistance, and there were no conversions to open surgery. Intra-operative complications and device deficiency were not observed. The mean skin-to-skin operative time was 55 min (±14) for unilateral hernia repair and 94 min (± 24) for bilateral hernia repair. The mean robotic console time was 35 min (±14) for unilateral hernia and 71 min (± 23) for bilateral hernia repairs. The patients were discharged within 1 day (± 1 day) after surgery.

**Conclusion:**
Our experience with robotic TAPP utilizing the Dexter system confirms its feasibility and safety, with operative times aligning with those reported in the literature for other robotic platforms. This study demonstrates the practicality and safety of the robotic approach, even when adopted by a surgeon new to robotic techniques. The Dexter system emerges as a valuable device in the toolkit of hernia repair, offering promising results for both experienced robotic surgeons and those entering the field.

**Robotic-assisted repair of incisional hernia - early experiences of a university robotic hernia program and comparison with open and minimally invasive sublay technique (eMILOS)**
ID: 422

Gabriel Plitzko[1], Björn-Ole Stüben[1], Anastasios Giannou[1], Matthias Reeh[1], Jakob Izbicki[1], Nathaniel Melling[1], Michael Tachezy[1]

[1]*Klinik für Allgemein-, Viszeral- und Thoraxchirurgie, Universitätsklinikum Hamburg-Eppendorf, Hamburg, Deutschland*

**Background:**
With robotic surgical devices an innovative tool has stepped into the arena of minimally invasive hernia surgery. It combines the advantages of open (low recurrence rates and ability to perform complex procedure such as transverse abdominis release) and laparoscopic surgery (low rate of wound and mesh infections, less pain). However, a superiority to standard minimally invasive procedures has not yet been proven. We present our first experiences of robotic mesh repair of incisional hernias and a comparison of our results with open and minimally invasive sublay techniques.

**Materials and methods:**
A retrospective analysis of all patients who underwent robotic-assisted mesh repair (RAHR) for incisional hernia between April and November 2022 (RAHR group) and patients who underwent open sublay (Sublay group) or eMILOS hernia repair (eMILOS group) between January 2018 and November 2022 was carried out. Patients in the RAHR group were matched 1:2 to patients in the Sublay group by Propensity Score Matching. Patient demographics, preoperative hernia characteristics and cause of hernia, intraoperative variables and postoperative outcomes were evaluated. Furthermore, a subgroup analysis of only midline hernia was performed.

**Results:**
A total of 21 patients received robotic-assisted incisional hernia repair. Procedures performed included robotic retro-muscular hernia repair (r-RMHR, 76%), with transverse abdominis release in 56% of the cases. In one patient r-RHMR was combined with robotic inguinal hernia repair. Two patients (10%) were operated with total extraperitoneal technique (eTEP). Robotic-assisted transabdominal preperitoneal hernia repair (r-TAPP) was performed in three patients (14%). Median (range) operating time in the RAHR group was significantly longer than in the Sublay and eMILOS group (291 (122-311) vs. 109,5 (48-270) minutes vs. 123 (100-192) minutes, respectively, $p<0.001$). The meshes applied in the RAHR group were significantly compared to the Sublay (mean (SD) 529±311 $cm^2$ vs. 356±231, $p=0.037$), but without a difference compared to the eMILOS group (mean (SD) 596±266 $cm^2$). Median (range) length of hospital stay in the RAHR Group was significantly shorter compared to the Sublay group (3 (2-7) vs. 5 (1-9) days, $p=0.032$), but not significantly different to the eMILOS group. In short term follow-up, no hernia recurrence was observed in the RAHR and eMILOS group, with 9% in the Sublay group. The subgroup analysis of midline hernia revealed very similar results.

**Conclusion:**
Our data show a promising outcome after robotic assisted incisional hernia repair, but no superiority compared to the eMILOS technique. However, RAHR is a promising technique especially for complex hernia in patients with relevant risk factors, especially immunosuppression. Longer follow up times are needed to accurately assess recurrence rates and large prospective trials are needed to show superiority of robotic compared to standard open and minimally invasive hernia repair.

**Laparoscopic posterior hiatoplasty and esophagophrenicopexy for symptomatic hiatal hernia after total gastrectomy with temporary incarceration episodes**
ID: 666

Reiko Wießner[1], Alexander Gehring[1], Mattes Lüth[1], Christiane Hoth[1], Tina Görl[1]

[1]*Ribnitz-Damgarten, Bodden-Kliniken Ribnitz-Damgarten GmbH, Ribnitz-Damgarten, Deutschland*

**Background:**
A 41-year-old, slim female patient had been suffering from severe dysphagia for six months, accompanied by subileus symptoms after total gastrectomy 10 years ago for pT1b, pN3a, (7/54), cM0, V1, L1, Pn1, R0, G3/4 (UICC stage II b) of signet ring cell adenocarcinoma of the stomach. Further diagnosis using various imaging modalities (CT, MRI), endoscopy including capsule endoscopy provided evidence of symptomatic irreducible esophagojejunostomy with hiatal hernia in the posterior mediastinum.

**Materials and methods:**
The patient suffered from extreme nausea with vomiting and pain in the upper and middle abdomen at regular, approximately 6-week intervals (NAS 4/10). Serochemically, an unremarkable CrP level and a single leukocytosis of 11.52 Gpt/l were noted during the respective inpatient admissions. Electrolytes, renal retention parameters, transaminases, and U status were normal. The CT scan excluded ileus and peritoneal carcinomatosis as late manifestations of gastric carcinoma. Both CT and endoscopy revealed an anastomosis in the posterior mediastinum with a long blind limb and evidence of a partial (transient) hernia. This hernia had not been present on previous years' endoscopies by the same examiner.

**Results:**
Intraoperatively, after adhesiolysis, an irreducible, intermediately well incarcerated esophagojejunostomy with small hiatal hernia (HSA: 4 $cm^2$) was confirmed. Laparoscopic reduction of the esophagojejunostomy, posterior hiatoplasty and esophagophrenicopexy on the right side were performed.

**Conclusion:**
Irreducible and incarcerated hiatal hernias after total gastrectomy are extremely rare and represent a challenge for every surgeon. The incidence of hiatal hernia is 0.19% three years after gastrectomy with laparoscopic resections leading to a higher incidence (0.53%). In the literature, herniation with complications usually occurs within the first year after gastrectomy - in this respect, our case 10 years later was an absolute rarity.

**How often is prophylactic parastomal mesh placement performed after rectal resection without sphincter preservation? An analysis of German nationwide hospital discharge data among 41,697 patients**
ID: 777

Christoph Paasch[1], Elisa Kobelt[1], Sebastian Lünse[1], Sophie Heisler[1], Ralph Lorenz[1], Richard Hunger[1], Rene Mantke[1]

[1]*Universitätsklinikum Brandenburg an der Havel, Allgemein- und Viszeralchirurgie, Brandenburg, Deutschland*

**Background:**
The European Hernia Society guidelines of parastomal hernias, published in 2017, strongly recommend prophylactic synthetic nonabsorbable mesh upon the construction of a permanent end colostomy to reduce the incidence of parastomal hernias. This study aims to evaluate the implementation of the guidelines in Germany.

**Materials and methods:**
This is a retrospective multicentric analysis conducted in December 2022 at the University Hospital Brandenburg an der Havel. Anonymous data on rectal resection without sphincter preservation in the period 2010-2020 were extracted from the German nationwide hospital discharge data set. Individuals with a hernia and <18 years old were excluded. Another exclusion criterion was a performed colectomy or proctocolectomy with an ileoanal pouch and placement of an absorbable mesh. The primary endpoint was the annual rate of prophylactic parastomal mesh placement following rectal resection without sphincter preservation in Germany. Cases reporting both nonabsorbable mesh placement and rectal resection without sphincter preservation were considered prophylactic mesh insertions.

**Results:**
A total of 41,697 patients received a rectal resection without sphincter preservation and without nonabsorbable mesh placement. Among these individuals 27,089 were male and 14,608 were female. The rate of reoperations (3.1%) and the length of hospital stay (25.3 days ±19.32) remained almost constant during these 10 years. The rate of prophylactic mesh placement was increasing from 0.2% (n=8) in 2010 to 6.4 % (n=198) in 2020.

**Conclusion:**
Currently, only the minority of patients who have undergone rectal resection without sphincter preservation receive prophylactic mesh insertion.

**Minimal Invasive Approach for Repairing Perineal Hernia: How we do it**
ID: 905

Sarah Kalmbach[1], Christoph Steidle[1], Andre Mihaljevic[1], Robert Bachmann[1]

[1]*Klinik für Allgemeine, Viszeral- und Transplantationschirurgie , Hoppe-Seyler-Straße 3, Tübingen, Deutschland*

**Background:**
One rare but challenging complication associated with both abdominoperineal excision and extralevator abdominoperineal excision is the development of perineal hernias [1]. These hernias involve the protrusion of abdominal contents through weakened tissues or surgical defects in the perineal area.
The incidence of perineal hernia after abdominoperineal excision or extralevator abdominoperineal excision varies significantly, ranging from 1% to 26% [2]. A higher incidence of postoperative perineal hernias is associated with the more radical extralevator abdominoperineal excision procedure.
Managing symptomatic perineal hernias post-surgery presents a formidable challenge. The recurrence rate following surgical repair is high, primarily due to difficulties in securely fixing prosthetic materials, and there is no widely accepted standard for the best repair method.
The repair of these hernias can be carried out through various approaches, including either abdominal, perineal or combined methods, and may involve open or laparoscopic techniques, as well as options such as primary suture closure, mesh placement, or myocutaneous flaps [1], [3]
The latest studies also explore prophylactic techniques to prevent the development of postoperative perineal hernias [4].

The aim of this case presentation, in addition to demonstrating the surgical procedure, was to analyze the intraoperative and postoperative course.

**Materials and methods:**
This case report retrospectively analyzes a patient who underwent laparoscopic abdominal repair for a incarcerated perineal hernia, considering clinical and demographic factors, surgical duration, postoperative complications, and hospital stay duration.

**Results:**
This concerned an 81-year-old female patient who underwent a combined minimally invasive (DaVinci Xi robot-assisted) and open abdominoperineal resection with extralevator dissection following HOLM, along with the creation of an end colostomy, after neoadjuvant pre-treatment, due to rectal carcinoma involving the sphincter. Thirteen months later, the patient presented urgently with symptoms of bowel obstruction and abdominal pain. The cause of her complaints was identified as an incarcerated perineal hernia with a hernia orifice measuring 11.4x5.7 cm, and the herniation of small bowel loops. Due to the presentation of a mechanical ileus, the decision was made to perform an emergency exploratory laparoscopy.

In this surgical procedure, the patient is positioned in a lithotomy position after inserting a urinary bladder catheter. After establishing laparoscopic access, a head-down (Trendelenburg) position was assumed. This positioning allows for the subsequent mobilization of the herniated small bowel loops, and the hernia is visualized from an abdominal perspective. To close the hernia orifice, a 15 x 15 Mesh Dynamesh IPOM is used, which is placed with adequate overlap of the hernia orifice edges and with the anti-adhesive side facing the visceral side. Fixation is performed at the level of the sacrum bone and the lateral pelvic wall using Secure Straps Tackers, while avoiding the vascular structures (internal iliac artery) and nerves. The remaining fixation is achieved with fibrin glue on the uterine posterior wall.
The surgical procedure lasted for 1 hour and 34 minutes. The patient recovered quickly from the intervention and was able to leave the clinic after 3 days. During the clinical follow-up 6 months after the procedure, the patient reported feeling well. Additionally, in the imaging conducted as part of oncological surveillance 11 months after the procedure, no recurrence was observed.

**Conclusion:**
In summary, we conclude that laparoscopic hernia repair is a safe and feasible procedure that can be performed within a reasonable timeframe. Postoperatively, it is associated with a short hospital stay and minimal pain, allowing for a swift return to daily activities.

[1] J. Li and W. Zhang, "How we do it: repair of large perineal hernia after abdominoperineal resection," *Hernia*, vol. 21, no. 6, pp. 957–961, Dec. 2017, doi: 10.1007/S10029-017-1685-9/FIGURES/2.

[2] A. Balla *et al.*, "Perineal hernia repair after abdominoperineal excision or extralevator abdominoperineal excision: a systematic review of the literature," *Tech Coloproctol*, vol. 21, no. 5, pp. 329–336, May 2017, doi: 10.1007/S10151-017-1634-8/TABLES/4.

[3] A. M. Samar and G. Branagan, "Perineal hernia repair after extralevator abdominoperineal excision, how we do it (PERineal Laparoscopic Sling: PERLS Technique)," *Langenbecks Arch Surg*, vol. 407, no. 5, pp. 2187–2191, Aug. 2022, doi: 10.1007/S00423-022-02457-0/TABLES/4.

[4] Y. Shen *et al.*, "Closure of pelvic peritoneum with bladder peritoneum flap reconstruction after laparoscopic extralevator abdominoperineal excision: A prospective stage II study," *J Surg Oncol*, vol. 128, no. 5, pp. 851–859, Oct. 2023, doi: 10.1002/JSO.27382.

### Risk factors for post-operative Ileus in patients with anterior resection for rectal cancer.

ID: 12

Dimitrios Prassas[1 2], Dinah Hallmann[3], Wolfram Trudo Knoefel[2]

[1]*Katholisches Klinikum Essen, Klinik für Allgemein- und Viszeralchirurgie, Essen, Deutschland*,
[2]*Uniklinik Düsseldorf, Klinik für Allgemein-, Viszeral- und Kinderchirurgie, Düsseldorf, Deutschland*,
[3]*St. Marien-Hospital Mülheim an der Ruhr, Department of Surgery, Mülheim an der Ruhr, Deutschland*

**Background:**

Postoperative Ileus (POI) is a major cause of morbidity in patients undergoing colorectal surgery. Aim of our study was to evaluate potential risk factors for POI in cases with anterior resection for rectal cancer.

**Materials and methods:**

A retrospective cohort study was performed on patients that underwent open anterior resection for rectal cancer between 2004 and 2018 at a single tertiary referral center. Uni- and multivariate analysis was performed to identify potential risk factors for POI.

**Results:**

A total of 136 patients were included. POI was observed in 18 patients (13.2%). Advanced age was a statistically significant risk factor both in the uni- as well as in the multivariate analysis. An increase in age by each one year was found to increase the odds of POI by 5% [95%CI: 0.4%-9.7%; p=0.032].

**Conclusion:**

Increased age was identified as a non-modifiable, patient-related risk factor for POI after anterior resection for rectal cancer. This finding is of particular importance as it turns the focus on the elderly patient and underlines the need to closely monitor this patient subgroup postoperatively

Abstracts – DCK 2024 – 141st Congress of the German Society of Surgery – Leipzig, April 24–26 • DOI 10.1515/iss-2024-2003
Innov Surg Sci 2024; 9 (Special Suppl 1): s18–s203

**Use of a novel digital leakage notification system (Heylo™) for ostomy care results in an increase of patient QoL by lowering the mental burden of leackage. The ASSISTER Trial, a Randomized Controlled Cross-over study showing positive healthcare effects.**
ID: 44

Peter Ambe[1], Amrei Steinhoff[2], Elin Brunckhorst[2], Martin Vestergaard[3], Teresa Adeltoft Ajslev[3]

[1]*Universität Witten / Herdecke, Witten, Deutschland*, [2]*Coloplast GmbH, Medical Affairs, Hamburg, Deutschland*, [3]*Coloplast A/S, Humlebaek*

**Background:**
Digital health wearables with sensor technologies are emerging and the prospect of potential benefits for patients and the healthcare system are significant.
Living with a stoma comes along with patients' impairments in daily life. Up to 92% of these patients worry about leakage. Leakage has both mental and physical consequences, such as fear of leaving the house and thus inability to work, impaired sleep and peristomal skin complications. A direct negative association exists between the numbers of leakages experienced each week, month, or year and reported Quality of Life (QoL).
The purpose of the present study was to investigate care effects of a novel digital leakage notification system (Heylo™) in ostomy care.

**Materials and methods:**
A randomized, controlled, open-labeled, cross-over trial was conducted in Germany. Subjects were randomized to either Heylo™ or *Standard of Care* (*SoC*) treatment for 8 weeks, whereafter subjects crossed over to the opposite treatment for an additional 8 weeks. Primary and secondary endpoints were: Emotional impact score (0-100) and participation in society score (0-100), measured by the Ostomy Leakage Impact (OLI) and WHO disability assessment tools.
Data were summarized by mean (SD) and analyzed using paired generalized mixed modelling taking time and study period into account.

**Results:**
The intention-to-treat population constituted of 139 adult subjects (age 50.7±13.5 years), 63% had an ileostomy and 51% were females. Average time since stoma creation was 6.1 years, including 22% (n=31) with a stoma for <3 months. After 8 weeks use of Heylo™ both the emotional impact score and participation in society changed significantly (with Δ11.4, p<0.001 and Δ4.2, p=0.001, respectively), reflecting improved emotional health and better ability to participate in society.

**Conclusion:**
Use of the novel digital leakage notification system demonstrated positive healthcare effects with improved QoL and less burden of living with a stoma, reflected as less embarrassment, living with dignity and better capability to work, or participating in society and interacting with people.

### Prospective evaluation of the (multi)-resistant pathogen spectrum in acute appendicitis: Impact on clinical outcomes

ID: 126

Faruk Koca[1], Sara Fatima Faqar-Uz-Zaman[1], Michael Hogardt[2], Volkhard Kempf[2], Wolf Otto Bechstein[1], Patrizia Malkomes[1]

[1]*Goethe-Universität Frankfurt, Universitätsklinikum, Klinik für Allgemein-, Viszeral-, Transplantations- und Thoraxchirurgie, Frankfurt am Main, Deutschland*, [2]*Goethe-Universität Frankfurt, Universitätsklinikum, Institut für Medizinische Mikrobiologie und Krankenhaushygiene, Frankfurt am Main, Deutschland*

**Background:**
Bacterial infections are a significant factor in postoperative morbidity following appendectomy. A previous retrospective study suggested that multidrug-resistant organisms (MDRO) may play a crucial role in the course of disease. However, there is currently a lack of prospective data to substantiate these findings. Thus, the aim of this prospective study was to determine the bacterial spectrum present in appendiceal and rectal swabs from patients with acute appendicitis in order to facilitate effective treatment. The study was registered in the German Clinical Trials Registry Platform under the identifier DRKS00028610.

**Materials and methods:**
We prospectively enrolled all adult patients with acute appendicitis who underwent surgical treatment at our hospital between April 2022 and July 2023. The appendiceal bacterial spectrum and MDRO frequency in rectal swabs were analysed using state of the art microbiology diagnostics. Clinical data were extracted from electronic medical records to assess correlation of bacterial spectrum with incidence of surgical side infections, postoperative complications, duration and type of antibiotic therapy. A telephone follow-up was performed 30 days after surgery.

**Results:**
A total of 105 patients were included. In 29 (27.6%) of the patients, pathogens with multiple antibiotic resistance were found in the appendix swab. Among them, six patients showed the presence of MDROs according to the CDC (Centers for Disease Control and Prevention) in their appendix swabs, while 11.4% of all patients were tested positive for MDROs in rectal swabs, including 10 *Escherichia* coli, one *Pseudomonas aeruginosa* and one *Enterococcus* sp..
The subgroup of patients with multiple antibiotic resistance had a significantly higher incidence of postoperative complications than those without (31% vs. 10.5%, p=0.017). Specifically, there were more Clavien-Dindo grade 3 complications (17.2% vs. 2.6%, p=0.007), a higher incidence of deep wound infections (CDC grade A3) (10.3% vs. 1.3%, p=0.028), a greater need for postoperative antibiotic therapy (65.5% vs. 40.8%, p=0.029), and a greater need to escalate antibiotic therapy (37.9% vs. 15.8%, p=0.019). Furthermore, the group with multiple antibiotic resistance had a longer hospital stay, with a mean of 5.83 days compared with 4.26 days in the other group (p=0.028). Similar, the duration of antibiotic therapy was significantly longer in the group with multidrug resistance (mean in days: 5.38 vs. 3.47, p = 0.008).

**Conclusion:**
Our prospective study confirms the impact of multi-drug resistant pathogens on post-appendectomy morbidity, as their presence significantly increases the risk of complications, prolonged hospital stay and the need for prolonged antibiotic therapy.

### Spinal anesthesia improves postoperative pain control after colorectal surgery: preliminary results of a prospective study

ID: 159

Michael Frey[1], Andrea Wirsching[2], Anne Seidl[2], Isabelle Obrecht[2], Simone Hasler-Gehrer[2], Andreas Keerl[2], Andrea Kopp-Lugli[2], Michael Heesen[2], Antonio Nocito[2]

[1]*Universitätsspital Zürich, Klinik für Viszeral- und Transplantationschirurgie, Zürich, Schweiz*,
[2]*Kantonsspital Baden, Klinik für Allgemein-, Viszeral-, Thorax- und Gefässchirurgie, Baden, Schweiz*

**Background:**
Spinal anaesthesia as an adjunct to general anaesthesia has been shown to improve perioperative pain management after colorectal surgery. A subsequent reduction of opioid analgesics might further shorten postoperative ileus and costs in colorectal surgery. We aim to assess the impact of spinal anaesthesia on pain control, postoperative ileus and costs after colorectal surgery.

**Materials and methods:**
Data of consecutive patients with colorectal resections not receiving an ostomy between October 1$^{st}$ 2021 and July 31$^{st}$ 2022 were entered into a prospective database and included in the preliminary analysis. Peri- and postoperative pain scores (numeric rating scale, NRS) and –medication, parameters of bowel function (flatus, stool, diet) and costs were compared. Prolonged postoperative ileus was previously defined by inability to tolerate a regular diet, radiological ileus and/ or absence of flatus for 24 hours after the 4$^{th}$ postoperative day.

**Results:**
Spinal anaesthesia was added to general anaesthesia in 53 of 88 patients included in our preliminary analysis. Patient age and body mass index were similarly distributed between groups. Comorbidity (ASA class 3 and 4) was more frequent in patients not receiving spinal anaesthesia (51% vs. 21%. p=0.003). Spinal anaesthesia was associated with decreased NRS scores at 3h (0 vs 4, p= 0.019), 12h (2 vs. 4, p=0.007) and 24h (2 vs. 5, p=0.002) following surgery. Our preliminary analysis of 88 patients (50% of sample size calculation) did not show any difference in time-to-flatus, time-to-stool, time-to-normal diet and prolonged postoperative ileus between the two groups. Cost analysis of 34 patients operated in 2021 did not show a difference between patients with and without spinal anaesthesia (20'227 CHF vs. 20'876CHF).

**Conclusion:**
Spinal anaesthesia improves postoperative pain control. Our preliminary analysis did not show a decrease of postoperative ileus and costs after colorectal resection.

**Damage Control Surgery Reduces Stoma Rate in Perforated Diverticulitis – an International, Retrospective Multicenter Analysis**
ID: 361

Paul Ritschl[1], Valentin Ritschl[2], Dario Tartaglia[3], Antonio Brillantino[4], Mia Kim[5], Johannes Lauscher[6], Igors Iesalnieks[7], Ayman Agha[8], Alexander Perathoner[9], Elisabeth Gasser[9], Jaroslav Presl[10], Ramin-Raul Ossami-Saidy[1 11], Johann Pratschke[1], Maximilian Sohn[12]

[1]*Chirurgische Klinik Campus Charité Mitte/ Campus Virchow Klinikum, Charité - Universitätsmedizin Berlin, Berlin, Deutschland*, [2]*Medizinische Universität Wien, Section for Outcomes Research CeMSIIS – Center for Medical Statistics, Informatics, and Intelligent Systems, Wien, Österreich*, [3]*Hospital Cisanello Pisa, Pisa, Italy*, [4]*Hospital Antonio Cardarelli - Emergency Room, Napoli, Italy*, [5]*München Klinik Harlaching, München, Deutschland*, [6]*Charité Campus Benjamin Franklin, Klinik für Allgemein- und Viszeralchirurgie | CBF , Berlin, Deutschland*, [7]*Evangelisches Krankenhaus Kalk, Köln, Deutschland*, [8]*München Klinik Bogenhausen, München, Deutschland*, [9]*Medizinische Universität Innsbruck., Innsbruck, Österreich*, [10]*Landeskrankenhaus Salzburg - Universitätsklinikum der PMU, Salzburg, Österreich*, [11]*Krankenhaus Waldfriede, Berlin, Deutschland*, [12]*Isar Klinikum, Chirurgie, München, Deutschland*

**Background:**
Perforated diverticulitis represents a common reason for emergency surgery. Sigmoid resection followed by a primary anastomosis (PRA) in combination with a loop-ileostomy, Hartmann's procedure (HP; for septic, instable patients), laparoscopic lavage or damage control surgery (DCS) can be performed. DCS comprises a two-stage concept with an initial resection of the perforated colon segment with oral and aboral blind closure followed by a definitive reconstruction at scheduled second laparotomy with either anastomosis (± loop ileostomy) or a Hartmann's procedure after 24-48 h. The aim of this study was to evaluate real world outcome in 9 different hospitals of which 5 used the DCS concept.

**Materials and methods:**
Nine hospitals from Germany, Italy or Austria (5 DCS; 4 non-DCS) participated in this study. All consecutive patients were included between January 1st 2014 (and if DCS group: starting when the first DCS was performed) and September 30th 2020. Primary outcome was ostomy rate at the time of hospital discharge. The study is registered within the clinicaltrials.gov register: NCT04220840.

**Results:**
A total of 558 patients were included in the final analysis with 193 being treated in non-DCS hospitals. There was no significant difference in regard to the prevalence of feculent peritonitis (Hinchey 4), sex, active malignancy or medical immunosuppression. However, patients in the non-DCS group were significant older (non-DCS 70,2 ± 13,8 vs DCS 66,9 ± 13,9; p= 0,007). On the other side patients in DCS hospitals had significant worse “Mannheim Peritonitis Index” (non-DCS 20.4 ± 8,0 vs DCS 22,3 ± 8,0; p=0,008). Patients in non-DCS hospitals received in 58% a HP, in 20,2% PRA without diverting ileostomy and in 12,4% PRA with diverting ileostomy. Due to complications some patients with primary anastomosis underwent additional ostomy formation, thus finally 61,5% of all patients left the hospital with end-colostomy and 17,7% with ileostomy. In the DCS hospitals 75% of all patients were treated with DCS and 15,9% received primary HP. Of all DCS patients, 29,3% received a secondary HP, 53,6% got an anastomosis without ileostomy and 15,6% received a secondary anastomosis with an ileostomy resulting in a 54% ostomy rate. The difference on ostomy rate between DCS and non-DCS hospitals was statistically significant (non-DCS 20,8% vs DCS 45,6%; p<0,001).
Other secondary outcomes as the incidence of complications, including patient death was comparable between the 2 investigated groups. In addition, the length of stay (hospital as well as ICU stay) was not significant different between the groups. Due to the nature of a 2-step procedure patients in the DCS group had significant more surgeries during the hospital stay (non-DCS 1,51 ± 1,10 vs DCS 2,63 ± 1,89; p<0.001).

**Conclusion:**

Although international guidelines meanwhile recommend PRA as a standard approach, we could identify HP to be still applied in a relevant percentage in perforated diverticulitis. Consequent use of DCS leads not only to a significant reduction of HP, but also to a significant decrease of the overall stoma rate. The formation of enterostomies is associated with a relevant rate of complications such as dehydration, renal failure, prolapse, and parastomal hernia. Moreover, additional surgery is necessary for stoma reversal. Thereby, DCS potentially can reduce the long-term complication rate, the need for rehospitalization and interval-reoperation and by this, supports an improved quality of life.

Abstracts – DCK 2024 – 141st Congress of the German Society of Surgery – Leipzig, April 24–26 • DOI 10.1515/iss-2024-2003
Innov Surg Sci 2024; 9 (Special Suppl 1): s18–s203

**Lymphatic Mapping in Colon Cancer Depending on Injection Time and Tracing Agent: A Systematic Review and Meta-Analysis of Prospective Designed Studies**
ID: 383

Katharina Lucas[1], Nathaniel Melling[1], Anastasios Giannou[1], Matthias Reeh[1], Oliver Mann[1], Thilo Hackert[1], Jakob Izbicki[1], Daniel Perez[1], Julia-Kristin Graß[1]

[1]*Klinik für Allgemein-, Viszeral- und Thoraxchirurgie - Universitätsklinikum Hamburg-Eppendorf - UKE, Hamburg, Deutschland*

**Background:**
An optimized lymph node yield leads to better survival in colon cancer, but extended lymphadenectomy is not associated with survival benefits. Lymphatic mapping shows several colon cancers feature aberrant drainage pathways inducing local recurrence when not resected. Currently, different protocols exist for lymphatic mapping procedures. This meta-analysis assessed which protocol has the best capacity to detect tumor-draining and possibly metastatic lymph nodes.

**Materials and methods:**
A systematic review was conducted according to PRISMA guidelines, including prospective trials with in vivo tracer application. The risk of bias was evaluated using the QUADAS-2 tool. Traced lymph nodes, total resected lymph nodes, and aberrant drainage detection rate were analyzed.

**Results:**
Fifty-eight studies met the inclusion criteria, of which 42 searched for aberrant drainage. While a preoperative tracer injection significantly increased the traced lymph node rates compared to intraoperative tracing (30.1% (15.4, 47.3) vs. 14.1% (11.9, 16.5), $p = 0.03$), no effect was shown for the tracer used ($p = 0.740$) or the application sites comparing submucosal and subserosal injection (22.9% (14.1, 33.1) vs. 14.3% (12.1, 16.8), $p = 0.07$). Preoperative tracer injection resulted in a significantly higher rate of detected aberrant lymph nodes compared to intraoperative injection (26.3% [95% CI 11.5, 44.0] vs. 2.5% [95% CI 0.8, 4.7], $p < 0.001$). Analyzing 112 individual patient datasets from eight studies revealed a significant impact on aberrant drainage detection for injection timing, favoring preoperative over intraoperative injection (OR 0.050 [95% CI 0.010–0.176], $p < 0.001$) while indocyanine green presented itself as the superior tracer (OR 0.127 [95% CI 0.018–0.528], $p = 0.012$).

**Conclusion:**
Optimized lymphatic mapping techniques result in significantly higher detection of aberrant lymphatic drainage patterns and thus enable a personalized approach to reducing local recurrence.

**Is the POLARS tool suitable for predicting low-anterior resection syndrome (LARS) after rectal resection ?**
ID: 536

Katharina Schwenker[1], Frederike Franke[1], Matthias Martin[1], Thomas Becker[1], Julius Pochhammer[1]

[1]*Klinik für Allgemeine, Viszeral-, Thorax-, Transplantations- und Kinderchirurgie, UKSH, Campus Kiel, Kiel, Deutschland*

**Background:**
Radical resection of rectal cancer can lead to persistent impairment of bowel function, known as low anterior resection syndrome (LARS), which can significantly affect quality of life. In the last two years, several nomograms have been published for predictive purposes, and radiological methods are also included [1, 2, 3]. Nevertheless, it is still unclear how reliably the risk of developing LARS after surgery can be determined. First published in 2018, the POLARS tool was designed to preoperatively predict the occurrence and severity of LARS in patients with rectal cancer [4]. The aim of this study was to evaluate the accuracy of POLARS in predicting the occurrence of LARS.

**Materials and methods:**
A total of 119 patients with rectal cancer who underwent robotic or laparoscopic rectal resection with continuity restoration surgery between 6/2013 and 4/2022, were stoma-free, recurrence-free, and alive were contacted by letter and telephone for this retrospective study. Of these, 84 patients answered the specific LARS questionnaire to assess bowel function [5]. The POLARS nomogram was used to calculate the predictive value for the occurrence of LARS. Subsequently, the predicted and actual values were compared.

**Results:**
Study participants were 27 women (32.1%) and 57 men (67.9%) with a median age at surgery of 61.1 years (standard deviation: 10.6 years). The median tumor height was 10 cm (min 3- max 18 cm), 38 patients received neoadjuvant therapy (45.2%), of these 25 (29.8%) received complete radiochemotherapy. Treated by low anterior resection with total mesorectal excision were 71 (84.5%), by robotic procedure 70 (83.3%), 64 (76.2%) received a protective ostomy. Follow-up was at median 50 months (13 - 150) after surgery. LARS score was at median 29 points (0-41) and did not correlate with the time of follow-up. Consequently, 22 patients (26.2%) had no LARS, 22 (26.2%) had "minor" LARS, and 40 (47.6%) had "major" LARS. The median POLARS score was 27 (17-24) points. Predicted was no LARS in 5 pat. (6.0%), a "minor" LARS in 27 (32.1%), a "major" LARS in 52 (61.9%). The grade of LARS was correctly predicted in 22 patents (26.2%), underestimated in 31 (36.9%), and overestimated in 31 (36.9%). Thus, the positive likelihood ratio for a "major" LARS was 0.55, the negative likelihood ratio 1.31, and the test accuracy 41.7%.

**Conclusion:**
LARS is a common sequela after rectal resection. The frequencies for the various manifestations of LARS in the population studied are consistent with those of other published data [6]. The POLARS score, which can be collected preoperatively, was found to be too inaccurate in predicting the risk and severity of LARS to perform a risk assessment with patients preoperatively. It remains to be tested and seen whether the prediction models published in the past two years perform better in external validations.

1. Paku M, Miyoshi N, Fujino S, Hata T, Ogino T, Takahashi H, Uemura M, Mizushima T, Yamamoto H, Doki Y, Eguchi H. Development and evaluation of a Japanese prediction model for low anterior resection syndrome after rectal cancer surgery. BMC Gastroenterol. 2022 May 13;22(1):239. doi: 10.1186/s12876-022-02295-w. PMID: 35562665; PMCID: PMC9102936.
2. Qin Q, Huang B, Wu A, Gao J, Liu X, Cao W, Ma T, Kuang Y, Guo J, Wu Q, Shao B, Guan Q, Yao H, Zhang X, Wang H; Chinese Radiation Intestinal Injury Research Group. Development and

Validation of a Post-Radiotherapy Prediction Model for Bowel Dysfunction After Rectal Cancer Resection. Gastroenterology. 2023 Aug 23:S0016-5085(23)04905-3. doi: 10.1053/j.gastro.2023.08.022. Epub ahead of print. PMID: 37625498.

3. Mori R, Uemura M, Tsuboyama T, Fujino S, Hata T, Ogino T, Takahashi H, Miyoshi N, Mizushima T, Doki Y, Eguchi H. The prediction of postoperative anorectal dysfunction after low anterior resection for lower rectal cancer by measuring the volume of defecation-related muscles. Surg Today. 2022 May;52(5):745-754. doi: 10.1007/s00595-022-02478-8. Epub 2022 Mar 24. PMID: 35322295.

4. Battersby NJ, Bouliotis G, Emmertsen KJ, Juul T, Glynne-Jones R, Branagan G, Christensen P, Laurberg S, Moran BJ; UK and Danish LARS Study Groups. Development and external validation of a nomogram and online tool to predict bowel dysfunction following restorative rectal cancer resection: the POLARS score. Gut. 2018 Apr;67(4):688-696. doi: 10.1136/gutjnl-2016-312695. Epub 2017 Jan 23. PMID: 28115491.

5. Bregendahl S, Emmertsen KJ, Lous J, Laurberg S. Bowel dysfunction after low anterior resection with and without neoadjuvant therapy for rectal cancer: a population-based cross-sectional study. Colorectal Dis. 2013 Sep;15(9):1130-9. doi: 10.1111/codi.12244. PMID: 23581977.

6. Bolton WS, Chapman SJ, Corrigan N, Croft J, Collinson F, Brown JM, Jayne DG. The Incidence of Low Anterior Resection Syndrome as Assessed in an International Randomized Controlled Trial (MRC/NIHR ROLARR). Ann Surg. 2021 Dec 1;274(6):e1223-e1229. doi: 10.1097/SLA.0000000000003806. PMID: 32097165

**The influence of metabolic factors on early-onset colorectal carcinoma: A systematic review and meta-analysis**
ID: 572

Jacques Hilbert[1], Fernando Topfstedt[1], Stephan Herrmann[1], Claudia Läßle[1], Hannes P. Neeff[1], Stephan Fichtner-Feigl[1], Gabriel Seifert[1]

[1]*Department of General and Visceral Surgery, Medical Center, University of Freiburg*

**Background:**
Colorectal cancer (CRC) is the third most common cancer and the second leading cause of cancer-related death. Early-onset (eoCRC) and late-onset colorectal carcinoma (loCRC) are distinguished based on the age at diagnosis, most commonly at age 50. Despite declining rates of loCRC, there has been an alarming increase in eoCRC incidence in many countries, including the US, China, Japan, and several European nations. The underpinning mechanisms remain unclear, with genetic, environmental, and lifestyle factors likely playing a significant role.
Patients with eoCRC often present with more advanced, poorly differentiated stages, and distal tumor location. Existing CRC screening programs are designed to detect loCRC, leaving eoCRC cases underdiagnosed. Approximately one-third of eoCRC cases have a hereditary or familial link, while the majority are sporadic. Understanding the factors driving earlier CRC incidence and identifying metabolic risk factors could aid in developing tailored screening strategies and raising public awareness.
Quantifying the association of metabolic risk factors for eoCRC endpoints is extremely challenging and requires high-quality data. The prevalence of metabolic comorbidities such as type 2 diabetes (T2D) and obesity is significantly higher in patients with eoCRC compared to loCRC patients.
This systematic review and meta-analysis aim to consolidate the current evidence concerning the independent associations of metabolic disorders with the incidence rate, stage at diagnosis, and mortality in patients with eoCRC.

**Materials and methods:**
We systematically searched for articles in Medline and Web of Science databases, selecting 23 studies that met our inclusion criteria. We excluded studies that did not adjust for age or did not include at least 50 early-onset CRC cases or did not test the association of metabolic risk factors with CRC-specific outcome measures. We did not exclude studies that provided descriptive results only and stratification on age and metabolic factors. These studies provided valuable insights into the associations of metabolic risk factors with eoCRC incidence, age at diagnosis, and prognosis. We analyzed data from diverse study designs, including cohort studies, case-control studies, and cross-sectional studies, and assessed evidence levels using the GRADE approach.

**Results:**
Obesity: Our meta-analysis unveiled compelling evidence indicating that obesity at the time of CRC diagnosis and obesity during late adolescence are robustly linked to a heightened risk of eoCRC. The summary relative risk (RR) for obesity at diagnosis was 1.77 (95%CI: 1.40-2.23), underscoring the significant association between obesity and eoCRC. Notably, we observed that a higher BMI did not significantly impact disease-free survival in individuals aged 40-49 years with eoCRC, although it is important to acknowledge that this estimate was not adjusted for potential confounding factors such as family history of CRC or inflammatory bowel disease (IBD).
Type 2 Diabetes (T2D): Our analysis identified T2D at the time of CRC diagnosis as an independent risk factor for eoCRC. Notably, this association was particularly pronounced in males, with a compelling link between early-onset T2D and increased eoCRC-incidence. Moreover, we found that patients with T2D exhibited a 10-year-cumulative risk of CRC-incidence akin to that of the general population at age 50, approximately 4 to 5 years earlier.
Hyperlipidemia (HLD): Our review of studies investigating HLD and eoCRC revealed heterogeneous results. While some studies reported a significant association, particularly in individuals aged 20-39

years, conflicting findings were observed. These discrepancies may be attributed to differences in study design, different definitions of HLD, and diverse adjustments for potential confounders. Furthermore, our analysis indicated that HLD diagnosed five years or more prior to eoCRC diagnosis was independently associated with shorter long-term survival.
Arterial Hypertension (HTN): Our findings indicated that HTN represents an independent risk factor for eoCRC incidence, with the strongest association observed in males aged 40-49 years. Nevertheless, the relationship between HTN and eoCRC varied by age and sex, with weaker associations in females and no significant associations for patients aged 20-29 or 50-59 years. However, it is essential to acknowledge that not all studies replicated these findings.
Metabolic Syndrome (MetS): Our systematic review included a single study assessing the role of MetS in eoCRC incidence, utilizing the International Diabetes Federation (IDF) criteria for MetS definition. This study reported a significant association between MetS and a higher incidence of eoCRC, especially in male sex. The presence of more than one metabolic comorbidity was associated with worse long-term survival, particularly in females. These findings underscore the importance of considering MetS as a potential driver of eoCRC and emphasize the need for tailored risk assessment and preventive strategies targeting individuals with metabolic risk factors.

**Conclusion:**
In conclusion, our systematic review and meta-analysis shed light on the complex interplay between metabolic risk factors and early-onset colorectal cancer (eoCRC). Robust evidence supports the independent associations of obesity, T2D, and, to some extent, hypertension and hyperlipidemia, with eoCRC incidence. Obesity, particularly during late adolescence and early adulthood, emerges as a significant risk factor for eoCRC, with weight gain further exacerbating this risk. Early-onset T2D is linked to an earlier cumulative risk of CRC incidence compared to the general population, with a particularly strong association in males.
Hypertension displays gender- and age-specific associations with eoCRC, with a more pronounced effect in males and patients aged 40-49 years. However, not all studies corroborate these findings, indicating potential variations based on factors such as hypertension stage, gender, and age. The evidence for hyperlipidemia remains inconclusive, with potential associations, particularly in younger individuals, warranting further investigation. Metabolic syndrome appears to elevate the risk of eoCRC, especially among males, and the presence of multiple metabolic comorbidities is linked to poorer long-term survival, predominantly in females.
These findings underscore the clinical relevance of metabolic risk factors as potential drivers of eoCRC, informing future risk assessment and preventive strategies. Specific screening programs and interventions targeting individuals with metabolic risk factors may be essential in addressing the rising incidence of eoCRC.
However, further research is imperative to elucidate the underlying mechanisms and develop more refined risk prediction models, ultimately improving clinical management and outcomes for this growing patient population.

**NOTCH pathway inhibition increases radiosensitivity in a context-dependent manner**
ID: 604

Hannah Flebbe[1], Melanie Spitzner[1], Carolin Schneider[1], Lukas Krauss[1], Michael Ghadimi[1], Günter Schneider[1], Marian Grade[1]

[1]*Universitätsmedizin Göttingen, Allgemein-, Viszeral- und Kinderchirurgie, Göttingen, Deutschland*

**Background:**
Preoperative chemoradiotherapy followed by surgical resection represents a standard treatment for locally advanced rectal cancer. We recently demonstrated that activation of the NOTCH signaling pathway increases treatment resistance. In the present study, our aim was to evaluate the effect of inhibitors of the NOTCH-signaling pathway on (chemo-) radiosensitivity.

**Materials and methods:**
Two rectal cancer cell lines (SW1463 and SW837) with different modes of NOTCH activation were either treated with LY3039478, a y-secretase-inhibitor, or RIN1, which targets RBPJ, the key downstream transcription factor of the NOTCH pathway. Subsequently, colony formation, cell cycle analysis and RNA-seq was performed.

**Results:**
The effects of NOTCH pathway inhibition were different between SW837 and SW1463. While treatment with 5 µM LY3039478 resulted in sensitization of SW837, but not SW1463, 2.5 µM RIN1 increased sensitivity to chemoradiotherapy in both cell lines. RIN1 induced a G2-arrest 24 hours after irradiation in SW837, while there was a significant decrease of cells in the G1-phase after treatment with RIN1 in SW1463. To define pathways regulated by the NOTCH pathway inhibitors, RNA-seq data were analyzed. Interestingly, substantially more genes were significantly up- or downregulated in SW1463 compared to SW837 with only minimally overlapping genes. Interestingly, however, GSEA analysis revealed that similar pathways were deregulated in both cell lines.

**Conclusion:**
These results indicate that inhibitors of the NOTCH signaling pathway may play a role in sensitizing rectal cancer cells to irradiation therapy, which should be further evaluated.

**Avoidance of temporary ileostomies by virtual ileostomy creation ("ghost stoma") in appropriate patients with rectal cancer and perforated or complicated sigmoid diverticulitis**
ID: 761

Reiko Wießner[1], Alexander Gehring[1], Claudia Tolla-Jensen[1], Tina Görl[1]

[1]*Ribnitz-Damgarten, Bodden-Kliniken Ribnitz-Damgarten GmbH, Ribnitz-Damgarten, Deutschland*

**Background:**
In 2007, an Italian study presented for the first time the "virtual" placement of an ileostomy in patients with rectal cancer. Since then, several studies, some of them randomized controlled, have been published with impressive results. In 2018, we introduced the placement of virtual ileostomies in our hospital for rectal cancer in appropriate patients and expanded the indication for perforated and complicated sigmoid diverticulitis.

**Materials and methods:**
Since May 2018, virtual ileostomatas have been created in a total of 10 women and 3 men. Five patients were indicated for rectal cancer. Four patients required emergency laparoscopic or conventional surgery for perforated sigmoid diverticulitis type 2c with putrid lower abdomen or four-quadrant peritonitis. Two patients had chronic recurrent diverticulitis type 3c with sigmoid vaginal fistula, interenteric fistula, or sigmoid stenosis, and two patients developed anastomotic insufficiency after laparoscopic sigmoid resection on the 6th and 7th p. o. day, respectively, leading to laparoscopic resection with creation of a virtual stoma.

**Results:**
The average age of our patients was 68.6 years (range 32 - 97 years) with ASA values between 2 - 4. Hospital stay averaged 16 days (range 8 - 49 days). Anastomotic height after sigmoid resection was on average 10 cm a. a. (range 6 - 13 cm a. a.) and in patients with rectal cancer was on average 5.6 cm (range 4 - 7 cm a. a.). In one patient (sigmoid diverticulitis type 3c, bronchial, urothelial, prostate carcinoma) the virtual stoma had to be converted into a "real" double ileostomy due to anastomotic insufficiency. This was closed 7 weeks later after secondary healing of the insufficiency. In two patients with rectal cancer, a real ileostoma had to be created once at late insufficiency (17th p. o. day after surgery and 10 days after discharge, anastomosis at 4 cm a. a.) and on the 4th p. o. day (anastomosis at 5 cm a. a.). The reins of the virtual stoma were removed in the remaining 11 patients on average on the 7th p. o. day.

**Conclusion:**
In addition to the indication for the creation of a virtual ileostoma in deep anterior rectal resection, complicated and perforated sigmoid diverticulitis with the development of peritonitis also represents an indication in special cases. Not only can the creation of a primary anastomosis avoid a discontinuity resection according to Hartmann in perforated sigmoid diverticulitis, but also the morbidity, mortality (fluid loss, electrolyte loss) as well as the quality of life (risk factor for LARS) caused by an ileostomy could be circumvented in 10 of 13 cases.

**Laparoscopic resection rectopexy significantly affects preexisting urinary symptoms in female patients.**
ID: 851

Luis Fernando Sangueza Quintanilla[1], Silvia Kraemer[1], Canan Ceran[1], Matthias Kraemer[1]

[1]*Allgemein- und Visceralchirurgie, St. Barbara-Klinik, Hamm, Deutschland*

**Background:**
This study aims to assess the effects of laparoscopic resection rectopexy on urinary symptoms.
It has previously been noted that following pelvic organ prolaps (POP) surgery, some patients report changes in urinary function. So far, not much is known about the extent of such changes. A possible influence of surgery in the posterior compartment on the anterior compartment is not known.

**Materials and methods:**
This is a prospective observational study with 100 consecutive female patients indicated for laparoscopic resection rectopexy. Stated urinary symptoms, pre- and postoperative "International Consultation on Incontinence Questionnaire – Urinary Incontinence Short Form" (ICIQ – UI SF), supplemented by a "quality of life "(QoL) visual analogue scale, and residual urine measurements (RUM) were compared and correlated.

**Results:**
Postoperative QoL was significantly improved, irrespective of preexisting urinary symptoms. Twenty-four (24%) patients noticed improved urinary function. This corresponded with 42% of 45 patients who had positive preoperative ICIQ scores indicating preexisting urinary symptoms. Conversely, 14 (14%) patients noticed a postoperative increase of urinary complaints. The stated symptom change was only in part reflected by changes of the ICIQ scoring. Comparing ICIQ, 19 (19%) patients scored "better" postoperatively against 8% scoring worse; 5 of the 8 patients experienced "de novo" symptoms. The improved postoperative ICIQ scoring was highly significant. RUM did not sufficiently correlate to symptoms/ICIQ for any meaningful conclusion.

**Conclusion:**
Laparoscopic resection rectopexy had predominantly beneficial and to a lesser extent detrimental effects on urinary symptoms. Effects were highly significant; they were mainly noted in patients with preexisting urinary complaints. So far, it is not possible to predict such effects on an individual basis. It remains questionable whether alternative surgeries for rectal prolapse have a similar effect on urinary incontinence. Without more factual knowledge and awareness about the extent of potential "collateral" effects of pelvic floor repair procedures, expert guidance of patients appears limited.

Abstracts – DCK 2024 – 141st Congress of the German Society of Surgery – Leipzig, April 24–26 • DOI 10.1515/iss-2024-2003
Innov Surg Sci 2024; 9 (Special Suppl 1): s18–s203

**Intramural versus percutaneous drainage placement: Multistate model for understanding objectifiable variables on clinical decision for interventional treatment of pancreatic fistula after left-sided pancreatectomy.**
ID: 792

Matthäus Felsenstein[1]

[1]*Charité Universitätsmedizin Berlin, Augustenberger Platz 1, Berlin*

**Background:**
Patients after left-sided pancreatectomies can experience postoperative pancreatic fistula (POPF) with fluid-collections with need of clinical intervention. Percutanous drainage placement may be effective but can prolong hospitalization and impact quality of life. Endoscopic intervention with installation of trans-gastric stents has been established as a versatile alternative. Following up on our experience with endoscopic treatment of retrogastric fluid collections, we aimed to define objectifiable measures for clinical decision making.

**Materials and methods:**
During the periode of 2018-2021 we evaluated 162 left-sided pancreatic resections at Charité-Unversitätsmedizin Berlin. We identified patients with biochemical leackage (BL), as well with clinically relevant pancreatic fistula (POPF B/C). Only patients with available CT-/MRI- scans were included and was defined date of diagnosis. Complication management included conservative treatment (antibiotics, irrigation of intraoperative drain), intervention treatment (percutaneous drain, endoscopic drain) and revision surgery. Success rates as well as clinical resolutions were compared. We employed a multi-state model (MSM) to compare interventional (percutaneous versus endoscopic drainage) methodologies and included clinical variable that could have impacted clinical decision making.

**Results:**
Majority of patients were operated for oncologic indications (84.7%). Surgical treatment included open (54.9%) or minimal-invasive surgery (42%). Clinically-relevant pancreatic fistula (POPF B/C) was detected in 64 patients (39.1%). Descriptive characterization via the step-up concept, revealed that individuals were treated via conservative treatment 37(35%), percutaneous 21(20%) or endoscopic treatment 43(41%), as well revision surgery 2(1.9%). We could show that size as well as location of the intraabdominal fluid collection impacted the decision rationale for endoscopic over percutaneous drainage placement. Accounting for volumetric measurements and location of the fluid collection revealed that endoscopic drainage placement significantly improved likelihood for entrance into a resolved state.

**Conclusion:**
The endoscopic intramural stent placement is a safe, feasible and efficient, interdisciplinary treatment modality after left-sided pamcreatectomies. Multi-state analyses revealed increased and earlier resolution of post-operative pancreatic fistula with retro-gastric fluid collections. Interdisciplinary concepts allow for reduced hospitalization and earlier admission for oncologic patients to undergo oncologic treatment.

**Cologne Ergonomic Measurement for Robotic Surgery (CEMRobSurg) using the Hugo™ RAS System**
ID: 152

Stefanie Brunner[1], Dolores Thea Müller[1], Rabi Raj Datta[1], Jennifer Eckhoff[1], Christian Storms[2], Seung-Hun Chon[1], Thomas Schmidt[1], Christiane Bruns[1], Hans Fuchs[1]

[1]*Department of General, Visceral, Cancer and Transplantat Surgery, University Hospital Cologne, Köln, Deutschland*, [2]*Faculty of Medicine, University of Cologne, Köln*

**Background:**
The ergonomic advantages and challenges of robotic surgery on the well-being of surgeons are mainly unexplored. The latest surgical robot available on the European market is the Hugo™ RAS System manufactured by Medtronic. This newly available system has four modular robot arms, facilitating procedural handling of the robot. The surgeon's console is open, allowing eye contact between the surgeon and the assistant at the operating table.
The aim of this study is to report the Cologne Ergonomic Measurement for Robotic Surgery (CEMRobSurg) using the Hugo™ RAS System in our training laboratory CeMIT.

**Materials and methods:**
The CEMRobSurg setup was developed in our Center and previously used to evaluate Ergonomics in esophageal cancer surgery (ERASE study) using the Da Vinci Surgical System. Four parameters were chosen in order to measure an ergonomic posture when using the Hugo™ RAS System. The heart rate was measured continuously using a polar-band. Secondly, the noise in the operating room was measured while using the Hugo™ RAS System. A camera was taking pictures every two seconds of the operating surgeon in order to assess body posture. In addition, a software was designed in order to calculates all joint angels (see Fig 1.). Finally, a touch sensor was developed to evaluate the usage of the arm rest (see Fig. 2). The ergonomic measurements were conducted while the subject was performing a structured validated training program. Three robotic exercises were chosen: Peg Board, Rope Walk and Ring Walk.

**Results:**
The CEMRobSurg setup is part of the surgical track for students which was succesfully implemented in our academic center in order to expose medical students to robotic surgical technology. This is an ongoing study and the evaluation of surgical residents and expert robotic surgeons to CEMRobSurg is the next step. First results show that especially in the neck region, a non-ergonomic position was detected when performing robotic surgery.

**Conclusion:**
This study is the first to demonstrate ergonomic measurements using the CEMRobSurg setup on the Hugo™ RAS System. CEMRobSurg is transferable and will be used prospectively on other robotic surgery platforms.

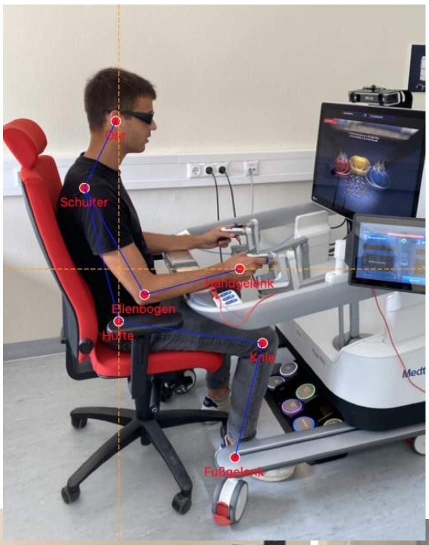

Figure 1.: Evaluation of the body posture. All joint angles are calculated in order to determine the ergonomic body posture using Hugo™ RAS System.

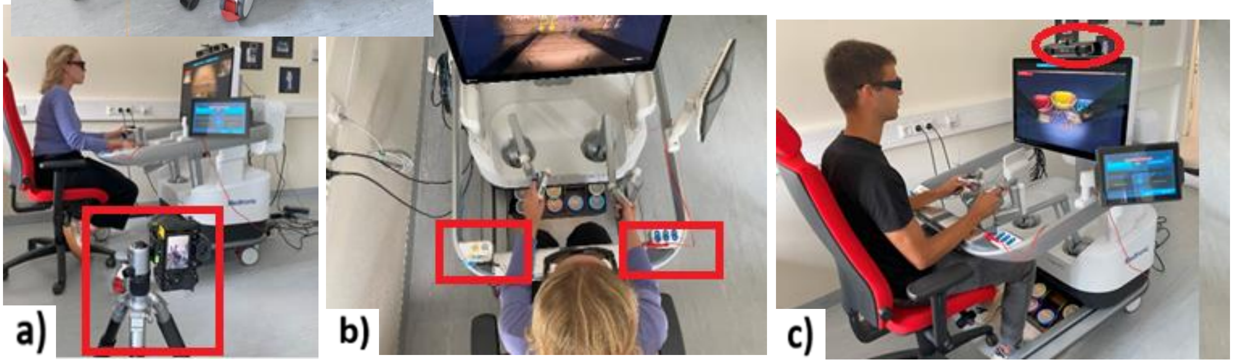

Figure 2. (a) usage of a camera to evaluate the posture of the operating surgeon. (b) usage of touch sensors to evaluate the use of the arm rest. (c) a sound recorder was used to evaluate the general noise in the room

**Hybrid pelvic exenterations: Robotic abdominal multiorgan resection followed by perineal specimen extraction and reconstruction.**
ID: 324

Hannes Neeff[1], Gabriel Seifert[1], Andrei Taran[2], Maximilian Klar[2], Andreas Jud[1], Julia Spiegelberg[1], Lars Schmitt[1], Stefan Fichtner-Feigl[1]

[1]*Universitätsklinik Freiburg; Department Chirurgie, Klinik für Allgemein- und Viszeralchirurgie, Freiburg, Deutschland*, [2]*Universitätsklinik Freiburg, Klinik für Frauenheilkunde, Freiburg, Deutschland*

**Background:**
Interdisciplinary multivisceral resections of the pelvis involve the removal of pelvic organs through laparotomy or via a minimally invasive approach. Much of the postoperative morbidity arises from the surgical access. A Hybrid approach using robotically assisted minimally invasive resection of the pelvic organs before perineal retrieval is thought to be beneficial.

**Materials and methods:**
We show the case of a posterior pelvic exenteration for recurrent vulvar adenocarcinoma with rectal invasion causing stenosis using a robotically assisted hybrid approach with an interdisciplinary team.

**Results:**
A minimally invasive hybrid approach is feasible, time efficient and safe when combining multidisciplinary teams. Reconstructive measures such as omental flaps etc. as well as stoma formation can also be done minimally invasive. The possibilities of perineal reconstruction of the pelvic floor using gracilis- or gluteal-fold flaps remains unaltered.

**Conclusion:**
Oncologic centers with experience in robotically assisted oncologic surgery can safely perform multiorgan resections in multidisciplinary settings using a hybrid approach as the individual steps of the operation e.g. rectal resection, hysterectomy and/or cystectomy have already become routine in some robotic centers.

**Impact of noise on laparoscopic performance - a prospective, open-label, randomized crossover trial**
ID: 448

Jean-Paul Bereuter[1], Mark Enrik Geißler[1], Rona Geißler[1], Sophia Schmidt[1], Nathalie Buck[1], Juliane Weiß[1], Grit Krause-Jüttler[1 2], Jürgen Weitz[1 2 3], Marius Distler[1 2], Florian Oehme[1], Felix von Bechtolsheim[1 2]

[1]*Department of Visceral, Thoracic and Vascular Surgery, Faculty of Medicine and University Hospital Carl Gustav Carus , Dresden, Deutschland*, [2]*Centre for Tactile Internet with Human-in-the-Loop (CeTI), Technische Universität Dresden, Dresden, Deutschland*, [3]*National Center for Tumor Diseases (NCT), Faculty of Medicine and University Hospital Carl Gustav Carus, Dresden, Deutschland*

**Background:**
Complex surgical procedures take a considerable amount of time and surgeons perform several complex cases daily, leading to exhaustion and fatigue. Despite these long working hours and a high workload, surgeons must stay focused during surgery to master complex procedures and guarantee optimal surgical results. During long operating times in the operating room (OR) surgeons are exposed to different types of noise, for instance machine alarms, phone calls and interacting objects. The aim of this study was to evaluate the effect of noise during laparoscopic surgery on the surgeons' performance and tissue handling.

**Materials and methods:**
This study included 30 medical students of the medical faculty Carl Gustav Carus in Dresden. All students were laparoscopic novices, who participated in a structured laparoscopic training curriculum until reaching a predefined proficiency. For the trial, all participants had to perform four laparoscopic tasks (Peg transfer, circle cutting, laparoscopic suture and knot and the Balloon resection) once under the influence of a realistic OR noise environment and once in a controlled silent lab environment. The order of intervention and control trial was randomized. During both the intervention and the control trial, students performed the four laparoscopic tasks on the ForceSense system (Medishield B.V., Delft, The Netherlands). The ForceSense system was used to measure the force exertion (peak and mean non-zero-force) as well as motion parameters (path length, mean instrument speed, volume of motion, etc.). During the intervention, students were exposed to a predefined noise audio at 70 dB. This audio included recorded sounds from the operating room, which were continuously played during the tasks. The audio tape was standardized with noises (e.g. anesthesia- and surgery-related noise) playing every 15-30 seconds. The order of sound effects varied to prevent habituation to the audio sequence. Primary endpoints were tissue handling defined by force exertion, task completion time and error rate. To assess the psychological workload the NASA-TLX score was used. The psychological impact of noise on participants was evaluated using a self-designed questionnaire.

**Results:**
All participants finished the trial and were assessed. Overall, no significant differences could be observed between both groups. For instance, during noise exposure, the task completion time was not significantly different from the control measurement when analyzing the performed tasks, such as the Peg transfer (ctrl: 134.65 seconds vs. noise: 132.55 seconds; $p = 0.70$), circle cutting (ctrl: 194.4 seconds vs. noise: 180.53 seconds; $p = 0.11$), Balloon resection (ctrl: 191.42 seconds vs. noise: 209.76 seconds; $p = 0.50$) and surgical suture and knot (ctrl: 231.04 vs. noise: 272.91 seconds; $p = 0.14$). Similarly, when analyzing the applied non-zero force during the laparoscopic tasks, no statistically significant differences were found in the Peg transfer task (ctrl: 0.83 N vs. noise: 0.85 N; $p = 0.64$), in the circle cutting task (ctrl: 0.83 N vs. noise: 0.82 N; $p = 0.69$), in the Balloon resection task (ctrl: 1.19 N vs. noise: 1.36 N; $p = 0.07$) and surgical knot task (ctrl: 0.91 N vs. noise: 0.94 N; $p = 0.20$) between control and noise conditions. In addition, concerning their maximal applied force, participants revealed no statistically significant differences regarding the Peg transfer (ctrl: 3.45 N vs. noise: 3.43 N; $p = 0.60$), circle cutting (ctrl: 2.99 N vs. noise: 2.97 N; $p = 0.85$), the Balloon resection (ctrl: 5.71 N vs. noise: 5.88

N; p = 0.85) and the surgical knot task (ctrl: 3.98 N vs. noise: 4.45 N; p = 0.33) when comparing the control and the noise measurements. The motion analysis for mean speed did not show significant differences between the control and the noise measurement for the left and right hand for any of the exercises performed. Similarly, the total path length was not differing significantly for the performed tasks for both measurements. In addition, the measured total volume of motion also showed no significant differences for the left and right hand for any of the conducted exercises. However, the observed error rate was higher under noise exposure in complex tasks such as the circle cutting (error rate: ctrl: 13.3% vs. noise: 30%; p = 0.005). In addition, participants were less precise when performing the laparoscopic suture and knot task (precision: ctrl: 10% vs. noise: 60%; p < 0.001).

**Conclusion:**
Our study revealed neither adverse nor beneficial systemic effects of a realistic OR noise environment on laparoscopic surgical skills. In general, based on our results, laparoscopic motion and tissue handling skills of surgical novices do not seem to deteriorate significantly under the impact of OR noise. However, even when not generally harming the students' tissue handling skills, the observed impact on precision and the error rate during advanced tasks warrants attention. Therefore, further research on the effect of noise on surgical skills could consider different aspects of noise or investigate the impact of noise in a more realistic clinical setting for instance how it might affect the performance of surgeons instead of surgical novices.

### Active aspiration versus simple compression to remove residual gas from the abdominal cavity after laparoscopic surgery a randomized clinical trial

ID: 826

Ahmed Abdelsamad[1 2]

[1]*Ev. Krankenhaus Lippstadt, Lippstadt, Deutschland*, [2]*Universität Witten/Herdecke, Witten, Deutschland*

**Background:**
After laparoscopic surgical procedures, residual gas in the abdominal cavity can cause postoperative pain, which is commonly located in the shoulder region. Previous studies suggested that post-laparoscopy pain can be prevented by active suctioning of intraabdominal gas at the end of surgery.

**Materials and methods:**
This randomized controlled trial (registered at DRKS 00023286) compared active suctioning versus manual compression in their ability to reduce pain after laparoscopic cholecystectomy. Patients scheduled for laparoscopic cholecystectomy were eligible for trial participation. The primary outcome measure was postoperative pain intensity after 12 h. All patients were examined by MRI scanning to quantify the intraabdominal gas volume after the intervention.

**Results:**
As planned, 60 patients were recruited. The two groups (n= 30 each) were very similar at the end of surgery. Active suctioning reduced the amount of residual pneumoperitoneum more than simple compression (median volume 1.5 versus 3.0 ml, p= 0.002). The primary outcome measure, abdominal pain after 12h, was slightly lower in the intervention group (-0.5 points, 95% confidence interval +0.5 to -1.7), but without reaching statistical significance (p= 0.37). After 12 h, shoulder pain was present in 10 patients in each group (p= 1.0). Independent of group assignment, however, residual gas volume was significantly associated with higher pain intensity.

**Conclusion:**
Active suctioning appears to have only a minor preventive effect on post-laparoscopy pain, probably because evacuation of the pneumoperitoneum remains incomplete in some patients. Other more effective maneuvers for gas removal should be preferred.

### The Serious Complications After Laparotomy (SEAL) model: Development of a prognostic model for emergency laparotomy

ID: 664

Stamatios Kokkinakis[1 1], Evangelos I. Kritsotakis[2], Konstantinos Paterakis[1], Garyfallia-Apostolia Karali[1], Vironas Malikides[1], Anna Kyprianou[1], Melina Papalexandraki[1], Charalampos S. Anastasiadis[3], Odysseas Zoras[3], Nikolas Drakos[4], Ioannis Kehagias[4], Dimitrios Kehagias[4], Nikolaos Gouvas[5], Georgios Kokkinos[5], Ioanna Pozotou[5], Panayiotis Papatheodorou[5], Kyriakos Frantzeskou[5], Dimitrios Schizas[6], Athanasios Syllaios[6], Ifaistion M. Palios[7], Konstantinos Nastos[8], Markos Perdikaris[8], Nikolaos V Michalopoulos[8], Ioannis Margaris[8], Evangelos Lolis[9], Georgia Dimopoulou[9], Dimitrios Panagiotou[10], Vasiliki Nikolaou[10], Georgios K. Glantzounis[11], George Pappas-Gogos[11], Kostas Tepelenis[11], Georgios Zacharioudakis[12], Savvas Tsaramanidis[12], Ioannis Patsarikas[12], Georgios Stylianidis[13], Georgios Giannos[13], Michail Karanikas[14], Konstantinia Kofina[14], Markos Markou[14], Emmanuel Chrysos[1], Konstantinos Lasithiotakis[1]

[1]*Department of General Surgery, University Hospital of Heraklion, University of Crete, School of Medicine, Heraklion, Greece*, [2]*Laboratory of Biostatistics, School of Medicine, University of Crete, Heraklion, Greece*, [3]*Department of Surgical Oncology, University Hospital of Heraklion, University of Crete, School of Medicine, Heraklion, Greece*, [4]*Department of Surgery, University General Hospital of Patras, School of Medicine, University of Patras, Patras, Greece*, [5]*Department of Surgery, General Hospital of Nicosia, School of Medicine, University of Cyprus, Nicosia, Cyprus*, [6]*First Department of Surgery, National and Kapodistrian University of Athens, Laikon General Hospital, Athens, Greece*, [7]*Second Propaedeutic Department of Surgery, National and Kapodistrian University of Athens, Laikon General Hospital, Athens, Greece*, [8]*Department of Surgery, University General Hospital Attikon, School of Medicine, University of Athens, Athens, Greece*, [9]*Department of Surgery, General Hospital of Volos, Volos, Greece*, [10]*Department of Surgery, General Hospital of Trikala, Trikala, Greece*, [11]*Department of Surgery, University Hospital of Ioannina, Ioannina, Greece*, [12]*Department of Surgery, Ippokrateion General Hospital of Thessaloniki, School of Medicine, Aristotle University of Thessaloniki, Thessaloniki, Greece*, [13]*Second Department of Surgery, Evangelismos General Hospital, Athens, Greece*, [14]*Department of Surgery, University General Hospital of Alexandroupolis, School of Medicine, University of Thrace, Alexandroupolis, Greece*

**Background:**

Emergency laparotomy (EL) encompasses a spectrum of procedures associated with considerable morbidity and mortality worldwide. A prognostic model was developed and validated for serious postoperative complications following EL.

**Materials and methods:**

A prospective multicentre cohort of EL patients from Greece and Cyprus was used for model development. The outcome of interest was serious complications (Clavien-Dindo grade III or greater). The Least Absolute Shrinkage and Selection Operator (LASSO) regression was used for predictor selection. The model's performance was evaluated with the use of discrimination and calibration metrics, and Decision Curve Analysis was used to analyse the clinical usefulness of the model. Internal validation with bootstrapping was employed to provide optimism-corrected measures. The newly developed model was benchmarked against the American College of Surgeons National Surgical Quality Improvement Program's (ACS-NSQIP) Surgical Risk Calculator.

**Results:**

The final SErious complications After Laparotomy (SEAL) model was developed with 6 predictors retained by the LASSO: preoperative albumin, blood urea nitrogen, American Society of Anaesthesiology score, sepsis or septic shock, dependent functional status, and ascites. Its discrimination (optimism-corrected c-statistic: 0.80, 95% confidence interval [CI]: 0.79 - 0.81) and calibration (optimism-corrected calibration slope: 1.01, 95%CI: 0.99-1.03) were superior to the

Abstracts – DCK 2024 – 141st Congress of the German Society of Surgery – Leipzig, April 24–26 • DOI 10.1515/iss-2024-2003
Innov Surg Sci 2024; 9 (Special Suppl 1): s18–s203

recalibrated ACS-NSQIP, demonstrating better clinical utility with the DCA. The SEAL model is presented with a nomogram.

**Conclusion:**

The newly developed parsimonious SEAL model can assist with the risk stratification of EL patients. Further conclusions regarding its performance in different settings can be drawn after external validations are performed.

Abstracts – DCK 2024 – 141st Congress of the German Society of Surgery – Leipzig, April 24–26 • DOI 10.1515/iss-2024-2003
Innov Surg Sci 2024; 9 (Special Suppl 1): s18–s203

**Vascularity of the gastric conduit predicts complications after Ivor-Lewis esophagectomy**
ID: 261

Julian Lemties[1], Carolin Scheidt[1], Naita Wirsik[1], Lenadra Lukomski[1], Anders Grabenkamp[1], Alexander Stier[1], Quaas Alexander[2], Su-Ir Lyu[2], Thomas Schmidt[1], Hans Fuchs[1], Wolfgang Schröder[1], Christiane Bruns[1], Lars Schiffmann[1]

[1]*Uniklinik Köln, Klinik für Allgemein-, Viszeral-, Tumor- und Transplantationschirurgie, Köln, Deutschland*, [2]*Uniklinik Köln, Klinik für Pathologie, Köln, Deutschland*

**Background:**
Anastomotic leakage (AL) contributes to postoperative morbidity and mortality after Ivor-Lewis esophagectomy (OE). Vascular high-risk patients with aortic calcification or a stenosis of the coeliac trunc show a significantly increased risk of AL. We have previously shown that ischemic conditioning of the gastric conduit prior esophagectomy in these high-risk patients is a safe procedure that induces neoangiogenesis at the anastomotic site. This retrospective study aims to further test the hypothesis that vascularization of the gastric conduit in two stage OE has direct implications for anastomotic healing and postoperative morbidity.

**Materials and methods:**
80 patients who underwent two stage esophagectomy from 2016-2021 at our center were analyzed from a prospectively maintained database. Histologic sections from gastric donuts derived from the stapler esophagogastric anastomosis were stained against CD34 to detect intramucosal vessels. Analysis of micro vessel density (MVD) was performed blinded using ImageJ. Patients were stratified into three groups (low/intermediate/high MVD). MVD was correlated to clinical outcome measures.

**Results:**
Patients with high MVD showed a significantly lower rate of anastomotic leakage in comparison to patients with low MVD (0% vs. 25% p=0,047). In line with this, a high MVD was associated with a lower rate of major complications (Clavien Dindo>IIIa) (10% vs.45% p=0,031) and a shorter hospital stay (16,9 days vs. 25,8 days p=0,001).

**Conclusion:**
Vascularization of the gastric conduit might function as surgical biomarker of anastomotic leakage in patients undergoing two-stage esophagectomy. Prospective trials have to further substantiate this finding.

### Defining Benchmarks for Gastric Cancer Surgery - A Global Multicenter Analysis

ID: 296

Felix Berlth[1], Marcel Schneider[2], Jeesun Kim[3], Maria Bencivegna[4], Yukuta Sugita[5], Peter Grimminger[6], Giovanni De Manzoni[4], Souya Nunobe[5], Han-Kwang Yang[3], Christian Gutschow[2]

[1]*Department of General, Visceral and Transplant Surgery, University Hospital Tübingen, Tübingen, Deutschland*, [2]*Department for General and Transplantation Surgery, University Hospital Zurich, Zurich, Schweiz*, [3]*Department of Surgery, Seoul National University Hospital, Seoul, Korea, Rep. of South*, [4]*Department of Surgery, University Hospital Verona, Verona, Italy*, [5]*Department of Gastroenterological Surgery, Cancer Institute Hospital, Tokyo, Japan*, [6]*Department of General, Visceral and Transplant Surgery, University Medical Center Mainz, Mainz, Deutschland*

**Background:**
Oncological gastrectomy remains associated with relevant morbidity. Our aim was to define "best possible" outcomes for total (TG) and distal (DG) gastrectomies, while also accounting for regional differences.

**Materials and methods:**
Of 9356 gastrectomies, performed over a 5-year period at 43 specialized centers on 5 continents, we selected 1569 benchmark patients based on criteria of low comorbidity (ASA score ≤2, age ≤65 years, BMI 19-29kg/m2, no relevant comorbidities). Endpoints included postoperative morbidity classified according to Clavien-Dindo (CD) and the Comprehensive Complication Index (CCI). Benchmark values or “best achievable results” were defined as $25^{th}/75^{th}$ percentiles of median outcome parameters in participating centers.

**Results:**
Benchmark patients underwent TG (n=498) or DG (n=1071) with minimally invasive surgery used in 66.7%; 16.2% developed at least one complication and 7.5% experienced major morbidity (≥CD IIIA). Overall, the most common complication was anastomotic leakage (2.7%), and 90-day mortality rate was 0.4%.
Benchmark values for TG were ≥91.6%, ≥26, <7.1%, ≤7.6%, ≤20.0%, ≤9.3%, ≤51.4, 0% (Europe/America) and ≥99.4%, ≥49, <1.0%, ≤1.8%, ≤21.2%, ≤11.3%, ≤24.8, 0% (East Asia) for R0 resection rate, LN yield, escalation of care, readmission, overall and major complications, CCI, and 90-day mortality. In DG, these benchmark values were 100%, ≥24, 0%, 0%, ≤14.6%, ≤2.2%, ≤28.0, 0% (Europe/America)and ≥99.2%, ≥39, <0.6%, ≤0.4%, ≤14.8%, ≤6.5%, ≤24.8 and 0% (East Asia).

**Conclusion:**
This study presents global benchmark values that can be used to evaluate performance and outcome quality in total and distal oncologic gastrectomy.

**Are there any differences concerning clinical outcome after the surgical management of patients with stomach or duodenal perforation?**
ID: 341

Saleh Lahes[1], Gudrun Wagenpfeil[2], Matthias Glanemann[3]

[1]*Bochum, Uniklinik Knappschaftskrankenhaus Bochum, Bochum, Deutschland*, [2]*Institute for Medical Biometrics, Epidemiology and Medical Computer Science, Saarland University, Homburg/Saar, Germany, Homburg, Deutschland*, [3]*Department of General, Visceral, Vascular and Pediatric Surgery, Saarland University, Homburg*

**Background:**
Free perforation of the stomach or the duodenum usually necessitates emergency surgery. In fact, perforation is associated with short-term mortality and morbidity in up to 30 and 50% of patients, respectively, which is caused by secondary peritonitis and sepsis. This retrospective study aimed to compare the early postoperative clinical outcome of patients with stomach and duodenal perforation focusing on morbidity and mortality in order to figure out differences which could, if available, be managed by potential changes in surgical management.

**Materials and methods:**
The incidence of any postoperative complication (83.3 vs. 55.4%; p=0.005), the rate of planned reoperation (22.2 vs. 5.4%; p=0.012), median operation time (132 vs. 92 min; p=0.009), as well as the median hospital stay after surgery (19.5 vs 12.5 days, p=0.023) were significantly increased in patients with DP compared to those with SP. In addition, surgical (47.2 vs. 32.4%; p=0.14) and non-surgical complications (52.8 vs. 41.9%; p=0.31), as well as mortality (33.3 vs. 18.9%; p=0.15) were common in the total patient population, and even higher in DP than in SP patients.

**Results:**
The incidence of any postoperative complication (83.3 vs. 55.4%; p=0.005), the rate of planned reoperation (22.2 vs. 5.4%; p=0.012), median operation time (132 vs. 92 min; p=0.009), as well as the median hospital stay after surgery (19.5 vs 12.5 days, p=0.023) were significantly increased in patients with DP compared to those with SP. In addition, surgical (47.2 vs. 32.4%; p=0.14) and non-surgical complications (52.8 vs. 41.9%; p=0.31), as well as mortality (33.3 vs. 18.9%; p=0.15) were common in the total patient population, and even higher in DP than in SP patients.

**Conclusion:**
The postoperative problems which might occur after surgery for DP or SP were almost comparable, in both situations being common and life-threatening. However, patients with DP experienced these problems significantly more often, indicating a more complex injury and thus requiring considerably more medical facilities for their treatment.

### VATS: The guiding light in the treatment of extramural esophageal ruptures

ID: 359

Nandita Raman[1], Christina Lohrenz[1], Daniel Benten[1], Stefan Meierling[1]

[1]*Asklepios Klinikum Harburg, Hamburg, Deutschland*

**Background:**
Historically esophageal ruptures (ER) have been considered a highly lethal condition with mortality rates on average ranging von 10-40%. The etiology of which itself is as multifarious as its complications. When etiologies range from idiopathic to Boerhaave syndrome and from connective tissue disorders to esophageal carcinoma, one can see why not only detection but also treatment can prove to be difficult. Its complications, just as difficult, usually leads if not to death but to a significant decrease in quality of life. Modern medicine, however, has finally advanced to a degree that the treatment of ER can offer the highest chance of survival than ever before.

**Materials and methods:**
The Pittsburgh Severity Score (PSS) is used to determine the severity of the patient’s condition based on multiple factors. For less severe cases with intramural ruptures, a conservative procedure including endoscopic treatment is preferred. However, for higher scores with extramural ruptures, treatment usually varies based on severity and the experience in the center in which it is treated.
Traditionally, thoracoscopic approaches to esophageal repair and debridement are only considered in certain cases. In our department, we have treated 13 cases of spontaneous ER over the past two years - 3 cases of Boerhaave Syndrom, 3 cases after esophageal resection due to carcinoma, 1 case of late stage lung cancer, 2 cases of postoperative complications due to non-oncological operations and 2 cases after endoscopic intervention. Through the early decision to conduct thoracoscopic debridement of both thorax sides, we were able to contain the inflammation and bacterial progress by ensuring a sterile as possible environment after thorough lavage. Through a second look VATS a few days later, we could also monitor the success of endoscopic eso-sponge treatment as well as suture greater defects. In our experience, we believe thoracoscopic treatment is crucial to better treatment surveillance, ascertainment of results, and to avoid life threatening complications such as pleural empyema and mediastinitis.

**Results:**
In this presentation, we will describe our standardized treatment plan for a statistically and significantly lower mortality rate for extramural esophageal ruptures. In our single center experience, historically, and more specifically over the past two years, following our standardized protocol we have achieved a 100% survival rate over all ages and etiologies for severe ER.

**Conclusion:**
We believe VATS treatment to be the guiding light in our treatment protocol for ER.

**An International Delphi Consensus on the Crucial Technical Steps in Lymphadenectomy and Anastomosis Creation in Minimally Invasive Total Gastrectomy for Gastric Cancer**
ID: 371

Amila Cizmic[1], Ivan Romic[2], Andrea Balla[3], Nicolò Barabino[4], Sheraz Markar[5], Felix Nickel[1], Anuja T Mitra[6]

[1]*Klinik für Allgemein-, Viszeral- und Thoraxchirurgie, Universitätsklinikum Hamburg-Eppendorf, Hamburg, Deutschland*, [2]*Department of Hepatobiliary Surgery & Liver Transplantation, University Hospital Centre Zagreb, Croatia*, [3]*Coloproctology and Inflammatory Bowel Disease Surgery Unit, IRCCS San Raffaele Scientific Institute, Milan, Italy*, [4]*Department of Surgical Sciences & Integrated Diagnostic, University of Genoa, Italy*, [5]*Nuffield Department of Surgical Sciences, University of Oxford, United Kingdom*, [6]*Department of Surgery & Cancer, Imperial College London, United Kingdom*

**Background:**
Minimally invasive total gastrectomy (MIG) is the preferred approach for treating patients with gastric cancers. MIG is an advanced procedure that requires technical expertise, particularly when performing lymphadenectomy and anastomosis creation for oncological indications. To improve clinical outcomes and maintain confidence in the reliability of MIG, there must be a consensus on key technical steps of lymphadenectomy and anastomosis creation, which are currently lacking. The aim of this study was to determine an expert consensus from an international panel regarding these integral technical steps required to perform MIG for oncological indications using the Delphi method.

**Materials and methods:**
Twenty-eight expert upper gastrointestinal and general surgeons participated in three online Delphi rounds. MIG was deconstructed into integral technical steps on lymphadenectomy and anastomosis creation through local and international expert opinion and literature evidence generating a 100-point scoping survey. In all three rounds, the expert panelists voted on the issues generated in previous rounds concerning importance, difficulty, or agreement using an online questionnaire. The consensus was defined if >80% of the panel agreed to a statement. Internal consistency and reliability were evaluated using Cronbach's alpha, and univariate statistics were undertaken on individual outcome measures. The Delphi was closed when consensus was reached.

**Results:**
Three Delphi rounds yielded 41 consensus statements regarding MIG lymphadenectomy and general aspects of anastomosis creation. Cronbach's alpha for round 1 was 0.896 and 0.886, and for round 2, 0.848 and 0.779, according to the type of answers regarding difficulty or importance.

**Conclusion:**
This is the first international Delphi consensus regarding the essential technical steps of performing MIG for gastric cancers. The results of this consensus inform best surgical practices and provide a platform for creating surgical video-assessment tools designed to measure surgical quality in MIG.

### Enhancing R0-Resection Rates: Comparative Analysis of Robotic-Assisted Thoracoscopic Ivor Lewis Esophagectomy (RAMIE) Versus Open Transthoracic Approaches at a High-Volume Center

ID: 412

Jens Peter Hölzen[1], Lukas Fortmann[1], Ann-Kathrin Eichelmann[1], Carsten Szardenings[2], Andreas Pascher[1], Mazen Juratli[1]

[1]*Klinik für Allgemein-, Viszeral- und Transplantationschirurgie , Universitätsklinikum Münster, Münster*, [2]*Institut für Biometrie und klinische Forschung, Universität Münster , Münster*

**Background:**
Robotic-assisted minimally invasive esophagectomy (RAMIE) is increasingly used. The aim of this study is the analysis of short-term clinical and oncological outcomes of RAMIE compared to open transthoracic esophagectomy approaches (OTE).

**Materials and methods:**
A propensity score matched analysis of 185 RAMIEs and 223 OTEs following standardized Ivor Lewis esophagectomy was performed. After 1:1 nearest neighbor matching to account for confounding by covariates, outcomes of 181 RAMIEs and 181 OTEs were compared.

**Results:**
The patient characteristics showed significant differences in the age distribution and in comorbidities such as coronary heart disease, arterial hypertension, and anticoagulant intake. The R0-resection rate of RAMIE (96.7 %) was significantly higher than OTE (89.0 %, $p = 0.004$). 30-day mortality and hospital-mortality showed no significant differences. Postoperative pneumonia rate following RAMIE (12.7 %) was significantly reduced (OTE 28.7 %, $p < 0.001$). RAMIE had a significant shorter intensive care unit stay ($p < 0.001$) and shorter hospital stay ($p < 0.001$).

**Conclusion:**
This retrospective propensity score matched single-center study established that RAMIE has superior short-term oncological and clinical outcomes compared to OTE. This is crucial as the higher R0-resection rate possibly predicts for increased long-term survival. The better overall postoperative convalescence may help to improve the risk management of esophagectomies.

**Risk factor analysis for developing major complications following esophageal surgery-a two centre study.**
ID: 469

Björn-Ole Stüben[1], Gabriel Plitzko[2], Louisa Stern[2], Rainer Schmeding[1], Karl-Frederick Karstens[2], Reeh Matthias[2], Jürgen-Walter Treckmann[1], Jakob Izbicki[2], Fuat Saner[1], Jan Neuhaus[1], Michael Tachezy[2], Dieter Hoyer[1]

[1]*Klinik für Allgemein-, Viszeral- und Transplantationschirurgie, Universitätsklinikum Essen, Essen, Deutschland*, [2]*Klinik für Allgemein-, Viszeral- und Thoraxchirurgie, Universitätsklinikum Hamburg-Eppendorf, Hamburg, Deutschland*

**Background:**
Esophagectomy still carries a high risk of morbidity and mortality compared to other major surgeries. With the aim of creating an easy-to-use clinical preoperative risk assessment tool as well as validating previously described risk factors for major complications following surgery, all patients undergoing esophagectomy at two major tertiary medical centres in Germany from 2008-2020 were analysed.

**Materials and methods:**
In total, 450 patients underwent esophagectomy for esophageal carcinoma at the Department of General, Visceral and Thoracic Surgery at the University Medical Centre, Hamburg, or at the Department for General, Visceral and Transplant Surgery at the Medical Centre University Duisburg-Essen, Germany (admitted from January 2008 to January 2020) and were retrospectively analysed. All patients which received either minimally invasive, hybrid or open surgery were included. Epidemiological as well as perioperative data was analysed to identify risk factors which impact major complication rates. The incidence of major complications was the primary endpoint of the study.

**Results:**
The mean age was 63 years with a bimodal distribution. There was a male predominance across the cohort (81% compared to 19%). Alcohol abuse ($p = 0.0341$), chronic obstructive pulmonary disease ($p=0.0264$) and cardiac comorbidity ($p=0.0367$) were all associated with a significantly higher risk of major complications in multivariate analysis. Neoadjuvant chemotherapy appeared to significantly reduce the risk of major complications after surgery ($p<0.0001$).

**Conclusion:**
We demonstrated various patient related risk-factors which increase the rate of major complications following esophagectomy. Patient tailored prehabilitation programs before esophagectomy focusing on minimising these risk factors may lead to better surgical outcomes, and this should be analysed in further studies where standardised prehabilitation measures are put in place.

**The role of multimodal therapy in the treatment of adenocarcinoma of esophagus and gastroesophageal junction. Analysis from the German Cancer Registry Groupof the Society of German Tumor Centers (GCRG/ADT)**
ID: 485

Markus Kist[1], Thaer S. A. Abdalla[1], Ruslan Arify[1], Monika Klinkhammer-Schalke[2], Sylke Ruth Zeissig[2], Kees Kleihues-van Tol[2], Ulrich Friedrich Wellner[1 2], Tobias Keck[1], Michael Thomaschewski[1], Richard Hummel[3]

[1]*Lübeck, Department of Surgery, University Medical Center Schleswig-Holstein, Campus Lübeck, Lübeck, Deutschland*, [2]*Berlin, German Cancer Registry Group of the Society of German Tumor Centers—Network for Care, Berlin, Deutschland*, [3]*Greifswald, Department of Surgery, University Medical Center Greiswald, Greifswald, Deutschland*

**Background:**
Esophageal resection is the only curative treatment option for patients with resectable esophageal adenocarcinoma. Multimodal regimes like perioperative chemotherapy and radiochemotherapy have become essential adjuncts to improve survival. Nevertheless, the optimal modal therapy remains controversial.

**Materials and methods:**
This population-based analysis was derived from 16 German cancer registries from 2000 to 2019. Patients with adenocarcinoma of the esophagus (EAC) or gastroesophageal junction (GEJ) with available cTNM stages were included in the final analysis.

**Results:**
Out of 24730 patients with EAC and GEJ, cTNM staging was documented in 9213 patients and was divided into Stage I (n=1405 (14%)), Stage II (n=1360 (14%)); Stage III (n=2680 (28%)); Stage IV (n=4293 (44%). The incidence of Stage IV decreased among the time periods (GEJ: 2000-2006 (71%), 2007-2012 (47%), 2013-2018 (40%); EAC: 2000-2006 (48%), 2007-2012 (35%), 2013-2018 (34%)), curative intended therapy increased drastically among the time periods (GEJ: 2000-2006 (23%), 2007-2012 (41%), 2013-2018 (50%); EAC: 2000-2006 (29%), 2007-2012 (48%), 2013-2018 (52%)), respectively. The 5 years OS was favorable in patients who received multimodal therapy compared to resection only in EAC stadium II for perioperative chemotherapy and in GEJ stadium II and III for perioperative Chemotherapy and neoadjuvante RCT + surgery. Perioperative Chemotherapy was the favorable multimodal modality in EAC stage III and GEJ stage III, whereas neoadjuvante RCT + surgery was favorable for GEJ stadium II.

**Conclusion:**
The integration of multimodal therapy regimes has increased survival in patients with EAC and GEJ and should be part of treatment in patients with cTNM Stage II.

### RAMIE is Associated with Increased Textbook Outcome: Improving Overall and Disease-Free Survival

ID: 538

Philippa Seika[1], Jonas Raakow[1], Max Magnus Maurer[1], Eva Maria Dobrindt[1], Ramin Raul Ossami Saidy[1], Paul Ritschl[1], Matthias Biebl[2], Johann Pratschke[1], Christian Denecke[1]

[1]*Charité Universitätsmedizin Berlin, Chirurgische Klinik, Berlin*, [2]*Ordensklinikum Linz GmbH Barmherzige Schwestern & Elisabethinen, Linz, Österreich*

**Background:**
Surgical complications affect oncological outcomes in esophageal surgery. However, the comprehensive nature of perioperative outcomes for patients with esophageal cancer is not adequately captured by individual quality measures. We conducted an analysis to explore the relationship between textbook outcome (TO), a novel composite parameter, and oncological outcomes after MIE and RAMIE respectively.

**Materials and methods:**
We included all patients who underwent total minimally invasive esophagectomy (MIE) and robotic-assisted esophagectomy (RAMIE) for primary esophageal or gastric cancer with curative intent between 2015 and 2021. TO was defined as negative resection margins (R0), retrieval of >20 lymph nodes, absence of major perioperative complications (Clavien-Dindo 3), no reintervention, no unplanned ICU readmission, hospital stay not exceeding 21 days, absence of 30-day readmission and mortality. Matched pair analysis was performed to adjust for selection bias in the robotic surgery group. We analyzed factors affecting the achievement of TO using a logistic regression model and the association between TO and long-term survival using a multivariate Cox proportional-hazards model.

**Results:**
Out of the 216 patients included in this study, 97 (44.3%) met all the TO criteria. Laparoscopic and robotic MIE procedures were performed on 81.5% (n=176) and 19.9% (n=43) cases, respectively. TO was achieved in 42.05%(n=74) of laparoscopic and 53.49% (n=23) of robotic cases. Anastomotic leakage occurred in 13.4% (n=29) of patients. Multivariate analysis indicated that tumor size and cardiovascular disease were associated with the achievement of TO after MIE. Following matched pair analysis, robotic surgery was significantly associated with the achievement of TO (p=0.030, OR 0.357, CI 95%(0.140-0.908)). After a mean follow-up period of 421 days (range: 11-834 days), patients with perioperative TO had a higher overall survival (OS) rate compared to those without perioperative TO (p=0.004, OR 0.234, CI 95% (0.087-0.627)).

**Conclusion:**
RAMIE for esophageal cancer offers an advantage over MIE by increasing the rate of textbook outcome. Achieving TO is associated with enhanced long-term survival in esophageal cancer patients after both MIE and RAMIE and warrants continued emphasis on surgical quality improvement.

### Linear Stapled Esophagojejunostomy is Associated with Reduced Anastomotic Leakage Rates Following Total Gastrectomy

ID: 555

Friederike Martin[1], Philippa Seika[1], Jonas Raakow[1], Eva Maria Dobrindt[1], Ramin Raul Ossami Saidy[1], Matthias Biebl[2], Johann Pratschke[1], Christian Denecke[1]

[1]*Charité Universitätsmedizin Berlin, Chirurgische Klinik, Berlin*, [2]*Ordensklinikum Linz GmbH Barmherzige Schwestern & Elisabethinen, Linz, Österreich*

**Background:**

After total gastrectomy, anastomosis-related complications such as leak, or stricture can impede overall survival. Previously, circular stapled esophagojejunostomy was the standard technique in our center. However, since 2021, a linear stapled esophagojejunostomy (Overlap) has been increasingly performed for reconstruction.

**Materials and methods:**

This study encompasses all patients who underwent either total or transhiatal extended gastrectomy with curative intent for gastric cancer between 2017 and 2023. A matched pair analysis was conducted to pair patients who had undergone linear anastomosis with those after circular anastomosis. The criteria for matching included age, ASA score, the surgical procedure undertaken, and tumor size. The correlation between the type of anastomosis and perioperative morbidity and mortality was examined using a logistic regression model.

**Results:**

From September 2017 to September 2023, 138 total and transhiatal extended gastrectomies with esophagojejunostomy were performed in our center. For reconstruction, a Linear stapler (Echelon powered 60mm, Johnson & Johnson) was employed in 32 cases (23.1%), and a Circular stapler was used in 106 cases (76.8%) (EEA™ OrVil™, Medtronic; n=8; 21.1% Echelon Circular powered, Johnson & Johnson). Postoperative mortality was absent, and the rate of surgical morbidity stood at 15.2% (n=21). Within this timeframe, a case (3.4%) of anastomotic leakage was noted in six cases following transhiatal extended gastrectomy. No anastomosis-related complications were observed post-establishment of a linear esophagojejunostomy.

**Conclusion:**

Implementing a linearly stapled esophagojejunostomy is technically feasible and safe, particularly in centers with substantial experience in linearly stapled anastomoses derived from bariatric surgery. The adoption of this novel anastomosis technique has notably diminished the incidence of anastomotic leakage after esophagojejunostomy significantly in our center.

### Robotic-assisted Vascularized Autologous Posterior Rectus Sheath Flap for Hiatal Hernia Repair after Minimally-Invasive Esophagectomy (+VIDEO)

ID: 614

Luca Giulini[1], Markus Diener[1], Attila Dubecz[1]

[1]*Klinikum Nuernberg - PMU, Department of Surgery , Nuernberg, Deutschland*

**Background:**
Robotic-assisted hiatal repair with vascularized autologous posterior rectus sheath was recently described as promising technique for hiatal reinforcement in patients with giant hiatal hernias. We describe our initial experience with this technique in patients with postoperative diaphragmatic hernia after minimally-invasive esophagectomy for cancer.

**Materials and methods:**
Robotic-assisted laparoscopy is performed and the herniated intestine is retracted in the abdominal cavity. The blood supply of the ligamentum teres and of the posterior rectus sheath is controlled with indocianine-green. Posterior rectus sheath is than dissected under preservation of the vascular supply through the ligamentum teres. The flap is than placed behind the left liver lobe, and fixed with multiple non-resorbable sutures without hiatal closure.

**Results:**
A total of 3 patients who previously underwent transthoracic minimally-invasive esophagectomy for cancer were treated with this method. There were no intraoperative or postoperative adverse events. No dysphagia or recurrences were registered at short-term follow-up

**Conclusion:**
Robotic-assisted vascularized autologous posterior rectus sheath flap might represent a safe, feasible and reliable method for hiatal hernia repair after esophagectomy

**Impact of preoperative sarcopenia on outcome for esophageal cancer patients undergoing esophagectomy**
ID: 637

Jennifer Merten[1], Nabila Gala Nacul Mora[2], Jens Hölzen[1], Mazen Juratli[1], Andreas Pascher[1], Ann-Kathrin Eichelmann[1]

[1]*University Hospital of Münster, Department of General, Visceral and Transplant Surgery, Münster, Deutschland*, [2]*University Hospital of Münster, Department of Clinical Radiology, Münster, Deutschland*

**Background:**
Sarcopenia is increasingly recognized as an independent predictor of clinical outcomes of cancer patients such as post-operative complications and prolonged hospital stays. Additionally, sarcopenia can also negatively impact overall survival because of reduced ability to tolerate treatments like neoadjuvant therapy and surgery. At particular risk among cancer patients is the esophageal cancer population because of tumor stenosis leading to dysphagia and impossibility of oral feeding in many patients. The purpose of the current study was to investigate the effect of preoperative sarcopenia on postoperative complications in patients undergoing esophagectomy for esophageal cancer.

**Materials and methods:**
This retrospective study includes all patients who underwent full-robotic abdominothoracic esophageal resection because of esophageal cancer at our institution between 06/2020 and 12/2022. The skeletal muscle index (SMI) was assessed using the preoperative computed tomographic staging scans by measuring the cross-sectional muscle area at the third lumbar vertebral level. Following cut off SMI values were used to define sarcopenia: men: 52,4 $cm^2/m^2$, women: 38,5 $cm^2/m^2$ according to [1]. Recorded parameters included demographic data, neo-adjuvant treatment, histologic findings, postoperative course and overall- and disease-free survival.

**Results:**
A total of 113 cases were included in this preliminary analysis. 97 patients (86%) were classified as having sarcopenia. Of these, 38 patients (40%) had a preoperatively or during esophageal resection implemented percutaneous endoscopic jejunostomy (PEJ) tube to ensure enteral nutrition. Despite age, groups were comparable regarding neoadjuvant treatment and histologic findings (mean age sarcopenic patients: 66y vs. 61y non-sarcopenic patients, $p = 0.03$). There was no difference in the incidence of occurrence of anastomotic leakage, pulmonary complications, need for re-operation, length of (ICU) stay and overall- and disease-free survival between sarcopenic and non-sarcopenic patients.

**Conclusion:**
Incidence of sarcopenia is very high among esophageal cancer patients. In our study population, however, preoperative sarcopenia failed to predict complications or long-term outcome after esophagectomy for esophageal cancer. 40% of the sarcopenic patients received a PEJ. This relatively high proportion of implemented PEJ could have contributed to the observed comparable outcomes between sarcopenic and non-sarcopenic patients. This observation underlines the importance of prehabilitation and nutritional support for patients with preoperative sarcopenia.

1. Prado, C.M., et al., *Prevalence and clinical implications of sarcopenic obesity in patients with solid tumours of the respiratory and gastrointestinal tracts: a population-based study.* Lancet Oncol, 2008. **9**(7): p. 629-35.

**Quality of life and independent factors associated with poor digestive function after Ivor Lewis esophagectomy**
ID: 658

Valerian Dirr[1], Diana Vetter[1], Thomas Sartoertti[2], Marcel Schneider[1], Francesca Da Canal[1], Christian A. Gutschow[1]

[1]*Klinik für Viszeral- und Transplantationschirurgie, Zürich, Schweiz*, [2]*Klinik für Diagnostische und Interventionelle Radiologie, Zürich, Schweiz*

**Background:**
Transthoracic esophagectomy results in a radical change in foregut anatomy with multifaceted consequences on digestive physiology. The aim of this study was to identify factors associated with poor functional outcomes by assessing multiple dimensions of digestive performance and health-related quality of life (HRQL).

**Materials and methods:**
Patients with cancer-free survival following Ivor Lewis esophagectomy were included. Four functional syndromes (dysphagia, gastroesophageal reflux disease (GERD), delayed gastric conduit emptying (DGCE), and dumping syndrome (DS)) and HRQL were assessed using specifically designed questionnaires. Patient outcomes were compared with healthy controls. Independent factors associated with poor digestive performance were identified by multivariable analysis.

**Results:**
Sixty-five patients after surgery and 50 healthy volunteers participated in this study. Compared with controls, patients had worse outcome for dysphagia, GERD, DS, and HRQL, but not for DGCE. Multivariate analysis showed a significant correlation of reduced digestive performance with ASA score, squamous cell cancer, and open or hybrid surgical access and (neo)adjuvant therapy. In contrast, no individual patient factor was found to be associated with dumping syndrome.

**Conclusion:**
Digestive function and HRQL are substantially impaired after Ivor Lewis esophagectomy for cancer. Comorbid patients undergoing multimodal treatment and open access surgery for squamous cell carcinoma have the highest risk of poor functional outcome.

Abstracts – DCK 2024 – 141st Congress of the German Society of Surgery – Leipzig, April 24–26 • DOI 10.1515/iss-2024-2003
Innov Surg Sci 2024; 9 (Special Suppl 1): s18–s203

**Does the percentage of signet ring cells (SRC) influence response and survival in esophagogastric adenocarcinoma (EGA) treated by neoadjuvant chemotherapy followed by surgery?**
ID: 710

Sabine Schiefer[1 2], Timon Fiedler[3], Nerma Crnovrsanin[2], Isabell Schütte[2], Georg Martin Haag[4], Henrik Nienhüser[2], Leila Sisic[2]

[1], [2]*Chirurgische Klinik, Allgemein-, Viszeral-, Transplantationschirurgie, Heidelberg, Deutschland*, [3]*Uniklinik Heidelberg, Pathologisches Institut, Heidelberg*, [4]*Nationales Zentrum für Tumorerkrankungen, Medizinische Onkologie, Heidelberg*

**Background:**
The number of new cases for esophagogastric cancer is slowly declining in the last years but subgroup analyses show an increase in the incidence of signet ring cell carcinoma (SRCC). The prognostic impact of neoadjuvant chemotherapy (nCTX) in patients with esophagogastric SRCC is still controversial. Aim of this study was to compare the clinicopathological features and the impact on the prognosis and histopathological response of esophagogastric cancer patients with various amounts of signet ring cells.

**Materials and methods:**
Patients with signet ring cells in their final histopathology were identified from January 2001 to December 2020 in a prospectively maintained database. Patients who underwent nCTX followed by surgical resection were included. After PAS staining a pathologist screened each tissue sample. According, to the number of signet ring cells in their histopathology and the consensus on the pathological definition and classification of poorly cohesive gastric carcinoma patients were categorized into 3 groups: (1) poorly cohesive NOS (<10% SRC), (2) combined poorly cohesive NOS and SRCC (11-89% of SRC) (3) SRC type (>90% of SRC). Overall survival (OS) was calculated according to Kaplan-Meier (log rank test), and histopathological response was determined according to Becker. Univariate and multivariate cox regression analyses were performed.

**Results:**
A total of 169 patients were included in this study. 57.4% of the patients were male with a median age of 60 years. FLOT regime was given to 52.7% of the patients, an Epirubicin-based chemotherapy was given to 31.4%, and 11.2% received other kinds of chemotherapy.
Most patients underwent a total gastrectomy (42.6%). Other surgical procedures were transhiatal extended gastrectomy (21.3%), subtotal gastrectomy (15.4%), Ivor-Lewis procedure (16.6%) and esophagogastrectomy (4.1%), depending on tumor localization.
89 patients showed <10% SRC (group 1), 43 11-89% SRC (group 2), and 27 ≥ 90% group 3). The majority of tumors were locally advanced (77% cT3) and showed local lymph node invasion (85% cN+) in clinical staging. There was no significant difference in between the three groups for patient or treatment characteristics. 90.5% of the patients were identified as non-responders to neoadjuvant chemotherapy, however, response rate did not differ significantly between the SRC groups (p=0.351). There was no significant difference with regard to ypT- (p=0.648), ypN- (p=0.860), ypM- (p=0.659), and R-category (p=0.722) between the three groups.
Survival did not differ according to percentage of SRC (p=0.45). Median OS was 31.5 months in group 1, 23.0 months in group 2, and 38.8 months in group 3.
Univariate analysis revealed cT- (p=0.014), cM-category (p<0.01), localization (p<0.01), ypT- (p<0.01), ypN- (p<0.01), ypM-category (p<0.01), R-category (p<0.01), and histopathological response (p=0.016) as prognostic factors for OS. In multivariate analysis localization, cT-, ypN-, ypM-, and R- category were selected as independent prognosticators for OS.

**Conclusion:**

This retrospective analysis shows that survival for SRCC does not seem to be associated with the percentage of SRC after nCTX followed by resection. The majority of patients with SRCC were non-responders to neoadjuvant chemotherapy. However, we found no association between percentage of SRC in the resection specimen and histopathological response according to Becker.

Limitations were limited patient numbers, different CTX regimens applied, and especially lack of preoperative biopsy specimens.

Our analysis reveals research on this topic is still lacking and requires more focus in the future to find optimal therapeutic strategies for SRCC patients.

**Nodal metastatic pattern in gastric cancer: a descriptive study of a single institution cohort**
ID: 749

Marlene Schelletter[1 2], Mickael Chevallay[1], Christian Toso[1], Stefan Mönig[1], Minoa Karin Jung[1]

[1]*Hôpitaux universitaires de Genève, Genève, Schweiz*, [2]*Universität Heidelberg, Heidelberg, Deutschland*

**Background:**
Gastric cancer can cause various nodal metastatic patterns. Our leading question is if any relation can be evidenced between the metastatic LN stations and tumor location, pT-stage and histological subtype. The aim of this descriptive study is to detect a possible metastatic pattern in gastric adenocarcinoma. This should enforce the need for a D2 lymphadenectomy in gastrectomy.

**Materials and methods:**
LN stations were examined for all gastric cancer patients who underwent total or subtotal gastrectomy (HUG, single institution trial).

**Results:**
Over a period of 6 years and 8 months (May 2016 to December 2022) a total number of 86 patients were included in this study who underwent either total or subtotal gastrectomy. Positive lymph node metastases were found in 42 (49%) patients. The mean number of harvested lymph nodes was 46,4 and the mortality rate was 0%.
Middle tumors exhibited the highest prevalence of lymph node metastases (53%) compared to proximal and distal tumors (48%, 45%).
Proximal tumors primarily metastasized to LN station 2 and 9 (16%, 12%); nevertheless, metastases were also frequently found in distal LN stations 5 and 6 (8%, 16%).
In case of a tumor in the middle third, LN stations 1, 6, 8, 9 and 11 were each highly affected (16%) and LN stations 3 and 7 were very highly affected (21%). In contrast, a low frequency of LN metastases was observed in station 2 and 4sa (0%, 5%).
Distal tumors metastasized most frequently to nearby LN stations 4d and 6 (8%, 15%) meanwhile they could also present a high likelihood for metastases in proximal LN stations such as 7 and 9 (8%, 8%).
Diffuse and mixed type gastric cancer showed a higher prevalence (50%, 72%) for LN metastases than the intestinal type (42%).
The higher the pT stage, the more likely nodal metastases were found. A pT stage under 2 did not present LN metastases.

**Conclusion:**
LN stations close to the tumor location have a higher prevalence of LN metastases. Nevertheless, metastases can be found in any LN station regardless of the tumor location, pT stage or histological subtype. The findings reinforce the need for a D2 lymphadenectomy as it is required according to the JGCA-Classification (Japanese Gastric Cancer Association). In our cohort the lymphatic metastatic invasion pattern of adenocarcinoma of the middle third allowed for subtotal gastrectomy omitting removal of station 2 and 4sa in selected patients in respect of sufficient proximal margin.

Abstracts – DCK 2024 – 141st Congress of the German Society of Surgery – Leipzig, April 24–26 • DOI 10.1515/iss-2024-2003
Innov Surg Sci 2024; 9 (Special Suppl 1): s18–s203

**Prevalence and long-term implications of preoperative anemia in patients undergoing elective general surgery – a retrospective cohort study at a university hospital**
ID: 40

Tamara Braunschmid[1], Alexandra Graf[2], Ernst Eigenbauer[2], Gerhard Schak[3], Klaus Sahora[4], David Baron[5]

[1]*, Klinik Floridsdorf, Wiener Gesundheitsverbund, Wien, Österreich*, [2]*Center for Medical Data Science, Medical University of Vienna, Wien, Österreich*, [3]*Philips Austria, Wien, Österreich*, [4]*Universitätsklinik für Allgemeinchirurgie, Medizinische Universität Wien, Wien, Österreich*, [5]*Universitätsklinik für Anästhesie, Intensivmedizin und Schmerztherapie, Medizinische Universität Wien, Wien, Österreich*

**Background:**
Awareness of the negative impact of preoperative anemia on perioperative morbidity and mortality is rising. Anemia is a potentially modifiable factor, and its therapy might improve patient outcome in elective surgery. Nevertheless, patients with preoperative anemia frequently undergo elective surgery without receiving adequate preoperative treatment.
The aim of this retrospective study was to assess the prevalence of anemia in a cohort of patients undergoing elective general surgery at a university hospital. Furthermore, we investigated the influence of anemia on short-term and long-term postoperative outcome.

**Materials and methods:**
In this single-center cohort study, we analyzed 6908 adult patients who underwent elective general surgery. Patients undergoing day-clinic surgery were excluded. In all patients, preoperative hemoglobin (Hb) concentration and hematocrit (Hct) was available**.**

**Results:**
Of all patients analyzed, 32.9% were anemic (21.0% mild, 11.8% moderate, 1.1% severe). Median time to last follow up was 5.2 years. During the whole study period, 27.1% of patients died (1.2% died during the hospital stay); median time to death was 1.3 years. Patients with preoperative anemia had significantly higher mortality rates ($p<0.001$) and a higher probability of postoperative complications ($p<0.001$). Likewise, receiving blood transfusions was associated with a higher risk of death ($p<0.001$).

**Conclusion:**
This retrospective single-center analysis confirmed that preoperative anemia is common, and is a significant risk factor for unfavorable postoperative outcome. As anemia is a modifiable risk factor, the implementation of a PBM concept is crucial to reduce detrimental postoperative events associated with anemia.

Abstracts – DCK 2024 – 141st Congress of the German Society of Surgery – Leipzig, April 24–26 • DOI 10.1515/iss-2024-2003
Innov Surg Sci 2024; 9 (Special Suppl 1): s18–s203

**Impact of enhanced recovery after surgery protocol compliance on outcome after pancreatic surgery – a large single center analysis**
ID: 319

Alina Ritter[1], Freya Brodersen[1], Mara Goetz[1], Christine Nitschke[1], Tobias Schneider[1], Björn Wellge[2], Jakob R. Izbicki[1], Faik G. Uzunoglu[1], Thilo Hackert[1], Thilo Welsch[1]

[1]*Klinik für Allgemein-, Viszeral- und Thoraxchirurgie, Universitätsklinikum Hamburg-Eppendorf*, [2]*Klinik für Allgemein-, Viszeral- und Kinderchirurgie, Universitätsmedizin Göttingen*

**Background:**
The Enhanced Recovery After Surgery (ERAS) protocol for patients with pancreatic resections considers more than20 components with predominantly evidence-based recommendations aiming to reduce postoperative morbidity and shorten hospital stay. The present study aimed to investigate the effect of ERAS compliance on patient morbidity and hospital stay based on a large single center series.

**Materials and methods:**
Patients who underwent elective pancreatic resection at a certified ERAS center between August 2014 and June 2023 were included. Datasets were retrospectively collected from a prospectively maintained ERAS database (ERAS® Interactive Audit System [EIAS]). The impact of individual ERAS components as well as compliance on morbidity, 30-day mortality and hospital stay were assessed by univariate and multivariate analyses.

**Results:**
In total, 667 patients were included (female 44.3%) with a median age of 65 years. Pancreatoduodenectomy, distal and total pancreatectomy were performed in 44.8%, 14.1%, and 17.2%, respectively. A minimal-invasive approach was used in 16.9%. Ninety-three patients (13.94%) achieved a high compliance of ≧70%. High ERAS compliance was associated with a decreased rate of severe complications (Clavien-Dindo >3a) as well as a reduced 30-day mortality rate. In line with this, the duration of hospital stay was significantly shorter in patients with high compliance. In a multivariate analysis, restrictive intravenous fluid administration, early mobilization on the first postoperative day (POD 1), minimal-invasive surgery, and early removal of urinary catheters were associated with a shorter hospital stay. Restrictive intravenous fluid management and early mobilization on POD 1 were independently correlated with fewer complications. Importantly, implication of ERAS items was generally not associated with a significant increase in adverse outcomes.

**Conclusion:**
High ERAS protocol compliance after pancreatic surgery was achieved by less than a fifth of the patients in clinical routine. Early mobilization during the first days following the operation and restrictive fluid management are key indicators for optimized short-term outcome. Thus, studies to evaluate feasibility and effectiveness of specific mobilization protocols are required. However, the study cannot completely exclude that early complications may also bias the extent of mobilization.

**Multidisciplinary tumor boards in liver disease: evaluation in a high-volume center using the German DGAV StuDoQ|Liver registry**
ID: 543

Svenja Sliwinski[1], Franziska Stickl[1], Jan Heil[1], Michael Heise[1], Hanan El Youzouri[1], Wolf Bechstein[1], Ekaterina Petrova[1], Andreas Schnitzbauer[1]

[1]*University Hospital Frankfurt, Goethe University Frankfurt/Main , Department for General, Visceral, Transplant and Thoracic Surgery, Frankfurt am Main, Deutschland*

**Background:**
Individualized patient care is most efficient. Therefore, multidisciplinary boards (MDB) are gold standard in patient care, to discuss all potential options and the evidence behind the treatment. We evaluated quality markers case presentation in tumor boards pre- and postoperatively as well as adhesion to recommendation and impact on postoperative outcome for patients undergoing liver surgery for malign primary and secondary tumors.

**Materials and methods:**
A retrospective evaluation of all patients in the unicentric StuDoQ liver data base was performed. A total of 384 patients with liver disease whose cases were submitted to an MDB discussion between January 2019 and March 2023 were identified. Clinical data, management recommendations, adherence and treatment regimens were reviewed. Correlations with outcomes such as mortality and major postoperative complications according to the Clavien-Dindo (CD) classification were performed. $\chi 2$, univariate and multivariate analyses were undertaken using SPSS.

**Results:**
There were 360 (93.8%) preoperative MDB discussions on 384 patients before liver surgery. Patients with HCC (n=116, 96.7%), CCC (n=86, 94.5%) and colorectal liver metastasis (n=49, 98%) were discussed and received specific recommendation for surgery. Postoperative discussions were partially less frequent (HCC n= 112, 93.3%, CCC n=83, 91.2%, CRLM n=49, 100%).
A preoperative recommendation for induction chemotherapy was given for 33 patients of which 8 (24.2%) preferred to be primarily resected.
Postoperative recommendation for chemotherapy was given in 104 cases (HCC n=20, 20% / CCC/ 73, 80.2% / CRLM n=7, 14%) and the ratio of patients receiving complete treatment was 19.2% (n=20). Factors for deviating from the recommended protocol were rejection by patients (n=10, 11.9%), side effects of chemotherapy (n=14, 16,7%), progressive disease (n=3, 3.6%) and others (n=29, 34.5%). 44 patients (42.3%) started treatment of which only 45% (n=20) finished it.
Univariate analyses revealed higher rates of 90-day readmission, number of reoperations and mortality in patients not receiving postoperative tumor board discussions ($p<0.001$). Regarding quality management factors in liver surgery such as adequate preoperative staging, R0 resection and adequate number of lymph nodes resected (≥6) and pre- as well as postoperative MDB discussions were applicable to 25 (27.47%) cases in the subgroup of CCC and in 97 (80.8%) of HCC cases, accounting for 52.6% (n=202) of malign cases.

**Conclusion:**
Quality standards are essential and tumor board discussions were undertaken in more than 91% of all malign cases. Non-adherence to multidisciplinary tumor board recommendations can have patient-related causes. Non-discussion of cases postoperatively might affect clinical outcomes.

### The Impact of Surgical Timing on Outcome in Uncomplicated vs Complicated Appendicitis - A Retrospective Population-Based Study

ID: 672

Konstantin Lukas Uttinger[1], Daniel Seehofer[1], Armin Wiegering[2]

[1]*Universitätsklinikum Leipzig, VTTG, Leipzig*, [2]*Universitätsklinikum Würzburg, Viszeralchirurgie*

**Background:**
Acute appendicitis is a global disease with high incidence. There is conflicting evidence regarding the association between time from admission to surgery and surgery during emergency hours with operative outcome.

**Materials and methods:**
This is a retrospective population-wide analysis of anonymized hospital billing data (2010-2021) from all admissions for acute appendicitis in Germany. The primary outcome was a composite clinical endpoint (CCE) and mortality by in-hospital surgical delay and surgical timing.

**Results:**
855,694 admissions for acute appendicitis were included, of which 27.6% were complicated cases. 52.2% of cases were operated on within 6 hours, 89.6% within 24 hours. Age, male sex, comorbidity, and time from admission to surgery were associated with the proportion of the observed CCE and in-hospital mortality. Time from admission to surgery was associated with a clinically relevant increase of CCE after 12 hours in complicated appendicitis (OR 1.19, 95% CI 1.14-1.21) and after 24 hours in uncomplicated appendicitis (OR 1.10, 95% CI 1.02-1.19). An increase in mortality was present only in complicated appendicitis and observed after 72 hours (OR, 1.72, 95% CI, 1.38-2.15). Performance of surgery during emergency hours (6 pm – 6.59 am) was associated with an increase of CCE and mortality (OR between 1.14 and 1.49, $p<0.001$-0.014) in complicated and uncomplicated appendicitis.

**Conclusion:**
This work found an increase in CCE and mortality after an in-hospital surgical delay of 12 hours in complicated appendicitis and an increase of CCE after 24 hours in uncomplicated appendicitis. Both CCE and mortality were increased if appendectomy was performed during emergency hours.

Abstracts – DCK 2024 – 141st Congress of the German Society of Surgery – Leipzig, April 24–26 • DOI 10.1515/iss-2024-2003
Innov Surg Sci 2024; 9 (Special Suppl 1): s18–s203

**Short- and long-term outcomes of patients with postoperative arrhythmia after liver surgery**
ID: 880

Felix Rühlmann[1 2], Deborah Engelhardt[1], Alma Franziska Mackert[1], Mara Sophie Hedicke[1], Tobias Tichelbäcker[3], Andreas Leha[4], Markus Bernhardt[1 2], Michael Ghadimi[1], Thorsten Perl[1], Azadeh Azizian[1 2], Jochen Gaedcke[2]

[1]*Universitätsmedizin Göttingen, Klinik für Allgemein-, Viszeral- und Kinderchirurgie, Göttingen, Deutschland*, [2]*Städtisches Klinikum Karlsruhe gGmbH, Klinik für Allgemein- und Viszeralchirurgie, Karlsruhe, Deutschland*, [3]*Uniklinik Köln, Herzzentrum zu Köln, Klinik III für Innere Medizin, Köln, Deutschland*, [4]*Universitätsmedizin Göttingen, Institut für Medizinische Statistik, Göttingen, Deutschland*

**Background:**
New-onset postoperative arrhythmia (PA) has been previously described as a pivotal risk factor for postoperative morbidity and mortality after visceral surgery. However, there is a lack of data concerning liver surgery. The incidence and impact of new-onset postoperative arrhythmia after liver surgery was therefore analysed in a monocentric study.

**Materials and methods:**
In total, $n = 460$ patients (221 female, 239 male) who underwent liver surgery between January 2012 and April 2020 without any prior arrhythmia in their medical history were included in this retrospective analysis. Clinical monitoring started with the induction of anaesthesia and was terminated with discharge from the intensive care unit (ICU) or intermediate care unit (IMC). Follow-up included documentation of complications during the hospital stay as well as long-term survival analysis.

**Results:**
Postoperative arrhythmia after liver surgery was observed in 25 patients, corresponding to an incidence of 5.4%. The occurrence of arrhythmia was significantly associated with intraoperative complications ($p < 0.05$), liver fibrosis/cirrhosis ($p < 0.05$), bile fistula/bile leakage/bilioma ($p < 0.05$), and organ failure ($p < 0.01$). Survival analysis showed a significantly poorer overall survival of patients who developed postoperative arrhythmia after liver surgery ($p < 0.001$).

**Conclusion:**
New-onset postoperative arrhythmia after liver surgery has an incidence of only 5.4% but is significantly associated with higher postoperative morbidity and poorer overall survival.

**Laparoscopic ischemic conditioning prior esophagectomy in selected patients: Clinical and translational results of the prospective ISCON trial**
ID: 102

Lars Schiffmann[1], Eline de Groot[2], Daniel Pinto Dos Santos[3], Benjamin Babic[1], Alexander Quaas[4], Hans Fuchs[1], Seung-Hun Chon[1], Jelle Ruurda[2], Hamid Kashkar[5], Richard van Hillegersberg[2], Christiane J. Bruns[1], Wolfgang Schröder[1]

[1]*Klinik und Poliklinik für Allgemein-, Viszeral-, Tumor- und Transplantationschirurgie, Uniklinik Köln, Köln, Deutschland*, [2]*Department of Surgery, University Medical Center Utrecht*, [3]*Institut für diagnostische und interventionelle Radiologie , Uniklinik Köln*, [4]*Institut für Pathologie*, [5]*Institut für molekulare Immunologie, Universität zu Köln*

**Background:**
Patients with calcifications of the aorta or a stenosis of the coeliac trunc have an increased risk of anastomotic leakage (AL) following Ivor-Lewis esophagectomy. Ischemic conditioning (ISCON) of the stomach prior to esophagectomy is thought to advance gastric microcirculation at the anastomotic site. The prospective ISCON trial (NCT03896399) was initiated to proof the safety and feasibility of laparoscopic ISCON with partial gastric devascularization 14 days prior esophagectomy. We here report promising clinical and translational results of this trial.

**Materials and methods:**
Twenty esophageal cancer patients with major calcifications of the thoracic aorta or a stenosis in the celiac trunk were included in the ISCON trial. Patients underwent laparoscopic ISCON by occlusion of the left gastric and the short gastric arteries followed by esophagectomy after an interval of 12–18 days. The primary endpoint was complications Clavien-Dindo ≥ grade 2 after ISCON. Biopsies of the fundus were taken before and after ISCON. Vascularization of the gastric mucosa was assessed to detect the degree of gastric vascularity.

**Results:**
None of the 20 patients developed intraoperative or postoperative complications after ISCON. Median hospital stay after ISCON was 2 days (range: 2–4 days). 30-day and 90-day in-hospital mortalities were 0%. All patients underwent esophagectomy (median time to operation post ISCON 14 days). 3 Patients (15 %) experienced an anastomotic leak after esophagectomy.
ISCON biopsies of the gastric mucosa demonstrated a significant increase in microvascular density after ISCON as compared to the corresponding gastric sample before the intervention (mean area density CD34 1.970 ± 0.1619 SEM vs. mean 3.427 ± 0.3824 SEM, p=0.0020).
We furthermore found that the three patients in our collective that experienced an anastomotic leakage had a significantly less ‘angiogenic response’ upon ISCON compared to patients’ without anastomotic complications (mean -99.22 ± 68.73 SEM vs. mean 266.7 ± 80.28 SEM, p= 0.0329).

**Conclusion:**
We can demonstrate that laparoscopic ISCON is a safe procedure that induces significant neo-angiogenesis in the gastric conduit suggesting an improved vascularization at the anastomotic site. Further trials have to demonstrate whether these findings translate into a significant reduction in anastomotic leakage in these patients.

**Gut Brush Border Enzyme Intestinal Alkaline Phosphatase as biomarker to predict outcome in patients undergoing abdominal surgery**
ID: 438

Viktor von Ehrlich-Treuenstätt[1], Michael Ingrisch[2], Clara Kolbe[1], Ulrich Wirth[1], Jens Werner[1], Wolfgang Kunz[2], Florian Kühn[1]

[1]*LMU München, Klinik für Allgemein-, Viszeral- und Transplantchirurgie*, [2]*LMU München, Klinik für Radiologie*

**Background:**
Sarcopenia is associated with a worse outcome in patients undergoing abdominal surgery. To assess body composition, new A.I.-based CT evaluation tools (Body Composition Analysis) show increasing accuracy in identifying patients with sarcopenia. The brush border enzyme intestinal alkaline phosphatase (IAP) is an important regulator of gut barrier function, intestinal homeostasis, and prevents endotoxemia by detoxifying lipopolysaccharides (LPS). IAP-deficiency has recently been linked to various pathologies such as diabetes, metabolic syndrome, frailty, and a decreased life span, while supplementing the naturally occurring enzyme has been proven beneficial in several studies.
This study aimed to investigate the role of IAP as a potential biomarker for frailty and postoperative outcome in patients undergoing abdominal surgery.

**Materials and methods:**
We conducted a prospective cohort study to investigate the association between IAP, LPS, sarcopenia, surgical outcomes, and overall survival in patients undergoing abdominal oncologic surgery. A total of 54 patients were enrolled, pre- and postoperative IAP and LPS levels were measured in stool and blood samples, along with clinical parameters for multivariate analysis. The diagnosis of sarcopenia was based on the skeletal muscle mass of L3 measured by a CT-based A.I. Body Composition Analysis.

**Results:**
Our results showed that preoperative IAP levels were significantly lower in patients with sarcopenia (p=0.0323) and old age (p=0.0205), with preoperative LPS showing a tendency to be higher in sarcopenic patients. Patients with low preoperative IAP activity had significantly higher postoperative LPS values (p=0.0196) with higher morbidity in surgical outcomes (Clavien-Dindo III-IV; Organ space infection), lower overall survival (p=0.0010) and an elongated hospitalization than those with regular preoperative IAP activity.

**Conclusion:**
In conclusion, our study highlights the potential role of IAP as a potential biomarker for sarcopenia and postoperative outcome in patients undergoing oncologic surgery.

**Correlation of weekday and readmissions to ICU after robotic assisted minimally invasive surgery (RAMIE)**
ID: 75

Dominique Hart[1], Dolores Thea Müller[2], Hendrik Drinhaus[1], Sandra Stoll[1], Fabian Dusse[1], Stefanie Brunner[2], Jennifer Eckhoff[2], Thomas Schmidt[2], Lars Schiffmann[2], Wolfgang Schröder[2], Christiane J. Bruns[2], Wolfgang A. Wetsch[1], Hans Fuchs[2]

[1]*Klinik für Anästhesiologie und Operative Intensivmedizin , Universität zu Köln, Medizinische Fakultät und Uniklinik Köln, Köln*, [2]*Klinik und Poliklinik für Allgemein-, Viszeral-, Tumor- und Transplantationschirurgie, Universität zu Köln, Medizinische Fakultät und Uniklinik Köln, Köln*

**Background:**
RAMIE has recently shown to significantly reduce postoperative mortality and morbidity in patients undergoing esophagectomy and therefore improving short term outcomes. However, while parameters like pneumonia and anastomotic leak rates have been assessed before, the literature lacks an analysis of the impact of weekday of surgery on length of stay in the intensive care unit (ICU-LOS) and readmission rates to ICU. Aim of this study was to analyze the correlation between weekday of surgery, ICU-LOS and readmission rates to ICU after RAMIE at a high-volume center.

**Materials and methods:**
A retrospective analysis of our dataset of all patients undergoing RAMIE was performed at our European High-Volume Center from 04/2017 – 12/2022. Patients with an inhospital mortality or ICU-LOS > 100 days were excluded from the analysis. We defined ICU-LOS (in days) as count of nights spent in an intensive care unit. A comparative analysis with Chi-Square Test and one-way analysis of variance was performed.

**Results:**
A total of 197 patients were included in the analysis. Mean postoperative ICU-LOS was 2.8 (SD 2.3). Longest ICU-LOS was seen for surgery performed on Wednesdays with a mean of 3.4 days (SD 2.8 days), with was a statistically significant correlation (p = 0.003). Details are depicted in table 1, figure 1. ICU readmission was necessary in 18.8% of all cases (n= 37 patients) and did not significantly correlate with neither the first ICU-LOS (p = 0.73) nor weekday of surgery (p = 0.42). Further details are depicted in table 2.

**Conclusion:**
Even though standardized protocols are in place for this procedure, there seems to be an association between ICU-LOS and weekday of surgery. Further analysis of factors contributing to weekday differences is needed and the effect of weekdays and clinical decisions is yet unclear. Interestingly, no correlation between weekday and readmission rates to ICU or ICU-LOS and readmission was observed

**ICU-LOS in days**

| Weekday of surgery | Mean | N | Standard-Deviation | Median | Minimum | Maximum | % of total Count |
|---|---|---|---|---|---|---|---|
| Monday | 2.27 | 26 | 1.538 | 2.00 | 1 | 7 | 13.2% |
| Tuesday | 2.86 | 64 | 2.811 | 2.00 | 1 | 22 | 32.5% |
| Wednesday | 3.44 | 45 | 2.751 | 2.00 | 2 | 14 | 22.8% |
| Thursday | 2.33 | 33 | 1.633 | 2.00 | 1 | 10 | 16.8% |

| | | | | | | | |
|---|---|---|---|---|---|---|---|
| Friday | 2.66 | 29 | 1.111 | 3.00 | 1 | 6 | 14.7% |
| Total | 2.80 | 197 | 2.308 | 2.00 | 1 | 22 | 100.0% |

Table 1: Correlation of weekday of surgery and length of hospital stay.
Chi-Square-Test by Monte-Carlo Method: p = 0.003

**Readmission weekday-correlated**

| | | Monday | Tuesday | Wednesday | Thursday | Friday | |
|---|---|---|---|---|---|---|---|
| Readmission | no | 21 | 50 | 41 | 26 | 22 | 160 |
| | yes | 5 | 14 | 4 | 7 | 7 | 37 |
| Total | | 26 | 64 | 45 | 33 | 29 | 197 |

Table 2. Correlation of weekday of surgery and readmission rates to ICU.

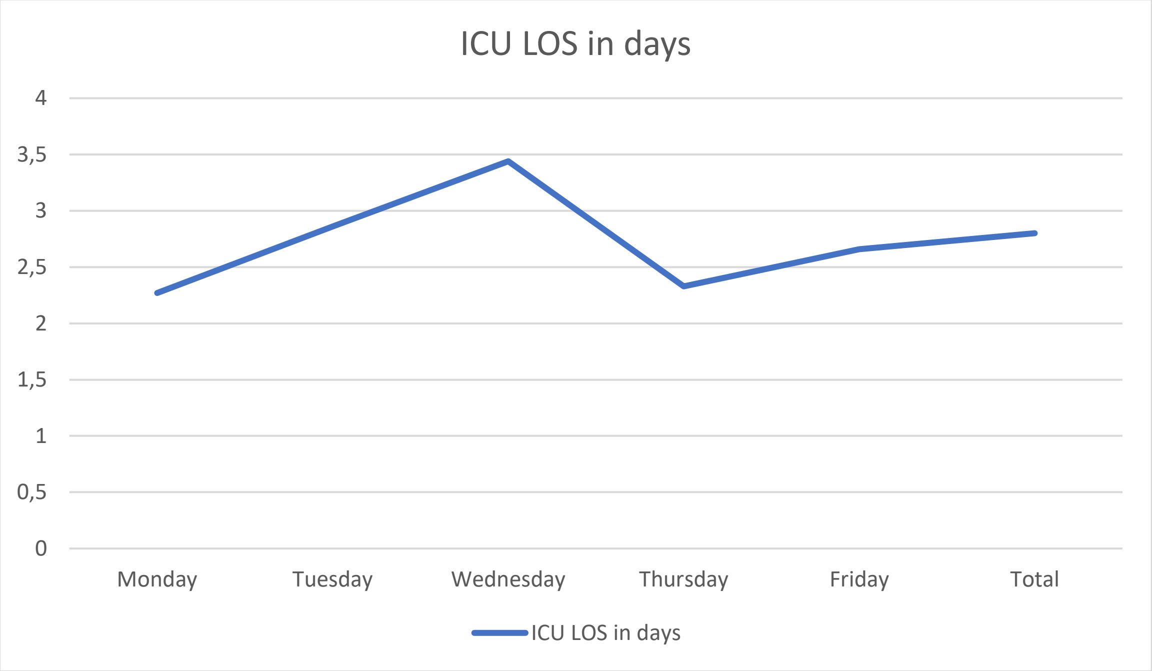

**Profile of a perfect outcome (Clavien Dindo 0) after robotic assisted minimally invasive Ivor-Lewis esophagectomy (RAMIE)**
ID: 76

Dolores Thea Müller[1], Stefanie Brunner[1], Jennifer Eckhoff[1], Christian Storms[1], Alissa Reisewitz[1], Yinan Xu[1], Thomas Schmidt[1], Wolfgang Schröder[1], Christiane J. Bruns[1], Hans Fuchs[1]

[1]*Klinik und Poliklinik für Allgemein-, Viszeral-, Tumor- und Transplantationschirurgie, Universität zu Köln, Medizinische Fakultät und Uniklinik Köln, Köln*

**Background:**
RAMIE has recently shown to improve short-term and some oncological outcomes for patients. However, it is yet unclear, which patients are prone to develop complications and studies have yet to identify who is safe to be discharged early with a textbook outcome.

**Materials and methods:**
A retrospective analysis of our prospectively collected dataset of all patients undergoing RAMIE at our High-Volume Center (annual volume > 250 upper GI cancer cases) from 04/2017–01/2023 was performed. Patients were grouped according to their postoperative outcome as either perfect outcome (Clavien Dindo 0) or complication (Clavien Dindo □ 1). A comparative analysis was performed.

**Results:**
A total of 180 patients were included in the analysis. Table1 shows the demographic characteristics of both groups. Increased complications were seen for female sex (p=0.0427). No differences were seen for other characteristics. Table2 shows further details of patients with a perfect outcome. Duration of surgery(p=0.6643) and single lung ventilation(p=0.7355) did not differ between groups. In patients with complications, first onset of complications was seen at a median of day 3 (range0 -12). From patients that developed late complications(>POD 6), only 1 had an anastomotic leak, while all others presented with a delayed gastric conduit emptying.

**Conclusion:**
Females showed a significant higher chance for developing postoperative complications after RAMIE. Furthermore, POD6 seems to play a crucial role in the clinical course of patients. Onset of severe complications was seen before POD6 in all but one patient while patients with a perfect outcome started oral feeding on POD6, had chest drains and peridural catheters removed. Further analysis has yet to reveal if this finding can be used to identify patients who are safe to be discharged early.

*Table 1: Comparison of demographic characteristics between patients with a textbook outcome and ones with any complication.*

| | Clavien Dindo 0 | Clavien Dindo > 0 | |
|---|---|---|---|
| | *N (%) / mean (range)* | *N (%) / mean (range)* | *P value* |
| **N** | 72 | 108 | - |
| **Age (years)** | 60.8 (36 - 79) | 62.8 (37 - 82) | 0.1391 |
| **Female** | 7 (9.7) | 24 (22.2) | 0.0427 |
| **BMI (kg/m²)** | 25.48 (18.94 - 35.35) | 25.7 (15.62 - 42.25) | 0.6768 |
| **Neoadjuvant Treatment** | | | |
| **CROSS** | 35 (48.6) | 52 (48.1) | 1 |
| **FLOT** | 26 (36.1) | 39 (36.1) | 1 |
| **None** | 11 (15.3) | 17 (15.7) | 1 |

| Preoperative Comorbidities | | | |
|---|---|---|---|
| **hepatic** | 8 (11.1) | 4 (3.7) | 0.0678 |
| **Kidney** | 2 (2.8) | 6 (5.6) | 0.4790 |
| **Cardiac** | 12 (16.7) | 23 (21.3) | 0.5647 |
| **Vascular** | 35 (48.6) | 56 (51.9) | 0.7611 |
| **Pulmonary** | 6 (8.3) | 11 (10.2) | 0.7976 |
| **Diabetes** | 8 (11.1) | 10 (9.3) | 0.8008 |
| **Pathology** | | | |
| **Squamous cell carcinoma** | 12 (16.7) | 16 (14.8) | 0.8343 |
| **Adenocarcinoma** | 58 (80.6) | 91 (84.3) | 0.5497 |
| **Other/Benign** | 2 (2.8) | 1 (0.93) | 0.5648 |
| **T0** | 12 (16.7) | 28 (25.9) | 0.1998 |
| **T1a/b** | 13 (18.1) | 18 (16.7) | 0.8420 |
| **T2** | 14 (19.4) | 14 (13) | 0.2948 |
| **T3** | 31 (43.1) | 41 (38) | 0.5362 |
| **T4** | 1 (1.4) | 6 (5.6) | 0.2454 |
| **Benign/unknown** | 1 (1.4) | 1 (0.93) | 1 |
| **N0** | 40 (55.6) | 63 (58.3) | 0.7595 |
| **N1** | 14 (19.4) | 22 (20.4) | 1 |
| **N2** | 11 (15.3) | 11 (10.2) | 0.3560 |
| **N3** | 6 (8.3) | 12 (11.1) | 0.6192 |
| **R0** | 67 (93) | 104 (96.3) | 0.4871 |
| **R1** | 5 (7) | 4 (3.7) | 0.4871 |

*Table 2: Intraoperative and postoperative characteristics of clinical course of patients with a textbook outcome after robotic assisted minimally invasive esophagectomy.*

| | **Clavien Dindo 0** |
|---|---|
| | *N (%) / mean (range)* |
| **N** | 72 |
| **Intraoperative parameters** | |
| **Duration of Surgery (min)** | 371 (235 – 615) |
| **Single lung ventilation (min)** | 182.9 (90 – 317) |
| **Postoperative parameters** | |
| **Length of ICU stay (days)** | 2 (1 – 5) |
| **Length of hospital stay (days)** | 11.7 (9 – 17) |
| **Begin oral feeding* (POD)** | 6 (4-8) |
| **Peridural catheter*** | 60 (96.8) |
| **Removal of peridural catheter* (POD)** | 6 (1 – 12) |
| **Removal of right chest drain* (POD)** | 6 (3 – 9) |

| | |
|---|---|
| **Left chest drain*** | 11 (17.4) |
| **Postoperative test results** | |
| **CRP level POD 3** | 103.9 (20 – 260) |
| **CRP level POD 5** | 67.9 (15 – 196) |
| **CRP level POD 7** | 41.7 (10 – 131) |

*Data only available for n=62 patients

### Evaluation of Ergonomics and Surgeon's Stress Levels during Robotic Assisted Minimally Invasive Esophagectomy – The ERASE Trial

ID: 83

Dolores Thea Müller[1], Juliane Ahn[2], Stefanie Brunner[1], Jennifer Eckhoff[1], Christian Storms[1], Julia Poggemeier[1], Thomas Schmidt[1], Wolfgang Schröder[1], Michael Thomas[1], Carolin Höfler[2], Christiane J. Bruns[1], Hans Fuchs[1]

[1]*Klinik und Poliklinik für Allgemein-, Viszeral-, Tumor- und Transplantationschirurgie, Universität zu Köln, Medizinische Fakultät und Uniklinik Köln, Köln*, [2]*Fakultät für Kulturwissenschaften, Köln International School of Design, Technische Hochschule Köln*

**Background:**

Although often underestimated especially in a surgical work environment, ergonomics are essential for endurance, efficiency, and a long and healthy work life. An efficient and ergonomic design of the operating room has been widely discussed and guidelines have been imposed to create the optimal workspace. The ERASE trial(Ergonomics in Robotic ASissted minimally invasive Esophagectomy)aims to analyze the surgeon's posture in combination with the intraoperative subjective and objective stress level identifying ergonomic requirements in the robotic operating room.

**Materials and methods:**

A variety of validated intraoperative measurements are applied. Heart Rate Variability (HRV)is recorded with a chest band and corresponding sensor. A stress index, parasympathetic and sympathetic nervous system index (PNS, SNS) are calculated using specific software (Kubios Oy, Finnland) Fig 1. For subjective evaluation of stress level, surgeons are asked to fill out a short form of the State-Trait-Anxiety-Inventory(STAI) questionnaire. For evaluation of posture, a camera is used to capture the surgeon at the console during surgery. An image analyses can be created showing average posture of the surgeon.A touch sensor is attached to the surgeon console measuring if and how long the arm rest is used. Furthermore, a sound level measurement is performed (Fig2). Video recordings of the endoscopic camera are obtained and synchronized with the data.

**Results:**

From 10/22 – 08/23 four surgeons were included in the study and n = 7 measurements were performed during robotic assisted minimally invasive esophagectomy. Mean operating time for the thoracic part was 159 min (range 127 – 201min). Mean heart rate was 88.5/min with a mean minimum heart rate of 57/min and a mean maximum heart rate of 120/min. Complex tasks such as the anastomotic phase and use of a stapling device did not show a significant increase in heart rate. Mean stress index was 2.4, mean PNS index was 3.91 (range 3.45 - 4.37), mean SNS index was 0.06 (range 0.01-0.1). Mean LF/HF ratio was 0.785. An increased heart rate was seen during bleeding and non-console time. Analysis of touch sensor data revealed a use of the arm rest of 74.6 % of the time. The left arm rest was used in 69.4% of the time compared to 79.8% for the right arm rest (Fig3).

**Conclusion:**

Surgical ergonomics are an important aspect of the surgeon's everyday work life, however, no validated and standardized measurements evaluating ergonomics in the operating room exist. Preliminary results of our study revealed the need for improvement of ergonomics. The ERASE trial is currently recruiting and further analysis and application of the presented methods to future cases of robotic assisted minimally invasive esophagectomy will be performed.

Abstracts – DCK 2024 – 141st Congress of the German Society of Surgery – Leipzig, April 24–26 • DOI 10.1515/iss-2024-2003
Innov Surg Sci 2024; 9 (Special Suppl 1): s18–s203

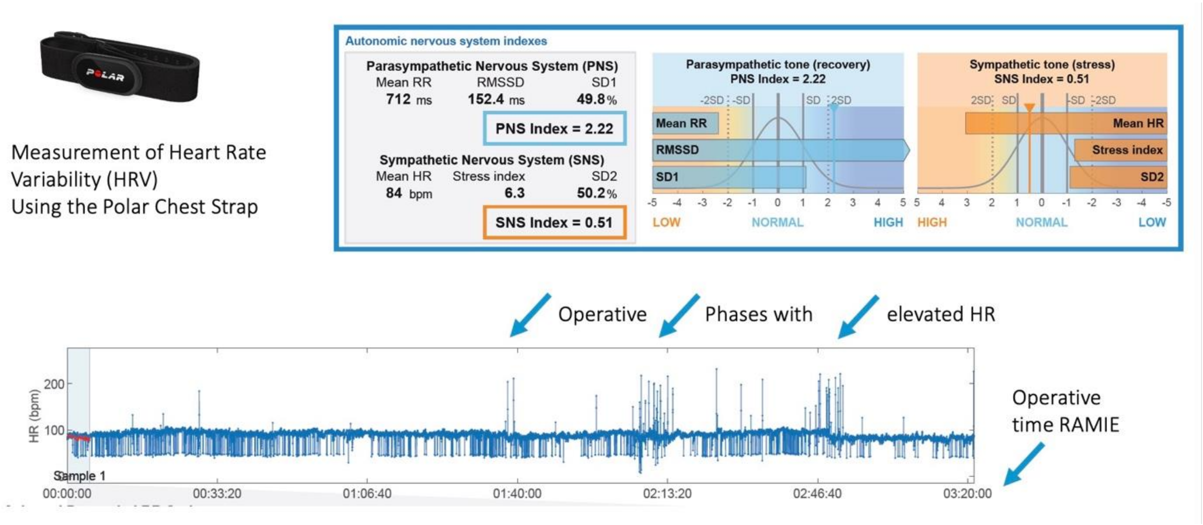

Figure 1: Objective evaluation of stress level and heart rates during RAMIE

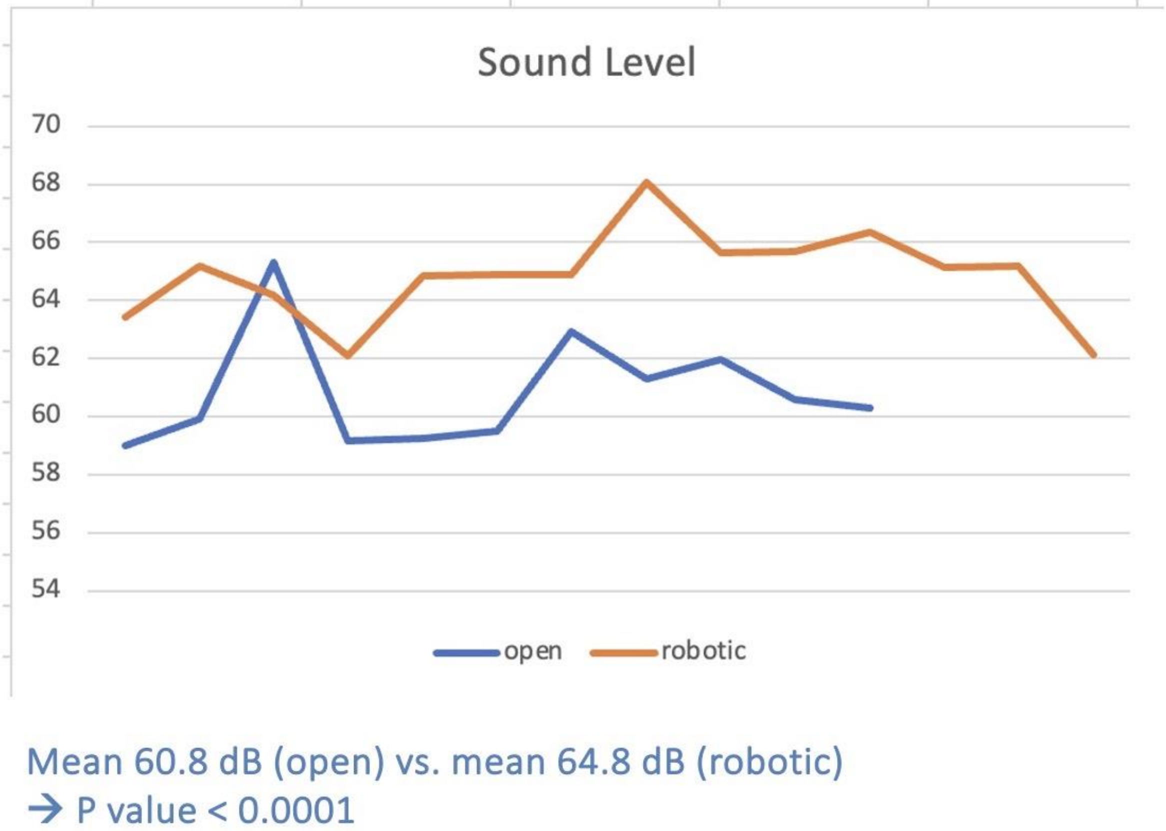

Figure 2: Statistically significant differences were found in the sound level when comparing open to robotic assisted minimally invasive esophagectomy.

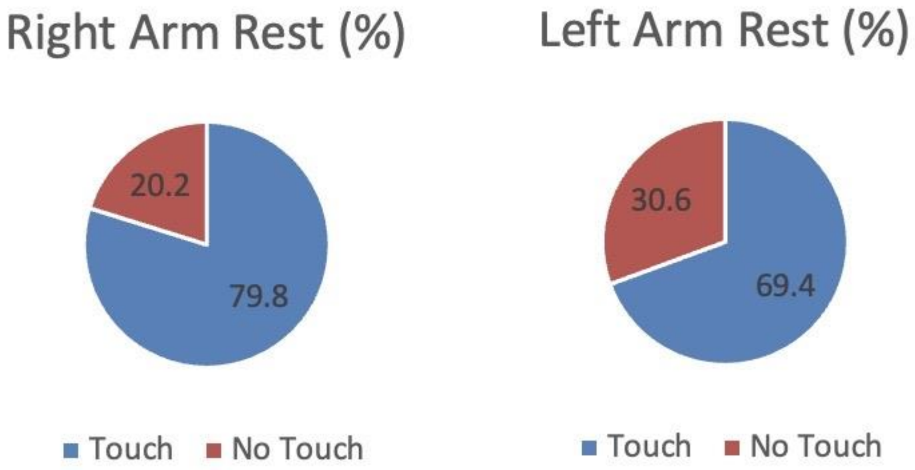

Figure 3: Differences in the use of arm rest between the left and right arm rest were observed.

**The Dresden Surgical Anatomy Dataset - Semantic segmentations of abdominal structures for AI-based object detection**
ID: 101

Matthias Carstens[1], Franziska Maria Rinner[1], Sebastian Bodenstedt[2], Alexander Jenke[2], Jürgen Weitz[1], Marius Distler[1], Stefanie Speidel[2], Fiona Kolbinger[1]

[1]*Universitätsklinikum Carl Gustav Carus Dresden, Klinik für Viszeral-, Thorax- und Gefäßchirurgie , Dresden, Deutschland*, [2]*NCT Dresden, Department of Translational Surgical Oncology (TSO)*

**Background:**
Laparoscopic surgery is a widely used technique for minimally-invasive and robot-assisted procedures, offering advantages like shorter hospital stays, reduced blood loss, faster recovery and enhanced surgical visualization. The rapidly developing methods and techniques provided by the usage of AI (Artificial Intelligence), more precisely the automated recognition of instruments, organs and other anatomical structures in laparoscopic images or videos, have the potential to make surgical procedures safer and faster. Open-source laparoscopic image datasets are limited, and existing datasets mostly comprise image-level annotations that allow the user to differentiate if a structure of interest is shown in an image without giving information about its specific spatial location and appearance. However, such labor-intensive pixel-wise annotations are required for a variety of machine learning tasks for image recognition in the context of surgical data science. The Dresden Surgical Anatomy Dataset provides 13195 pixel-wise annotated and weak-labelled images of abdominal structures to address this gap.

**Materials and methods:**
Based on temporally annotated video data from 32 robot-assisted rectal resections or extirpations, a total of 13195 images displaying different intraabdominal organs (colon, liver, pancreas, small intestine, spleen, stomach, ureter, vesicular glands) and anatomical structures (abdominal wall, inferior mesenteric artery, intestinal veins) with at least 1000 images from at least 20 different patients for each organ or anatomical structure were extracted. For a realistic representation of common laparoscopic obstacles, it features various levels of organ visibility including small or partly covered organ parts, motion artefacts, inhomogeneous lighting and smoke or blood in the field of view. Images were stored in PNG format at a resolution of 1920 × 1080 pixels.
For pixel-wise segmentation *3D Slicer* was used. Each individual image was semantically annotated according to a guideline by three medical students with basic experience in minimally-invasive surgery. One specific anatomical structure was segmented in each image. Subsequently, the three individual annotations were automatically merged using the *STAPLE* algorithm and individual annotations alongside merged segments were reviewed and adjusted by a physician with three years of experience in minimally-invasive surgery. In addition, a subset was annotated with respect to the exact location of all visible organs (multi-organ-segmentation).
Weak labels providing information about the visibility of different anatomical structures in the entire image were annotated by one medical student with basic experience in minimally-invasive surgery and reviewed by a second one in each of the 13195 images.

**Results:**
In total, the dataset comprises 13195 images from 32 robot-assisted surgeries with semantic segmentations (colon: 1374, liver: 1023, pancreas: 1173, small intestine: 1168, spleen: 1191, stomach: 1430 single plus multi-organ segmentation, ureter: 1275, vesicular glands: 1198, abdominal wall: 1206, inferior mesenteric artery: 1090 and intestinal veins: 1067). These annotations are provided as two versions of the PNG-files: one raw image that has been extracted from the surgery video file and one image that contains the mask of the expert-reviewed semantic segmentation.
Weak labels of organ visibility are provided for all images in CSV format.

**Conclusion:**

Adding anatomical knowledge to laparoscopic data, this dataset bridges a major gap in the field of surgical data science and serves as a basis for a variety of machine learning tasks in the context of image recognition-based surgical assistance functions. Potential applications include the development of smart assistance systems through automated segmentation tasks, the establishment of unsupervised learning methods, or registration of preoperative imaging data (e.g. CT, MRI) with laparoscopic images for surgical navigation.

Abstracts – DCK 2024 – 141st Congress of the German Society of Surgery – Leipzig, April 24–26 • DOI 10.1515/iss-2024-2003
Innov Surg Sci 2024; 9 (Special Suppl 1): s18–s203

**Surgeon, take care of yourself, so you can care for others: Exploring stress entities with the robotic Senhance Surgical System**
ID: 129

Vivianda Menke[1], Olaf Hansen[1], Bernhard Uhl[1], Nicole Sigrist-Uhl[1], Johannes Schmidt[2], Ludger Staib[3], Francesco Bianco[4], Zeljko Kastelan[5], Tomislav Kulis[5], Narimantas Samalavicius[6]

[1]*Evangelisches Krankenhaus Wesel GmbH, Wesel, Deutschland*, [2]*Lakumed Kliniken, Krankenhaus Landshut-Achdorf, Landshut, Deutschland*, [3]*Klinikum Esslingen, Esslingen, Deutschland*, [4]*University of Illinois at Chicago, Chicago, United States*, [5]*University hospital centre Zagreb, Department of Urology, Zagreb, Croatia*, [6]*Klaipeda University Hospital, Department of Surgery, Urology and Gynaecology, Klaipeda, Lithuania*

**Background:**
The era of robotic surgery is now. The next step is to show the advantages of robotic surgery. This is a difficult task. While robot assisted surgery offers precise and minimally invasive techniques, it was hypothesized to shorten recovery times and improve patient outcomes. However, large studies that compared laparoscopy to robotic assisted surgery did not find the significance that we expected. While there have been multiple publications on robotic systems' medical and procedural achievements, more emphasis should be put on the surgeon's experience, especially in comparison with laparoscopic surgery. By combining the precision of robotic systems with the dexterity of skilled surgeons, the technology of the Senhance Surgical System (Asensus Surgical, Durham, North Carolina, U.S.A) has opened new possibilities for minimally invasive surgeries. Technological advancements have brought numerous patient benefits (Muaddi H, et al. Clinical Outcomes of Robotic Surgery Compared to Conventional Surgical Approaches (Laparoscopic or Open): A Systematic Overview of Reviews. Ann Surg 2021; 273(3):467–73), and they have also introduced unique changes that benefits the surgeons' well-being. The present report aims to systematically examine the stress impact on surgeons by comparing the robotic Senhance Surgical System to laparoscopic surgery.

**Materials and methods:**
The well-established "SURG-TLX" survey is used to measure distinct stress entities. The "SURG-TLX" survey is a modified version of the NASA-TLX, validated for surgery by M. Willson. Surgeons who conducted procedures in general surgery (Cholecystectomy, Inguinal Hernia (Uni- and Bilateral), Right Hemicolectomy, Left-Sided Colorectal Resections, Fundoplication, Sleeve gastrectomy, Hiatal Hernia), urology (Radical Prostatectomy), and gynecology (Total Hysterectomy) with the Senhance Surgical System or via laparoscopic surgery were asked to answer the "SURG-TLX" survey questionnaire (see below) within 15 min after completing surgery. Data was collected from senior surgeons in six centers, including the Evangelisches Hospital Wesel, Wesel, Germany; Hospital Landshut-Achdorf, Landshut, Germany; Hospital Esslingen, Esslingen, Germany; University Hospital Center Zagreb, Zagreb, Croatia; Klaipeda University Hospital, Klaipeda, Lithuania; Mount Sinai Hospital, Chicago, IL, US. The "SURG-TLX" questionnaire consists of three parts. The first part defines the procedure, procedural method (robotic or laparoscopic surgery), and surgeon's role during procedure (laparoscopic, (robotic) bedside, or (robotic) cockpit), as well as identifies the surgeon. In the second part, the surgeon rates overall situational stress experience in its various entities. Stress levels of six dimensions are indicated on a rating scale ranging from 0 to 100, with 0 meaning non-existing stress and 100 representing maximal stress. Each entity stands alone, describing distinct situations, and is evaluated individually. In the third part, all six categories are paired with each of the other categories, which lead to 15 questions in total, displaying the paired superiority questionnaires. The superiority number (0-5) for each stress entity is multiplied by the rating scale (0-100). Therefore, results can range from 0 to 500. For the "overall stress" score, the results of all the 15 multiplications are added and divided by 15. Besides presenting the overall outcome, we compared stress categories by methods per distinct procedures. Finally, we also compared differences in stress scores between surgeon positions (laparoscopic vs. (robotic) bedside and laparoscopic vs. (robotic) cockpit) to further explore differences in stress experience and exposure. Statistics were performed by a senior statistician. The parametric t-test was used for statistical analysis, and results were displayed as mean and standard deviation. Statistical relevance was defined as p-value ≤ 0.05.

**Results:**
Our analysis was performed on 350 "SURG-TLX" questionnaires. 287 surgeries were performed with the Senhance Surgical System, while 63 were operated via laparoscopic surgery. Regarding the surgeon’s role in robotic surgery, 97 questionnaires were obtained from a bedside role and 190 from a cockpit surgeon. Comparing both methods, our results presented no significant difference in the “Overall Stress” level between robotic (cockpit and bedside surgeon) and laparoscopic (main and assistant surgeon) surgery (robotic vs. laparoscopic: p.value = 0.427). However, mental demands were significantly higher in robotic surgery compared to laparoscopic surgery (robotic vs. llaparoscopic: p.value < 0.001), while physical demands were reduced considerably in robotic surgery (robotic vs. laparoscopic: p.value < 0.001). Regarding the stress dimensions for a radical prostatectomy, physical demands (robotic vs. laparoscopic: p.value = 0.006) and distraction (robotic vs. laparoscopic: p.value ≤ 0.001) presented significantly reduced scores in robotic surgery. Additionally, we explored experienced stress between laparoscopic, (robotic) bedside, and (robotic) cockpit surgeons. The “overall stress” score showed, cockpit surgeons experienced significantly less stress than laparoscopic surgeons (cockpit vs. laparoscopic: p.value = 0.018). Mental demands indicated significantly higher scores (cockpit vs. laparoscopic: p.value= 0.015), while physical demands were reduced (cockpit vs. laparoscopic: p.value < 0.001) in cockpit surgeons compared to laparoscopic surgeons. Further, reduced distraction for robotic surgery reached significance when comparing only cockpit and laparoscopic surgeons (cockpit vs laparoscopic: p.value = 0.0270).

**Conclusion:**
This finding suggests that robotic surgery with the Senhance Surgical System contributes to a favorable stress profile for cockpit surgeons. Overall, the combination of physical separation, ergonomic seating, and 3D vision with an eye-tracking camera may collectively contribute to a more focused and concentrated surgical experience, thereby reducing physical and distraction-related stress. However, we did not explore reasons for reduced physical stress in robotic surgery. Therefore, larger studies examining physical stress and ‘level of comfort’ are needed. In conclusion, we recommend conducting larger surveys with independent participants to gain a more comprehensive understanding of the relationship between surgeons’ stress and robotic surgery, as well as the differences between robotic vs. laparoscopic and even robotic vs. open surgery.

**Data-Driven Pancreatic Surgery: IMI-EDC - Paving the Way for AI Analysis**
ID: 237

Thomas Pausch[1], Max Blumenstock[2], Christin Tjaden[1], Christian Niklas[2], Ulrike Heger[1], Nathalia Giese[1], Rosa Klotz[1], Martin Loos[1], Martin Dugas[2], Christoph Michalski[1]

[1]*Universitätsklinikum Heidelberg, Allgemein-, Viszeral- und Transplantationschirurgie, Heidelberg, Deutschland*, [2]*Universitätsklinikum Heidelberg, Institut für Medizinische Informatik, Heidelberg, Deutschland*

**Background:**
Modern healthcare relies on sophisticated data analytics, with Electronic Data Capture (EDC) and Electronic Health Records (EHR) systems at the forefront. We present the pilot study of the EDC/EHR-system IMI-EDC, implemented in the European Pancreas Centre (EPC) at Heidelberg University Hospital. IMI-EDC aims to seamlessly integrate clinical data for upcoming AI analyses.

**Materials and methods:**
We assessed IMI-EDC's implementation, focusing on data readiness for AI research. We compared patient comfort, data quality, and workflow between IMI-EDC and the previous paper-based system of health records. EPC outpatients submitted medical history and quality of life survey (EORTC-QLQ-C30) using IMI-EDC via tablets. Additionally, we compared 100 IMI-EDC submissions with 100 paper submissions for data completeness, correctness, and clarity. Data transfer times to a scientific SQL database were compared between eight IMI-EDC and seven paper submissions.

**Results:**
IMI-EDC seamlessly integrated into the clinical workflow. Tablet users demonstrated higher comfort and submitted more complete documents (98/100) compared to paper users (66/100). Data transfer to the scientific database was significantly faster with IMI-EDC (4.2 mins) than with paper (9.9 mins). These successes prompt an expansion of the system throughout outpatient and inpatient surgery.

**Conclusion:**
IMI-EDC's success in ensuring data completeness and efficiency in clinical data transfer positions it as a vital tool for upcoming AI analyses. It enables single-source “triple-flow” data science into clinical surgery, research, and quality control. Its scalability within the hospital ecosystem offers the promise of streamlined data preparation for advanced AI research.

**A feasibility study on measuring bound attention during planning of a complex liver surgery**
ID: 242

Tim Schneider[1], Timur Cetin[1], Stefan Uppenkamp[2], Dirk Weyhe[1], Thomas Muender[3], Anke Reinschluessel[3], Daniela Salzmann[1], Verena Uslar[1]

[1]*Carl von Ossietzky Universität, Universitätsklinik für Viszeralchirurgie, Oldenburg, Deutschland*, [2]*Carl von Ossietzky Universität, Department für Medizinische Physik und Akustik, Oldenburg, Deutschland*, [3]*Universität Bremen, Digital Media Lab, Bremen, Deutschland*

**Background:**
Modern surgery has seen the emergence of innovative techniques aimed at minimizing invasiveness to enhance patient safety, exemplified by minimally invasive procedures (Dick & Gebhard, 2015). Nevertheless, these approaches impose greater cognitive and physical demands on the surgical team, particularly the surgeon, due to reduced visibility and increased physical strain, potentially compromising patient safety (Bogdanova et al., 2016).
One example for technological advance in surgery involves Augmented Reality (AR) and Virtual Reality (VR) simulations, which offer 3D models of anatomical structures for examination and analysis. These simulations, projected through glasses, aid preoperative planning.
To integrate AR and VR technologies effectively into clinical practice, their safety, ergonomics, and impact on workplace strain must be thoroughly evaluated (Kramme, 2011). While past assessments of AR and VR glasses have primarily relied on subjective measures like the NASA-TLX questionnaire or performance metrics (Funk & Schmidt, 2021), our study seeks to objectively measure mental workload through electrophysiological recordings and compare them with subjective assessments.
From a neuroergonomic perspective, replacing subjective questionnaires with objective measurements is advantageous, as self-reporting is prone to biases, while objective measures directly assess neurophysiological and behavioral responses in real-time (Rosanne et al., 2021).

**Materials and methods:**
Nine experienced liver surgeons participated in this study, chosen meticulously from a specific target group to ensure the study's quality. All participants were recruited from the University of Oldenburg's department of visceral surgery at PIUS-Hospital, Oldenburg, and provided informed consent. The study received approval from both the University of Oldenburg's medical ethical committee (reference number: 2020-150) and the PIUS-Hospital staff council.
The background stimulus comprised two 50 ms sinusoidal tone pips with 5 ms rise/fall times. The standard tone (500 Hz) occurred 88% of the time, while the rarer target tone (1 kHz) had a 12% occurrence, both presented at 75 dB SPL with a one-second inter-stimulus interval. For the general experimental paradigms, Ghani et al. (2020) were used as a guide.
The study explored the impact of three conditions on focused attention during liver surgery planning: 1) AR headset, 2) VR headset, 3) PC with traditional radiological images. In the AR and VR conditions, participants viewed segmented 3D models alongside radiological images, while in the PC condition, they only had access to radiological images. Participants were instructed to use all available functions in their respective conditions for planning liver resections. After each condition the participants were asked to indicate their subjective perceived stress using the NASA-TLX questionnaire.
For the experiment, a mobile EEG amplifier was connected to a standard 10/20 EEG setup on a desktop computer. Two speakers were placed beside the computer, and participants stood two meters in front of them. Controllers were provided for VR interaction, while gesture control was used for AR.

**Results:**
Event-related potentials were seen mainly on the electrodes of the central row and the midline (AFz to Pz and F3/C3 as well as F4/P4) (Fig. 1). To statistically verify the differences in amplitude of the grand averages, the work of Handy (2005) was used as a guide. The intervals for P1, N1, and P2 were marked accordingly (Fig. 1(a)). The resulting peaks and latencies of each participant were then subjected to an rmANOVA with the factors of patient case, condition, electrode, and ERP components.

Abstracts – DCK 2024 – 141st Congress of the German Society of Surgery – Leipzig, April 24–26 • DOI 10.1515/iss-2024-2003
Innov Surg Sci 2024; 9 (Special Suppl 1): s18–s203

A significant difference was found between the ERP components ($F(1.718; 13.743) = 46.754$; $p < 0.001$). The tendencies observed in the grand averages were not confirmed by the statistical analysis. No significant differences were found between patient cases, conditions, and electrodes.

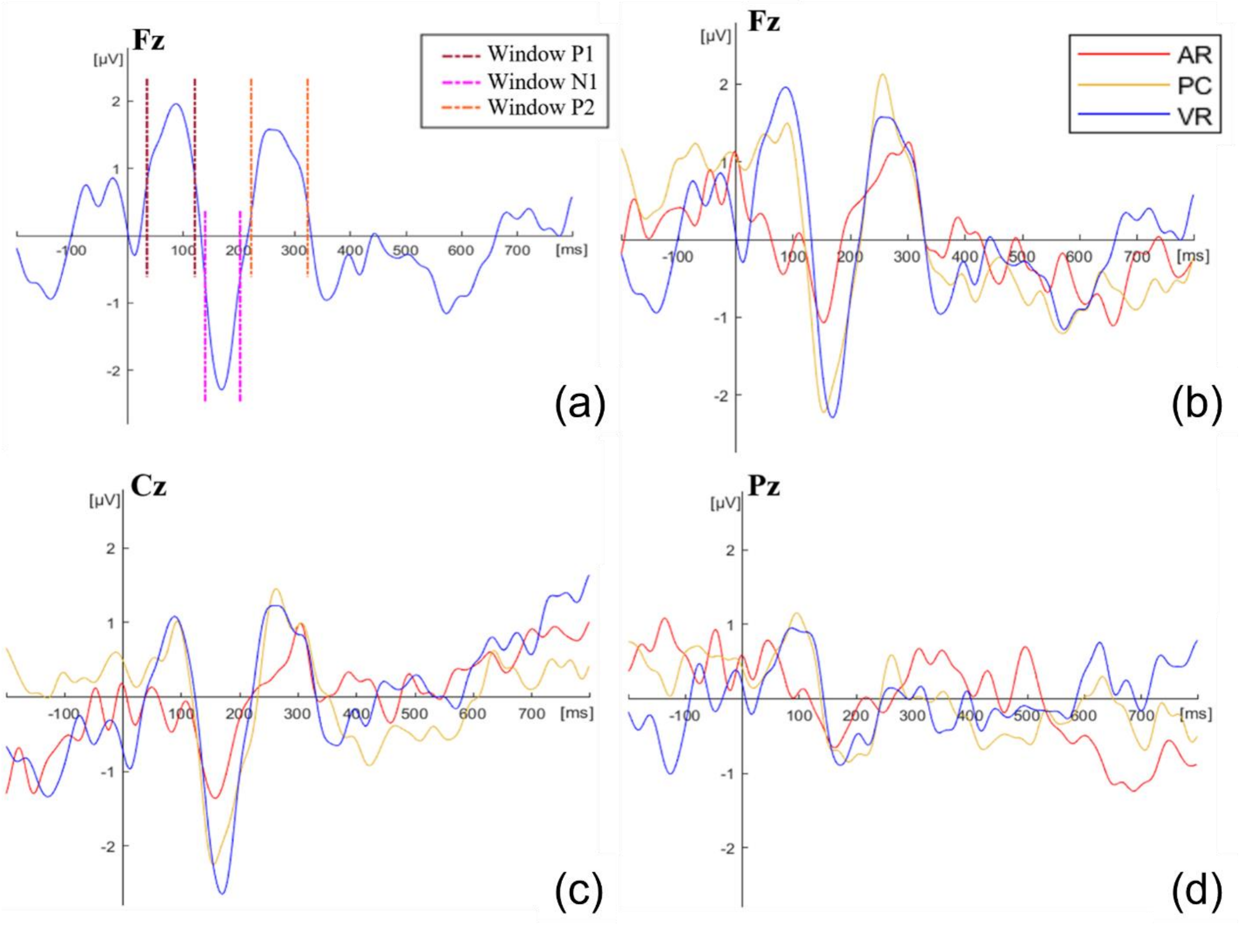

*Figure 3: Grand-Averages of Event-related potentials as response to the target tone for statistically analysed electrodes under all three conditions: (a): Temporal windows for the event-related components P1, N1 and P2, exemplarily shown for electrode position Fz; Grand-Averages of electrode Fz (b), Cz (c) and Pz (d).*

**Conclusion:**
We conclude that the use of a passive oddball paradigm is a feasible approach to measure the workload during surgical planning and thereby probably also during other medical applications when evaluating the strain, the use of AR or VR technology puts on the users' cognitive capabilities. The evaluated study design, using an oddball paradigm, proved to be a good opportunity to determine workplace strain objectively. In future studies, it may replace subjective questionnaires and enhance the significance of the measured workplace strain achieved by the objectivity provided.
Furthermore, we consider that especially the use of AR technology before the first use seems to require a certain amount of training, possibly due to the novelty of gesture control. However, we are confident, that with future developments in AR technologie and gesture recognition, those issues will become irrelavant in the future. In any case, the differences in workload between AR and VR highlight the need for intuitive user interaction and ease of use for any new technology, especially when applied in a medical setting.

**References**
[1} Bogdanova, Rositsa; Boulanger, Pierre; Zheng, Bin (2016): Depth Perception of Sur-geons in Minimally Invasive Surgery. In: Surgical innovation 23 (5), S. 515–524. DOI: 10.1177/1553350616639141.

[2] Dick, Jochen; Gebhard, Florian (2015): Zukunft der intraoperativen Bildgebung und Navigation. In: OP-JOURNAL 30 (03), S. 188–195. DOI: 10.1055/s-0034-1383340.
[3] Funk, Johannes; Schmidt, Ludger (2021): Evaluation of an Augmented Reality In-struction for a Complex Assembly Task. In: i-com 20 (1), S. 63–72. DOI: 10.1515/icom-2021-0005.
[4] Ghani, Usman; Signal, Nada; Niazi, Imran Khan; Taylor, Denise (2020): A novel ap-proach to validate the efficacy of single task ERP paradigms to measure cogni-tive workload. In: International journal of psychophysiology : official journal of the International Organization of Psychophysiology 158, S. 9–15. DOI: 10.1016/j.ijpsycho.2020.09.007.
[5] Handy, Todd C. (Hg.) (2005): Event-related potentials. A methods handbook. Cam-bridge, Mass.: MIT Press (A Bradford book).
[6] Kramme, Rüdiger (Hg.) (2011): Medizintechnik. Verfahren - Systeme - Informations-verarbeitung. 4. Aufl. 2011. Berlin, Heidelberg: Springer Berlin Heidelberg. On-line verfügbar unter http://nbn-resolving.org/urn:nbn:de:bsz:31-epflicht-1592699.
[7] Rosanne O, Albuquerque I, Cassani R, Gagnon J-F, Tremblay S and Falk TH (2021) Adaptive Filtering for Improved EEG-Based Mental Workload Assessment of Ambulant Users. Front. Neurosci. 15:611962. doi: 10.3389/fnins.2021.611962

**Is the Incidence of Low Anterior Rectum Syndrome (LARS) After Robotic Resections lower than the Laparoscopic ? A Retrospective Cohort Study**
ID: 252

Mazen Haydar[1], Nikolozi Labadze[1], Hagen Rudolph[1], Lutz Mirow[1]

[1]*Klinikum Chemnitz gGmbH, Klinik für Allgemein- und Viszeralchirurgie, Chemnitz, Deutschland*

**Background:**
Low Anterior rectum syndrome (LARS) is a multifactorial set of postoperative complaints that may develop after low anterior resection (LAR) surgery and can significantly affect the patient's quality of life.
The assumption that robotic surgery offers higher accuracy and more exact surgical dissection, which would result in better preservation of the autonomic pelvic nerves and therefore a lower incidence of LARS symptoms in comparison to laparoscopically performed LAR, should result in a lower incidence in LARS syndrome.
Hereby we represent our own institutional outcomes in comparing incidence of LARS after Robotic versus laparoscopic approaches.

**Materials and methods:**

We carried out a retrospective cohort study of all the minimally invasive performed rectal resections that took place from 21/07/2020 until 19/01/2023 in our institution. Exclusion criteria included patients that have bowel discontinuity in the form of protective ileostomy, transversostomy, known preoperative anal incontinence or postoperative anastomotic leakage.
The included patients completed the postoperative LARS Emmertson et al scoring questionnaire after a minimum of three postoperative months either at the follow-up visit or via telephone.

**Results:**
A total of 78 patients were included, 35 of which underwent the robotic LAR (group1) and 43 underwent laparoscopic LAR (group 2).
Of the 43 patients, which were operated laparoscopically, 25 had a tumour disease, 18 suffered from diverticular disease. The group of robotically operated patients (n=35) encompassed 14 patients with a tumour disease and 21 patients with a diverticular disease.
LARS score ranged in group 1 from 0 to 38 and in group 2 from 0 to 39.
The Null hypothesis assumed an identical distribution of the LARS score in the two groups. An Independent-Samples Mann-Whitney U Test was undertaken to avoid the bias accompanied with the assumption of a normal distribution of the two groups.
The Null hypothesis was rejected with a p-value for a two-tailed Mann-Whitney U test of 0.002. The significance level (α level) was set at 0.05 (5%).

**Conclusion:**
The incidence of LARS in group 1 with a mean rank of 30,86 who were operated robotically, was lower than the incidence of LARS in group 2 with a mean rank of 46,53 compared to patients who were operated laparoscopically. Thus we are presenting our results in support of the assumption of better postoperative outcomes in robotic versus laparoscopic LAR.
We will conduct further follow-up assessments of our patients, including LARS score evaluations at 6 and 12 months. These results will help confirm or refute our current findings
n future publications, we will evaluate various factors, including tumor invasion depth, neoadjuvant radiochemotherapy, and levels of oncological invasion, to assess their influence on LARS syndrome as we continue to observe our increasing number of patients.

**Comparison of the robotic-assisted and open surgical method for the development of postoperative sarcopenia in oncological esophageal and rectal resections**
ID: 321

Miriam Müller-Oerlinghausen[1], Felix Merboth[1], Heiner Nebelung[2], Andreas Bogner[1], Johannes Fritzmann[1], Marius Distler[1], Jürgen Weitz[1], Johanna Kirchberg[1]

[1]*Klinik und Poliklinik für Viszeral-, Thorax- und Gefäßchirurgie, Universitätsklinikum Carl Gustav Carus an der Technischen Universität Dresden*, [2]*Institut und Poliklinik für Diagnostische und Interventionelle Radiologie, Universitätsklinikum Carl Gustav Carus an der Technischen Universität Dresden*

**Background:**
Sarcopenia is a known risk factor for perioperative complications and poor oncologic outcomes. To date, few data are available to examine the impact of robotic versus open surgical methods on the development of postoperative sarcopenia in esophageal and rectal resections.

**Materials and methods:**
In the first part of the study, we analyzed the development of sarcopenia in the first 12 postoperative months in 168 patients undergoing robotic (RAMIE) or open esophagectomy (OE) after propensity score matching [1]. Sarcopenia was assessed by skeletal muscle index (SMI, cm2/m2) and psoas muscle thickness per height (PMTH, mm/m) on axial computed tomography scans in the first postoperative year.
In the second part of the study, we compared the development of postoperative sarcopenia in 94 patients after open and 55 patients after robotic oncologic rectal resection.

**Results:**
After 1-to-1 propensity score matching, 67 patients were assigned to the RAMIE or the OE group. SMI was significantly lower in the OE group compared to the RAMIE group at the third (43.2 ± 7.6 cm2/m2 vs. 49.1 ± 6.9 cm2/m2, $p = 0.001$), sixth (42.7 ± 7.8 cm2/m2 vs. 51.5 ± 8.2 cm2/m2, $p < 0.001$), and ninth (43.0 ± 7.0 cm2/m2 vs. 49.9 ± 6.6 cm2/m2, $p = 0.015$) postoperative months. Analogous results were found for PMTH. Preliminary analyses show first evidence for improved survival of patients with lower sarcopenia after esophagectomy at short follow-up of 11 months.
For rectal resection, initial analyses showed no significant differences in SMI and PMTH in the first 12 postoperative months between the robotic and open groups (SMI 6 months postoperatively 42,17 ± 8,18 $cm^2/m^2$ versus 42,10 ± 9,47 $cm^2/m^2$ , $p = 0.97$; SMI 12 months postoperatively .44,67 ± 8,57 $cm^2/m^2$ versus 42,36 ± 9,91 $cm^2/m^2$, $p = 0.75$).

**Conclusion:**
Our study demonstrates a previously unknown advantage of the robotic compared with the open surgical method for esophagectomy in terms of reduced postoperative sarcopenia. These results provide further evidence for the use of the robotic procedure in the multimodality therapy of esophageal cancer.
Regarding rectal cancer, further analyses are needed to determine whether advantages of the robotic surgical method may be demonstrable at the lower gastrointestinal tract for subgroups as at the upper gastrointestinal tract.

*1. Merboth, F., et al., Robotic Esophagectomy Compared With Open Esophagectomy Reduces Sarcopenia within the First Postoperative Year: A Propensity Score-Matched Analysis. J Thorac Oncol, 2023. 18(2): p. 232-244.*

Abstracts – DCK 2024 – 141st Congress of the German Society of Surgery – Leipzig, April 24–26 • DOI 10.1515/iss-2024-2003
Innov Surg Sci 2024; 9 (Special Suppl 1): s18–s203

**Effectiveness and Feasibility of Short-Course Simulator Training for Robotic Surgery Novices – A Randomized Controlled Trial (FastSim)**
ID: 323

Sortirios Emmanouilidis[1], Tina Groß[2], Benjamin Müssle[1], Anna Klimova[3], Thilo Hackert[2], Thilo Welsch[2]

[1]*Klinik für Allgemein-, Viszeral- und Thoraxchirurgie, St. Elisabethenklinikum Ravensburg, Oberschwabenklinik*, [2]*Klinik für Allgemein-, Viszeral- und Thoraxchirurgie, Universitätsklinikum Hamburg-Eppendorf*, [3]*Core Unit for Data Management and Analytics, National Center for Tumour Diseases (NCT), Dresden*

**Background:**
As robot-assisted surgery (RAS) is on the rise, a feasible and effective simulator training for surgical residents is required. Given the absence of a widely established training program, this study compares the effectiveness and feasibility of two simulator training regimens for preparing RAS novices.

**Materials and methods:**
In this randomized controlled trial, RAS novices were randomized 1:1 into a conventional arm (CONV), which included 4 repetitions of 5 simulator exercises in two sessions, and a fast training arm (FAST), with two repetitions of the same 5 simulator exercises in a single training session. The exercises were carefully designed to equip all participants with the basic skills required for a subsequent final exercise on the DaVinci skills simulator (dVSS). The primary endpoint was the mean efficiency score achieved in the final exercise.

**Results:**
Fifty-two participants (22 males) were randomized. The FAST/CONV participants were characterized by: age 30–50 years (n=12/13). no prior laparoscopic experience (n=14/12), general surgery background (n=12), and residents or consultants (n=11/12), respectively. The primary endpoint results showed a mean efficiency score of 48.2 ± 26.7 (FAST) versus 52.3 ± 30.4 (CONV) in the intention-to-treat analysis (p=0.527) and 48.19 ± 26.13 (FAST) versus 52.42 ± 31.06 (CONV) in the per-protocol analysis (p=0.473). There were no significant differences in penalty score (-54.73 ± 32.21 [FAST] versus -54.96 ± 31.81 [CONV], p=0.955) or overall score (19.19 ± 24.67 [FAST] versus 23.32 ± 27.07 [CONV], p=0.696). The most significant efficiency and penalty score improvement was observed between the first and fourth repetition in the CONV arm.
Interestingly, participants aged over 50 years and consultants performed worse compared with younger participants, students and resident doctors. Participants interested in robotic surgery outperformed those with little or no interest. The majority of all participants believed that more training would have yielded in an improved performance test. Stress levels, concentration, and exhaustion were similar in both groups.
FAST arm participants completed a training session in approximately 61 minutes (without any interruption), while in the CONV arm participants required around 92 minutes (with 9.6% of interruptions during the training sessions). The interest of performing robot-assisted surgeries in the future was higher in both arms after training.

**Conclusion:**
A training of 10 –20 exercises on the dVSS with ascending levels is insufficient to succeed in complex simulation exercises. Experienced surgeons do not require less training compared with students or residents. Simulation training is feasible and motivating. The findings emphasize the need for tailored training programs and continuous skill development in robotic surgery for diverse participant profiles.

### Early Prediction of Postsurgical Infections Using a Bayesian Approach

ID: 386

Hugo Armando Guillen-Ramirez[1], Daniel Sanchez-Taltavull[1], Sarah Peisl[1], Stéphanie Perrodin[1], Karen Triep[2], Olga Endrich[2 3], Guido Beldi[1]

[1]*Universitätsklinik für Viszerale Chirurgie und Medizin, Inselspital Bern, Bern, Schweiz*, [2]*Medical Directorate, Medizincontrolling, Inselspital, Universitätsspital Bern, Bern, Schweiz*, [3]*Department of Clinical Chemistry, Inselspital, Universitätsspital Bern, Bern, Schweiz*

**Background:**

Postoperative infections significantly affect morbidity and mortality rates, underscoring the need for improved predictions of infection risks. Traditional methods for evaluating preoperative phenotypes and monitoring inflammation markers postoperatively typically analyse individual parameters in a static context. Our research investigates the utilization of Bayesian approaches to develop a more dynamic and holistic model, integrating multiple parameters and examining their temporal variations concurrently. We hypothesize that a model integrating perioperative laboratory and CRP values will better predict postoperative infections than using a singular value such as CRP alone.

**Materials and methods:**

This study performed a retrospective examination of data that were prospectively amassed from patients subjected to surgical procedures at our institution within the timeframe of January 2014 to September 2022 and for whom perioperative laboratory values were available. The primary endpoint was postoperative infection. A series of machine learning algorithms were initially evaluated; subsequently, a Bayesian approach was utilized on the training dataset, constituting 80% of the total data, aiming to establish a risk of infection score. This derived score was subsequently assessed and validated utilizing the remaining 20% of the dataset.

**Results:**

A total of 59,758 patients who underwent 75,245 surgeries across all surgical departments within one University hospital were included. Our approach detected alterations that facilitated the earliest predictions of infection, for instance, CRP changes between postoperative day 0 and day 2 in visceral surgery. The CRP curves for each patient were meticulously characterized, and signal processing algorithms were applied to discern clusters of characteristic curves that are indicative of infection outcomes. Further, a collection of clinical features was integrated into a multidimensional model to visualize and understand the cluster-specific infection risks more comprehensively.

**Conclusion:**

By integrating multidimensional datasets through Bayesian models, we can identify predictors with greater precision compared to conventional multiple regression analysis, attaining up to 80% in both precision and recall. This methodological advancement yields clinically pertinent information, facilitating more prompt detection of postoperative complications, thereby allowing timely intervention and management.

Abstracts – DCK 2024 – 141st Congress of the German Society of Surgery – Leipzig, April 24–26 • DOI 10.1515/iss-2024-2003
Innov Surg Sci 2024; 9 (Special Suppl 1): s18–s203

**ChatGPT vs. Surgeons on Pancreatic Cancer Queries: Accuracy & Empathy Evaluated by Patients and Experts.**
ID: 512

Rosa Klotz[1 2], Thomas Pausch[1], Jörg Kaiser[1], Maximilian Joos[1], Rüdiger Hecktor[2], Azaz Ahmed[3], Colette Doerr-Harim[1], Arianeb Mehrabi[1], Martin Loos[1], Susanne Roth[1], Christoph Michalski[1], Christoph Kahlert[1]

[1]*Universitätsklinikum Heidelberg, Chirurgische Klinik - Allgemein-, Viszeral- und Transplantationschirurgie, Heidelberg, Deutschland*, [2]*Studienzentrum der Deutschen Gesellschaft für Chirurgie, Heidelberg*, [3]*Universitätsklinikum Heidelberg, Medizinische Onkologie (NCT), Heidelberg, Deutschland*

**Background:**
Artificial intelligence (AI), including ChatGPT, offers potential support in clinical tasks and patient-clinician interactions, but its impact on such communication remains unexplored.

**Materials and methods:**
ChatGPT was compared with two experienced pancreatic surgeons in responding to ten pancreatic cancer surgery-related questions, co-designed with the Patient Advisory Board of the Study Center of the German Surgical Society. A blind evaluation of these responses, considering content congruency and clarity for non-specialists, was conducted by a group of patients and surgeons. Additionally, patient representatives assessed empathetic resonance.

**Results:**
From June 23 to July 21, 2023, a cohort comprising 24 patients and 25 surgeons participated in the evaluation. Of this group, ten surgeons and 11 patients thoroughly completed all survey items. Utilizing a quantitative scale from 1 (representing strong disagreement) to 5 (signifying full agreement), consensus was observed among both patients and specialists concerning the content delivered by ChatGPT and the engaged surgeons. The metrics for comprehensibility to a non-specialist audience consistently showed positive reception across all feedback. Moreover, in the evaluation of empathetic resonance, ChatGPT's responses mirrored those of the surgeons in the patient's view. A significant proportion of both the patient and surgeon populations ranked Surgeon 1's contributions foremost, followed closely by ChatGPT.

**Conclusion:**
This study demonstrates that the responses from both surgeons and ChatGPT to common queries from patients regarding pancreatic cancer surgery are comparable regarding reliability, lay comprehension and empathy as evaluated by patients and surgical experts. These findings highlight the promising potential of AI in enhancing patient-provider interactions.

**Virtual and interactive 3D vascular reconstruction prior to planned pancreatic head resection and complex vascular anatomy: A bench-to-bedside transfer of virtual reality**
ID: 678

Robert Templin[1], Verena Uslar[1], Dirk Weyhe[1]

[1]*Pius-Hospital Oldenburg, Allgemein- und Viszeralchirurgie, Oldenburg, Deutschland*

**Background:**
Abstract: Bühler's anastomosis (also known as Bühler's arcade) is an embryonic relic and represents an arterio-arterial connection between the superior mesenteric artery and the celiac trunk. It can be found as a variety in 1-3% of patients.

**Materials and methods:**
We present a case in which a patient with metatastatic squamous cell carcinoma of the lung has been in stable disease for 4 years under palliative therapy (most recently second-line therapy with Nevolumab). A locally advanced adenocarcinoma of the papilla vateri has now also been diagnosed. Besides the patient had to undergo right hemicolectomy and patch plasty of the celiac trunk and superior mesenteric artery due to colonic ischemia and arteriosclerotic disease with 50-70% stenosis of the superior mesenteric artery several years ago. For the planning of a pylorus-preserving pancreatic head resection and reconstruction according to Traverso-Longmire, the preoperative imaging was extended by two independent 3D-vascular reconstructions (3D-print and 3D VR representation).

**Results:**
The Bühler anastomosis was detected in an out-of-hospital digital 3D reconstruction. This was created as an off-label use in a research project with the Frauenhoferinstitut Bremen (VIVA TOP). In the actual clinical CT-angiography, this variant had not been described. Intraoperatively, the presence of Bühler's anastomosis was confirmed. It had to be spared to avoid possible subsequent bowel ischemia.

**Conclusion:**
Increasing digitalization in surgical and perioperative preparation, in addition to its special requirements, holds great potential for better planning and improved patient safety.

**Serious Games for Training of Complications in the Context of surgical Laparoscopy**
ID: 705

Sophie-Caroline Schwarzkopf[1]

[1]*Viszeral-, Thorax- und Gefäßchirurgie, Universitätsklinikum Carl Gustav Carus Dresden, Dresden, Deutschland*

**Background:**
The complete surgical resection of the tumor represents the crucial therapeutic option in visceral surgery. Intra- and postoperative complications occur in up to every second operation. However, the potential risk, severity, and impact can be significantly reduced through early detection, accurate assessment, and timely initiation of appropriate therapy. To register early warning signs of intra- and postoperative complications, it is important to train clinical staff to be attentive.
Serious gaming is an innovative teaching method that combines gameplay with knowledge acquisition, increased concentration, and rapid decision-making. This project aims to improve the management of intra- and postoperative complications during visceral surgical procedures by developing and validating a serious gaming application for clinical staff. The application's use ranges from training students to educating hospital personnel.

**Materials and methods:**
The serious game for training of complications in the context of surgical laparoscopy will be screen-based and will be created based on previously collected videos of laparoscopic surgeries from the Department of Visceral, Thoracic, and Vascular Surgery at the Carl Gustav Carus University Hospital in Dresden, which have already been annotated.
The participant will see a three-part screen, including the corresponding video and a still image from the video sequence, along with questions in the format of a serious game. The questions are designed to match the participant's level of training. For example, students may be asked about visible organs, while experienced surgeons may be asked about the next steps in the operation, potential complications during the demonstrated surgical step, visible complications, and their management.

**Results:**
At the time of submission, specific results from the particular study cannot be presented since data collection will only begin in the fall of this year. However, from previous serious gaming studies, it can be demonstrated that the use of serious gaming is widely embraced within the surgical community and holds the promise of fostering continuous and consistent game-based learning through realistic case scenarios. This, in turn, contributes to the training and education of medical staff. The distinctive strengths of this learning format lie in its close resemblance to real-life situations, accessibility, and the up-to-date medical information it provides.
We anticipate a similar positive response in the context of this application and expect that the serious game will be promising both for preparing surgeons for operations and for general educational purposes among hospital staff.

**Conclusion:**
The purpose of the serious gaming application is to facilitate continuous and consistent learning through game-based approaches, utilizing realistic case scenarios. This aims to enhance the training and education of medical personnel. There are plans for the application's future integration into standard teaching and professional development. Insights gathered about potential challenges in the application development process could prove valuable in refining similar digital teaching and learning formats. Collectively, these formats have the potential to not only increase knowledge among participants but also benefit their patients, who will now be attended to by staff that is both sensitized and well-trained.

**Robotic anatomic liver resections: robotic sectionectomy versus robotic hemihepatectomy**
ID: 734

Emrullah Birgin[1 2], Marie Heibel[2], Patrick Téoule[2], Christoph Reißfelder[2], Nuh Rahbari[1 2]

[1]*Universitätsklinikum Ulm, Klinik für Allgemein- und Viszeralchirurgie, Ulm, Deutschland*,
[2]*Universitätsmedizin Mannheim, Chirurgische Klinik, Mannheim, Deutschland*

**Background:**
In the last decades, robotic and laparoscopic hepatectomies have been rapidly evolving. Although many hepatobiliary centers implemented laparoscopic hepatectomies as the standard of care for the treatment of both benign and malignant liver diseases, there is still some reluctancy to perform robotic hepatectomies globally. The treatment of deep lesions close to hepatic pedicles are challenging and often requires anatomic sectionectomy or hemihepatectomy. Therefore, there is an ongoing debate among liver surgeons to perform sectionectomy or hemihepatectomy if both resection techniques yield comparable perioperative outcomes and in particular negative resection margins. The aim of the present study was to analyze the safety of robotic sectionectomy (RS) versus robotic (extended) hemihepatectomy (RH).

**Materials and methods:**
All consecutive patients who underwent robotic hepatectomies between March 2021 and July 2023 were extracted from our prospectively collected institutional database. We included patients with RS (i.e., right anterior sectionectomy, right posterior sectionectomy, central hepatectomy, and left lateral sectionectomy) and (extended) RH, while patients with non-anatomic resections, segmentectomies, or other segment combinations were excluded. All patients underwent a pure robotic approach with a five-trocar technique including four robotic and one assist ports. Demographic data, perioperative outcomes and long-term outcomes were analyzed and compared between both groups. Statistical significance was set at a p-value of < 0.5.

**Results:**
During the study period, a total of 30 patients with a median age of 68 (58 – 77) years and a median BMI of 25 (23 – 28) kg/m$^2$ were included. Of these, 19 (63%) were males and 11 (37%) were females. Most patients underwent hepatectomy for secondary liver malignancies (53%). Sixteen patients underwent RS, and fourteen patients had RH. Among the study groups, the baseline characteristics were comparable. We found no significant differences in the length of postoperative stay, operative time, perioperative transfusions, and morbidity rates among the study groups. In the RS group, the duration of Pringle maneuver was significantly longer. Posthepatectomy outcomes in line with the International Study Group of Liver Surgery (ISGLS) were comparable between the study groups. All patients had negative resection margins. After a median follow-up of 11 months (4 – 17), two patients died in the early postoperative period due to cardiac arrest while 28 patients were alive. Nine patients with liver malignancies developed recurrent disease.

**Conclusion:**
Robotic hepatectomies are safe and associated with appropriate oncological outcomes. Sectionectomies are a safe alternative to major hepatectomies.

**Robotic surgery in children – indications, benefits and limitations. A 2-year single center experience**
ID: 742

Martin Lacher[1], Gabriel Goetz[1], Maria Moormann[1], Lena Bode[1], Richard Gnatzy[1], Richard Wagner[1], Jan-Hendrik Gosemann[1]

[1]*Klinik- und Poliklinik für Kinderchirurgie, Universitätsklinikum Leipzig, Leipzig, Deutschland*

**Background:**
Robotics is applied safely and successfully in a variety of adult surgeries and is gradually implemented in pediatric operations. The biggest challenge is to adapt the large robotic platform, originally designed for adults, to the small dimensions of children. The aim of this study was to analyze our pediatric robotic surgery program in children, which we started in 10/2021.

**Materials and methods:**
We assessed patients undergoing robotic procedures using the da Vinci XI surgical system (Intuitive Surgical, Inc., Sunnyvale, CA) from 10/2021- 09/2023. Analyzed parameters included surgical pathology, procedures, age, weight, conversion rate, operative time, and intra- and postoperative complications.

**Results:**
From 10/2021- 09/2023 a total of 40 children underwent primary robotic assisted laparoscopic surgery. Thirty-one procedures (78%) were urologic cases: pyeloplasty (N=10), robot-assisted laparoscopic extravesical ureteral reimplantation (RALUR; N=10), nephrectomy (N=5), hemi-nephrectomy (N=4), ovarian sparing excision of ovarian teratoma (N=1), nephroblastoma (Wilms-tumor) excision (N=1). Nine children (22%) underwent general surgery procedures: hemi-splenectomy (N=1), choledochal cyst excision & roux-Y choledochojejunostomy (N=1), fundoplication (N=4), abdominoperineal pull-through for Hirschsprungs disease (N=3). 58% of all patients were female. Mean age at surgery was 5.1 years (range: 15 months -17 years), median weight at surgery was 19.2kg (range: 9kg- 73kg). The mean console time was 92 min (range 84-118 min) for ureteral reimplantation (RALUR) and 94 min for pyeloplasty (range 63-117 min). The mean overall operative time (including docking + skin closure) was 119 min (range 105-171 min) for ureteral reimplantation (RALUR) and pyeloplasty was 143 min (range 112-165 min). None of the cases were converted to conventional laparoscopy. No intraoperative complications were experienced. One child after RALUR was readmitted with bladder emptying problems, which was self-limiting one month later.
According to our experience the main benefits of robotics over conventional MIS in children include stable magnified view, improved dexterity, better surgeon ergonomics, and motion scaling which makes suturing easier suturing in narrow spaces. Disadvantages remain lack of haptic feedback, increased costs and unfavorable cosmesis by using 8mm versus 3mm trocars.

**Conclusion:**
Our experience shows that technical and procedural skills of pediatric MIS can be transferred safely to the robotic approach. The main indication in our center are complex reconstructive urologic procedures. The lower weight limit seems to be 8-9 kg body weight in order to place trocars in an adequate distance. Docking times need to be reduced by increased training. We expect that with increased availability of robotic system, this technology will also drive the future in Pediatric Surgery.

**Radiomics and AI-based risk stratification in complex visceral surgery. Example of the preoperative fistula risk score (preFRS) in pancreatic surgery.**
ID: 806

Fiona Kolbinger[1 2], Julia Lamprecht[3], Stefanie Speidel[1 4], Jürgen Weitz[1 2 4], Ralf Hoffmann[3], Jens Kühn[1], Marius Distler[1 4]

[1]*Dresden, Department of Visceral, Thoracic and Vascular Surgery, University Hospital and Faculty of Medicine Carl Gustav Carus, Technische Universität Dresden, Dresden, Deutschland*, [2]*Dresden, Else Kröner Fresenius Center for Digital Health (EKFZ), Technische Universität Dresden, Dresden, Deutschland*, [3]*Dresden, Institute and Polyclinic for Diagnostic and Interventional Radiology, University Hospital Carl Gustav Carus Dresden, TU Dresden, Dresden, Deutschland*, [4]*, Division of Translational Surgical Oncology, National Center for Tumor Diseases (NCT), Partner Site Dresden, Dresden, Deutschland*

**Background:**
Risk stratification in complex visceral surgery is crucial to improve patient short- and long-term outcomes. Current risk stratification models mostly rely on intra- and postoperative data. Preoperative prediction of clinically relevant postoperative pancreatic fistula (CR-POPF), can augment surgical decision-making processes and may lead to better perioperative patient management.

**Materials and methods:**
We explore statistical and computational methods to predict CR-POPF from preoperatively available routine data. Frequency of CR-POPF and occurrence of salvage completion pancreatectomy during the hospital stay were associated with an intraoperative surgical (sFRS) and image-based preoperative CT-based (rFRS) fistula risk score, both considering pancreatic gland texture, pancreatic duct diameter and pathology, in 195 patients undergoing pancreatic head resection. Based on its association with fistula-related outcome, radiologically estimated pancreatic remnant volume was included in a preoperative (preFRS) score for POPF risk stratification. Moreover, we investigated machine learning-based approaches to predict CR-POPF from segmented CT imaging and clinical data points.

**Results:**
Intraoperatively assessed pancreatic duct diameter ($p < 0.001$), gland texture ($p < 0.001$) and high-risk pathology ($p < 0.001$) as well as radiographically determined pancreatic duct diameter ($p < 0.001$), gland texture ($p < 0.001$), high-risk pathology ($p = 0.001$), and estimated pancreatic remnant volume ($p < 0.001$) correlated with the risk of CR-POPF development. PreFRS predicted the risk of CR-POPF development (AUC = 0.83) and correlated with the risk of rescue completion pancreatectomy. In our subsequent radiomics approach, the risk prediction model based on four radiomic features and clinical data yielded similar results (AUC: 0.86).

**Conclusion:**
The results of the study are an examples that indicates that preoperative data can be useful for an accurate risk assessment in complex visceral surgery (e.g. CR-POPF).

**Is Bilateral Robotic-Assisted TAPP Superior to Laparoscopic TAPP? - A Randomized Controlled Trial.**
ID: 845

Ahmed Elsheikh[1 2], Ahmed Abdelsamad[1 2], Mike Langenbach[2]

[1]*Evangelisches Krankenhaus Lippstadt, Allgemein- und Viszeralchirurgie, Lippstadt, Deutschland*,
[2]*Universität Witten/Herdecke, Witten, Deutschland*

**Background:**
This study aims to compare the medical and economic outcomes of bilateral inguinal hernia repair using laparoscopic and robotic techniques concerning the duration of the operation, postoperative pain, use of analgesics, duration of hospital stay, rate of conversion to open procedure, carbon dioxide consumption, and the costs of the operation and hospital stay.

**Materials and methods:**
After approval of the ethics committee, the study will be conducted on 50 patients who will undergo bilateral inguinal hernia repair between October 2023 and October 2024. The patients will be divided into two groups: laparoscopic (n=25) and robotic (n=25).

**Results:**
The results are expected to show that the robotic technique for bilateral inguinal hernia repair is associated with shorter operative time, shorter length of hospital stay, higher quality of life, and higher patient satisfaction compared to the laparoscopic technique. However, it is currently assumed that robotic technique costs are notably higher than laparoscopic. According to our experience with robotic surgery, we suppose that in bilateral and especially complicated, combined, and recurrent inguinal hernias, due to the better visibility, precision, and dexterity of the robotic technique, the operating time decreases significantly so that robotic surgery costs become comparable to laparoscopic. Furthermore, patients benefit from reduced pain and fewer complications due to more precise work and less tissue trauma. Preliminary results will be processed and presented.

**Conclusion:**
Robotic bilateral hernia repair is a safe and feasible method. Yet it requires further investigation on a larger scale with other randomized multicentric studies and larger patient numbers.

Abstracts – DCK 2024 – 141st Congress of the German Society of Surgery – Leipzig, April 24–26 • DOI 10.1515/iss-2024-2003
Innov Surg Sci 2024; 9 (Special Suppl 1): s18–s203

**Are instrument activation patterns in Robotic Assisted Minimally Invasive Esophagectomy correlated to postoperative outcomes and surgeon factors?**
ID: 846

Jennifer Eckhoff[1 2], Dolores Thea Müller[2], Pieter de Backer[3], Wouter Bogaert[3], Yinan Xu[2], Guy Rosman[1], Ozanan Meireles[1], Alexandre Mottrie[3], Hans Fuchs[2]

[1]*Surgical Artificial Intelligence and Innovation Laboratory*, [2]*Uniklinik Köln, Klinik für Allgemein-, Viszeral-, Tumor- und Transplantationschirurgie*, [3]*ORSI Academy*

**Background:**
Robotic Assisted Minimally Invasive Esophagectomy (RAMIE) ranks among the most complex operations worldwide, with substantial mortality and morbidity rates. To leverage artificial intelligence (AI) for enhanced surgical and oncological safety in RAMIE a fundamental computer-vision based understanding of the individual components in the operating field is paramount. One pillar of surgical workflow recognition is instrument detection and tracking, which requires extensive spatial annotations and yields limited insight into instrument functionality. We hypothesize that on-screen information about instrument activation and use in robotic surgery can provide insights into procedure dynamics and clinical parameters pertaining to surgeon and patient factors. The objective of this study is to analyze instrument activation patterns in RAMIE.

**Materials and methods:**
We analyzed surgical video data from 32 RAMIE procedures performed by four surgeons with different levels of expertise in robotic esophageal surgery. We deploy an open source transformer model for text extraction from image using the on-screen display contrast to derive the log data of instrument activation (coagulation or cutting). We measured the average consecutive coagulation time per activation for each instrument and analyzed the clinical data pertaining to the RAMIE videos for postoperative outcomes classified by Clavien Dindo, surgeon expertise (Likert Scale 1-4) and patient characteristics (BMI).

**Results:**
The average duration of the analyzed RAMIE procedures was 2:38 hours. There was a distinctive activation pattern across the commonly used instruments in RAMIE, indicative of resection and reconstruction phases (Fig.1). The mean consecutive coagulation time (ACT) was 1.04 seconds per activation, with a median of 0.99 seconds. Less experienced surgeons displayed a lower ACT than experienced surgeons (Fig.2.A). With respect to Clavien Dindo classification, no correlation to ACT could be seen (Fig.2.B).

**Conclusion:**
On screen visual contrast information can be used to deduct instrument activation patterns in RAMIE, without the need for extensive spatial annotations of instruments. As access to sufficient volume and diversity of kinematic data and system logs is restricted, leveraging available visual contrasts from the robotic user interface provides additional insight into instrument usage. We will use regression analyses to evaluate the relations between clinical parameters and coagulation patterns and deploy temporal neural networks to extract statistical features about coagulation time signals to further explore dependencies between activation patterns and surgical expertise.

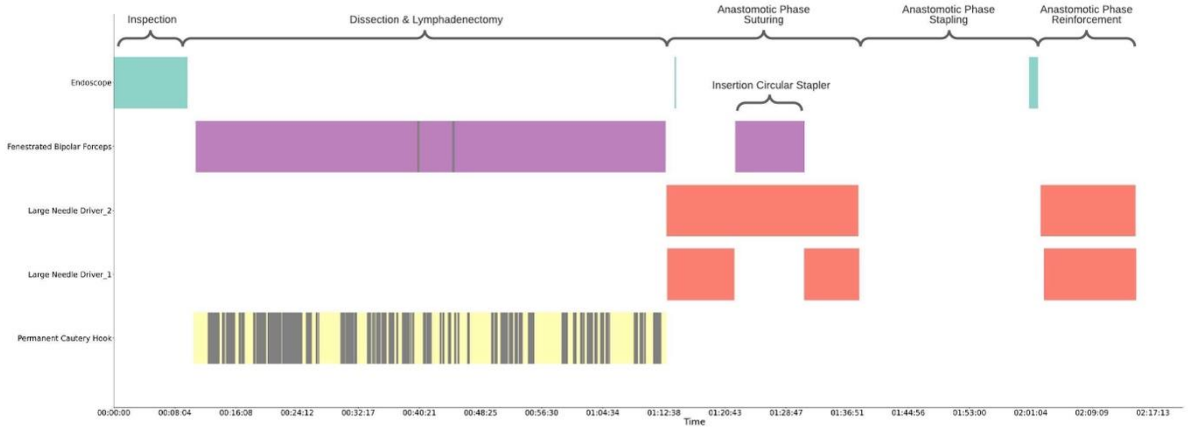

Figure 1: Characteristic instrument activation pattern in Robotic Assisted Minimally Invasive Esophagectomy, indicative of surgical phases

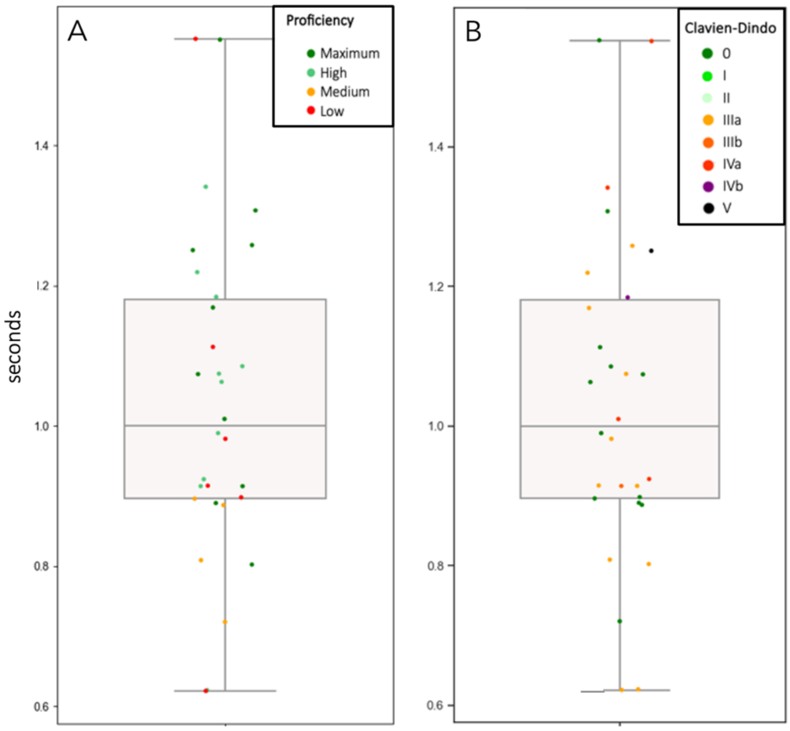

Figure 2: Average coagulation time per instrument activation correlated with (A) surgeon proficiency (B)postoperative complications (Clavien Dindo)

**The use of ChatGPT in surgery: evaluating its ability in responding patient questions**
ID: 848

Sebastian Lünse[1], Eric Wisotzky[2], Sophie Beckmann[2], Christoph Paasch[1], Richard Hunger[1], René Mantke[1]

[1]*Universitätsklinikum Brandenburg an der Havel, Klinik für Allgemein- und Viszeralchirurgie, Brandenburg an der Havel, Deutschland*, [2]*Fraunhofer-Heinrich-Hertz-Institut, HHI, Vision and Imaging Technologies, Berlin, Deutschland*

**Background:**
The artificial intelligence-based large language model ChatGPT® (OpenAI, San Francisco, USA) has taken human-machine interaction to the next level since its launch in November 2022. As a program that mimics human conversations over a text-based communication interface, ChatGPT has the potential to be used in a variety of healthcare settings, including patient information. The present pilot study aimed to assess the abilities of ChatGPT in responding patient questions in surgery.

**Materials and methods:**
A total of 30 commonly asked questions by patients about appendectomy, cholecystectomy, and inguinal hernia repair were submitted to ChatGPT (version 3.5) on September 12, 2023. The responses were assessed by experienced general and visceral surgeons using a modified global quality scale.

**Results:**
The study included 23 participating surgeons. The mean score for ChatGPT generated responses to patient questions was 4.04±0.45 (mean±standard deviation). Less than 20% of the respondents had prior experience with the use of artificial intelligence in clinical practice. The majority of participating surgeons (>80%) indicated that they would use ChatGPT to inform their patients in the future after appropriate further development.

**Conclusion:**
The artificial intelligence-based large language model ChatGPT has enormous potential to become a useful tool for information of patients in surgery. However, future implementation of ChatGPT into surgical practice should be done with caution and surgeons should consider it as a useful addition to their clinical experience and expertise.

**Prediction of long-term survival of patients with esophageal cancer and the effect of tumor biomarkers on predictive performance using machine and deep learning**
ID: 882

Leandra Lukomski[1], Juan Pisula[2], Naita Wirsik[1], Alexander Damanakis[1], Jin-On Jung[1], Karl Knipper[1], Rabi Datta[1], Wolfgang Schröder[1], Florian Gebauer[3], Thomas Schmidt[1], Alexander Quaas[4], Katarzyna Bozek[2], Christiane Bruns[1], Felix Popp[1]

[1]*Uniklinik Köln, Klinik und Poliklinik für Allgemein-, Viszeral-, Tumor- und Transplantationschirurgie, Köln, Deutschland*, [2]*Universität zu Köln, Center for Molecular Medicine Cologne (CMMC), Köln, Deutschland*, [3]*Helios Universitätsklinikum Wuppertal, Klinik für Allgemein-, Viszeral- und Onkologische Chirurgie, Wuppertal, Deutschland*, [4]*Uniklinik Köln, Institut für Pathologie, Köln, Deutschland*

**Background:**
Artificial Intelligence (AI) techniques such as Machine (ML) and Deep Learning (DL) recognize patterns in complex, big data. We use these methods to predict the long-term survival of patients with resectable esophageal cancer (EC). Moreover, we test whether AI methods are capable to predict survival at the time of the initial diagnosis (primary staging), how the addition of biomarker analysis in model training affects predictive performance, and whether biomarkers are equivalent to the Tumor, Node, Metastasis (TNM) classification in survival predictions.

**Materials and methods:**
We included 1002 patients with EC, 55 pre- and postoperative patient characteristics, and 55 immunohistochemically assessed biomarkers after surgical treatment between 1996 and 2021 for this retrospective study. We labeled the dataset by survival: Short-term survival (overall survival, OS: > 90 days but < 5 years + recorded death, n = 596) and long-term survival (OS: ≥ 5 years, n = 406). We trained four AI methods (Random Forest RF, XG Boost XG, Artificial neural network ANN, TabNet TN) and Logistic Regression (LR) for prediction of five-year survival status on an independent hold-out set (n = 100). We used the entire dataset including TNM und two data subsets without TNM for model training. The first data subset comprised of features of the medical history collected until primary staging (PS dataset). For the second data subset, we additionally included biomarkers to the PS dataset (PS dataset + biomarkers). We performed AI-driven feature selection to detect important variables for predictions.

**Results:**
Model training on the entire dataset produced comparable predictive performances for RF, XG, and ANN (Accuracy, ACC: 0.77/0.76/0.76/0.74/0.69; AUC: 0.80/0.83/0.84/0.78/0.73 RF/XG/ANN/TN/LR, respectively). LR was outperformed by the AI models. When models were trained with the PS dataset, predictive performance deteriorated. Surprisingly, when biomarkers were included to the PS dataset for model training, predictions improved and were comparable to model performances after training with the entire dataset (PS dataset versus PS dataset + biomarkers; ACC: 0.70 vs. 0.77/ 0.73 vs. 0.79/ 0.71 vs. 0.75/ 0.69 vs. 0.72/ 0.63 vs. 0.66; AUC: 0.77 vs. 0.83/ 80 vs. 85/ 76 vs. 86/ 70 vs. 76/ 70 vs. 69 RF/XG/ANN/TN/LR, respectively). We found following, important feature after AI-driven feature selection in all models after training with the entire dataset: pN status, pT status, p16 deletion, and Her2/neu amplification. After training with the PS dataset including biomarkers, following important predictive features appeared in all models: patient age at the time of surgery, TP-53 mutation, Mesothelin expression, TYMP expression, NANOG expression, IDO expressed on tumor-infiltrating lymphocytes, tumor infiltrating mast- and NK-cells.

**Conclusion:**
AI methods predict the long-term survival status of patients with EC and outperform LR, the state-of-the-art classification model. Survival status can be predicted with comparable predictive performance with patient data at an early stage of treatment if additional information of the tumor tissue is available. This suggests that individual predictions early in cancer treatment are possible with biomarkers and do not necessarily rely on the pathological TNM status after surgery. This study identifies important features for survival predictions that differ depending on the time of oncological treatment.

Abstracts – DCK 2024 – 141st Congress of the German Society of Surgery – Leipzig, April 24–26 • DOI 10.1515/iss-2024-2003
Innov Surg Sci 2024; 9 (Special Suppl 1): s18–s203

**Intestinal Dysbiosis as an intraoperative predictor of septic complications: Evidence from Human Surgical Cohorts and Mouse Model of Peritoneal Sepsis**
ID: 79

Daniel Spari[1], Simone Zwicky[1], Bahtiyar Yilmaz[1], Lilian Salm[1], Daniel Candinas[1], Guido Beldi[1]

[1]*Inselspital, Universitätsspital Bern, Department of Visceral Surgery and Medicine, Bern, Schweiz*

**Background:**
Major surgery exposes the intestinal microbiota to inflammatory, antibiotic, and other stressors, which alter the microbiota composition of the intestinal lumen and fecal contents, defined as intestinal dysbiosis (1–4). Intestinal dysbiosis is characterized by a reduction of obligate anaerobic bacteria such as Bacteroidota and Firmicutes and an increase of facultative anaerobic bacteria such as Proteobacteria (5). The Proteobacteria phylum includes the facultative pathogens *E. coli* and *Klebsiella spp*, which are often found in organs and infected wounds after surgery (6). As many infectious complications have its onset during rather than after surgery, it remains critical to determine, if dysbiosis develops already during surgery (7). We therefore investigated changes in the rectal microbiota immediately at the end of major surgery and compared the results to the individual preoperative baseline in two patient cohorts: i) patients that underwent rectal resection with a high risk for septic complications and ii) patients that underwent duodenopancreatic resection that lacked manipulation of the large intestine. Dysbiosis found in our patient cohorts was then modelled in mice and assessed in a cecal ligation and puncture model (CLP) as representative for septic surgical complications.

**Materials and methods:**
Before and directly at the end of rectal and duodenopancreatic resection, the microbiota composition of rectal specimens was analysed using 16S-rRNA sequencing. From the duodenopancreatic resection cohort, wound samples in case of superficial surgical site infection were analysed using 16S-rRNA sequencing. Intestinal dysbiosis was then modelled in mice using single oral administration of 0.15 mg vancomycin. To assess dynamic changes, feces was sampled over time and analysed using 16S-rRNA sequencing as well as Pacbio long-read sequencing to obtain strain level resolution. To assess septic complications, untreated (CTL) and vancomycin-treated (VAN) mice underwent CLP and sepsis score as well as cellular and cytokine immune response was assessed. Functional changes in microbiota community were assessed using PICRUSt2 pipeline.

**Results:**
Patients who underwent rectal and duodenopancreatic surgery both exhibited intestinal dysbiosis already at the end of surgery, which was characterized by an increase in Proteobacteria. Intestinal dysbiosis is of transient nature, since Proteobacteria have decreased to baseline levels at first postoperative follow-up. Wound samples that were derived from patients that underwent duodenopancreatic resection had low bacterial diversity and Proteobacteria were overtly abundant. Proteobacteria phylum was mainly composed of *Enterobacteriaceae* family, which includes the facultative pathogens *E. coli* and *Klebsiella spp*. Application of vancomycin in mice induced a transient increase in Proteobacteria, similar to changes observed in the patient cohorts and Proteobacteria phylum consisted mainly of *E. coli* species. In VAN mice, sepsis survival after CLP was impaired compared to CTL mice. The presence of *Enterobacteriacea* was the strongest predictor for impaired sepsis survival between these two groups. Cellular and cytokine response in the peritoneal fluid after CLP, was characterized by lower neutrophils count and cytokine dysregulation (IL-10, MCP-1, CXCL-1) in VAN mice. Functional analysis indicated increased virulence, altered signal transduction and metabolic changes in the microbiota of VAN mice.

**Conclusion:**
In the patient cohorts, intestinal dysbiosis emerged immediately but transiently at the end of surgery and was characterized by a strong increase in the Proteobacteria phylum, which mainly consisted of the *Enterobacteriaceae* family. In mice, single oral administration of vancomycin induced intestinal dysbiosis characterized by an expansion of Proteobacteria as in the patient cohorts, mainly consisting of *E. coli* species. The CLP sepsis model demonstrates significantly impaired survival and dysregulated cellular and cytokine immune response in dysbiotic mice. Furthermore, dysbiosis was associated with increased bacterial virulence, altered bacterial signaling and impaired bacterial metabolism of carbon sources, indicating a relationship between intestinal dysbiosis and outcome in patients undergoing major surgery.

1. Tarazi M, Jamel S, Mullish BH, Markar SR, Hanna GB. Impact of gastrointestinal surgery upon the gut microbiome: A systematic review. Surgery. 2022 May;171(5):1331–40.
2. Lauka L, Sobhani I, Brunetti F, Mestivier D, de'Angelis N. Human Colonic Microbiota and Short-Term Postoperative Outcomes in Colorectal Cancer Patients: A Pilot Study. Microorganisms. 2021 Dec 26;10(1):41.
3. Palmisano S, Campisciano G, Iacuzzo C, Bonadio L, Zucca A, Cosola D, et al. Role of preoperative gut microbiota on colorectal anastomotic leakage: preliminary results. Updat Surg. 2020 Dec;72(4):1013–22.
4. Schmitt FCF, Schneider M, Mathejczyk W, Weigand MA, Figueiredo JC, Li CI, et al. Postoperative Complications Are Associated with Long-Term Changes in the Gut Microbiota Following Colorectal Cancer Surgery. Life. 2021 Mar 16;11(3):246.
5. Lee JY, Cevallos SA, Byndloss MX, Tiffany CR, Olsan EE, Butler BP, et al. High-Fat Diet and Antibiotics Cooperatively Impair Mitochondrial Bioenergetics to Trigger Dysbiosis that Exacerbates Pre-inflammatory Bowel Disease. Cell Host Microbe. 2020 Aug;28(2):273-284.e6.
6. Lake JG, Weiner LM, Milstone AM, Saiman L, Magill SS, See I. Pathogen Distribution and Antimicrobial Resistance Among Pediatric Healthcare-Associated Infections Reported to the National Healthcare Safety Network, 2011–2014. Infect Control Hosp Epidemiol. 2018 Jan;39(1):1–11.
7. Mangram AJ, Horan TC, Pearson ML, Silver LC. Guideline for Prevention of Surgical Site Infection, 1999. 1999;27(2).

**Expression pattern of antimicrobial alpha- and beta-host defense peptides in appendicitis acuta - a prospective experimental study**
ID: 379

Steffen Axt[1], Carolina Girnstein[1], Jonas Johannink[1], André Mihaljevic[1], Andreas Kirschniak[2]

[1]*Tübingen, Klinik für Allgemeine, Viszeral- und Transplantationschirurgie, Tübingen, Deutschland*,
[2]*Maria Hilf Krankenhaus Mönchengladbach, Klinik für Allgemeine und Viszeralchirurgie, Mönchengladbach, Deutschland*

**Background:**
Appendicitis acuta has a lifetime prevalence of 7-8% and is the most common visceral surgical emergency with an incidence of 90-100 per 100 000 inhabitants per year in industrial countries. Despite this, the exact aetiology of the disease are not sufficient investigated especially the influence of host defence peptides, an essential part of the innate non-specific immune defence,
The objective of this study was to determine the expression pattern of the antimicrobial defense peptides, the so-called alpha and beta host defense peptides, in appendicitis acuta in order to clarify their influence on the aetiology.

**Materials and methods:**
Study population recruitment and sample acquisition of a total of 147 appendix full-wall samples was performed for further determination of the expression pattern of antimicrobial alpha- and beta-host defense peptides in appendicitis acuta. Values for HD 5, HD 6, hBD 1, hBD 2, hBD 3, and IL8 expression were determined.

**Results:**
Determination of the transcription level of interleukin 8 revealed a significant difference with p=<0.001 between simple and complex appendicitis. HD 5 expression demonstrated a significant reduction in transcripts between no acute appendicitis and acute appendicitis (p=0.002). A significant reduction in the transcription numbers of HD 6 expression was also detected between no acute appendicitis and acute appendicitis (p=0.006). For hBD 1, there were approximately significant differences in expression between no appendicitis and acute (p=0.053) or complex appendicitis (p=0.055). No significant difference could be demonstrated in the expression of HBD 2 between an inconspicuous appendix and appendicitis. There was also no significant difference in HBD 3 expression between an inconspicuous appendix and appendicitis (p=0.304).

**Conclusion:**
The changes in the expression patterns of the host defense peptides, especially alpha host defense peptides, may indicate that these may have an influence on the development of appendicitis acuta. Further studies should clarify the relationship of the expression patterns of the host defense peptides as a possible etiology of acute appendicitis.

**Successful temporary machine perfusion of marginal liver grafts early after retrieval or shortly before implantation**
ID: 45

Jan Georgi[1], Charlotte von Horn[1], Bastian Lueer[1], Laura Malkus[1], Thomas Minor[1]

[1]*Universitätsklinikum Essen, Essen, Deutschland*

**Background:**
The benefit of machine perfusion during storage of liver grafts retrieved after cardiac death should be investigated as applied either at the beginning or near the end of the preservation period.

**Materials and methods:**
Rat livers were explanted 20 min after cardiac arrest of the donor and cold-stored (CS) for 18h. Other grafts were additionally subjected to 2h of normothermic machine perfusion (MP) either 3h after retrieval (early MP) or 3 h before reperfusion (late MP), thus extending total ischemic time to 20h. The 3h period should represent a short transport period between a resident regional pumping center and the explant or implant hospital, respectively. Viability of all livers was assessed thereafter by warm reperfusion in vitro.

**Results:**
In comparison to the controls, both regimens significantly improved hepatic recovery upon post-preservation reperfusion as evaluated by enzyme release, bile production and energetic recovery. Molecular upregulation of pro-inflammatory signals was also significantly mitigated. No functional differences between early and late machine perfusion could be disclosed.

**Conclusion:**
In conclusion, our data suggest that it might not be necessary to hurry with the attempt to connect the graft to a machine early after retrieval.

**Preoperative C-reactive-protein-to-albumin ratio as a predictor of patient- and graft-survival after deceased-donor liver transplantation.**
ID: 181

David Erren[1], Franziska Meister[1], Karsten Große[2], Tom J.G. Gevers[3], Robert Siepmann[4], Daniel Truhn[4], Steven W.M. Olde Damink[5], Tom Lüdde[6], Zoltan Czigany[7], Theresa H. Wirtz[2], Tony Bruns[2], Florian Vondran[1], Iakovos Amygdalos[1]

[1]*University Hospital RWTH Aachen, Department of General, Visceral and Transplantation Surgery*, [2]*University Hospital RWTH Aachen, Department of Internal Medicine III*, [3]*Maastricht University Medical Center+, Division of Gastroenterology and Hepatology, Department of Internal Medicine*, [4]*University Hospital RWTH Aachen, Department of Diagnostic and Interventional Radiology*, [5]*Maastricht University Medical Center+, Department of Surgery*, [6]*University Hospital Düsseldorf, Department of Gastroenterology, Hepatology and Infectiology*, [7]*University Hospital Heidelberg, Department of General, Visceral and Transplantation Surgery*

**Background:**
Advances in immunosuppression and treatment of complications have led to favorable outcomes after deceased-donor liver transplantation (DDLT). However, various patient factors still affect long-term survival and graft loss. This study examines the predictive value of preoperative C-reactive-protein-to-albumin ratio (CAR) regarding recipient survival (RS) and graft survival (GS) after DDLT.

**Materials and methods:**
This retrospective study included DDLT recipients between 2010 and 2023, at the University Hospital RWTH Aachen. Patients with re-transplantations, living-donor, or split-liver transplantations and those dying within 90 days of transplantation were excluded. Predictive abilities of CAR were examined through receiver operating characteristic curve (ROC) analyses and cut-offs were defined using the area under the curve (AUC) and the Youden Index (YI). Differences in survival were compared using Kaplan-Meier analysis and the log rank test. Risk factors for RS and GS were analyzed with Cox regression.

**Results:**
A total of 444 patients were included, with a mean RS of 110 months (95% CI 105-116 months) and GS of 106 months (95% CI 100-112 months). A good predictive ability for CAR was shown for various survival and graft loss endpoints, with the best AUC being observed for 3-year RS (3YRS, AUC=0.67, $p<0.001$) and 3-year GS (3YGS, AUC=0.65, $p<0.001$). The Youden Index analysis identified an ideal cut-off of CAR=41% for both 3YRS (YI= 0.35) and 3YGS (YI=0.32). Patients with CAR≥41% had significantly lower mean 3YRS (30 vs. 34 months, $p<0.001$) and 3YGS (29 vs. 33 months, $p<0.001$). Cox regression analysis showed a hazard ratio of 3.78 (95% CI 2.19-6.53, $p<0.001$) for 3YRS and 3.11 (95% CI 1.93-5.01, $p<0.001$) for 3YGS, for patients with CAR≥41%.

**Conclusion:**
Higher preoperative CAR is associated with inferior long-term outcomes after DDLT, particularly 3-year recipient- and graft survival. This easily accessible bedside tool may serve as an additional method for identifying patients, who would benefit from optimization of physical and nutritional status whilst waiting for an organ, as well as closer follow-up after liver transplantation.

**440 Consecutive Hand-Assisted-Retroperitoneoscopic-Donor-Nephrectomies (HARP). A Comprehensive Analysis of the Intraoperative Challenges and Potential Pitfalls.**
ID: 238

Tristan Wagner[1], Marielle Hummels[1], Wasim Kamel[1], Christine Kurschat[2], Martin Kann[2], Rabi Raj Datta[1], Roger Wahba[1], Christiane J. Bruns[1], Dirk Ludger Stippel[1], Michael Thomas[1]

[1]*Universitätsklinikum Köln, Klinik und Poliklinik für Allgemein-, Viszeral- und Tumorchirurgie, Köln, Deutschland*, [2]*Universitätsklinikum Köln, Innere Medizin II - Nephrologie, Rheumatologie, Diabetologie und Allgemeine Innere Medizin, Köln, Deutschland*

**Background:**
This analysis highlights the various pitfalls and challenges encountered in living kidney transplantation. This study aims to evaluate the outcomes and trends within an established living kidney program, providing valuable insights into the effectiveness and advancements in this area of transplantation.

**Materials and methods:**
A retrospective analysis was conducted on a cohort of living kidney transplant recipients who underwent transplantation at a specialized german transplant center between 06/2010 and 06/2023. Relevant data, recipient demographics, donor characteristics, surgical details, post-transplant outcomes, and long- term follow-up, were extracted from prospective database. Statistical analyses were performed to assess graft survival, patient survival. Complications were categorized in vascular, urinary tract associated, device- and donor associated pit falls.

**Results:**
A total of 440 living kidney transplant procedures were included in the study. The mean age of the recipients was 52.8 ± 11 years, with a slight female predominance 58.5% female. All kidney grafts were taken out with hand assisted total retroperitoneous nephrectomy (HARP). 65% (286) were left kidneys. No conversion surgery had to be performed. Time of kidney explantation was 108.4 ± 27.9 min, time of the whole donation surgery 130 ± 27.5 min, warm ischemic time during kidney harvest was 122 sec ± 57, blood loss of the recipients was 49.2 ± 42 ml, 64 donors 16.4 % had multiple arteries and veins. In 228 (51.8%) cases potential pit falls were detected. 102 Vascular-(58.1%), 5 urinary tract- (2.2%), 46 donor- (25.9%) and 13 device associated pit falls (2.9%). If pit falls or deviating procedure were pointed out time of kidney explantation was longer 113 ± 28.52 p <0.001, warm ischemic time during kidney harvest was longer 143 ± 70 sec p <0.001 and blood loss was significant higher 60.1 ± 54.4 p<0.001.

**Conclusion:**
The importance of blood loss, warm ischemia time and operative time should not be underestimated especially in donors with multiple arteries and veins. By addressing these pitfalls, we can strive to improve the overall success and long-term outcomes of living donor renal transplantation and enhance the quality of life for transplant recipients.

**Three Decades of Liver Transplantation for Biliary atresia:Outcomes of 3721 Liver Transplants from CTS Registry**
ID: 375

Arash Nickkholgh[1 2], Christian Unterrainer[3], Caner Süsal[3], Arianeb Mehrabi[1], Hien Tran[3]

[1]*Ruprecht-Karls University of Heidelberg, Department of General, Abdominal and Transplant Surgery, and Liver Cancer Center Heidelberg,, Heidelberg, Deutschland*, [2]*Klinikum Ludwigshafen, Klinik für Allgemein-, Viszeral- und Thoraxchirurgie, Ludwigshafen am Rhein, Deutschland*, [3]*Ruprecht-Karls University of Heidelberg, Institute of Immunology, Heidelberg, Deutschland*

**Background:**
Biliary atresia (BA) is one of the most common indications for pediatric liver transplantation (LT). This registry study presents the challenges and achievements in the field of LT for BA within the last 30 years.

**Materials and methods:**
Data obtained from the Collaborative Transplant Study (CTS - www.ctstransplant.org) on all patients undergoing LT for BA performed from 1 January 1990 to 31 December 2019 in 69 centers from 22 countries were analyzed. Graft survival was defined as a combined endpoint, defined as the time from LT to either patient’s death or re-transplantation or last known contact (whichever came first).

**Results:**
Three thousand seven hundred and twenty-one LTs (1667 male, 2054 female) for biliary atresia were documented within three decades. Less than half of the recipients (1589, 42.7%) were under 12 months of age at the time of transplantation for biliary atresia, followed by children of age group 1-2 years old (1129, 30.3%). Most grafts came from deceased donors (2829, 76%); pediatric / adolescent donors comprised almost half (1290, 46%) of the deceased donor population. The most common graft types used in LT for biliary atresia were the split grafts from deceased donors (1646, 58%), followed by whole organs (1031, 36%), and grafts from living donors (892, 24%; female to male ratio 1.3). Reduced size (as well as otherwise anatomically modified) deceased donor grafts were used in only around 5% of LTs (n=150).
3298 (88.6%) were primary LTs; 423 (11.4%) were re-LTs. Re-LTs showed a down-trend to almost a half from 14.3% in 1990-1999 compared to 7.7% within 2010-2019. Being transplanted in 1990-1999, donor age older than 40 years (50 years in living donor LT), cold ischemia times longer than 10 hours (only in deceased donor LTs), ABO-incompatibility, high urgent LTs (only in deceased donor LTs), and Re-LTs were independent risk factors for graft loss.

**Conclusion:**
Excellent outcomes have been achieved through LT for BA patients over the last three decades with significantly improved 5-year survival within the last decade, where the survival difference between the deceased and the living donor LTs narrows, so that it loses the statistical significance.

**Comparative Analysis of Mono Hypothermic Oxygenated Machine Perfusion (mHOPE) and Dual Hypothermic Oxygenated Machine Perfusion (dHOPE) Versus Static Cold Storage (SCS) in Liver Preservation Prior to Transplantation**
ID: 549

Dionysios Koliogiannis[1], Moritz Drefs[1], Malte Schirren[1], Dominik Koch[1], Severin Jacobi[1], Elnaz Payani[1], Ganildo Cepele[1], Fabian Horné[1], Jens Werner[1], Markus Guba[1]

[1]*Klinik für Allgemein-, Viszeral- & Transplantationschirurgie - LMU München, München, Deutschland*

**Background:**
The advancement of machine perfusion techniques has introduced a new era of graft preservation and promises better transplant outcomes. In this study, we undertake a comprehensive comparative analysis of machine hypothermic oxygenated perfusion and traditional static cold storage (SCS) in liver graft preservation. In addition, we highlight the differences in the use of mono HOPE (mHOPE) and dual HOPE (dHOPE). Our study is based on our experience from late 2019 to present at the Munich site and represents the largest study addressing this question.

**Materials and methods:**
In this retrospective study, we evaluate the impact of mHOPE and dHOPE on liver transplant outcomes compared to the conventional SCS approach. Our study population includes 171 patients who received liver grafts preserved with HOPE (84 mHOPE & 87 dHOPE) and a propensity score-matched cohort of SCS-treated patients between November 2019 and September 2023. We analyze multiple parameters including ischemia/reperfusion injury, graft function, biliary complications, perioperative course, post-reperfusion syndrome, postoperative complications, ICU utilization, and hospitalization.

**Results:**
Our results from 171 liver grafts preserved with HOPE show that both mHOPE and dHOPE are superior regarding the incidence of ischemia/reperfusion injury compared with SCS (peak AST: 1794 U/l to 2877 U/l, $p < 0.001$; peak ALT 912 U/l to 1594U/l, $p < 0.001$, respectively). Better early and late graft function in the HOPE group did not result in a reduction of need and duration of intensive care treatment, but in the length of hospital stay (ICU-stay 7 vs. 9 days; hospital stay: 35.4 vs. 44.2 days). Although HOPE showed significant advantages in most end points compared to SCS-grafts, no significant differences in ischemia/reperfusion injury, early and late graft function, or incidence of post-reperfusion syndrome were found between the 2 modalities of HOPE maintenance. However, a slight trend in favor of dHOPE was observed, indicating a potential reduction in biliary complications.

**Conclusion:**
To our knowledge, this is the largest comparative analysis highlighting the superiority of HOPE over conventional SCS in graft preservation before liver transplantation. The significant reduction in ischemia/reperfusion injury, improved graft function, and reduced post-reperfusion syndrome underscore the pivotal role of HOPE in improving liver preservation techniques. Although there were no significant differences between the outcomes of the two HOPE modalities, our results argue for the adoption of machine perfusion as the standard procedure for organ preservation before liver transplantation.

**Sarcopenic obesity may predict worse liver regeneration after right graft living donor liver transplantation**
ID: 641

Aladdin Ali Deeb[1], Johannes Fritsch[1], Utz Settmacher[1], Falk Rauchfuß[1]

[1]*Universitätsklinikum Jena, Klinik für Allgemein-, Viszeral und Gefäßchirurgie, Jena, Deutschland*

**Background:**
Sarcopenic obesity impairs the outcome after liver transplantation. The effect of this on liver regeneration has not yet been studied. The aim of this study was to evaluate the potential effect of body composition changes on liver volume gain after living donor liver transplantation.

**Materials and methods:**
We observed liver regeneration in 100 patients who underwent living donor liver transplantation using right lobe grafts (Segments V-VIII). Liver volumetry and body composition analysis were performed based on computed tomography images with special software. The gain of liver volume was calculated between two points in time considering the absolute and percentage values: before surgery and early after surgery with a median time of 10 days. Pearson's correlation and multivariate analysis using stepwise multiple regression were used to examine the potential correlation between body composition and liver volume gain.

**Results:**
Liver volume increase was significantly negatively correlated with adipose tissue in the body stem (r = - 0.4, P < 0.001) and positively correlated with psoas mass (r = 0.24, P = 0.02). These results correspond with those of the multiple regression analysis, which indicated adipose tissue (ß = - 1.0, P < 0.001) and psoas mass (ß = 0.12, P < 0.001). The presence of malignancy as an indication for liver transplantation was another significant independent factor negatively affecting liver growth (ß = - 13.1, P = 0.046).

**Conclusion:**
Sarcopenic obesity predicts an impaired liver volume increase after living donation. This could worsen the postoperative outcome. The role of alimentary interventions and exercises in improving body composition and thus postoperative outcome should be evaluated through prospective interventional studies.

**Roboter-assistierte Lebendnierenspende – Eine Zentrumsanalyse von 107 Spendern**
ID: 803

Anne-Sophie Mehdorn[1], Robin Behrens[1], Malte Lang[1], Alexander Bernsmeier[1], Magdalena Kleinke[1], Kevin Schulte[2], Thomas Becker[1], Felix Braun[1], Jan Beckmann[1], Michael Linecker[1]

[1]*Universitätsklinikum Schleswig-Holstein, Klinik für Allgemeine, Viszeral-, Thorax-, Transplantations- und Kinderchirurgie, Kiel, Deutschland*, [2]*Klinik für Nephrologie, Kiel*

**Background:**
Aufgrund des weiterhin zunehmenden Spenderorganmagels mit einer mittleren Wartezeit auf eine Nierentransplantation von aktuell 10 Jahren in Deutschland, ist es unumgänglich Lebendnierenspendeprogramme mit einer möglichst geringen Morbidität und Mortalität zu etablieren. Die ursprünglich offene Spendernehprektomie ist heutzutage zugunsten eines besseren Komplikationsprofils und einem besseren postoperativen Ergebnis mittlerweile fast vollständig von minimalinvasiven Techniken abgelöst worden. Dazu gehören eine Reihe von chirurgischen Techniken, wie reine oder handassistierte, laparoskopische, retroperitoneoskopische oder transluminale endoskopische chirurgische Eingriffe über natürliche Öffnungen (NOTES). Ziel all dieser Techniken ist es die Morbidität der Operation für die Spender auf ein Minimum zu reduzieren und gleichzeitig eine optimale Nierentransplantatfunktion zu erreichen. Aktuell gilt die Roboter-assistierte Chirurgie als das präziseste und versatilste minimalinvasive Operationsverfahren. Nationale und internationale Erfahrungen an einer größeren Anzahl an konsekutiven Lebendnierenspendern ist jedoch gering. Ziel dieser Arbeit ist die systematische Analyse eines Roboter-assistierten Lebendnierenspendeprgrammms an einem norddeutschen Transplantationszentrum.

**Materials and methods:**
Alle konsekutiven Roboter-assistierten Lebendnierenspenden sowie die dazugehörigen Empfänger wurden in einer prospektiv geführten Datenbank an der Klinik für Allgemeine, Viszeral-, Thorax-, Transplantations- und Kinderchirurgie am Universitätsklinikum Schleswig-Holstein, Campus Kiel erfasst. Diese Eingriffe wurden von einer Gruppe an robotererfahrenen Chirurgen unter Anwendung eines speziellen Protokolls unter Verwendung der da Vinci-Roboterplattform durchgeführt. Primärer Endpunkt der Studie bestand darin, die technische Machbarkeit und das Sicherheitsprofil der Technik einschließlich der perioperativen chirurgischen Komplikationsrate und der mittelfristigen funktionellen Ergebnisse bei Spender und Empfänger zu bewerten.

**Results:**
Zwischen 2010 und 2022 wurden insgesamt 107 konsekutive Roboter-assistierte Spendernephrektomien durchgeführt. Die Spender waren mehrheitlich männlich (60%; n=64), 54 (±9) Jahre alt und hatten einen BMI von 26 (±4) kg/m$^2$. Vierundfünfzig Prozent (n=53) der Spender und Empfänger waren blutsverwandt, die restlichen Spender waren dem Empfänger emotional nahe stehend, wie Ehe- oder Lebenspartner oder Freunde. Es wurden etwas mehr rechte Spendernephrektomien (55%; n=57) als linke durchgeführt. Die Spendernephrektomien wurden vollständig Roboter-assistiert durchgeführt und dauerten im Mittel 178 (±58) min. Zwanzig Spendernieren (19%) verfügten über eine Mehrgefäßversorgung eines oder beider Gefäße. Konversionen waren nicht notwendig. Eine durch den Assisttrokar erzeugte Dünndarmdeserosierung, die mit einer Naht versorgt wurde, war die einzige intraoperativen Komplikation im Rahmen der Spendernephrektomien. Postoperativ kam es bei den Spendern zu fünf Komplikationen Clavien-Dindo Grad II (Wundinfektionen und –hämatome) und einer Komplikation Clavien-Dindo Grad IIIa (Lymphozele mit interventioneller Pigtailanlage). Im Mittel waren die Spender 4 (±2) Tage hospitalisiert. Das präoperative Kreatinin betrug 74 (±14) µmol/l; bei Entlassung 116 (±24) µmol/l. Das Kreatinin bei der letzten Nachsorge betrug 115 (± 25) µmol/l. Die Spender wurden durschnittlich 33 (± 39) Monate im Haus nachgesorgt und/oder zu heimatnahen Nephrologen überführt. Bei zwei Spendern wurde im Verlauf ein neu aufgetretener arterieller Hypertonus festgestellt, bei einem weiteren Spendern wurde eine postoperative Proteinurie und prädiabetische Stoffwechsellage detektiert. Alle neu aufgetretenen Nebenerkrankungen ließen sich konservativ behandeln und traten nach mehr als 90 Tagen nach der Nierenspende auf.
Die Transplantatempfänger waren durchschnittlich 47 (±16) Jahre alt, 56% (n=60) waren männlich und 72% (n=77) waren vor der Transplantation bereits dialysepflichtig gewesen. Die durchschnittliche Dialysedauer vor Transplantation betrug 25 (± 20) Monate. Die häufigsten Erkrankungen, die zur Niereninsuffizienz geführt hatten, waren autosomal-dominante polyzystische Nierenerkrankungen (n = 22, 20%), fokal segmentale Glomerulosklerose (n = 17 16%) und IgA-Nephropathie (n=14, 14%). Für 92% (n=98) der Patienten war die Lebennierenspende die erste Nierentransplantation. Die kalte Ischämiezeit betrugt 158 (±54) min und die warme Ischämiezeit 32 (±12) min. Siebenundneunzig Prozent (n=103) der Transplantate zeigten eine primäre Funktion; eine postoperative Dialyse war in zwei Fällen (2%), eine Plasmapherese in drei Fällen (3%) notwendig. Postoperative Komplikationen traten bei 65% (n=70) der Patienten auf. Dies beinhaltete Komplikationen Clavien-Dindo Grad I in 5 Fällen (5%), Grad II in 51 Fällen (48%), Grad IIIa in einem Fall (1%) und Grad IIIb in 6 Fällen (6%) sowie Grad IVa in einem Fall (1%). Die Empfänger waren im Durchschnitt 19 (±12) Tage hospitalisiert. Der Nachsorgezeitraum der Empfänger betrug im Mittel 64 (± 46) Monate. Kreatinin bei der letzten Nachsorge betrug 169 (±145) µmol/l, Harnstoff 11 (±7) mmol/l. Im Nachsorgezeitraum verloren 10 Transplantate (9%) ihre Funktion, die Ursachen hierfür waren chronische Abstoßungen (n=5), die Rekurrenz der Grunderkrankung (n=3) und Mikroangiopathien (n=2). Drei Transplantate (3%) wurden nach dem Funktionsverlust entfernt.

**Conclusion:**
Roboter-assistierte Lebennierenspenden konnten im vorliegenden konsekutiven Spenderkollektiv von 107 Patienten sehr sicher und komplikationsarm durchgeführt werden. Die mittel- und langfristige Nierenfunktion der Spender zeigte sich erfreulich stabil. Ebenso konnten die Spendernieren komplikationsarm transplantiert werden und zeigten im Nachbeobachtungszeitraum eine stabile Transplantatfunktion. Somit bietet aus unserer Sicht die roboter-assitierte Spendernephrektomie deutliche Vorteile für Spender und Operateur und ist in erfahrenen Händen gegenwärtig als Operationsmethode der Wahl zu betrachten.
